# The first high-altitude autotetraploid haplotype-resolved genome assembled (*Rhododendron nivale* subsp. *boreale*) provides new insights into mountaintop adaptation

**DOI:** 10.1093/gigascience/giae052

**Published:** 2024-08-07

**Authors:** Zhen-Yu Lyu, Xiong-Li Zhou, Si-Qi Wang, Gao-Ming Yang, Wen-Guang Sun, Jie-Yu Zhang, Rui Zhang, Shi-Kang Shen

**Affiliations:** Ministry of Education Key Laboratory for Transboundary Ecosecurity of Southwest China, Yunnan Key Laboratory of Plant Reproductive Adaptation and Evolutionary Ecology, Institute of Biodiversity, School of Ecology and Environmental Science, Yunnan University, Kunming 650504 Yunnan, China; Ministry of Education Key Laboratory for Transboundary Ecosecurity of Southwest China, Yunnan Key Laboratory of Plant Reproductive Adaptation and Evolutionary Ecology, Institute of Biodiversity, School of Ecology and Environmental Science, Yunnan University, Kunming 650504 Yunnan, China; Ministry of Education Key Laboratory for Transboundary Ecosecurity of Southwest China, Yunnan Key Laboratory of Plant Reproductive Adaptation and Evolutionary Ecology, Institute of Biodiversity, School of Ecology and Environmental Science, Yunnan University, Kunming 650504 Yunnan, China; Ministry of Education Key Laboratory for Transboundary Ecosecurity of Southwest China, Yunnan Key Laboratory of Plant Reproductive Adaptation and Evolutionary Ecology, Institute of Biodiversity, School of Ecology and Environmental Science, Yunnan University, Kunming 650504 Yunnan, China; School of Life Sciences, Yunnan Normal University, Kunming 650500 Yunnan, China; School of Life Sciences, Yunnan Normal University, Kunming 650500 Yunnan, China; Ministry of Education Key Laboratory for Transboundary Ecosecurity of Southwest China, Yunnan Key Laboratory of Plant Reproductive Adaptation and Evolutionary Ecology, Institute of Biodiversity, School of Ecology and Environmental Science, Yunnan University, Kunming 650504 Yunnan, China; Ministry of Education Key Laboratory for Transboundary Ecosecurity of Southwest China, Yunnan Key Laboratory of Plant Reproductive Adaptation and Evolutionary Ecology, Institute of Biodiversity, School of Ecology and Environmental Science, Yunnan University, Kunming 650504 Yunnan, China

**Keywords:** autotetraploid, evolutionary history, harsh environment, mountaintop adaptation, *Rhododendron*

## Abstract

**Background:**

*Rhododendron nivale* subsp. *boreale* Philipson et M. N. Philipson is an alpine woody species with ornamental qualities that serve as the predominant species in mountainous scrub habitats found at an altitude of ∼4,200 m. As a high-altitude woody polyploid, this species may serve as a model to understand how plants adapt to alpine environments. Despite its ecological significance, the lack of genomic resources has hindered a comprehensive understanding of its evolutionary and adaptive characteristics in high-altitude mountainous environments.

**Findings:**

We sequenced and assembled the genome of *R. nivale* subsp. *boreale*, an assembly of the first subgenus *Rhododendron* and the first high-altitude woody flowering tetraploid, contributing an important genomic resource for alpine woody flora. The assembly included 52 pseudochromosomes (scaffold N50 = 42.93 Mb; BUSCO = 98.8%; QV = 45.51; S-AQI = 98.69), which belonged to 4 haplotypes, harboring 127,810 predicted protein-coding genes. Conjoint *k-*mer analysis, collinearity assessment, and phylogenetic investigation corroborated autotetraploid identity. Comparative genomic analysis revealed that *R. nivale* subsp. *boreale* originated as a neopolyploid of *R. nivale* and underwent 2 rounds of ancient polyploidy events. Transcriptional expression analysis showed that differences in expression between alleles were common and randomly distributed in the genome. We identified extended gene families and signatures of positive selection that are involved not only in adaptation to the mountaintop ecosystem (response to stress and developmental regulation) but also in autotetraploid reproduction (meiotic stabilization). Additionally, the expression levels of the (group VII ethylene response factor transcription factors) *ERF VIIs* were significantly higher than the mean global gene expression. We suspect that these changes have enabled the success of this species at high altitudes.

**Conclusions:**

We assembled the first high-altitude autopolyploid genome and achieved chromosome-level assembly within the subgenus *Rhododendron*. In addition, a high-altitude adaptation strategy of *R. nivale* subsp. *boreale* was reasonably speculated. This study provides valuable data for the exploration of alpine mountaintop adaptations and the correlation between extreme environments and species polyploidization.

## Introduction

*Rhododendron* L. is the largest genus in Ericaceae and the largest woody plant genus in the Northern Hemisphere, with more than 1,000 species. It is also representative of the highly diverse Sino-Himalayan flora in East Asia, shaped by the topographic and climatic heterogeneity resulting from the uplift of the Qinghai–Tibet Plateau [1, 2]. Furthermore, *Rhododendron* is one of the few woody flowering species that is dominant in plant communities found within the delicate subalpine to alpine transition zone and presents a perfect opportunity to explore the mechanisms behind the evolution and adaptation of alpine woody plants [3, 4]. In *Rhododendron, Rhododendron nivale* subsp. *boreale* Philipson et M. N. Philipson is one of the few woody flowering plants discovered to be distributed at altitudes above 5,000 m and is one of the few polyploid (2n = 4x = 52) woody plants in the Qinghai–Tibet Plateau [1, 5, 6]. *R. nivale* subsp. *boreale*, a member of the subg. *Rhododendron*, is a small-leaved, highly branched shrub distributed at high altitudes of mountaintops (up to alt. 5,400 m) down to the mountainsides (∼3,200 m). This species demonstrates remarkable adaptability, as evidenced by its diverse habitats, including alpine meadows, forest edges, and metal mining areas [7]. Currently, *R. nivale* subsp. *boreale* is an important ornamental plant resource in mountainous plateau areas and is used in traditional Tibetan medicine [8, 9]. Therefore, exploring the evolutionary patterns and adaptation mechanisms of *R. nivale* subsp. *boreale* not only promotes the understanding of alpine adaptation evolution in woody plants but also establishes a basis for the commercial exploitation of high-altitude ornamental plants.

Genetic perspectives provide a better understanding of evolution and adaptive differentiation [10]. However, the paucity of genomic data is a significant impediment to research advancement [11]. For example, in a recent high-altitude adaptation study, only 7 alpine plant genomes were used, indicating that the genetic resources of alpine plants are far from sufficient compared to the diversity of the high-altitude flora [12]. Moreover, polyploidy likely enhanced the adaptability of alpine plants to harsh environments [13]. Unfortunately, acquiring polyploid genetic data remains challenging, particularly for autopolyploids with highly similar subgenomes. Currently, the assembly of autopolyploid genomes presents significant challenges, resulting in the publication of only a select number of such genomes, including those of *Medicago sativa, Saccharum spontaneum, Solanum tuberosum*, and *Rheum officinale* [14–17]. To further understand the evolution and adaptation of the alpine flora, additional genetic resources, particularly of polyploids, are essential.

Polyploidy has been theorized to be both a potential evolutionary roadblock and a catalyst for evolutionary breakthroughs and the proliferation of species [18]. On the one hand, following polyploidy events, rapid shifts in gene expression and epigenetic modifications can bestow the polyploid with an almost instant competitive edge, which is usually reflected in their broader geographical ranges compared with their diploid ancestors [19, 20]. Therefore, polyploidy tends to be ecologically advantageous and occurs in variable climatic regions, such as the Qinghai–Tibet Plateau alpine and pan-Arctic regions [21, 22]. Comprehending these adaptive mechanisms in high-altitude polyploids not only clarifies evolutionary dynamics but also provides insights into conservation strategies. On the other hand, auto- and allopolyploids face a significant obstacle: the accurate segregation of chromosomes during meiosis [23, 24]. In recent years, our understanding of the molecular basis for polyploid adaptations to meiotic challenges has significantly increased; however, compared to allotetraploids, little is known about the molecular mechanisms underlying the stabilization of autotetraploid meiosis [25]. Advances in molecular technology and, subsequently, more genetic resources will provide new insights into the survival, evolution, adaptation, and conservation of polyploids.

Here, we present a haplotype-resolved tetraploid genome of mountaintop plant *R. nivale* subsp. *boreale* from an altitude of 4,287.5 m, which is the first chromosome-level genome assembly of the subgenus *Rhododendron*. Based on this assembly, we identified polyploid types, deciphered whole-genome duplication (WGD) events, and investigated which genes or gene families are potential candidates involved in alpine mountaintop adaptation and the survival of polyploids. This genome not only establishes the groundwork for comprehending the evolution and adaptation of *Rhododendron* species but also offers valuable genetic resources to investigate the origin, recombination, and differentiation of polyploid species.

## Results

### Genome estimation, sequencing, and assembly

**Figure 1:**
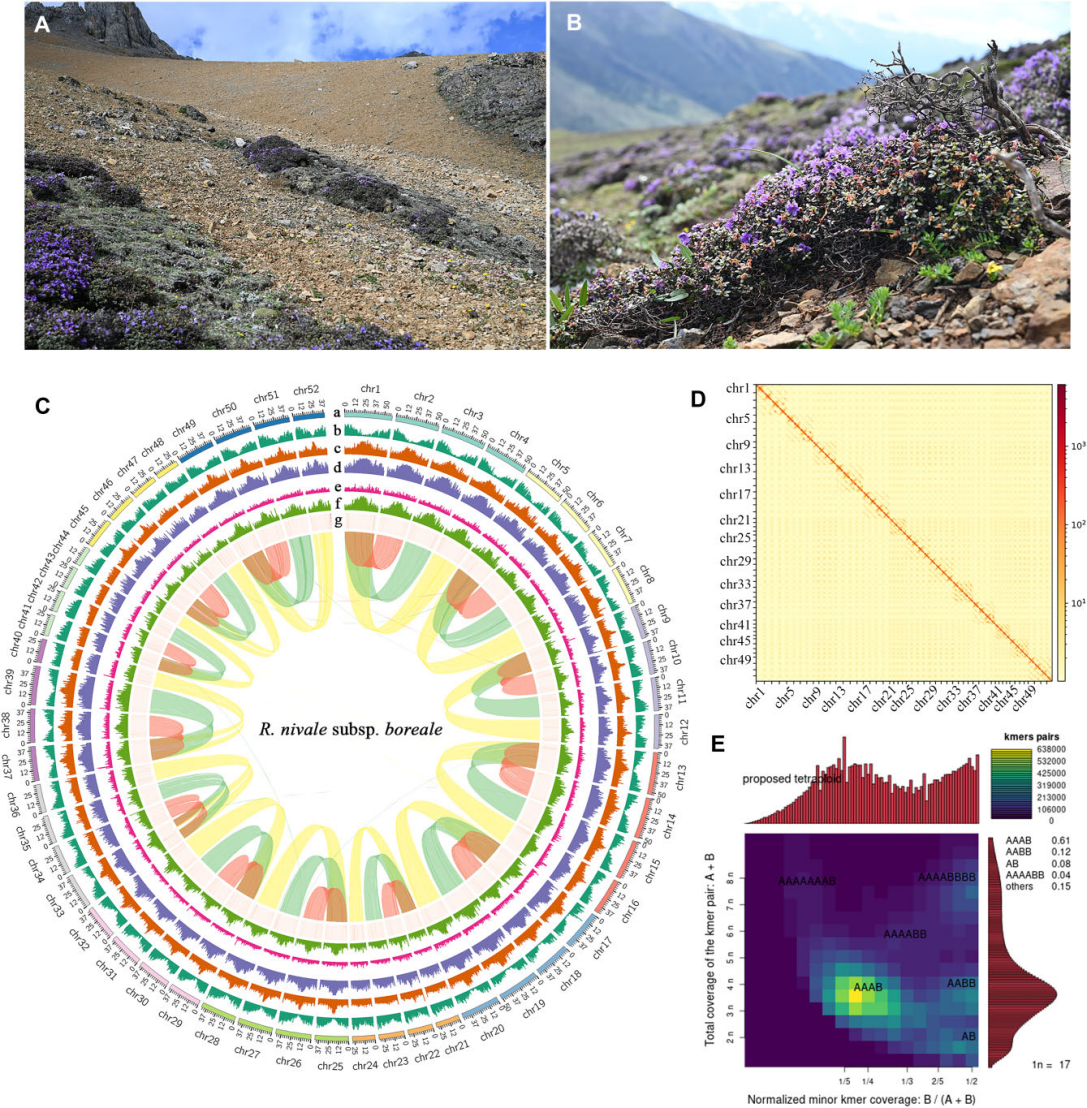
Habitat and genomic characteristics of *R. nivale* subsp. *boreale*. (A) Habitat. (B) Habit. (C) Genome landscape. a, 52 pseudochromosomes, which belong to 13 homologous groups, and the length of the pseudochromosome; b, gene density; c, GC density; d, transposon element density; e, copia density; f, gypsy density; g, tandem repeat density. Curved lines inside the circles link syntenic genes between different pseudochromosomes, the synteny between haplotype 1 and haplotype 2 is indicated in red, the synteny between haplotype 1 and haplotype 3 is indicated in green, and the synteny between haplotype 1 and haplotype 4 is indicated in yellow. (D) Hi-C heatmap for assembled pseudochromosomes. (E) Smudgeplot analysis based on 21 *k-*mers.

*R. nivale* subsp. *boreale* samples were collected from the alpine region at an altitude of 4,300 m, treated with liquid nitrogen, and sequenced (Fig. 1A). We obtained a total of 33.35 Gb of PacBio CCS long reads with an average length of 15.86 kb and an N50 length of 16.15 kb (Supplementary Table S1). A genome survey was performed based on DNB-seq short reads (95.61 Gb; Supplementary Table S2), and the result revealed an estimated genome size of 2.48 Gb, which was consistent with that estimated by flow cytometry (Supplementary Figs. S1, S2; Supplementary Table S3). This species was identified as a tetraploid based on *k-mer* analysis (Fig. 1E). The 3 initial assembly sizes assembled using Hifiasm, Canu v1.9, and HiCanu were 2.48 Gb, 2.39 Gb, and 2.40 Gb, respectively (Supplementary Tables S4, S8). The assembled version of Hifiasm was used for subsequent analysis, as it ensures higher integrity of both genes and long terminal repeats (LTRs). The long reads and next-generation sequencing (NGS) reads were mapped to the unitig-level assembly to assess the assembly quality. Long-read and whole-genome sequencing (WGS) reads were mapped to 99.87% and 99.59%, respectively, and RNA-seq reads exhibited mapping rates exceeding 94% (Supplementary Table S5). The average GC content was 41.10%. We used the AllHiC algorithm to improve the genome assembly to the chromosome level using 138.98 Gb of Hi-C data. After manual checking, a total of 2.17 Gb of unitigs were anchored to 52 pseudochromosomes (scaffold N50 = 42.93 Mb), ranging from 23.70 to 59.70 Mb in length and containing 4 haplotypes (13 pseudochromosomes per haplotype) (Fig. 1C; Supplementary Table S6). The Hi-C heatmap clearly showed the interactions of 13 homologous groups (Fig. 1D), with high similarity observed between the pseudochromosomes within each homologous group.

This genome was assembled with a high consensus quality value (QV = 45.51; error rate = 0.0028%) and high *k-*mer completeness (97.79%) (Supplementary Table S7). BUSCO assessment indicated that the completeness of the conserved embryophyte genes was 98.8% (Supplementary Table S8). The quality of the genome structure at the reference genome level (assembly quality indicators of large structural fragments; S-AQI = 98.69) was assessed using CRAQ (Supplementary Table S9).

### Annotation

A repeat sequence of 1,549,068,457 bp was identified, accounting for 62.63% of the genome assembly (Supplementary Table S10). The richest category of repeats was LTRs (43.12%), with *Gypsy* and *Copia* accounting for 33.66% and 5.88% of the repeats, respectively (Supplementary Fig. S3). In addition, the LTR assembly index (LAI) was greater than 14 (n1:14.78, n2:14.84, n3:14.35, and n4:14.38) based on LTR annotation, which indicated that the assembly met the reference category standards. By combining *ab initio*, homology, and transcriptome data predictions, 127,810 protein-coding genes were predicted, with an average gene length of 4,736.07 bp. The total length of the coding sequences (CDSs) was 148,274,600 bp, and the average number of CDSs per gene was 4.8 (Supplementary Table S11). The completeness of 98.6% of the annotated protein-coding genes of *R. nivale* subsp. *boreale* was assessed using BUSCO. Of the protein-coding genes, 96.86% were annotated functionally (Supplementary Table S12). The ratio of mono‐exonic (single‐exon) genes to multiexonic (multiple‐exon) genes was 0.245. We annotated 17,049 candidate noncoding RNAs, including 703 microRNAs (miRNAs), 3,672 transfer RNAs (tRNAs), 5,373 small nuclear RNAs (snRNAs), and 7,301 ribosomal RNAs (rRNAs) (Supplementary Table S13).

### Confirmation of autotetraploid

Polyploids are commonly found in plants. However, the origins of the polyploids differ and include both homologous and heterologous origins. Extensively studied allotetraploids such as peanuts, cotton, and wheat [26–28] exhibit significant subgenomic differences, allowing for their division into distinct subgenomes. In contrast to allotetraploids, the high similarity among haplotypes greatly increases the difficulty in assembling autotetraploids. To determine the polyploid type of *R. nivale* subsp. *boreale*, we employed *k-*mer analysis, collinearity analysis, and phylogenetic analysis for cross-validation. The 21 *k-*mer frequency analysis revealed 4 distinct peaks (located at 33, 68, 106, and 136) (Supplementary Fig. S2), which was highly similar to the results for autotetraploids (*M. sativa* and *S. spontaneum*). Nucleotide heterozygosity is an important criterion for determining polyploid types [29]. Nucleotide heterozygosity form analysis of *R. nivale* subsp. *boreale* showed 2.53% AAAB and 1.28% AABB (Supplementary Table S3), which was consistent with the expectation that the heterozygous rate of autotetraploid AAAB would be greater than that of AABB.

**Figure 2:**
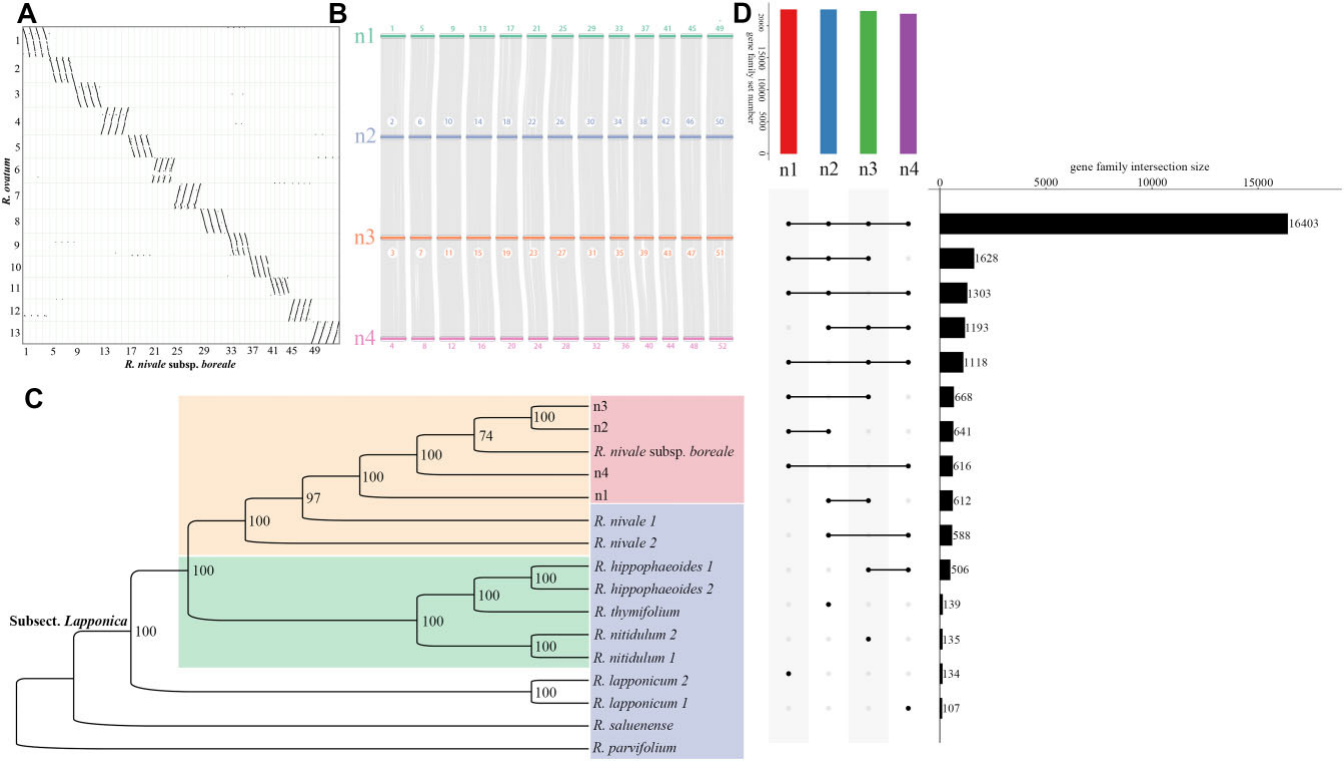
Phylogenetic and comparative analysis between related species and haplotypes. (A) Dot plot between *R. nivale* subsp. *boreale* and *R. ovatum*. (B) Syntenic blocks between 4 haplotypes. (C) Phylogenetic relationships of subsect. *Lapponica* based on the maximum likelihood (ML) analysis; yellow and green blocks show *R. nivale* and the sister clade of *R. nivale*, respectively; red block represents the data generated in this study (n1, n2, n3, n4, and *R. nivale* subsp. *boreale* represent the 4 haplotypes and transcriptome of *R. nivale* subsp. *boreale*, respectively), and the blue block represents downloaded species data. (D) Gene family characteristics between 4 haplotypes.

Nevertheless, these methods were insufficient to identify the polyploid type. For example, although genomic analysis indicated higher AAAB than AABB, *Artemisia argyi* was identified as an allotetraploid [30]. To further determine the polyploid type of *R. nivale* subsp. *boreale*, synteny analysis was performed based on syntenic blocks. As expected, the dot plot and syntenic blocks indicated synteny among the 4 haplotypes (Fig. 2A), with 20,172 gene pairs showing synteny between haplotypes 1 and 2, 20,249 between haplotypes 2 and 3, and 19,883 between haplotypes 3 and 4 (Fig. 2B, Supplementary Fig. S4). Additionally, we downloaded transcriptome data from 11 samples of 7 closely related species (Supplementary Table S14) to infer the phylogenetic positions of the 4 haplotypes of *R. nivale* subsp. *boreale* and identified a monophyletic group consisting of 6 species (*Rhododendron nitidulum, Rhododendron hippophaeoides, Rhododendron thymifolium, Rhododendron nivale, R. nivale* subsp. *boreale*, and *Rhododendron lapponicum*) of subsect. *Lapponica* with high support. A clade containing all *R. nivale* subsp. *boreale* (including the transcriptome and 4 haplotypes) and 2 *R. nivale* was supported by 100% bootstrapping (Fig. 2C).

Moreover, 25,791 orthogroups were identified in the 4 haplotypes, with 16,403 shared by all, 5,242 shared by 3, 3,631 shared by 2, and only 515 (n1:134; n2:139; n3:135; n4:107) unique to each haplotype genome, showing high genetic similarity among haplotypes (Fig. 2D). Overall, the combined results of *k-*mer analysis, collinearity analysis, and phylogenetic analysis indicated that *R. nivale* subsp. *boreale* is an autotetraploid species.

### Comparative analysis and recent polyploidization

The phylogenetic position and divergence times of *R. nivale* subsp. *boreale* were inferred from 18 other species, including 12 species of Ericales (10 *Rhododendron*), 2 species of Cornales, 1 species of Gentianales, 1 species of Vitales, 2 species of monocotyledons, and 1 sister species to all angiosperms (*Amborella*). Altogether, 666,442 genes were used to infer orthology. A total of 625,661 genes (93.9%) clustered into 37,844 orthologous gene families, of which 6,547 were shared across all species (Fig. 2B; Supplementary Table S15). In total, 209 single-copy gene families were identified. In total, 146 gene families, comprising 369 genes, were found to be specific to *R. nivale* subsp. *boreale* (Supplementary Fig. S5). These species-specific genes were enriched in 12 KEGG pathways and 136 Gene Ontology (GO) terms, including arginine biosynthesis, nitrogen metabolism, and flavonoid biosynthesis (Supplementary Tables S16, S17).

**Figure 3:**
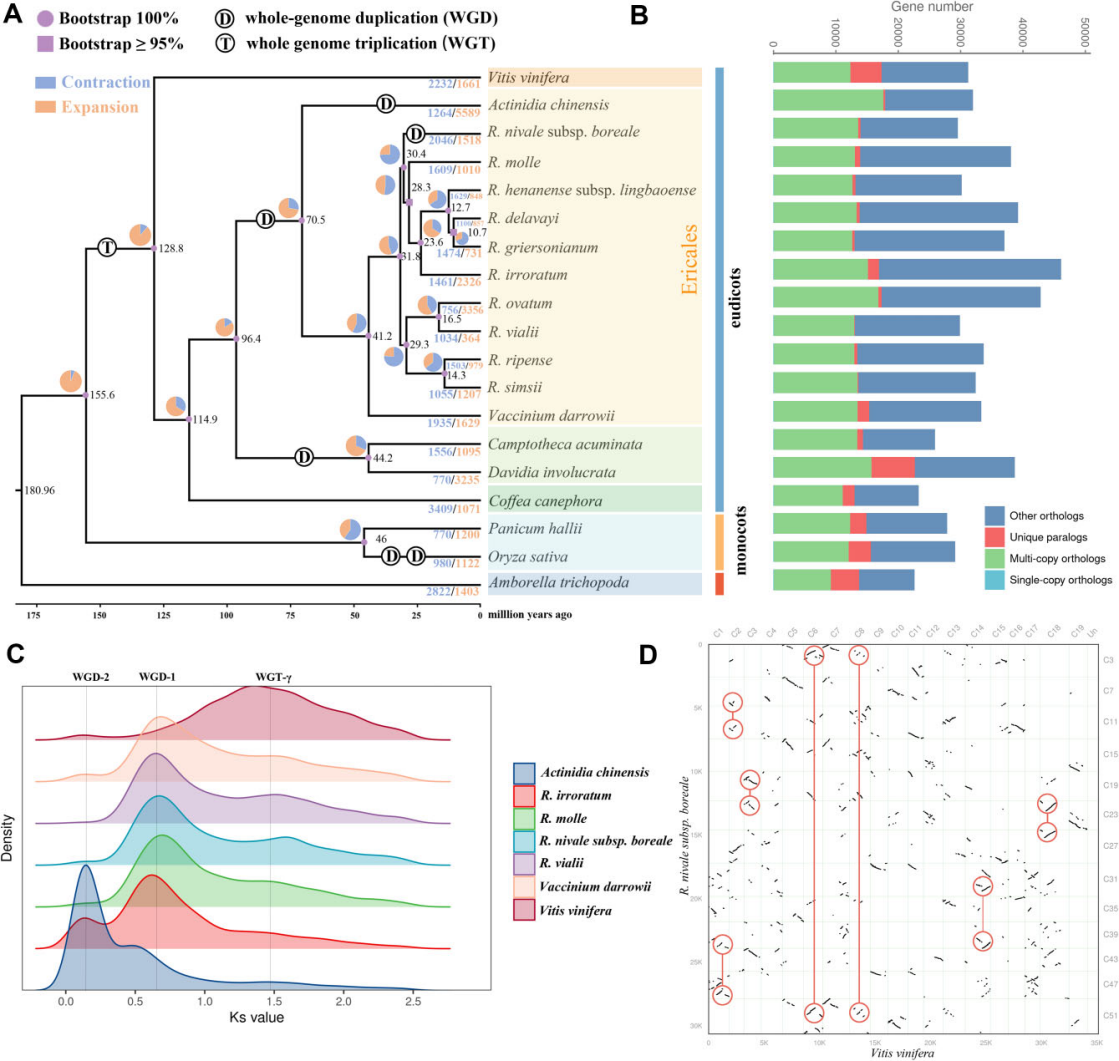
Comparative genomic analysis. (A) ML phylogenetic tree showing the relationship between *R. nivale* subsp. *boreale* and 18 other species. Estimated divergence times (Mya) are labeled at nodes in black. Bootstrap values are displayed on the nodes in circles (100%) and squares (≥95%). Expansion (orange) and contraction (blue) of gene families are shown on the branch, contraction and expansion of ancestors are represented by a pie chart, and extant species are indicated by numbers. WGD and WGT events are marked with D and T, respectively. (B) A number of other orthologs, unique paralogs, multicopy orthologs, and single-copy orthologs in 19 species. (C) *Ks* of paralog frequency distribution chart of 7 species, namely, 6 Ericales (*Actinidia, Vaccinium* and subg. *Hymenanthes*, subg. *Furthermore*, subg. *Rhododendron*, subg. *Tsutsusi*, 1 species each) and 1 *V. vinifera*; polyploidization events are represented by dotted lines. (D) Homologous gene dot plots between *R. nivale* subsp. *boreale* and *V. vinifera*. The red box exemplifies the orthologous ratio of 1:2 between *V. vinifera* and *R. nivale* subsp. *boreale*.

In total, 209 single-copy orthologous genes were used to reconstruct phylogenetic relationships using IQ-TREE. All nodes were supported by high bootstrap values (>95%). The results supported that *Rhododendron* is a monophyletic group, and the 10 species of *Rhododendron* were divided into 4 clades representing 4 subgenera (*Tsutsusi, Rhododendron, Pentanthera*, and *Hymenanthes*) (Fig. 3A). The time tree inferred from the MCMCtree suggested that the ancestor of *Rhododendron* separated from the common ancestor of *Rhododendron* and *Vaccinium darrowii* approximately 41.2 million years ago (Mya). The split between *R. nivale* subsp. *boreale* and the sister groups (*Rhododendron mole, Rhododendron henenense, Rhododendron delavayi, Rhododendron griersonianum*, and *Rhododendron irroratum*) was 30.4 Mya, while the divergence time of *Rhododendron molle* was 28.3 Mya (Fig. 3A).

The synonymous substitution rate (*Ks*) of orthologs and paralogs of 7 species (4 *Rhododendron* species, 1 *Vaccinium*, 1 *Actinidia* species, and 1 *Vitis*) was calculated to determine the WGD events that occurred in *R. nivale* subsp. *boreale*. Polyploidy analysis indicated that *Rhododendron* and *V. darrowii* experienced 2 rounds of ancient polyploidy events, whereas *Actinidia chinensis* experienced 3. Similar peaks were observed for all 4 *Rhododendron* species and *V. darrowii*. The farthest peak also revealed an ancient γ whole-genome triplication (WGT-γ) event common to *Rhododendron* and other core eudicots, which was inferred to have occurred 122–164 Mya from a previous study [31]. In addition, the peak at *Ks* of the paralogs approximately 0.65 Mya suggests another polyploidization event in *Rhododendron, V. darrowii*, and *A. chinensis* estimated to have occurred at approximately 78 Mya (Fig. 3C, Supplementary Fig. S9). In the dot plot comparing *Vitis vinifera* and *R. nivale* subsp. *boreale* (Fig. 3D, Supplementary Figs. S7, S8), nearly every grape chromosome exhibited 2 highly compatible chromosomal regions in *R. nivale* subsp. *boreale* (orthologous ratio 1:2) (Fig. 3D).

### Analysis of positive selection

Using multiple models, we expected to provide more accurate detection of the selection signals of *R. nivale* subsp. *boreale*. First, adaptive branch-site random effects likelihood (aBSREL) was used to test for positive selection in the high-altitude branch of each gene by examining positively selected genes (PSGs). Subsequently, we employed clade model C (CmC) to further evaluate foreground and background selection differences compared with the null model M2a_rel to exclude genes not affected by selection pressure. Twenty potential PSGs were identified by both models (Supplementary Table S18). Positive selection sites were detected using the mixed effects model of evolution (MEME) and the Contrast-FEL, with the number of positive selection sites per gene ranging from 0 to 19 (Supplementary Table S19). Four genes exhibited no significant positive selection sites. Finally, 16 genes met all model criteria and were identified as PSGs (Supplementary Table S19). In addition, 19 genes were identified as PSGs using KaKs_Calculator and were functionally annotated (Supplementary Table S20). These PSGs are associated with various biological processes, including meiosis recombination (*TOP3α*), nucleotide excision repair (*UVR8, RAD23B*), leaf surface wax metabolism (*LTG30, LTP1*), auxin transporter (*ABCB19*), signal transduction and regulation (*M3K1, CAGC1*), and biological clock regulation (*ESD4*). Additionally, we explored the selection pressure within 13 homologous groups where similar pressures were observed (Supplementary Fig. S6).

### Gene duplication and family evolution

**Figure 4:**
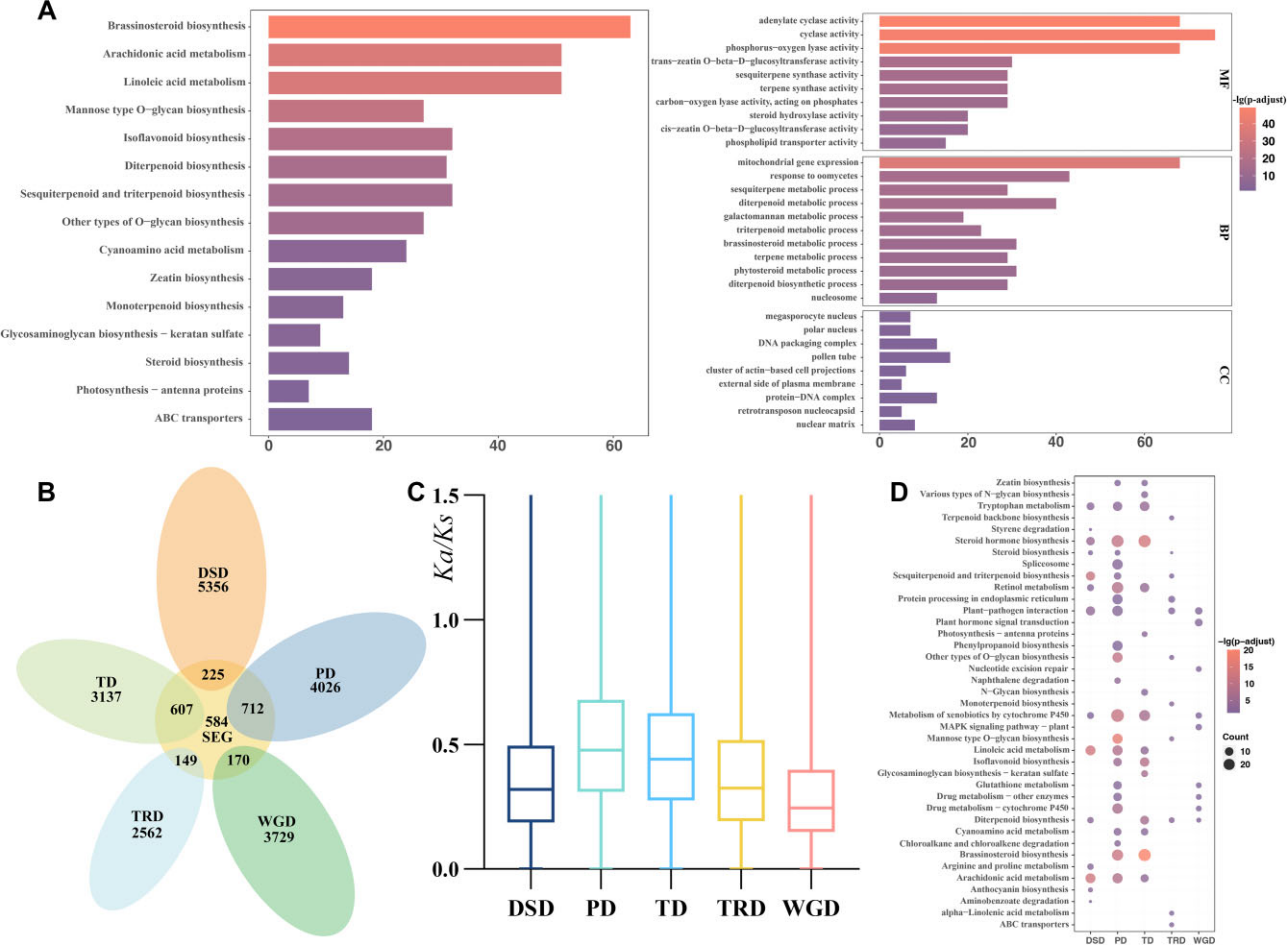
KEGG and GO enrichment and gene duplication analysis of *R. nivale* subsp. *boreale*. (A) KEGG (left) and GO (right) enrichment of genes in significantly expanded gene families. (B) Venn diagram showing the number of shared and specific gene duplications between the significantly expanded genes (SEGs) and 5 categories of duplications (DSD, dispersed duplications; PD, proximal duplications; TD, tandem duplications; TRD, transposed duplications; WGD, whole-genome duplications). (C) *Ka/Ks* ratios of the 5 types of duplications. (D) KEGG pathway enrichment analysis of the 5 duplication types.

Based on the ultrametric tree, the gene family evolution of 19 species was compared with that of the most recent common ancestor (MRCA). Overall, 3,356 orthogroups expanded in *R. nivale* subsp. *boreale*, while only 756 orthogroups contracted. Among these, 375 and 79 orthogroups expanded and contracted significantly, respectively. GO and KEGG enrichment analyses suggested that the significantly expanded orthogroups were primarily enriched in pathways such as brassinosteroid (BR) biosynthesis, terpenoid biosynthesis, and isoflavonoid biosynthesis (Fig. 4A).

To explore the connection between gene duplication and gene family expansion, 20,673 duplicated genes were identified and classified into five categories: 3,899 WGDs (18.86%), 2,711 transposed duplications (TRDs; 13.11%), 3,744 tandem duplications (TDs; 18.11%), 4,738 proximal duplications (PDs; 22.92%), and 5,581 dispersed duplication (DSDs; 27.00%) (Fig. 4B). Among these, PDs and TDs contributed the most to the expansion of gene families. Moreover, ω (*Ka/Ks*) ratios of all duplication categories were calculated, revealing that PDs and TDs demonstrated superior ω scores compared to other types, while the lowest ω score was for WGDs (Fig. 4C). KEGG functional enrichment analysis indicated that the functions of genes shared by the significantly expanded orthogroups and 5 different duplication types were differentiated. WGD genes were enriched in plant hormone signal transduction and nucleotide excision repair, TRDs were implicated in plant–pathogen interactions and *O*-glycan biosynthesis, gene family expansions related to arachidonic acid metabolism and linoleic acid metabolism were mainly contributed by DSDs, and duplications of TDs and PDs were associated with BR biosynthesis, cytochrome P450, and isoflavonoid biosynthesis (Fig. 4D; Supplementary Tables S21–S30).

### Expression of alleles

**Figure 5:**
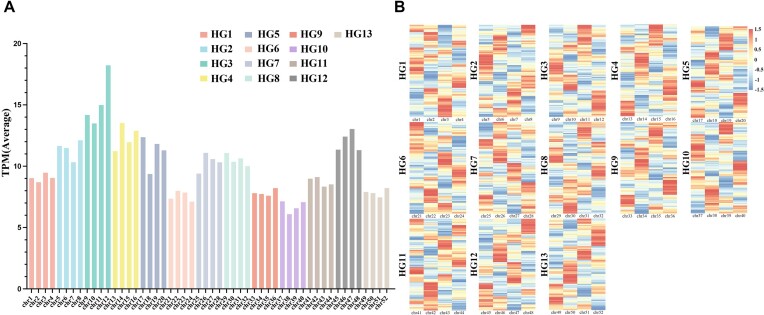
Single-matched allelic expression analysis. (A) The total amount of single-match allelic expression of 52 pseudochromosomes; the colors represent 13 homologous groups (HGs). (B) Heatmap clustering analysis of single-match alleles in screening the position of DELs in *R. nivale* subsp. *boreale*. Each row represents a set of differentially expressed alleles, and each column represents a chromosome. The heatmap shows a homologous group.

Transcriptome data from the roots, stems, leaves, and buds of *R. nivale* subsp. *boreale* were used to explore allelic expression patterns. Overall, 77,892 genes, representing 60.94% of all the genes, were expressed in at least 1 tissue. In single-matching 4 alleles (1:1:1:1), 12,642 of the 14,550 alleles were expressed in at least 1 allele. The expression levels of chromosomes within each homologous group were similar, with homologous group 3 showing a higher transcript expression than the other groups (Fig. 5A). Homologous group 10 had the lowest expression level. We selected genes with a transcripts per kilobase per million mapped reads (TPM) value ≥1 to compare the differences in expression levels between haplotypes, and 6,388 single-match gene groups were identified. Finally, 3,844 of the 6,388 (60.17%) single-match gene groups were identified as differentially expressed loci (DELs), with DEL ratios ranging from 56.14% to 63.93% per pseudochromosome. The DELs were randomly distributed across the genome (Fig. 5B).

### Evolution of the APETALA2/ethylene responsive factor (AP2/ERF)

**Figure 6:**
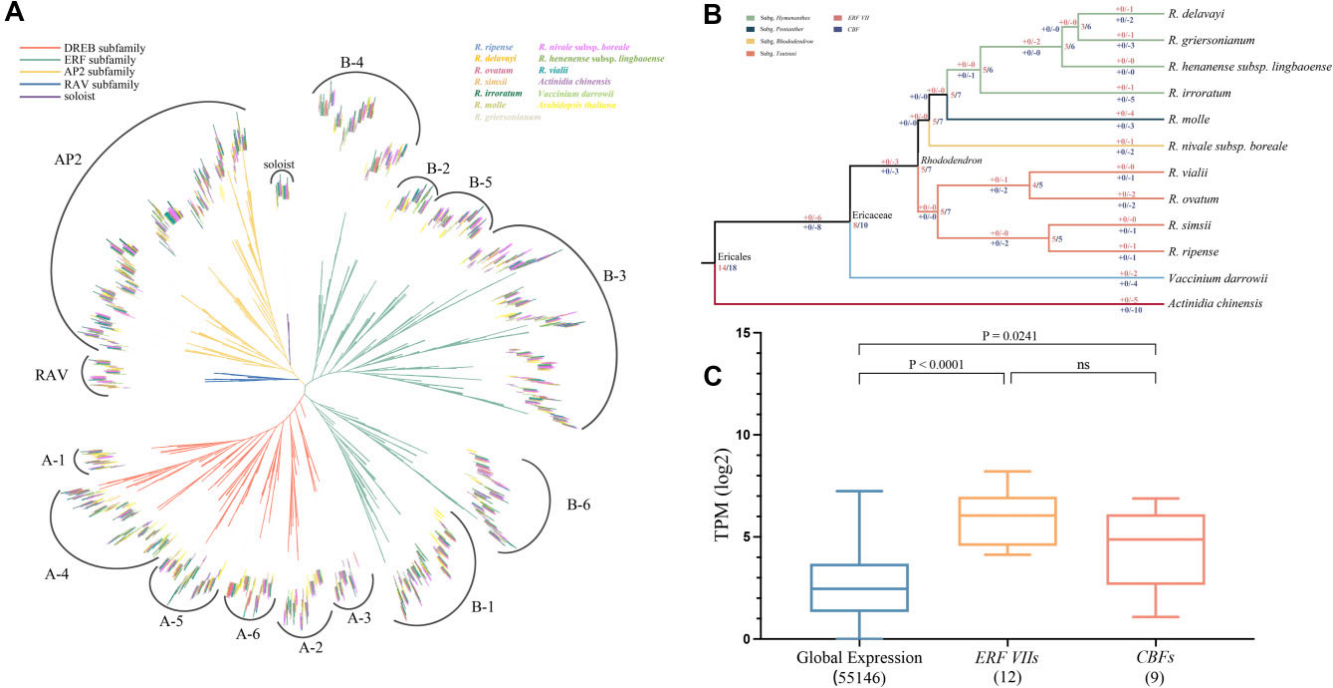
Identification and evolution of key family and genes for adaptation to low mountaintop temperature and hypoxia. (A) Rootless ML phylogenetic tree based on ultra-fast 1,000 bootstrap samplings showed diversified AP2/ERF superfamily in 13 species, including 10 *Rhododendron* species and *A. thaliana*, kiwifruit, and *V. darrowii*. The color of the clades indicates 5 subfamilies of the AP2/ERF superfamily. The labels are differently colored according to species. (B) Schematic diagram of the gain and loss of key genes in 12 species of Ericales; numbers in pink and blue depict *ERF VII* and *CBF* gene family turnover. The numbers in the rectangles and circles represent the number of genes in ancestral and existing species. The + and − signs represent the gain and loss of genes, respectively. (C) Expression levels of *ERF VIIs* and *CBFs*. The numbers in parentheses indicate the number of genes that are expressed. *P* represents the adjusted *P* value.

Plant APETALA2/ethylene responsive factors (AP2/ERFs) and cytochrome P450s (CYPs), which likely play important roles in the adaptation of plants to high altitudes, participate in a multitude of biochemical pathways and fulfill various functions in the realms of growth and protection, including responses to UV irradiation, dehydration, and pathogens [32, 33]. Therefore, AP2/ERF and CYP families were explored. We identified the AP2/ERF family in 10 *Rhododendron* and 2 related species (*A. chinensis* and *V. darrowii*) using the HMMer method. In total, 2,397 genes were identified as belonging to the AP2/ERF family in 12 species after artificially confirming the presence of the AP2 domain (Supplementary Table S31). For convenience, haplotypes were extracted from the haplotype-resolved assemblies of *R. nivale* subsp. *boreale* and *R. vialii* to compare the gene counts within the family. Among *Rhododendron* species, *R. ovatum* exhibited the highest gene count (163), while *R. irroratum* contained only 88 genes. The majority of species in our study had gene counts of approximately 140. These genes were randomly distributed across 13 pseudochromosomes (Supplementary Figs. S10–S21). To further understand the phylogenetic mechanisms of the AP2/ERF family in *Rhododendron*, proteins of *Arabidopsis thaliana* and other 12 species were used to construct the phylogenetic tree. Consistent with previous research [33], 13 categories, including the AP2, ERF (B-1 to B-6), DREB (A-1 to A-6), RAV, and soloist subfamilies, were identified (Fig. 6A). Compared with *A. thaliana*, the categorization of B-3 was expanded in *Rhododendron*.

The alpine environment is variable, with significant temperature differences between day and night, strong ultraviolet radiation, and low oxygen partial pressure. Group VII ethylene response factor transcription factors (*ERF VII*s) are associated with altitude adaptation [34]. Hence, based on the homology of *ERF VIIs* in *A. thaliana*, we identified *ERF VIIs* in *Rhododendron*. Ten species of *Rhododendron* contained 1 (*R. molle*) to 5 (*R. simsii*) *ERF VIIs* (Supplementary Table S32). The phylogenetic tree topology showed that *ERF VIIs* were divided into 4 groups. *ERF VIIs* of *A. thaliana* genes were found in groups I, III, and IV (Supplementary Fig. S22). Group II consisted solely of Ericales genes, and 1 clade contained the alpine species of *Rhododendron*. Based on motif analysis, similar gene structures within the groups showed phylogenetic reliability (Supplementary Fig. S23). Interestingly, we estimated the turnover of *ERF VII* subfamilies using the maximum likelihood (ML) method and found a continuous decrease in the number of genes (Fig. 6B). However, among the 13 *ERF VII* genes, 12 were expressed in the roots, stems, leaves, and buds of *R. nivale* subsp. *boreale* and exhibited significantly higher expression levels than the global gene expression (Fig. 6C).

C-repeat binding factors/dehydration-responsive element binding protein 1 (*CBFs/DREB1s*) play a crucial role as transcription factors that regulate gene expression during cold acclimation. Consistent with previous studies, *CBFs* were categorized as A-1 of the dehydration-responsive element binding (DREB) subfamily, which is separated from A-4 of DREB [35]. We used the BLASTP program and 2 conserved sequences, PKRxAGRxKFxETRHPV and DSAWR, surrounding the AP2/ERF domain to accurately identify *CBF* genes. Sequence comparison with *A. thaliana* revealed that *CBFs* have a highly conserved domain. A total of 49 *CBFs* were identified from 10 *Rhododendron* species, of which *R. nivale* subsp. *boreale* contained 16 *CBFs* (12 *CBF* genes with 4 alleles and 2 *CBF* genes with 2 alleles) (Supplementary Table S32). Phylogenetic analysis indicated that *CBFs* of *Rhododendrons* could be divided into 3 groups (Supplementary Fig. S24). Similar conserved motifs were observed in each group, indicating the reliability of the relationship of *CBFs* (Supplementary Fig. S25). In addition, family turnover based on BadiRate was revalidated, and *CBFs* were continuously lost without gain, resulting in the contraction of *CBF* genes (Fig. 6B). Approximately only half (9/16) of the *CBFs* were expressed (Fig. 6C).

### Evolution of the CYP family

Using the *R. nivale* subsp. *boreale* genome we assembled, along with the genomes of 12 other closely related species, we investigated the evolutionary pattern of the CYP family. The number of CYP family members in each species ranged from 221 to 447, as determined using local BLASTP, hmmsearch, and manual checks (Supplementary Table S33). Among them, *R. irroratum*, which contains 447 CYPs, had the highest gene count. The identified CYP proteins varied in length, ranging from 303 to 621 amino acids. In addition, CYPs were unevenly located on different pseudochromosomes (Supplementary Figs. S10–S21).

**Figure 7:**
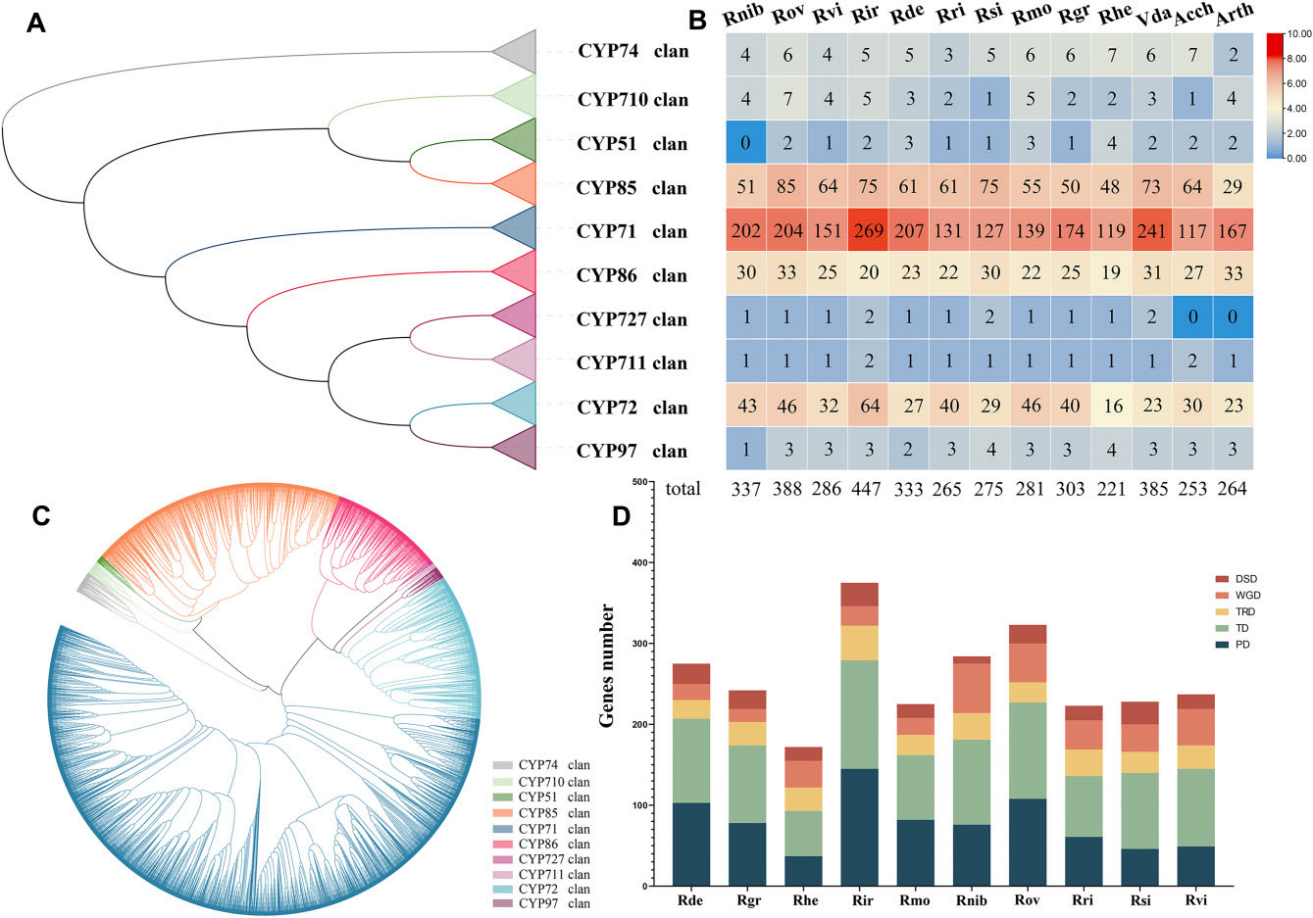
Characteristics of Ericales cytochrome P450 (CYP). (A) ML phylogenetic tree showing the relationship between 10 CYP clans (higher-order groupings of CYP families). (B) Heatmap showing the number of clan members for each species. (C) Phylogenetic tree of the CYP members of 13 species based on GTR (generalized time-reversible). Different clans are represented by different colors. (D) Number of CYP genes produced by duplication events in 10 species of *Rhododendron*.

To determine the phylogenetic relationship between CYPs, we constructed an unrooted ML phylogenetic tree using protein alignments that primarily contained conserved domains and compared them with CYP superfamily members from *A. thaliana*. Following the classification system proposed [36], all CYPs were categorized into 2 distinct types: A-type, which comprises the CYP71 clan, and non-A-type, which comprises the CYP51, CYP72, CYP74, CYP85, CYP86, CYP97, CYP710, CYP711, and CYP727 clans. Our analysis confirmed monophyly within each clan, with the CYP71 clan being the most gene-rich, representing over half of all identified CYPs. In contrast, the CYP711 and CYP727 clans were the smallest (Fig. 7A–C). In comparison to *A. thaliana* and *A. chinensis*, we noted a species-specific increase in the CYP family across different *Rhododendron* species, particularly within the CYP71, CYP85, and CYP72 clans. These expansions are likely to play pivotal roles in species-specific adaptations. The CYP71 clan was mainly involved in the biosynthesis of alkaloids, sesquiterpenoids, cyclic terpenoids, and flavonoids, whereas the CYP85 clan is implicated in the modification of cyclic terpenes and sterols in the BR, abscisic acid (ABA), and gibberellin (GA) pathways. CYP72 is involved in isoprenoid hormone catabolism. Unexpectedly, the CYP family of *R. henanense* subsp*. lingbaoense* was contracted, particularly the CYP72 clan (Fig. 7B). The patterns of duplicated gene pair identification showed that PDs and TDs remarkably contributed to the CYP family variation. In addition, WGD events accounted for a large proportion of duplications in *R. nivale* subsp. *boreale* and *R. ovatum* (Fig. 7D).

## Discussion

Climate change is expected to significantly impact mountaintop ecosystems. Understanding the evolutionary patterns and survival strategies of mountaintop species is imperative to protect them [37]. Polyploidy is beneficial for the survival of species in harsh environments [21]. Therefore, it is essential to understand the evolution and adaptation of extremely high-altitude environmental species from the perspective of polyploidy. In this study, we provide a chromosome-scale and haplotype-resolved autotetraploid genome of *R. nivale* subsp. *boreale* using the DNB-seq, PacBio CCS, and Hi-C sequencing platforms. The *R. nivale* subsp. *boreale* genome contains 52 pseudochromosomes divided into 13 homologous groups. Our assembly was estimated to be of high quality using 4 methods (Merqury, BUSCO, CRAQ, and LAI). In addition, we used various methods to determine autotetraploid identity. As the first autotetraploid genome of an alpine woody plant, the genome of *R. nivale* subsp. *boreale* has laid an important foundation for understanding the adaptation and evolution of woody plants in harsh environments at high altitudes. Consistent with previous systematics of *Rhododendron* studies, subg. *Rhododendron*, to which *R. nivale* subsp. *boreale* belongs, is a sister of subg. *Pentanthera* and subg. *Hymennanthes* [4]. Furthermore, our data suggest that the ancient WGD event in *R. nivale* subsp. *boreale* occurred approximately 78 Mya, which is probably shared with Ericaceae [38]. Overall, *R. nivale* subsp. *boreale* has recently experienced an additional WGD event, in addition to the WGT-γ event shared by the core eudicots and the WGD event shared by the Ericales. Recent polyploidy events, like those observed in other polyploids, are likely important factors for highly conserved pseudochromosomes without rearrangements [31]. The selection analysis revealed similar selection pressures in homologous groups (Supplementary Fig. S6). The different subgenomes of allopolyploids were under significantly different selection pressures, and it has been speculated that different haplotypes of autopolyploids probably also faced different selection pressures [39]. However, the distribution of *Ka*/*Ks* values did not differ between our homologous groups (Supplementary Fig. S6). Based on gene family evolution and positive selection analysis, we postulate the potential high-altitude adaptation strategies for *R. nivale* subsp. *boreale*.

Mountaintop ecosystems are exposed to high levels of UV radiation, low partial pressures of oxygen, and volatile temperatures and humidity [38]. Understanding the mechanisms of the adaptation of plants to high altitudes has long interested botanists. Altitude is positively correlated with UV radiation, with UV radiation rates increasing by 5.1–15% every 1,000 m increase in altitude [40]. Alpine plants employ various mechanisms to mitigate the effects of UV radiation, including cell wall surface modifications and the creation of a leaf cuticle consisting of cutin and cuticular waxes [41]. This cuticle serves as a protective shield against water loss and excessive UV radiation by forming a physical barrier between the plant surface and the environment [12]. Second, to enhance their tolerance to UV radiation and protect themselves from UV damage, plants accumulate flavonoids that absorb UV radiation from strong light [42]. Several genes involved in cuticle and UV tolerance, such as *CER1, FARs*, and *MYB27*, are positively selected in high-altitude plants [12]. We identified a similar situation in *R. nivale* subsp. *boreale* living in a high-altitude mountaintop environment. *LTP1* and *LTPG30* have been identified as PSGs, of which *LTP1* is associated with the biosynthesis and secretion of cuticular wax [43]. In wild-type bilberry (*Vaccinium myrtillus*), which is closely related to *Rhododendron*, expression of the *LTP* gene specific to the skin suggests its involvement in transporting wax compounds into the cuticle [44]. *LTPG30* performs similar functions [45]. Therefore, surface modification of the cell wall is likely to be the initial line of defense against UV damage in *R. nivale* subsp. *boreale*.

Flavonoids are widely recognized as important chemical compounds that protect plants from UV radiation [46]. We observed a significant expansion of the flavonoid gene family, which suggests that the absorption of UV radiation by flavonoid synthesis may be one of the key ways to reduce UV damage in *R. nivale* subsp. *boreale*. This finding was consistent with the conclusions of previous studies [12]. However, the creation of a wax barrier and biosynthesis of flavonoids that absorb UV radiation are insufficient to fully shield plant cells from the intense UV radiation found in mountainous environments. UV radiation not blocked by such barriers reaches deep into cells, damaging biological macromolecules such as DNA, thereby affecting the growth and development of various cells [47]. In this study, *UVR8* and *RAD23B*, which contribute to DNA repair, were positively selected. Specifically, *UVR8* enhances UV-B perception by interacting with the photomorphogenic repressor *COP1*, while *RAD23B* primarily collaborates with *RAD4* to facilitate nucleotide excision repair [48, 49]. These interactions are likely to augment the UV tolerance in *R. nivale* subsp. *boreale*.

In alpine environments, plants have evolved myriad morphological and physiological adaptations to contend with the rigor of high-altitude conditions [50]. *R. nivale* subsp. *boreale* native to mountaintops typically reaches heights of less than 30 cm, with leaves that seldom exceed 5 mm in both length and width; exhibits delayed flowering; and produces seeds that are nearly indiscernible. These characteristics are thought to be a response of *Rhododendron* to low temperatures at high altitudes, poor nutrition, and extremely short growth cycles [51]. Selection analysis revealed PSGs related to auxins, morphogenesis, and the biological clock. For instance, some PSGs identified are associated with auxin transport (*ABCB19*), seed size (*DA1*), and the biological clock (*ESD4*) [52–54]. Concurrently, the gene family for organ development, tissue development, and auxin polar transport expanded significantly. These genes and gene families, which are involved in growth and development, likely shape the special morphology of high-altitude plants such as *R. nivale* subsp. *boreale* and regulate the different stages of their developmental cycle in response to environmental changes, thus better adapting to the extreme spatial and temporal heterogeneity of mountaintop ecosystems [12, 55].

Low temperature (average annual temperature below 0°C), low partial pressure of oxygen (for every 1,000-m increase in altitude, air pressure drops by about 11%), and rapid weather changes (annual average diurnal temperature exceeding 20°C) are the main factors limiting alpine plant survival [56–58]. Under harsh alpine conditions, plants adapt by modifying their morphology, producing specific metabolites, and changing the distribution patterns of biomass [55]. In *R. nivale* subsp. *boreale*, we observed a significant expansion in the gene family associated with BRs, which are pivotal for sustaining plant physiological functions and significantly contribute to enhancing cold tolerance, drought resistance, and antioxidative capabilities [59, 60]. Hence, the expansion of the BR gene family probably enhanced the ability of *R. nivale* subsp. *boreale* to adapt to dramatically changing environments. Moreover, we speculated that the expansion of CYPs, which are associated with stress response, could potentially facilitate the successful adaptation of *R. nivale* subsp. *boreale* at high altitudes. To further understand the adaptability of *R. nivale* subsp. *boreale*, we assessed the dynamics of an important family of transcription factors (AP2/ERF), which is an important group of transcription factors responsive to abiotic stress [61], including key members that adapt to alpine conditions such as *ERF VIIs* and *CBFs/DREB1s. ERF VIIs* and *CBFs* are important transcription factors that respond to low-oxygen partial pressures and temperatures [34, 62]. Unexpectedly, the genes of *Rhododendron* for *ERF VIIs* and *CBFs* continued to be lost lost in low-oxygen, cold alpine environments. *R. nivale* subsp. *boreale* distributed over the alt. 4,000 m is no exception. The high expression levels of *ERF VIIs* in *R. nivale* subsp. *boreale* suggest a potential adaptation to low-oxygen environments, which warrants further experimental investigation. In response to temperature changes, *Rhododendron* species rely on several pathways to enhance their low-temperature resistance, including *CBF*-mediated cold tolerance and an integrated regulatory network of ABA, the MAPK cascade, and Ca^2+^ signaling [63, 64]. Our positive selection analysis results suggested that *M3K1* and *CNGC1*, which are associated with the MAPK cascade and Ca^2+^ signal transduction, might play important roles in low-temperature adaptation.

Polyploidy leads to rapid changes in gene expression and epigenetics, giving the polyploid a significant selective advantage over its diploid progenitors and serving as a crucial mechanism for plants to swiftly adjust to severe environmental stress [6, 18]. Moreover, differential splicing, which is a crucial mechanism in the eukaryotic stress response, changes rapidly after polyploidy and is associated with abiotic stress [65]. Certainly, the influence of genome doubling on phenotypes or life history traits can directly affect the likelihood of survival under challenging circumstances [18]. These include more viable seeds, more rapid growth, and stronger photosynthesis [19, 66]. These characteristics provide plants with great advantages under adverse environmental conditions.

The decline in the fitness of autopolyploids, especially young autopolyploids, is usually attributed to multivalent chromosome pairing during meiosis and mutations in crossover (CO) frequency and distribution [23, 67]. Consequently, autopolyploids experience significant disruptions in their developmental programs, resulting in a considerable reduction in seed production and a high incidence of aneuploid offspring [68]. Addressing these issues requires precise adaptive control of meiosis, such as reduced formation of multichromosome associations and reduced axis lengths [69]. The RECQ4a/4b (BLM)–TOP3α–RMI1 (BTR) complex plays a pivotal role in limiting CO outcomes and maintaining chromosome integrity [70, 71]. Mutations of *RECQ4*, one of its members, significantly affect the stability of polyploid meiosis [72]. Notably, *TOP3α*, an important gene related to the BTR complex, exhibits positive selection in *R. nivale* subsp. *boreale* and may serve as a key factor in promoting accurate chromosomal segregation during meiosis in autopolyploids.

Our results revealed that *R. nivale* subsp. *boreale* distributed on the mountaintop is an autotetraploid, which might be mediated by the harsh environment at high altitudes. Paleopolyploid events are shared among the other 10 *Rhododendron* species of diploids. Our conjecture regarding the alpine adaptation mechanisms of *R. nivale* subsp. *boreale* aligns with those of previous studies: cell wall modification, flavonoid biosynthesis, DNA repair, inhibition of chlorophyll synthesis, and auxin and BR biosynthesis and transduction are probably the main high-altitude adaptation pathways [12]. Polyploidization likely plays an important role in mountaintop survival because increased gene dosage can lead to enhanced stress tolerance, greater genetic diversity, and the potential for novel traits that enhance adaptability [18]. Notably, *TOP3α* is speculated to be an important gene during meiosis in autotetraploids, essential for the generation of normal gametes and implicated in the attenuation of CO events during meiosis. However, these hypotheses require verification through biological experiments. Moreover, the mechanism of the formation of natural polyploids remains to be fully elucidated, and the alpine environment, where polyploidy is concentrated, offers an ideal setting for investigation.

Overall, we assembled the first genome of subg. *Rhododendron*, a rare high-altitude woody autotetraploid genome that provides an important resource for the domestication of high-altitude ornamentals and our understanding of polyploid origin and evolution in mountaintop ecosystems.

## Materials and Methods

### Plant materials and sequencing

*R. nivale* subsp. *boreale* (NCBI:txid1701214) plant materials were collected from Baima Mountain, Dêqên County, Yunnan Province, China (99°4′13′′E, 28°20′24′′N, alt. 4,287.5 m). The plant materials were immersed in liquid nitrogen immediately after collection and preserved at −80°C. High-quality DNA isolated from young leaves was used to create the libraries. Long-read libraries were constructed and sequenced using the PacBio Sequel II sequencing platform. To construct Hi-C libraries, genomic DNA was cross-linked with formaldehyde and digested using the MboI restriction enzyme into 300- to 500-bp fragments, which were sequenced on the BGI DNB-seq sequencing platform. For short reads, DNA libraries were constructed and sequenced on the BGI DNB-seq sequencing platform. Three biological replicates of roots, stems, leaves, and buds of *R. nivale* subsp. *boreale* were sampled. The cDNA libraries were constructed and sequenced on a BGI DNB-seq sequencing platform.

### Genome survey

Flow cytometry and *k-*mer analysis were used to evaluate the genome of *R. nivale* subsp. *boreale*. The following procedures were used for flow cytometry: preparation of nuclear suspension, DNA-specific staining, and testing. We selected *R. griersonianum* as an internal control. Graphical analysis was performed using ModFit LT 5.0 [73] with a coefficient of variation (CV) controlled to within 5. For *k-*mer analysis, DNB-seq short-read clean data were used to count *k-*mer frequency with *k-*mer set to 21 using jellyfish v2.3.0 [74]. Genome size was estimated based on the 21 *k-*mer distribution. Ploidy was estimated using SmudgePlot v0.2.5 [29].

### Genome assembly and scaffolding

The PacBio circular consensus sequencing (CCS) long-read data were assembled using Hifiasm v0.18.9 with Hi-C integration [75], Canu v1.9 [76], and HiCanu v2.2 [77]. We used the parameters of the genome of *Saccharum spontaneum* [16]. The integrity and continuity of the assembly were assessed separately, and the highest quality assembly was used for the scaffolding. The ALLHiC pipeline was used to improve assembly at the chromosomal level based on 5 steps: pruning, partitioning, rescue, optimization, and construction [78]. Manual checks were conducted on potential misassemblies and corrected using Juicebox v1.11.08 [79]. Finally, the assembled genome was evaluated using BUSCO v5.4.6 [80], Merqury v1.3 [81], and Clipping information for Revealing Assembly Quality (CRAQ) v1.0.9 [82] using default parameters. Short reads mapped to the assembled genome using BWA v0.7.17-r1188 [83] and SAMtools v1.17 [84] were counted as properly paired.

### Genome annotation

*De novo* prediction and homology alignment were used to identify whole-genome repeats. The LTRs were initially identified using LTRharvest (RRID:SCR_018970) [85] and LTR_Finder (RRID:SCR_015247) [86]. LTR_retriever v2.9.4 [87] was used to accurately identify LTR retrotransposons (LTR-RTs), generate a nonredundant LTR-RT library, and generate the LAI. A homology search was conducted to predict repeat elements using RepeatMasker v4.1.4 [88]. tRNAs were annotated using tRNAscan-SE (RRID:SCR_008637) v2.0.9 [89], and rRNAs were identified using RNAmmer (RRID:SCR_017075) v1.2 [90]. Other noncoding RNAs, including miRNAs and snRNAs, were annotated by comparison using Infernal v1.1.4, with the Rfam database [91, 92].

We combined *ab initio*, homolog, and transcriptome-based strategies to predict the expression of high-quality protein-coding genes. In our transcriptome-based strategies, we used HISAT2 (RRID:SCR_015530) v2.2.1 [93] to align clean reads of the transcriptome with the genome. Trinity (RRID:SCR_013048) v2.14.0 [94] and StringTie (RRID:SCR_016323) v2.2.1 [95] were used to assemble transcripts. BRAKER3 [96] and PASA (RRID:SCR_014656) v2.5.2 [97] were used to predict gene structure based on the assembled transcripts and to generate *ab initio* gene predictor training sets. For *ab initio*, SNAP (RRID:SCR_007936) [98], GlimmerHMM (RRID:SCR_002654) v3.0.1, and GeneID v1.4 [99] were used to annotate gene structures based on the training sets. For homology-based prediction, protein sequences from a total of 8 species, namely, *A. thaliana, V. vinifera, Glycine max, Nicotiana attenuata, Oryza sativa, R. ovatum, R. griersonianum*, and *R. mole*, were aligned with the genome of *R. nivale* subsp. *boreale* using GeMoMa (RRID:SCR_017646) v1.9 [100]. All gene structures annotated using the above approaches were integrated using the EVidenceModeler (EVM) [101]. Functional annotation of genes was performed using EggNOG (RRID:SCR_002456) v5.0 [102], and protein sequences were aligned to the UniProt database using BLAST v2.6.0 [103].

### Identification of polyploid type

GenomeScope 2.0 [29] was used to count the proportion of nucleotide heterozygosity forms based on the 21 *k-*mer count distributions. AAAB < AABB indicates allotetraploidy, whereas AAAB > AABB indicates autotetraploidy. JCVI utility libraries [104] were used to analyze collinear relationships between haplotypes. To identify the relationship between different haplotypes and related species, 11 transcriptome datasets from 7 related species from previous studies with 4 haplotypes and transcripts of *R. nivale* subsp. *boreale* were used to reconstruct the phylogenetic tree. StringTie v2.2.1 [95] was used to assemble transcripts. An ML tree was reconstructed using IQ-TREE v2.2.2.2 [105] with 1,000 ultra-fast bootstrap replicates after single-copy orthologs were identified by OrthoFinder v2.5.4 [106].

### Comparative genomics analysis

The genomes of *A. chinensis, Amborella trichopoda, Camptotheca acuminata, Davidia involucrata, Oryza sativa, Panicum hallii, R. delavayi, R. griersonianum, R. henanense* subsp. *lingbaoense, R. irroratum, R. molle, R. ovatum, R. ripense, R. simsii, R. vialii, V. darrowii*, and *V. vinifera* were used for comparative genomics analysis with our assembly of *R. nivale* subsp. *boreale*. Single-copy orthologs were identified based on protein sequences using OrthoFinder (RRID:SCR_017118) v2.5.4 [106]. The protein sequences in each single-copy orthogroup were aligned using MUSCLE (RRID:SCR_011812) v5.1 [107] and filtered using trimAI v1.4 [108] and used to construct a phylogenetic tree using IQ-TREE v2. 2.2.2 [105] with 1,000 ultra-fast bootstrap replicates. The MCMCtree program in PAML (RRID:SCR_014932) v4.10 [109] was used to estimate the divergence times. Calibration times were obtained from the TimeTree database [110] and previous studies [4, 111]. A total of 4 calibration points were used to calibrate age: angiosperms, 168–194 Mya; monocots and eudicots, 142.1–163.5 Mya; *Rhododendron* crown, 54.5 Mya; and *P. hallii–O. sativa*, 41.4–51.9 Mya. Based on the ultrametric tree, the expansion and contraction of gene families were estimated using CAFÉ 5 [112]. Functional enrichment analysis of GO and KEGG was performed using the R package clusterProfiler v4.8.3 [113]. Synteny between different species was identified and visualized using the MCscan pipeline in JCVI [104] and MCScanX [114] with default parameters. The *Ks* values of the ortholog and paralog pairs were calculated using KaKs_Calculator v2.0 [115] after alignment with ParaAT v2.0 [116]. WGD times were estimated as *T* = *Ks*/2*r* (*T* is the WGD time and *r* is the rate of divergence). The value of *r* was obtained from a previous study [117].

### Selective analysis

Based on 1,122 single-copy conserved orthologs from Ericales (10 *Rhododendrons, V. darrowii*, and *A. chinensis*), we performed the positive selection analysis acting on the *R. nivale* subsp. *boreale* clade by running separate aBSREL, Clade Model, MEME, and Contrast-FEL. A gene was considered a PSG when it met all model criteria. aBSREL was implemented in HyPhy v2.5.48 [118] with exploratory analysis, representing an improved version of traditional “branch-site” models. The aBSREL test models both site-level and branch-level nonsynonymous-to-synonymous mutation ratio ω heterogeneity but does not test for selection at specific sites. To obtain more accurate PSGs, we used CmC to check the consistency of the model in PAML [109]. This model tested the differential selection pressure between the foreground branches and background for each gene. CmC was then compared with the null model M2a_rel using likelihood ratio tests [119].

To obtain information on specific sites during episodic selection, we applied MEME and Contrast-FEL. MEME tests [120] for sites that were subjected to episodic positive or diversifying selection performed for each gene. The MEME employs a mixed-effects maximum likelihood approach to test the hypothesis that individual sites are subject to episodic, positive, or diversifying selection. For each site, MEME infers 2 ω rate classes and the corresponding weights representing the probability that the site evolves under each corresponding ω rate class at a given branch. Contrast-FEL [121] was used to estimate the difference in ω at each site between different branch sets in codon alignments. The false discovery rate was used to correct for multiple comparisons.

To further understand the selection pressure characteristics of the high- and low-altitude genomes, we used KaKs_Calculator [115] to detect selected genes between *R. nivale* subsp. *boreale* (high altitude) and *R. ovatum* (low altitude). All genes with a *P* value <0.05 and ω (*Ka*/*Ks*) >1 were identified as candidate PSGs.

### Gene expression analysis

Clean reads of the transcriptome were mapped to the genome using STAR and gene expression levels were estimated using STAR v2.7.10b [122]. Accurate quantification (transcripts per kilobase per million mapped reads) of genes was performed using RSEM v1.3.3 [123]. We selected the expression levels of single-match alleles to explore the differences in expression between alleles. The 4 alleles were compared pairwise to identify the differentially expressed alleles. Pairs of alleles exhibiting less than a 2-fold difference in expression were classified as neutral, whereas all other pairs were categorized as nonneutral, that is, DEL. [124]. We used the Kruskal–Wallis test to assess differences in median values among multiple independent samples. The level of significance was set at *P* <  0.05.

### Identification of duplicate gene modes

Different modes of duplicated gene pairs were identified using the DupGen Finder pipeline [125]. The duplicated gene pairs were divided into 5 categories: WGDs, TDs, PDs, TRDs, and DSDs.

### Identification and analysis of key gene families

The AP2/ERF and cytochrome P450 (CYP) gene families were identified using HMMER (RRID:SCR_005305) v3.3.2 (HMMER.org). The structural domain files corresponding to AP2/ERF (PF00847) and CYP (PF00067) were obtained from the Pfam database [126]. A domain file is used as the first template to search for a family. The filtered domain sequences were used as species-specific templates in the second scan. The Pfam and CDD databases [127] were used to verify conserved domains. Conserved sequences containing the main domains were aligned using MAFFT (RRID:SCR_011811) v7.520 [128] and used to construct a phylogenetic tree of the gene family using FastTree v2.1.11 [129] with the GTR + CAT model. Phylogenetic analysis of *CBFs* and *ERF VIIs* was performed using IQ-TREE v2.2.2.2 with 1,000 replicates [105]. Gene motifs were predicted using MEME software v5.5.1 and visualized using TBtools v2.003 [130]. BadiRate v1.35 [131] was used to estimate family turnover rates based on likelihood-based methods.

## Supplementary Material

giae052_GIGA-D-23-00395_Original_Submission

giae052_GIGA-D-23-00395_Revision_1

giae052_GIGA-D-23-00395_Revision_2

giae052_Response_to_Reviewer_Comments_Original_Submission

giae052_Response_to_Reviewer_Comments_Revision_1

giae052_Reviewer_1_Report_Original_Submission Ben Mansfeld, PhD -- 2/21/2024 Reviewed

giae052_Reviewer_1_Report_Revision_1 Ben Mansfeld, PhD -- 5/15/2024 Reviewed

giae052_Reviewer_2_Report_Original_Submission Lav Yadav -- 3/1/2024 Reviewed

giae052_Reviewer_3_Report_Original_Submission Alan E. Yocca -- 3/5/2024 Reviewed

giae052_Reviewer_3_Report_Revision_1 Alan E. Yocca -- 6/6/2024 Reviewed

giae052_Supplemental_Files

## Data Availability

The raw sequencing data of this study have been deposited in the Sequence Read Archive at NCBI under Bioproject number PRJNA1040959. The genome assembly and annotation data are available on Figshare [132] and in the Genome Warehouse at the National Genomics Data Center under accession number GWHETKQ00000000.1. Supporting data are also available via the *GigaScience* database, GigaDB [133].

## Additional Files

**Supplementary Fig. S1**. Estimate of the *Rhododendron nivale* subsp. *boreale* genome via flow cytometry. (A) Flow cytometry of *R. griersonianum*. (B) Flow cytometry of *R. nivale* subp. *boreale* and *R. griersonianum*.

**Supplementary Fig. S2**. The *k-*mer distribution of the *R. nivale* subsp. *boreale* genome based on DNB-seq data.

**Supplementary Fig. S3**. The frequency of the 4 types of repetitive sequences in the genome of *R. nivale* subsp. *boreale*.

**Supplementary Fig. S4**. Dot analysis between 4 haplotypes of *R. nivale* subsp. *boreale* genome. (A) Dot analysis and syntenic depths between haplotype 1 (n1) and haplotype 2 (n2). (B) Dot analysis and syntenic depths between haplotype 2 (n2) and haplotype 3 (n3). (C) Dot analysis and syntenic depths between haplotype 1 (n3) and haplotype 2 (n4).

**Supplementary Fig. S5**. Flower plot indicating the orthologous gene families of 19 species.

**Supplementary Fig. S6**. *K*a/*K*s values of single-match alleles in 52 pseudochromosomes of *R. nivale* subsp. *boreale*. Homologous groups are distinguished by different colors.

**Supplementary Fig. S7**. Collinearity analysis between *R. nivale* subsp. *boreale* and *Vitis vinifera*. Green lines show the genes that have been doubled.

**Supplementary Fig. S8**. The syntenic depth analysis between *R. nivale* subsp. *boreale* and *Vitis vinifera*.

**Supplementary Fig. S9**. *K*s of ortholog frequency distribution chart between *R. nivale* subsp. *boreale* and 6 other species.

**Supplementary Fig. S10**. Chromosomal distribution map of the AP2/ERF and CYP genes in *Ac. chinensis*. The chromosome ID is indicated at the top of each chromosome. The blue lines indicate positions of the AP2/ERF gene, red lines indicate positions of the CYP gene, purple lines indicate positions of the *ERF VII* gene, and green lines indicate positions of the *CBF* gene.

**Supplementary Fig. S11**. Chromosomal distribution map of the AP2/ERF and CYP genes in *R. delavayi*. The chromosome ID is indicated at the top of each chromosome. The blue lines indicate positions of the AP2/ERF gene, red lines indicate positions of the CYP gene, purple lines indicate positions of the *ERF VII* gene, and green lines indicate positions of the *CBF* gene.

**Supplementary Fig. S12**. Chromosomal distribution map of the AP2/ERF and CYP genes in *R. griersonianum*. The chromosome ID is indicated at the top of each chromosome. The blue lines indicate positions of the AP2/ERF gene, red lines indicate positions of the CYP gene, purple lines indicate positions of the *ERF VII* gene, and green lines indicate positions of the *CBF* gene.

**Supplementary Fig. S13**. Chromosomal distribution map of the AP2/ERF and CYP genes in *R. henanense* subsp. *lingbaoense*. The chromosome ID is indicated at the top of each chromosome. The blue lines indicate positions of the AP2/ERF gene, red lines indicate positions of the CYP gene, purple lines indicate positions of the *ERF VII* gene, and green lines indicate positions of the *CBF* gene.

**Supplementary Fig. S14**. Chromosomal distribution map of the AP2/ERF and CYP genes in *R. irroratum*. The chromosome ID is indicated at the top of each chromosome. The blue lines indicate positions of the AP2/ERF gene, red lines indicate positions of the CYP gene, purple lines indicate positions of the *ERF VII* gene, and green lines indicate positions of the *CBF* gene.

**Supplementary Fig. S15**. Chromosomal distribution map of the AP2/ERF and CYP genes in *R. molle*. The chromosome ID is indicated at the top of each chromosome. The blue lines indicate positions of the AP2/ERF gene, red lines indicate positions of the CYP gene, purple lines indicate positions of the *ERF VII* gene, and green lines indicate positions of the *CBF* gene.

**Supplementary Fig. S16**. Chromosomal distribution map of the AP2/ERF and CYP genes in *R. nivale* subsp. *boreale*. The chromosome ID is indicated at the top of each chromosome. The blue lines indicate positions of the AP2/ERF gene, red lines indicate positions of the CYP gene, purple lines indicate positions of the *ERF VII* gene, and green lines indicate positions of the *CBF* gene.

**Supplementary Fig. S17**. Chromosomal distribution map of the AP2/ERF and CYP genes in *R. ovatum*. The chromosome ID is indicated at the top of each chromosome. The blue lines indicate positions of the AP2/ERF gene, red lines indicate positions of the CYP gene, purple lines indicate positions of the *ERF VII* gene, and green lines indicate positions of the *CBF* gene.

**Supplementary Fig. S18**. Chromosomal distribution map of the AP2/ERF and CYP genes in *R. ripense*. The chromosome ID is indicated at the top of each chromosome. The blue lines indicate positions of the AP2/ERF gene, red lines indicate positions of the CYP gene, purple lines indicate positions of the *ERF VII* gene, and green lines indicate positions of the *CBF* gene.

**Supplementary Fig. S19**. Chromosomal distribution map of the AP2/ERF and CYP genes in *R. simsii*. The chromosome ID is indicated at the top of each chromosome. The blue lines indicate positions of the AP2/ERF gene, red lines indicate positions of the CYP gene, purple lines indicate positions of the *ERF VII* gene, and green lines indicate positions of the *CBF* gene.

**Supplementary Fig. S20**. Chromosomal distribution map of the AP2/ERF and CYP genes in *R. vialii*. The chromosome ID is indicated at the top of each chromosome. The blue lines indicate positions of the AP2/ERF gene, red lines indicate positions of the CYP gene, purple lines indicate positions of the *ERF VII* gene, and green lines indicate positions of the *CBF* gene.

**Supplementary Fig. S21**. Chromosomal distribution map of the AP2/ERF and CYP genes in *V. darrowii*. The chromosome ID is indicated at the top of each chromosome. The blue lines indicate positions of the AP2/ERF gene, red lines indicate positions of the CYP gene, purple lines indicate positions of the *ERF VII* gene, and green lines indicate positions of the *CBF* gene.

**Supplementary Fig. S22**. Phylogenetic analysis of the ERF VII proteins in the 13 species. Groups I to IV are distinguished by 4 colors.

**Supplementary Fig. S23**. Conserved motifs analysis of ERF VII. (A) The phylogenetic tree of ERF VII proteins. (B) Conserved motifs of ERF VII. Different motifs are distinguished by color.

**Supplementary Fig. S24**. Phylogenetic analysis of the CBF proteins in the 13 species. Groups I to III are distinguished by 4 colors.

**Supplementary Fig. S25**. Conserved motifs analysis of ERF VII. (A) The phylogenetic tree of ERF VII proteins. (B) Conserved motifs of ERF VII. Different motifs are distinguished by color.

**Supplementary Table S1**. Statistics of PacBio sequencing data.

**Supplementary Table S2**. Statistics of DNB-seq sequencing data.

**Supplementary Table S3**. Genome survey of *R. nivale* subsp. *boreale*.

**Supplementary Table S4**. Statistics of the *R. nivale* subsp. *boreale* genome assembly.

**Supplementary Table S5**. The mapping rate of short reads and HifF reads to the *R. nivale* subsp. *boreale* genome.

**Supplementary Table S6**. Chromosome length distribution of *R. nivale* subsp. *boreale*.

**Supplementary Table S7**. Validation of *R. nivale* subsp. *boreale* genome assembly quality using Merqury.

**Supplementary Table S8**. Validation of genome assembly quality using BUSCO.

**Supplementary Table S9**. Quality of *R. nivale* subsp. *boreale* assembly assessment by CRAQ.

**Supplementary Table S10**. Prediction of *R. nivale* subsp. *boreale* repetitive sequences.

**Supplementary Table S11**. Statistics of *R. nivale* subsp. *boreale* gene structure prediction.

**Supplementary Table S12**. Statistics of gene function annotation.

**Supplementary Table S13**. Annotation of *R. nivale* subsp. *boreale* noncoding RNA genes.

**Supplementary Table S14**. Summary statistics of downloaded data.

**Supplementary Table S15**. Gene family of comparative genomics clustering result statistics.

**Supplementary Table S16**. KEGG pathway enrichment of the unique genes in *R. nivale* subsp. *boreale*.

**Supplementary Table S17**. GO enrichment of the unique genes in *R. nivale* subsp. *boreale*.

**Supplementary Table S18**. Selected analysis based on the clade model.

**Supplementary Table S19**. Genes, function, and site of positive selection.

**Supplementary Table S20**. Genes and function of positive selection based on kaks_calculator.

**Supplementary Table S21**. KEGG pathway enrichment of the genes shared by significant expanded orthogroups and dispersed duplication (DSD).

**Supplementary Table S22**. GO enrichment of the genes shared by significant expanded orthogroups and dispersed duplication (DSD).

**Supplementary Table S23**. KEGG pathway enrichment of the genes shared by significant expanded orthogroups and proximal duplication (PD).

**Supplementary Table S24**. GO enrichment of the genes shared by significant expanded orthogroups and proximal duplication (PD).

**Supplementary Table S25**. KEGG pathway enrichment of the genes shared by significant expanded orthogroups and tandem duplication (TD).

**Supplementary Table S26**. GO enrichment of the genes shared by significant expanded orthogroups and tandem duplication (TD).

**Supplementary Table S27**. KEGG pathway enrichment of the genes shared by significant expanded orthogroups and transposed duplication (TRD).

**Supplementary Table S28**. GO enrichment of the genes shared by significant expanded orthogroups and transposed duplication (TRD).

**Supplementary Table S29**. KEGG pathway enrichment of the genes shared by significant expanded orthogroups and whole-genome duplication (WGD).

**Supplementary Table S30**. GO enrichment of the genes shared by significant expanded orthogroups and whole-genome duplication (WGD).

**Supplementary Table S31**. Statistics of AP2/ERF superfamily of 12 species.

**Supplementary Table S32**. The number of genes in the AP2 superfamily, CBF, and ERF VII.

**Supplementary Table S33**. Statistics of the cytochrome P450 superfamily of 12 species.

## Abbreviations

ABA: abscisic acid; aBSREL: adaptive branch-site random effects likelihood; bp: base pair; BR: brassinosteroid; BUSCO: Benchmarking Universal Single-Copy Orthologs; CCS: circular consensus sequencing; CDS: coding sequences; CmC: clade model C; CO: crossover; CV: coefficient of variation; DEL: differentially expressed loci; DREB: dehydration-responsive element binding; DSD: dispersed duplication; GA: gibberellin; Gb: gigabases; GO: Gene Ontology; HG: homologous group; KEGG: Kyoto Encyclopedia of Genes and Genomes; LAI: LTR assembly index; LTR: long terminal repeat; LTR-RT: long terminal repeat retrotransposon; Mb: megabases; MEME: mixed effects model of evolution; miRNA: microRNA; ML: maximum likelihood; MRCA: most recent common ancestor; Mya: million years ago; NGS: next-generation sequencing; PD: proximal duplication; PSG: positively selected gene; QV: quality value; rRNA: ribosomal RNA; SEG: significantly expanded gene; snRNA: small nuclear RNA; TD: tandem duplication; TRD: transposed duplication; tRNA: transfer RNA; WGD: whole-genome duplication; WGS: whole-genome sequencing; WGT: whole-genome triplication.
