# Supplementary material for: The first high-altitude autotetraploid haplotype-resolved genome assembled (Rhododendron nivale subsp. boreale) provides new insights into mountaintop adaptation
Source: Gigascience. 2024 Aug 7;13:giae052. doi: 10.1093/gigascience/giae052 (PMC11304948; doi:10.1093/gigascience/giae052)
Supplement: giae052_GIGA-D-23-00395_Original_Submission [file giae052_giga-d-23-00395_original_submission.pdf]

GigaScience

The first high-altitude autotetraploid haplotype-resolved genome assembled (Rhododendron nivale subsp. boreale) provides new insights into mountaintop adaptation

--Manuscript Draft--

**Manuscript Number:**	GIGA-D-23-00395
**Full Title:**	The first high-altitude autotetraploid haplotype-resolved genome assembled (Rhododendron nivale subsp. boreale) provides new insights into mountaintop adaptation
**Article Type:**	Research
**Funding Information:**	Science and Technology Development Fund of Guidance from the Central Government to Locals (202207AB110016) — Prof. Shi-Kang Shen<br>Graduate Scientific Research Fund Project of Yunnan University (KC-22221373) — Mr. Zhen-Yu Lyu<br>Major Program for Basic Research Project of Yunnan Province (202101BC070002) — Prof. Shi-Kang Shen<br>National Natural Science Foundation of China (31870529) — Prof. Shi-Kang Shen

Abstract:

Background
Rhododendron nivale subsp. boreale Philipson et M. N. Philipson, is an alpine woody with ornamental qualities and serves as the predominant species in mountainous scrub habitats found at an altitude of ~4200 m. Despite ecological significance, the lack of genomic resources has hindered a comprehensive understanding of its evolutionary and adaptive characteristics in high-altitude environments.
Findings
Here, we sequenced and assembled the genome of R. nivale subsp. boreale, which is an assembly of the first subgenus Rhododendron and the first high-altitude woody flowering autotetraploid. The assembly included 52 pseudochromosomes (scaffold N50=42.93 Mb; BUSCO=98.8%; QV=45.51; S-AQI=98.69), which belonged to 4 haplotypes, harbor 127,810 predicted protein-coding genes. Comparative genomic analysis revealed that R. nivale subsp. boreale originated as a neopolyploid resulting from R. nivale and experienced two rounds of ancient polyploidy event. Transcriptional expression analysis showed that the expression differences of alleles were common, randomly distributed in the genome. We identified signatures of positive selection involved not only in adaptations to mountaintop ecosystem (response to UV radiation and developmental regulation), but also in strategy of autotetraploid reproduction (meiotic stabilization). Notably, highly expressed ERF VIIs aid survival in hypoxic mountaintop. Meanwhile, the extended families enriched in brassinosteroid biosynthesis, which enhanced adaptability to alpine weather and probably mediated by increased cytochrome P450 genes.
Conclusions
We assembled the first high-altitude autopolyploid genome and achieved the initial chromosome-level assembly within the subgenus Rhododendron. This study provides a valuable data into the exploration of alpine adaptation and the correlation between extreme environments and species polyploidization.

**Corresponding Author:**	Shi-Kang Shen<br>Yunnan University<br>Kunming, CHINA
**Corresponding Author Secondary Information:**	
**Corresponding Author's Institution:**	Yunnan University

Powered by Editorial Manager® and ProduXion Manager® from Aries Systems Corporation

Corresponding Author's Secondary Institution:	
First Author:	Zhen-Yu Lyu
First Author Secondary Information:	
Order of Authors:	Zhen-Yu Lyu
	Xiong-Li Zhou
	Si-Qi Wang
	Gao-Ming Yang
	Wen-Guang Sun
	Jie-Yu Zhang
	Rui Zhang
	Shi-Kang Shen
Order of Authors Secondary Information:	
Additional Information:	
Question	Response
Are you submitting this manuscript to a special series or article collection?	No
**Experimental design and statistics**<br><br>Full details of the experimental design and statistical methods used should be given in the Methods section, as detailed in our Minimum Standards Reporting Checklist. Information essential to interpreting the data presented should be made available in the figure legends.<br><br>Have you included all the information requested in your manuscript?	Yes
**Resources**<br><br>A description of all resources used, including antibodies, cell lines, animals and software tools, with enough information to allow them to be uniquely identified, should be included in the Methods section. Authors are strongly encouraged to cite Research Resource Identifiers (RRIDs) for antibodies, model organisms and tools, where possible.<br><br>Have you included the information	Yes

Powered by Editorial Manager® and ProduXion Manager® from Aries Systems Corporation

requested as detailed in our Minimum Standards Reporting Checklist?	
**Availability of data and materials**<br><br>All datasets and code on which the conclusions of the paper rely must be either included in your submission or deposited in publicly available repositories (where available and ethically appropriate), referencing such data using a unique identifier in the references and in the "Availability of Data and Materials" section of your manuscript.<br><br>Have you have met the above requirement as detailed in our Minimum Standards Reporting Checklist?	Yes

Manuscript Click here to access/download;Manuscript;manuscript.docx

Title: The first high-altitude autotetraploid haplotype-resolved genome assembled (*Rhododendron nivale* subsp. *boreale*) provides new insights into mountaintop adaptation

Authors: Zhen-Yu Lyu[1], Xiong-Li Zhou[1], Si-Qi Wang[1], Gao-Ming Yang[1], Wen-Guang Sun[2], Jie-Yu Zhang[2], Rui Zhang[1], Shi-Kang Shen[1*]

Affiliation:

[1]Ministry of Education Key Laboratory for Transboundary Ecosecurity of Southwest China, Yunnan Key Laboratory of Plant Reproductive Adaptation and Evolutionary Ecology, Institute of Biodiversity, School of Ecology and Environmental Science, Yunnan University, Kunming, 650504, Yunnan, China

[2]School of Life Sciences, Yunnan Normal University, Kunming, 650500, Yunnan, China

Corresponding authors: *Shi-Kang Shen, E-mail: ssk168@ynu.edu.cn; (ORCID: 0000-0002-0611-6763)

Telephone: +86-871-65933510; Fax: +86-871-65933510;

Postal address for corresponding authors: School of Ecology and Environmental Sciences, Yunnan University, No.2 Green lake North road Kunming, Yunnan, 650091, China

Words: 7034 (excluding references)

Figures: 7 color figures.

Abstract

Background

Rhododendron nivale subsp. *boreale* Philipson et M. N. Philipson, is an alpine woody with ornamental qualities and serves as the predominant species in mountainous scrub habitats found at an altitude of ~4200 m. Despite ecological significance, the lack of genomic resources has hindered a comprehensive understanding of its evolutionary and adaptive characteristics in high-altitude environments.

Findings

Here, we sequenced and assembled the genome of *R. nivale* subsp. *boreale*, which is an assembly of the first subgenus *Rhododendron* and the first high-altitude woody flowering autotetraploid. The assembly included 52 pseudochromosomes (scaffold N50=42.93 Mb; BUSCO=98.8%; QV=45.51; S-AQI=98.69), which belonged to 4 haplotypes, harbor 127,810 predicted protein-coding genes. Comparative genomic analysis revealed that *R. nivale* subsp. *boreale* originated as a neopolyploid resulting from *R. nivale* and experienced two rounds of ancient polyploidy event. Transcriptional expression analysis showed that the expression differences of alleles were common, randomly distributed in the genome. We identified signatures of positive selection involved not only in adaptations to mountaintop ecosystem (response to UV radiation and developmental regulation), but also in strategy of autotetraploid reproduction (meiotic stabilization). Notably, highly expressed *ERF VIIs* aid survival in hypoxic mountaintop. Meanwhile, the extended families enriched in brassinosteroid biosynthesis, which enhanced adaptability to alpine weather and probably mediated by increased cytochrome P450 genes.

Conclusions

We assembled the first high-altitude autopolyploid genome and achieved the initial chromosome-level assembly within the subgenus *Rhododendron*. This study provides a valuable data into the exploration of alpine adaptation and the correlation between extreme environments and species polyploidization.

Key words: Autotetraploid, Evolutionary history, Mountaintop adaptation, *Rhododendron*

1. Introduction

Rhododendron L. is not only the core genus of Ericaceae, the largest woody plant genus in the Northern Hemisphere with more than 1,000 species, but is also representative of the highly diverse Sino-Himalayan Flora in East Asia, shaped by the topographic and climatic heterogeneity resulting from the uplift of the Qinghai-Tibet Plateau [1,2]. Furthermore, *Rhododendron* is one of the few woody flowering species that exhibits dominance in plant communities found within the delicate subalpine to alpine transition zone and presents a perfect opportunity to explore the mechanisms behind the evolution and adaptation of alpine woody plants [3,4]. In *Rhododendron, R. nivale* subsp. *boreale* Philipson et M. N. Philipson is one of the few woody flowering plants discovered to be distributed at altitudes above 5000 m and one of the minority polyploid (2n=4x=52) woody plants in the Qinghai-Tibet Plateau [1,5,6]. *R. nivale* subsp. *boreale* belongs to Subg. *Rhododendron* is a small-leaved, much-branched, prostrate or erect shrub distributed from the mountaintop (up to alt. 5400 m) to the mountainside (~3200 m). In addition, the diversity of habitats, including alpine meadows, forest margins and metal mining areas, shows strong adaptability [7]. Meanwhile, *R. nivale* subsp. *boreale* has been developed as an important ornamental plant resource in plateau mountainous areas and is used in traditional Tibetan medicine [8,9]. Therefore, exploring the evolutionary model and adaptation mechanism of *R. nivale* subsp. *boreale* not only promotes the understanding of alpine adaptation evolution of woody plants but also lays a foundation for the commercial use of high-altitude ornamental plant.

The genetic perspective provides us with a better understanding of species evolution and adaptive differentiation [10,11]. However, the scarcity of genomic data makes the development

of research difficult [12]. For example, in a recent high-altitude adaptation study, self-incompatibility, cell wall modification, DNA repair and stress resistance are considered to be associated with high altitude survival, however only 5 alpine plant genomes were used, indicating that the genetic resources of alpine plants are far from sufficient compared to the large number of high-altitude plants [13]. Moreover, polyploidy probably enhanced the plant adaptability to harsh environment [14]. Unfortunately, acquiring polyploid genetic data remains a challenging endeavor, particularly in the case of autopolyploids with highly similar subgenomes. At present, limited by the difficulty of assembling autopolyploid genomes, only a limited number of autopolyploid genomes have been published, such as *Medicago sativa, Saccharum spontaneum, Solanum tuberosum* and *Rheum officinale* [15,16,17,18]. To further understand the evolution and adaptation of alpine flora, more genetic resources, especially polyploids, are essential.

Polyploidy has been theorized to be both a potential evolutionary roadblock and a catalyst for evolutionary breakthroughs and the proliferation of species [19]. On the one hand, following polyploidy, rapid shifts in gene expression and epigenetic modifications can bestow the polyploid with an almost instant competitive edge [20]. Diploid and polyploid natural taxa have notable differences in their ranges, usually if polyploids tend to have more expanded ranges than their diploid ancestors [21]. Therefore, polyploidy tends to be ecologically advantageous and occurs in variable climatic regions, such as the Qinghai-Tibet Plateau alpine and Pan-Arctic regions [22,23]. On the other hand, auto and allopolyploids face a significant obstacle, which is the accurate segregation of chromosomes during meiosis [24,25]. In recent years, we have seen a significant increase in our comprehension of the molecular foundation regarding

polyploid adaptations to meiotic challenges, but compared to allotetraploids, we know far less about the molecular mechanisms underlying the stabilization of autotetraploid meiosis [26]. In the face of advances in molecular technology, more genetic resources will provide new insights into the survival, evolution and adaptation mechanisms of polyploids.

Here, we present a haplotype-resolved tetraploid genome of *R. nivale* subsp. *boreale* from an altitude of 4,287.5 m, which is the first chromosome-level genome assembly of the subgenus *Rhododendron*. Based on this assembly, we identified the polyploid types, deciphered whole genome duplication (WGD) events and determined which genes or gene families play a role in alpine adaptation and the survival of polyploids. This genome not only establishes the groundwork for comprehending the evolution and adaptation of *Rhododendron* but also offers valuable genetic resources to investigate the origin, recombination, and differentiation of polyploid species.

2. Results

2.1 Genome estimation, sequencing and assembly

R. nivale subsp. *boreale* samples from mountaintop were collected and treated with liquid nitrogen and were then sequenced (Fig. 1A). We obtained a total of 33.35 gigabases (Gb) of PacBio CCS long reads with an average length of 15.86 kb and an N50 length of 16.15 kb (Table S1). A genome survey was performed based on DNBseq short reads (95.61 Gb; Table S2), and the result revealed an estimated genome size of 2.48 Gb, which was consistent with that estimated by flow cytometry (Fig. S1, S2; Table S3). Tetraploid was identified from the *k-mers* present in sequencing reads (Fig. 1E). The three initial assembly sizes were 2.48 Gb, 2.39

Gb and 2.40 Gb, which were assembled by Hifiasm, Canu v1.9 and Hicanu, respectively (Table S4). The assembled version of Hifiasm was used for subsequent analysis because it has higher integrity in genes and LTRs. The long reads and NGS reads were mapped to the unitig-level assembly to assess assembly quality. Long-reads and whole-genome sequencing (WGS) reads were mapped 99.87% and 99.59%, respectively, and RNA-seq reads were more than 94% (Table S5). The average GC content was 41.10%. We used the AllHiC algorithm to improve the genome assembly to the chromosome level by 138.98 Gb of Hi-C data. After manual checking, a total of 2.17 Gb of unitigs were anchored to 52 pseudochromosomes (scaffold N50=42.93 Mb), ranging in length from 23.70 to 59.70 megabase (Mb), which contained four haplotypes (13 pseudochromosomes per haplotype) (Fig. 1C; Table S6). The Hi-C heatmap clearly showed the interactions of 13 homologous groups (Fig. 1D). There was high similarity between the pseudochromosomes per homologous group.

This genome was assembled with a high assembly consensus quality value (QV=45.51; Error rate=0.0028%) and a high *k-mer* completeness (97.79%) (Table S7). BUSCO assessment indicated that the completeness of the genome assembly was 98.8% of the embryophyte conserved genes (Table S8). The quality of genome structure at the reference genome level (assembly quality indicators of large structural fragments; S-AQI=98.69) was assessed by CRAQ (Table S9).

2.2 Annotation

A repeat sequence of 1,549,068,457 bp was identified, accounting for 62.63% of the genome assembly (Table S10). The richest category of repeats was LTRs (43.12%), with *Gypsy* and *Copia* accounting for 33.66% and 5.88%, respectively (Fig. S3). In addition, the LTR

assembly index (LAI) was 12.19 based on LTR annotation, which indicated that the assembly met the reference category. By combining ab initio prediction, homology prediction, and transcriptome data prediction, 127,810 protein-coding genes were predicted, with an average gene length of 4,736.07 bp. The total length of coding sequences (CDS) was 148,274,600 bp, and the average number of CDSs per gene was 4.8 (Table S11). The completeness of 98.6% annotated protein-coding genes of *R. nivale* subsp. *boreale* was assessed by BUSCO. Of the protein-coding genes, 96.86% were functionally annotated (Table S12). We annotated 17,049 candidate noncoding RNAs, including 703 microRNAs (miRNAs), 3,672 transfer RNAs (tRNAs), 5,373 small nuclear RNAs (snRNAs) and 7,301 ribosomal RNAs (rRNAs) (Table S13).

2.3 Confirmation of Autotetraploid

Polyploid species are usually discovered in plants. However, the origin of polyploids was different, including both homologous and heterologous origins. Allotetraploids have been extensively studied, such as peanuts, cotton, and wheat [27,28,29]. These allotetraploid species had significant differences in subgenomes and therefore could be divided into different subgenomes. In contrast to allotetraploids, the high similarity of haploids greatly increases the difficulty of assembling autotetraploids. To determine the polyploid type of *R. nivale* subsp. *boreale*, we used *k-mer* analysis, collinearity analysis and phylogenetic analysis for cross-validation. 21 *k-mer* frequency analysis showed that four distinct peaks were displayed (located at 33, 68, 106 and 136) (Fig. S2), which were highly similar to the results of autoploids (*Medicago sativa* and *Saccharum spontaneum*). Moreover, nucleotide heterozygosity was considered to be an important criterion for determining polyploid types. The genome analysis

of *R. nivale* subsp. *boreale* showed that AAAB was estimated to be 2.53%, and AABB was estimated to be 1.28% (Table S3). This was consistent with the expectation that the heterozygous rate of autotetraploid AAAB is greater than that of AABB. Nevertheless, these were not sufficient to identify polypoid type. For example, genomic analysis of *Artemisia argyi* showed that AAAB > AABB but was identified as an allotetraploid [30]. Therefore, to further determine the polypoid type of *R. nivale* subsp. *boreale*, synteny analysis was performed based on syntenic blocks. As we expected, the dot plot and syntenic blocks indicated that a certain synteny was observed among the four haplotypes (Fig. 2A), of which 20,172 gene pairs exerted synteny between haplotype 1 and haplotype 2, 20,249 gene pairs exerted synteny between haplotype 2 and haplotype 3, and 19,883 gene pairs exerted synteny between haplotype 3 and haplotype 4 (Fig. 2B, S4). Meanwhile, we downloaded transcriptome data from 11 samples of 7 closely related species (Table S14) to infer phylogenetic relationships for four haplotypes of *R. nivale* subsp. *boreale*. The results showed that a monophyletic group consisted of 6 species (*R. nitidulum*, *R. hippophaeoides*, *R. thymifolium*, *R. nivale*, *R. nivale* subsp. *boreale* and *R. lapponicum*) of subsect. *Lapponica* with high support. A clade containing all four haplotypes of *R. nivale* subsp. *boreale* and two *R. nivale* was supported by 100% bootstrapping (Fig. 2 C). Therefore, four haploid chromosomes were considered to originate from the same species. Simultaneously, a total of 25,791 orthogroups were identified in the four haplotypes, of which 16,403 orthogroups shared four haplotypes, 5,242 orthogroups shared three haplotypes, 3,631 orthogroups shared two haplotypes and only 515 orthogroups (n1: 134; n2: 139; n3: 135; n4: 107) were unique to each haplotype genome, showing a high genetic similarity between the four haplotypes (Fig. 2D). Overall, the combined results of *k-mer* analysis, collinearity analysis

and phylogenetic analysis indicated that *R. nivale* subsp. *boreale* was an autotetraploid species.

2.4 Comparative analysis and recent polyploidization

The phylogenetic position and divergence times of *R. nivale* subsp. *boreale* were inferred with 18 other species, including 12 species of Ericales (10 *Rhododendron*), 2 species of Cornales, 1 species of Gentianales, 1 species of Vitales, 2 species of monocotyledons and 1 basal angiosperm. Altogether, 666,442 genes were used to infer orthology. A total of 625,661 genes (93.9%) were clustered into 37,844 orthologous gene families, of which 6,547 orthogroups were shared with all species (Fig. 2B; Table S15). Furthermore, 209 single-copy gene families were identified. A total of 146 gene families, including 369 genes, were specific to *R. nivale* subsp. *boreale* (Fig. S5). Furthermore, 369 genes were identified as unique in *R. nivale* subsp. *boreale*. These species-specific genes were enriched in 12 KEGG pathways and 136 GO terms, such as arginine biosynthesis, nitrogen metabolism and flavonoid biosynthesis (Table S16; S17).

Altogether, 209 single-copy orthologous genes were used to reconstruct phylogenetic relationships via IQ-TREE. All nodes were supported by high bootstrap values (>95%). The analysis results supported that *Rhododendron* is a monophyletic group, and 10 species of *Rhododendron* were divided into 4 clades that represent 4 subgenera (*Tsutsusi*, *Rhododendron*, *Pentanthera* and *Hymenanthes*) (Fig. 3A). The time tree inferred from the MCMCtree shows that the ancestor of *Rhododendron* separated from the common ancestor of *Rhododendron* and *Va. darrowii* approximately 41.2 Mya. The split between *R. nivale* subsp. *boreale* and the sister group (*R. mole, R. henenense, R. delavayi, R. griersonianum, R. irroratum*) was 30.4 Mya, while the divergence time of *R. molle* was shown to be 28.3 Mya (Fig. 3A).

The synonymous substitution rate (*Ks*) of orthologs and paralogs of 7 species (4 *Rhododendron* species, 1 *Vaccinium*, 1 *Actinidia* and 1 *Vitis*) was calculated to determine the whole-genome duplication (WGD) events that occurred in *R. nivale* subsp. *boreale*. The polyploidy analysis indicated that *Rhododendron* and *Va. darrowii* experienced two rounds of ancient polyploidy events, while *Ac. chinensis* experienced three. Similar peaks appeared in 4 *Rhododendron* species and *Va. darrowii*. Based on the whole-genome triplication (WGT) event of *Vi. vinifera*, the farthest peak revealed an ancient γ triplication (WGT-γ) event common to *Rhodoendron* and other core eudicots, which was inferred from a previous study to have occurred 122–164 Mya [32]. In addition, the recent peak at *Ks* ~0.65 indicated that another polyploidy of *Rhododendron*, *Va. darrowii* and *Ac. chinensis* occurred ~78 Mya (Fig. 3C, S9). However, the distribution of *Ks* showed that three peaks of *Ac. chinensis* each represented three ancient polyploidy events. In the dot plot between *Vi. vinifera* and *R. nivale* subsp. *boreale* (Fig. 3D, S7–8). We identified nearly every grape chromosome with two highly compatible chromosome regions in *R. nivale* subsp. *boreale* (orthologous ratio 1:2) (Fig. 3D).

2.5 Analysis of positive selection

A total of 44 genes were positively selected and functionally annotated based on Hyphy and kaks_calculator (Table S18; S19). These gene functions were mainly associated with biological regulation, response to stimulus, morphogenesis of plants and homologous recombinatio. For example, *RAD23B* is a UV excision repair protein and is involved in global genome nucleotide excision repair by acting as a component of the *RAD4* complex. *FLS* catalyzed the formation of flavonols from dihydroflavonols. *ASY3* was associated with greater meiotic stability in autotetraploids. *LTP1* was related to leaf surface wax metabolism. *ABCB19*

is an auxin transporter protein-encoding gene primarily involved in the development of roots, stems, leaves, anthers, and stamens. There is also an important cyclic nucleotide-gated ion channel, *CNCG1*, in calcium (Ca^{2+}) signal transduction. *CNGCs* are involved in plant responses to various abiotic and biotic stress conditions. In addition, we also explored the selection pressure within the 13 homologous groups. However, there are similar selection pressures in homologous groups (Fig. S6). According to previous studies, different subgenomes of allopolyploid were under significantly different selection pressures, and it was speculated that different haplotypes of homologous polyploids probably also faced different selection pressures [32]. However, our results do not support this.

2.6 Gene duplication and family evolution

Based on the ultrametric tree, the gene family evolution of 19 species was compared with their most recent common ancestor (MRCA). In all, 3,356 orthogroups were expanded in *R. nivale* subsp. *boreale*, while only 756 orthogroups were contracted. Among them, 375 and 79 orthogroups were significantly expanded and contracted, respectively. Gene Ontology (GO) and Kyoto Encyclopedia of Genes and Genomes (KEGG) enrichment analyses suggested that significantly expanded orthogroups were primarily enriched in pathways such as brassinosteroid biosynthesis, terpenoid biosynthesis and isoflavonoid biosynthesis (Fig. 4A).

To explore the connection between gene duplication and gene family expansion, a total of 20,673 duplicated genes were identified and classified into five categories. These included 3,899 whole-genome duplication (WGD; 18.86%) duplications, 2,711 transposed duplication (TRD; 13.11%) duplications, 3,744 tandem duplication (TD; 18.11%) duplications, 4,738 proximal duplication (PD; 22.92%) duplications and 5,581 dispersed duplication (DSD;

27.00%) duplications (Fig. 4B). Among them, PD and TD contributed the most to the expansion of gene families. Moreover, ω (*Ka/Ks*) ratios of all categories of duplications were calculated, and the results indicated that PD and TD demonstrated superior ω scores compared to other types; however, the lowest ω score was for WGD (Fig. 4C). KEGG functional enrichment analysis showed that the functions of genes shared by significantly expanded orthogroups and five different duplication types were differentiated. Among them, WGD genes were enriched in plant hormone signal transduction and nucleotide excision repair; TRD duplications implicated in plant-pathogen interaction and *O*-glycan biosynthesis; gene family expansions related to arachidonic acid metabolism and linoleic acid metabolism were mainly contributed by DSD; duplications of TD and PD were associated with brassinosteroid biosynthesis, cytochrome P450 and isoflavonoid biosynthesis (Fig 4D; Table S20–S29).

2.7 Expression of alleles

Transcriptome data from root, stem, leaf and bud of *R. nivale* subsp. *boreale* were used to explore allelic expression patterns. Overall, 77,892 genes, which represent 60.94% of all genes, exhibited expression in at least one tissue. In single matching four alleles (1:1:1:1), 12,642 of the 14,550 alleles were expressed in least one allele. The expression levels of chromosomes in each homologous group were similar, and the transcript expression of homologous Group 3 was higher than that of the other groups (Fig. 5A). Homologous Group 10 was identified as having the lowest expression level. We selected genes with TPM greater than 1 to compare the differences in expression levels between haplotypes and a total of 6,388 single-match gene groups were identified. Finally, 3,844 of the 6,388 (60.17%) single-match gene groups were identified as differential expression loci (DELs), with DEL ratios ranging from 56.14% to

63.93% in per pseudochromosome. The DELs were randomly distributed across the genome (Fig. 5B).

2.8 Evolution of the APETALA2/ethylene responsive factor (AP2/ERF)

Plant APETALA2/ethylene responsive factors (AP2/ERFs) and cytochrome P450 enzymes (CYPs) participate in a multitude of biochemical pathways and fulfill various functions in the realms of growth and protection, including responses to UV irradiation, dehydration, and pathogens [33,34]. Therefore, The AP2/ERF gene family and CYP family were studied. We identified the AP2/ERF family of 10 *Rhododendron* and 2 related species (*Actinidia chinensis*, *Vaccinium darrowii*) using the HMMer method. In total, 2,397 genes were identified as belonging to AP2/ERF families in 12 species after artificially determining the presence of the AP2 domain (Table S30). For convenience, a haplotype of two haplotype-resolved assembles (*R. nivale* subsp. *boreale* and *R. vialii*) was used in the family gene number comparison. In *Rhododendron*, the largest number of genes of any species was *R. ovatum* (163); however, *R. irroratum* contained only 88 genes. More species had approximately 140 genes. These genes were randomly distributed across 13 pseudochromosomes. To further understand the phylogenetic mechanism of the AP2/ERF family of *Rhododendron*, proteins of *Ar. thaliana* and other 12 species were used to construct the phylogenetic tree. Consistent with previous research, a total of 13 categorizations were identified, including the AP2 subfamily, ERF (B-1 to B-6) subfamily, DREB (A-1 to A-6) subfamily, RAV subfamily and soloist (Fig. 6A). Compared with *Ar. thaliana*, categorization of B-3 was expanded in *Rhododendron*.

The alpine environment was variable, with a significant temperature difference between day and night, strong ultraviolet radiation, and low oxygen partial pressure as its main

characteristics. However, the AP2/ERF gene family is an important transcription factor family in response to abiotic stress. It contains important transcription factors that adapt to alpine environments, such as Group VII ethylene response factor transcription factors (*ERF VIIs*) and *CBF/BREB1s* (*CBFs*). *ERF VIIs* are *ERF* (ethylene response factor) categorized B-2 members of the ERF/AP2 transcription factor family, which are within the clade of the ERF subfamily. Based on the homology of ERF VIIs in *Ar. thaliana*, we identified ERF VIIs in *Rhododendron*. Ten species of *Rhododendron* contain 1 to 5 *ERF VIIs*, with *R. simsii* having the highest number of genes (5) and the least containing only 1 (*R. molle*) (Table S31). The system tree topology shows that *ERF VIIs* were divided into four groups. *ERF VIIs* of *Ar. thaliana* genes were within Groups I, III, and IV (Fig. S10). Group II, however, contained only Ericales genes, and there was a clade that contained alpine species of *Rhododendron*. Based on motif and gene structure analysis, similar gene structures within the groups showed phylogenetic reliability (Fig. S11). Previous studies have shown that *ERF VIIs* play an extremely important role in adapting to high-altitude environments. Interestingly, in the important gene subfamily associated with high-altitude adaptation, we estimated the turnover of *ERF VII* subfamilies using the maximum likelihood method, and the results showed a continuous decrease in the number of genes in *ERF VIIs* (Fig. 6B). However, among the 13 *ERF VII* genes, 12 were highly expressed in roots, stems, leaves, and buds of *R. nivale* subsp. *boreale*, which was probably due to *R. nivale* subsp. *boreale* living in an environment with a low pressure of oxygen (Fig. 6C).

Similar gene loss to *ERF VIIs* also occurs in the *CBF* family. Multiple research studies indicate that C-repeat binding factors/dehydration-responsive element binding protein 1 s

(*CBFs/DREB1s*) play a crucial role as transcription factors in regulating gene expression during cold acclimation. Consistent with previous studies, *CBFs* were categorized as A-1 of the dehydration-responsive element binding (DREB) subfamily, which was separated from the categorized A-4 of DREB. We used the BLASTP program and two conserved sequences, PKRxAGRxKFxETRHPV and DSAWR, surrounding the AP2/ERF domain to accurately identify *CBF* genes. The sequence comparison with *Ar. thaliana* showed that *CBFs* had a highly conserved domain. A total of 49 *CBFs* were identified from species of 10 *Rhododendron*, of which *R. nivale* subsp. *boreale* contained 16 *CBFs* (12 *CBF* genes with four alleles; 2 *CBF* genes with two alleles) (Table S31). Phylogenetic analysis indicated that *CBFs* of *Rhododendron* were divided into 4 groups (Fig. S12). Similar conserved motifs appeared in each group, representing the reliability of the *Rhododendron CBF* relationship (Fig. S13). In addition, family turnover based on badiratd was revalidated, and *CBFs* were continuously lost without gain, resulting in the contraction of *CBF* genes (Fig. 6B).

2.9 Evolution of the CYP family

Based on the *R. nivale* subsp. *boreale* genome provided by us, together with 12 other closely related species, we explored the evolutionary pattern of the CYP family. The number of haplotype CYP family members for each species ranged from 221 to 447 via local BLASTP, hmmsearch methods and manual checks (Table S32). Among them, the largest number of genes identified was *R. irroratum*, which contains 447 *CYPs*. These variations include protein lengths, which range from 303 to 621 amino acids. In addition, the CYPs were unevenly located on different pseudochromosomes.

To determine the phylogenetic relationship of CYPs, an unrooted ML tree was constructed

from the alignment of proteins containing primarily conserved domains with CYP superfamily members of *Ar. thaliana*. According to a previous study of plant CYP evolution, all CYPs were categorized into 2 distinct types, including A-type and no-A-type CYPs. The A-type contains the CYP71 clan, while the CYP51 clan, CYP72 clan, CYP74 clan, CYP85 clan, CYP86 clan, CYP97 clan, CYP710 clan, CYP711 clan and CYP727 clan belong to the no-A-type. In our results, monophyly was determined in every clan, of which the CYP71 clan contained the most genes, accounting for more than 50% of all CYPs. The CYP711 clan and CYP 727 clan were identified as the smallest clans (Fig. 7A–C). In *Rhododendron*, compared to *Ar. thaliana* and *A. chinensis*, we also noticed a specific increase in the CYP family for different species. For instance, species-specific CYP expansion occurs in the CYP71 clan, CYP85 clan and CYP72. However, the other clan showed no notable variation. Therefore, the species-specific expansion of CYP clans probably plays an important role in different species. CYP71 clan was mainly involved in the biosynthesis of alkaloids, sesquiterpenoids, cyclic terpenoids and flavonoid synthesis; CYP85 clan involved in modification of cyclic terpenes and sterols in the brassinosteroid (BR), abscisic acid (ABA) and gibberellin (GA) pathways; CYP72 clan involved in catabolism of isoprenoid hormones. The expanded clans probably contributed significantly to the expansion of gene families. Unexpectedly, the CYP family of *R. henanense subsp. lingbaoense* was contracted, especially in the CYP72 clan (Fig. 7B). The modes of duplicated gene pair identification showed that PD and TD contributed more to the variation in the CYP family. In addition, WGD events occupied a large proportion of *R. nivale* subsp. *boreale* and *R. ovatum* (Fig. 7D).

3. Discussion

Climate change is likely to have a significant impact on mountaintop ecosystems, and understanding the evolutionary patterns and survival strategies of mountaintop species is imperative to protect them [35]. Meanwhile, polyploidy is considered beneficial for survival in harsh environments [22]. Therefore, it is essential to comprehend the evolution and adaptation of high-altitude extreme environmental species from the perspective of polyploidy. In this study, we provide a chromosome-scale and haplotype-resolved autotetraploid genome of *R. nivale* subsp. *boreale* using a combination of DNBseq, PacBio CCS and Hi-C sequencing platforms. This genome contains 52 pseudochromosomes and is divided into 13 homologous groups. Our assembly is estimated as high quality by four methods (Merqury, BUSCO, CRAQ and LAI). In addition, we used a variety of methods to determine autotetraploid identity. As the first autotetraploid genome of alpine woody plants, the genome of *R. nivale* subsp. *boreale* has laid an important foundation for understanding the adaptation and genome evolution of woody plants to harsh environments at high altitudes. Consistent with previous systematics of *Rhododendron* studies, Subg. *Rhododendron,* to which *R. nivale* subsp. *boreale* is a sister of Subg. *Pentanthera* and Subg. *Hymennanthes* [4]. Furthermore, our data suggest that the recent ancient WGD event in *R. nivale* subsp. *boreale was* estimated at approximately 78 Mya, which is probably shared with Ericaceae [36]. Overall, in addition to the WGT-γ event shared by the core eudicots and the WGD event shared by the Ericaceae, an additional WGD event recently occurred in *R. nivale* subsp. *boreale*. As with other polyploids, recent polyploidy events are probably an important factor in the observation of highly conserved pseudochromosomes without rearrangement [31].

Mountaintop ecosystems are exposed to high levels of UV radiation, low partial pressure

of oxygen and volatile temperature and humidity [35]. How plants adapt to high altitudes has always been a puzzle that botanists have been dedicated to unraveling. Previous studies have shown that UV dose rates increase by 5.1%–15% for every 1,000 m increase in altitude, which is associated with a weakening of the Rayleigh scattering effect at high altitudes [37,38,39]. Alpine plants employ various mechanisms to mitigate the effects of UV radiation, including cell wall surface modifications and the creation of a leaf cuticle consisting of cutin and cuticular waxes [40]. This cuticle serves as a protective shield against water loss and excessive UV radiation by forming a physical barrier between the surface and environment of plants [13]. In addition, plants accumulate flavonoids to absorb UV radiation from strong light, enhance their tolerance to UV radiation, and protect plants from UV harm [41,42]. Moreover, studies have shown that several genes involved in cuticle and flavonoid biosynthesis, including *CER1*, *FARs*, *MYB27* and *MYB48*, were positively selected [13]. We also identified a similar situation in *R. nivale* subsp. *boreale* living at high altitudes. *LTP1* and *LTPG30* are identified as positively selected genes, of which *LTP1* is associated with the biosynthesis and secretion of cuticular wax [43,44]. For example, among wild type bilberry (*Vaccinium myrtillus*) that are closely related to *Rhododendron*, the expression of the *LTP* gene specific to the skin suggests its involvement in transporting wax compounds into the cuticle [45]. In addition, *LTPG30* performs a similar function [46]. Therefore, the surface modification of the cell wall probably plays an important role in the high UV radiation of *R. nivale* subsp. *boreale*.

Flavonoids are widely recognized as important chemical compounds that protect plants from the effects of UV radiation [47]. Significant expansion of the flavonoid gene family and positive selection of its important synthesis-related *FLS* showed that absorption of UV

radiation by flavonoid synthesis is one of the important ways to reduce the UV damage of *R. nivale* subsp. *boreale*.Previous studies have reached similar conclusions [13]. However, only the creation of a wax barrier and the biosynthesis of flavonoids to absorb UV radiation are not sufficient to fully shield plant cells from the intense UV radiation found in mountainous environments. UV radiation missed by other barriers, which reaches deep into cells, causes damage to biological macromolecules such as DNA, thereby affecting the growth and development of various cells [48]. Fortunately, the gene (*RAD23B*) that contributes to DNA repair was identified as being positively selected [49]. *RAD23B* mainly interacts with *RAD4* to exercise the function of nucleotide excision repair, thereby increasing UV tolerance [50].

To adapt to the special environment of high altitude, plants have adopted different morphological and physiological strategies, such as lower height, leaf size and a tight seasonal cycle of growth and senescence [51,52]. We also identified some positive selection genes related to auxin and morphogenesis. For instance, *ABCB19, DA1* and *DGR2* are associated with auxin transport, organ size, and root development, respectively [53,54,55]. Simultaneously, the gene family for organ, tissue development and auxin polar transport was identified as significantly expanded. These genes and gene families involved in growth and development are considered to shape the special morphology of high-altitude plants and regulate the different stages of their development cycle in response to environmental changes, thus better adapting to the extreme spatial and temporal heterogeneity of mountaintop ecosystems [13,56].

Low temperature, low partial pressure of oxygen and large and rapid weather changes are also among the main factors limiting the survival of alpine plants [57]. In the face of harsh

environments, plants adapt to harsh alpine environments by modifying their morphology, producing specific metabolites and changing the distribution pattern of biomass [56,58,59]. The gene family for important plant hormones (Brassinosteroids; BRs) associated with plant development and stress resistance was identified as significantly expanded in *R. nivale* subsp. *boreale*. BRs play an essential role in maintaining the normal physiological functions of plants, including cell division and elongation in roots and stems, reproductive development, plant growth, leaf senescence, seed germination, stomatal development, and morphogenesis of plant light and heat. At anti-low temperatures, anti-drought and antioxidation also produce significant effects [60,61]. Hence, the expansion of the BR gene family probably enhances the ability of *R. nivale* subsp. *boreale* to adapt to dramatically changing environments. Moreover, CYPs is considered an essential biosynthetic component of endogenous chemicals, such as BRs and terpenoids [62,63]. Our results showed an increased number of CYP family genes in *R. nivale* subsp. *boreale*, which may be an important factor in the expansion of the terpenoid and BR gene families. To further understand the adaptability of *R. nivale* subsp. *boreale*, we identified an important family of transcription factors (AP2/ERF) that respond to abiotic stress [64]. In the AP2/ERF family, *ERF VIIs* and *CBFs* have been identified as important transcription factors in response to low oxygen partial pressure and low temperature [59,65]. Unexpectedly, the genes of Rhododendron for *ERF VIIs* and *CBFs* continued to be lost in the low-oxygen, cold alpine environment. Even *R. nivale* subsp. *boreale*, which is distributed over alt. 4000 m, is no exception. The difference is that *ERF VIIs* are highly expressed (12/13 expressed), while *CBFs* are expressed at relatively low levels (10/16 expressed) in *R. nivale* subsp. *boreale*. Hence, in mild hypoxia, the *ERF VII*-mediated inhibition of chlorophyll

biosynthesis diverts limited resources to other biological processes that do not rely on oxygen, thereby enabling *R. nivale* subsp. *boreale* to adapt and thrive in low-oxygen environments [110]. For temperature change, *R. nivale* subsp. *boreale* relies on several different pathways to improve low temperature resistance in addition to *CBFs*-mediated cold tolerance. For example, an integrated regulatory network of ABA, MAPK cascade and Ca^{2+} signaling comodulating and the interaction of *HY5–ELIP3* [66,67,68].

Polyploidy leads to rapid changes in gene expression and epigenetics, giving the polyploid a significant selective advantage over its diploid progenitors and serving as a crucial mechanism for plants to swiftly adjust to severe environmental stress [6,19]. A young polyploid displays novel gene expression patterns, along with a dominant expression level compared to the parental genome [69]. In all single-matched alleles of *R. nivale* subsp. *boreale*, four alleles were expressed in 61%. The presence of multiple alleles indicated that the expression level of *R. nivale* subsp. *boreale* is probably higher than that of the diploid ancestor. The predominance of polyploid expression was also verified in *Ar. thaliana* [69]. Moreover, differential splicing, which serves as a crucial mechanism in the eukaryotic stress response, is observed to change rapidly after polyploidy and is associated with abiotic stress [70,71]. Certainly, the influence of genome doubling on phenotype or life history traits can directly impact their likelihood of survival amidst challenging circumstances [19]. These include more viable seeds, more rapid growth and stronger photosynthesis [20,72]. These characteristics provide plants with great advantages in the face of adversity.

The fitness decline of autopolyploids, especially young autopolyploids, is usually due to the occurrence of multivalent chromosome pairing during meiosis, resulting in the separation

of abnormal chromosomes in the gametes and causing aneuploid and genetically unbalanced offspring [24,73]. Therefore, autopolyploids experience significant disruptions in developmental programs, resulting in a considerable reduction in seed production and a high incidence of aneuploid offspring [74]. To address this challenge, the precise adaptive control of meiosis, such as reduced formation of multichromosome associations, reduced axis length, and a tendency toward more rod-shaped bivalents in metaphase I, is important for stable polyploid meiosis [75]. Hence, *ASY1* and *ASY3*, which are associated with these processes, are part of the autopolyploid meiosis solution [75,76]. *ASY3* is positively selected in *R. nivale* subsp. *boreale*, which suggests that *ASY3* has important implications for the regulation of brief division of autopolyploids and is likely to be universal throughout polyploid angiosperms.

Our results reveal that *R. nivale* subsp. *boreale* distributed on the mountaintop is an autotetraploid, which is probably mediated by the harsh environment at high altitudes. Paleopolyploid events are shared with the other 10 *Rhododendron* species of diploids. Similar to previous study, cell wall modification, flavonoid biosynthesis, DNA repair, inhibition of chlorophyll synthesis, auxin and BR biosynthesis and transduction are the main high-altitude adaptation pathways [13]. In addition, polyploidization plays an important role in mountaintop survival due to preponderant expression [19]. During meiosis in autotetraploids, the production of normal gametes is heavily dependent on *ASY3*, which is associated with meiosis-specific protein synthesis. More importantly, the mystery of the formation of natural polyploidy has not been fully revealed, and the alpine environment where polyploidy is concentrated is the ideal area to reveal it. We assembled the first genome of Subge. *Rhododendron*, and a rare high-altitude woody autotetraploid genome that provides an

important resource for domestication of high-altitude ornamentals and our understanding of polyploid origin and evolution in mountaintop ecosystems.

4. Materials and methods

4.1 Plant materials and sequencing

R. nivale subsp. *boreale* plant materials were collected from Baima Mountain, Dêqên County, Yunnan Province, China (99°4’13”E, 28°20’24”N, alt. 4287.5 m). The plant materials were put into liquid nitrogen immediately after collection and preserved at -80 °C. High-quality DNA isolated from young leaves was utilized to create libraries. The long-read libraries were constructed and sequenced using the PacBio Sequel II sequencing platform. To construct Hi-C libraries, genomic DNA was cross-linked with formaldehyde and digested using the MboI restriction enzyme, resulting in 300–500 bp fragments, which were sequenced on the BGI DNBseq sequencing platform. For short reads, DNA libraries were constructed and sequenced via the BGI DNBseq sequencing platform. Three biological replicates of roots, stems, leaves and buds of *R. nivale* subsp. *boreale* were sampled. cDNA libraries were constructed and sequenced using the BGI DNBseq sequencing platform.

4.2 Genome survey

Flow cytometry and *k-mer* analysis methods were utilized to evaluate the genome of *R. nivale* subsp. *boreale*. For flow cytometry, the following procedures were followed: preparation of nuclear suspension, DNA-specific staining and testing. We selected *R. griersonianum* as an internal control. Graphical analysis was performed using Modifit 5.0 with the coefficient of variation (CV) controlled to within 5. For *k-mer* analysis, DNBseq short reads clean data were

used for counting *k-mer* frequency with *k-mer* set to 21 using jellyfish v2.3.0 [77]. The genome size was estimated based on the 21 *k-mers* distribution. The ploidy was estimated using Smudgeplot v0.2.5 [78].

4.3 Genome assembly and scaffolding

PacBio circular consensus sequencing (CCS) long-read data were assembled using Hifiasm v0.18.9 with Hi-C integration [79], Canu v1.9 [80] and HiCanu v2.2 [81]. We referred to the parameters of the genome of *Saccharum spontaneum* [17]. The integrity and continuity of the assembly were then assessed separately, and the highest quality assembly was used for scaffolding. The ALLHiC pipeline was used to improve assembly to the chromosomal level based on five steps: pruning, partitioning, rescue, optimization and construction [82]. Manual checks were conducted on potential misassemblies, and they were corrected using Juicebox v1.11.08 [83]. Finally, we evaluated the assembled genome using Benchmarking Universal Single-Copy Orthologs (BUSCO) v5.4.6 [84], Merqury v1.3 software [85] and Clipping information for Revealing Assembly Quality (CRAQ) v1.0.9 [86] with default parameters. The short reads mapped to the assembled genome using BWA v0.7.17-r1188 [87] and SAMtools v1.17 [88] were counted as the properly paired rate.

4.4 Genome annotation

De novo prediction and homology alignment were used to identify whole-genome repeats. The identification of long terminal repeats (LTRs) was initially done using LTRharvest [89] and LTR_Finder [90]. LTR_retriever v2.9.4 [91] was used to accurately identify LTR retrotransposons (LTR-RTs), generate a nonredundant LTR-RT library and generate the LTR assembly index (LAI). The homology search predicts repeat elements using RepeatMasker

v4.1.4 [92]. The transfer RNAs (tRNAs) were annotated using tRNAscan-SE v2.0.9 [93], and the ribosomal RNAs (rRNAs) were identified using RNAmmer v1.2 [94]. Other noncoding RNAs, including miRNAs and snRNA, were annotated through comparison using Infernal v1.1.4 with the Rfam database [95,96].

From the beginning, we combined homology-based and transcriptome-based strategies to predict high-quality protein-coding genes. For transcriptome-based strategies, BRAKER3 [97] was performed with transcriptome clean reads, which were obtained by HISAT2 v2.2.1 [98] aligned to the genome. Trinity v2.14.0 [99] and StringTie v2.2.1 [100] were used to assemble the transcripts. PASA v2.5.2 [101] was used to predict the gene structure based on the assembled transcripts and generated Ab initio gene predictor training sets. For Ab initio, SNAP [102], GlimmerHMM v3.0.1 and GeneID v1.4 [103] were used to annotate gene structures based on training sets. For homology-based prediction, a total of 8 species of protein sequences, including *Arabidopsis thaliana, Vitis vinifera, Glycine max, Nicotian attenuata, Oryza sativa, R. ovatum, R. griersonianum* and *R. mole*, were aligned to the genome of *R. nivale* subsp. *boreale* using GeMoMa v1.9 [104]. All gene structures annotated by the above approaches were integrated via EVidenceModeler (EVM) [105]. The functional annotation of genes was performed using EggNOG v5.0 [106], and protein sequences were aligned to the UniProt database using BLAST v2.6.0 [107].

4.5 Identification of polyploid type

Based on the 21 *k-mer* count distribution, we used GenomeScope 2.0 [78] to count the proportion of nucleotide heterozygosity forms. Normally, AAAB < AABB indicates an allotetraploid, while the autotetraploid plot has AAAB > AABB. The JCVI utility libraries

(https://zenodo.org/record/31631) were used to analyze collinear relationships between haplotypes. To identify the relationship between different haplotypes and related species, 11 transcriptome data from 7 related species in previous studies with 4 haplotypes and transcripts of *R. nivale* subsp. *boreale* were used to reconstruct the phylogenetic tree. StringTie v2.2.1 [100] was used to assemble transcripts. A maximum likelihood (ML) tree was reconstructed using IQ-TREE v2.2.2.2 [108], after single copy orthologs were identified by OrthoFinder v2.5.4 [109].

4.6 Comparative genomics analysis

The genomes of *Actinidia chinensis, Amborella trichopoda, Camptotheca acuminata, Davidia involucrata, Oryza sativa, Panicum hallii, R. delavayi, R. griersonianum, R. henanense* subsp. *lingbaoense, R. irroratum, R. molle, R. ovatum, R. ripense, R. simsii, R. vialii, Vaccinium darrowii* and *Vitis vinifera* were used for comparative genomics analysis along with our assembly of *R. nivale* subsp. *boreale*. Single-copy orthologs were identified via OrthoFinder v2.5.4 [109] based on protein sequences. The protein sequences in each single-copy orthogroup were aligned using MUSCLE v5.1 [110] and filtered by trimAI v1.4 [111] and were then used to construct a phylogenetic tree via IQ-TREE v2. 2.2.2 [108] with 1000 ultrafast bootstrap replicates. The MCMCtree program in PAML v4.10 [112] was used to estimate divergence times. Calibration times were obtained from the TimeTree database (http://timetree.org) and previous studies [4,113]. A total of four calibration points were used to calibrate age: angiosperms 168–194 Mya; monocots eudicots 142.1–163.5 Mya; *Rhododendron* crown 54.5 Mya; *Panicum hallii-Oryza sativa* 41.4–51.9 Mya. Based on the ultrametric tree, the expansion and contraction of gene families were estimated using CAFÉ 5 [114]. Functional enrichment

analysis of Gene Ontology (GO) and Kyoto Encyclopedia of Genes and Genomes (KEGG) was performed using the R package clusterProfiler v4.8.3 [115]. Synteny between different species was identified and visualized using JCVI (https://zenodo.org/record/31631) and MCScanX [116] with default parameters. The *Ks* value of ortholog and paralog pairs was calculated using KaKs_Calculator v2.0 [117] after alignment with ParaAT v2.0 [118]. WGD times were estimated following T=*Ks*/2r (T: WGD time; r: rate of divergence). The value of r was obtained from a previous study [119].

4.7 Identification of positively selected genes

Based on single-copy conserved orthologs, HyPhy v2.5.48 [120] was used to detect positively selected genes with adaptive branch-site random effects likelihood (aBSREL). To further understand the selection pressure characteristics of high-altitude and low-altitude genomes, we used KaKs_Calculator [117] to detect selected genes between *R. nivale* subsp. *boreale* (high altitude) and *R. ovatum* (low altitude). All genes with a P value < 0.05 and ω (*Ka*/*Ks*) > 1 were identified as candidate positively selected genes.

4.8 Gene expression analysis

The clean reads of the transcriptome were mapped to the genome using STAR, and the gene expression level was estimated using STAR v2.7.10b [121]. Accurate quantification (transcripts per kilobase per million mapped reads; TPM) of genes was performed by RSEM v1.3.3 [122]. We selected expression levels of single match alleles to explore differences in expression between alleles. Four alleles were pairwise compared to determine which alleles were differentially expressed. Pairs of alleles exhibiting less than a twofold difference in expression were classified as neutral, while all other pairs were categorized as nonneutral [123].

4.9 Identification of duplicate gene modes

The different modes of duplicated gene pairs were identified using the DupGen_finder pipeline [124]. All duplicated gene pairs were divided into five categories: whole-genome duplicates (WGD), tandem duplicates (TD), proximal duplicates (PD), transposed duplicates (TRD) and dispersed duplicates (DSD).

4.10 Identification and analysis of key gene families

The AP2/ERF and cytochrome P450 (CYP) gene families were identified using HMMER v3.3.2 (HMMER.org). Structural domain files corresponding to AP2/ERF (PF00847) and CYP (PF00067) were obtained from the Pfam database website (https://www.ebi.ac.uk/interpro/). The domain file was used as the first template for searching the family. The filtered domain sequences were taken as species-specific templates for a second scan. Furthermore, the Pfam and CDD (https://www.ncbi.nlm.nih.gov/cdd/) databases were used to verify the conserved domains. The conserved sequences containing the main domains were aligned using MAFFT v7.520 [125] and used to construct a phylogenetic tree of the gene family by IQ-TREE v2.2.2.2. Gene motifs were predicted using MEME software v5.5.1 and were visualized via TBtools v2.003 [126]. BadiTate v1.35 [127] was used to estimate family turnover rates based on likelihood-based methods.

Data availability

The raw sequencing data of this study have been deposited in the Sequence Read Archive (SRA) under Bioproject number PRJNA1040959. The genome assembly and annotation data are available at figshare (https://doi.org/10.6084/m9.figshare.24565225.v1).

Author contribution statement

ZY L: Conceptualization, Methodology, Visualization, Formal analysis, Writing - Original Draft, Writing - Review & Editing. **SK S:** Conceptualization, Methodology, Writing - Original Draft, Writing - Review & Editing. **SQ W:** Resources. **XL Z:** Resources. **R Z:** Resources. **GM Y:** Investigation. **JY Z:** Investigation. **WG S:** Investigation.

Funding

This study was supported by the Science and Technology Development Fund of Guidance from the Central Government to Locals (202207AB110016), Major Program for Basic Research Project of Yunnan Province (202101BC070002), National Natural Science Foundation of China (31870529), and the Graduate Scientific Research Fund Project of Yunnan University (KC-22221373).

Conflict of Interest

The authors declare that they have no competing interests.

References:

1. Fang R, Min TL. The floristic study on the genus *Rhododendron*. Acta Botanica Yunnanica. 1995;17:359–79.

2. Chen YS, Deng T, Zhou Z, Sun H. Is the East Asian flora ancient or not? Natl Sci Rev. 2018;5:920–32.

3. Basnett S, Rengaian G. A Comprehensive Review on the taxonomy, ecology, reproductive biology, economic importance and conservation status of Indian Himalayan Rhododendrons. Bot Rev. 2022;88:505–44.

4. Xia XM, Yang MQ, Li CL, Huang SX, Jin WT, Shen TT, Wang F, Li XH, Yoichi W, Zhang LH, Zheng YR, Wang XQ. Spatiotemporal Evolution of the Global Species Diversity of *Rhododendron*. Mol Biol Evol. 2022;39:msab314.

5. Darlington CD, Wylie AP. Chromosome atlas of flowering plants. George Allen and Unwin Ltd. Londonn, UK; 1955. p. 217–8.

6. Zhang J, Peng HW, Xia FC, Wang W. A comparison of seed plants' polyploids between the Qinghai-Tibet Plateau alpine and the Pan-Arctic regions. Biodiversity Science. 2021;29:1470–80.

7. Liu B, Zhao FM, Zhou H, Xia YP, Wang XY. Photoprotection conferring plant tolerance to freezing stress through rescuing photosystem in evergreen *Rhododendron*. Plant Cell Environ. 2022;45:2093–108.

8. Popescu R, Kopp B. The genus Rhododendron: An ethnopharmacological and toxicological review. J Ethnopharmacol. 2013;147:42–62.

9. Guo X, Dong Z, Li Q, Wan DG, Zhong JB, Dong D,Huang MZ. Flavonoids from *Rhododendron nivale* Hook. f delay aging via modulation of gut microbiota and glutathione metabolism. Phytomedicine. 2022;104:154270.

10. Badouin H, Gouzy J, Grassa CJ, Murat F, Staton SE, Cottret L, Lelandais-Briere C, Owens GL, Carrere S, Mayjonade B, et al. The sunflower genome provides insights into oil metabolism, flowering and Asterid evolution. Nature, 2017;546:148–52.

11. Hu YB, Wang XP, Xu YC, Yang H, Tong ZY, Tian R, Xu SH, Yu L, Guo YL, Shi P, et al. Molecular

mechanisms of adaptive evolution in wild animals and plants. Sci China Life Sci. 2023;66:453–95.

12. Marks R. A, Hotaling S, Frandsen P. B, VanBuren R. Representation and participation across 20 years of plant genome sequencing. Nat. Plants. 2021;7:1571–8.

13. Zhang X, Kuang TH, Dong WL, Qian ZH, Zhang HJ, Landis JB, Feng T, Li LJ, Sun YX, Huang JL, et al. Genomic convergence underlying high-altitude adaptation in alpine plants. J Integr Plant Biol. 2023;65:1620–35.

14. De Storme N, Geelen D. The impact of environmental stress on male reproductive development in plant: Biologival processes and molecular mechanisms. Plant Cell Environ. 2014;37:1–18.

15. Chen HT, Zeng Y, Yang YZ, Huang LL, Tang BL, Zhang H, Hao F, Li W, Li YH, Liu YB, et al. Allele-aware chromosome-level genome assembly and efficient transgene-free genome editing for the autotetraploid cultivated alfalfa. Nat Commun. 2020;11:2494.

16. Wang F, Xia ZQ, Zou ML, Zhao L, Jiang SR, Zhou Y, Zhang CJ, Ma YZ, Bao YT, Sun HH, et al. The autotetraploid potato genome provides insights into highly heterozygous species. Plant Biotechnol J. 2022;20:1996–2005.

17. Zhang Q, Qi YY, Pan HR, Tang HB, Wang G, Hua XT, Wang YJ, Lin LY, Li Z, Li YH, et al. Genomic insights into the recent chromosome reduction of autopolyploid sugarcane *Saccharum spontaneum*. Nat Genet. 2022;54:885–96.

18. Zhang HY, He Q, Xing LS, Wang RY, Wang Y, Liu Y, Zhou QH, Li XZ, Jia Z, Liu Z, et al. The haplotype-resolved genome assembly of autotetraploid rhubarb *Rheum officinale* provides insights into the genome evolution and massive accumulation of anthraquinones. Plant Commun. 2023;26:100677.

19. Van de Peer, Y, Ashman TL, Soltis PS, Soltis DE. Polyploidy: an evolutionary and ecological force in stressful times. Plant Cell. 2021;33:11–26.

20. Stevens AV, Nicotra AB, Godfree RC, Guja LK. Polyploidy affects the seed, dormancy and seedling characteristics of a perennial grass, conferring an advantage in stressful climates. Plant Biol. 2020;22:500–13.

21. Liu CJ, Wang YG. Does one subgenome become dominant in the formation and evolution of a polyploid?

Ann Bot. 2023;131:11–6.

22. Wang KL, Deng PR, Yao Z, Dong JY, He Z, Yang P, Liu YB. Biogeographic patterns of polyploid species for the angiosperm flora in China. J Syst Evol. 2022;61:776–89.

23. Heslop-Harrison JS, Schwarzacher T, Liu Q. Polyploidy: its consequences and enabling role in plant diversification and evolution. Ann. Bot. 2023;131:1–10.

24. Cifuentes M, Grandont L, Moore G, Chevre AM, Jenczewski E. Genetic regulation of meiosis in polyploid species: new insights into an old question. New Phytol. 2010;186:29–36.

25. Grandont L, Jenczewski E, Lloyd A. Meiosis and its deviations in polyploid plants. Cytogenet. Genome Res. 2013;140:171–84.

26. Bomblies, K. Learning to tango with four (or more): the molecular basis of adaptation to polyploid meiosis. Plant Reprod. 2022;36:107–24.

27. Gou XW, Bian Y, Zhang A, Zhang HK, Wang B, Lv RL, Li JZ, Zhu B, Gong L, Liu B. Transgenerationally precipitated meiotic chromosome instability fuels rapid karyotypic evolution and phenotypic diversity in an artificially constructed allotetraploid wheat (AADD). Mol, Biol, Evol. 2018;35:1078–91.

28. Huang G, Wu Z, Percy RG, Bai MZ, Li Y, Frelichowski JE, Hu J, Wang K, Yu JZ, Zhu YX. Genome sequence of *Gossypium herbaceum* and genome updates of *Gossypium arboreum* and *Gossypium hirsutum* provide insights into cotton A-genome evolution. Nat. Genet. 2020;52:516–24.

29. Zhang X, Pandey MK, Wang, JP, Zhao KK, Ma XL, Li ZF, Zhao K, Gong FP, Guo BZ, Varshney R, et al. Chromatin spatial organization of wild type and mutant peanuts reveals high-resolution genomic architecture and interaction alterations. Genome Biol. 2021;22:315.

30. Miao Y, Luo D, Zhao T, Du H, Liu Z, Xu Z, Guo L, Chen C, Peng S, Li JX, et al. Genome sequencing reveals chromosome fusion and extensive expansion of genes related to secondary metabolism in *Artemisia argyi*. Plant Biotechnol J. 2022;20:1902–1915.

31. Song AP, Su JS, Wang HB, Zhang ZR, Zhang XT, van de Peer Y, Chen F, Fang WM, Guan ZY, Zhang F, et al. Analyses of a chromosome-scale genome assembly reveal the origin and evolution of cultivated chrysanthemum. Nat Commun. 2023;14:2021.

32. Xu P, Xu J, Liu G, Chen L, Zhou ZX, Peng WZ, Jiang YL, Zhao ZX, Jia ZY, Sun YH et al. The allotetraploid origin and asymmetrical genome evolution of the common carp *Cyprinus carpio*. Nat Commun. 2019;10:4625.

33. Xu J, Wang XY, Guo WZ. The cytochrome P450 superfamily: key players in plant development and defense. J Integr Agric. 2015;14:1673–86.

34. Feng K, Hou XL, Xing GM, Liu JX, Duan AQ, Xu ZS, Li MY, Zhuang J, Xiong AS. Advances in AP2/ERF super-family transcription factors in plant. Crit Rev Biotechnol. 2020;40:750–76.

35. Shen SK, Zhou XL, Wang SQ, Lyu ZY, Zhang R, Liu Y, Long B. Protect fragile mountaintop ecosystems. Science. 2023;380:1114–5.

36. Wu XP, Zhang L, Wang XY, Zhang RA, Jin GH, Hu YT, Yang H, Wu ZZ, Ma YP, Zhang CJ, Wang JH. Evolutionary history of two evergreen Rhododendron species as revealed by chromosome-level genome assembly. Front. Plant Sci. 2023;14:1123707.

37. Blumthaler M, Ambach W, R Ellinger. Increase in solar UV radiation with altitude. J Photochem Photobiol B. 1997;39:130–4.

38 89. McKenzie RL, Johnston PV, Smale D, Bodhaine BA, Madronich S. Altitude effects on UV spectral irradiance deduced from measurements at Lauder, New Zealand, and at Mauna Loa Observatory, Hawaii. J Geophys Res-Atmos. 2001;106:22845–22860.

39. Dahlback A, Gelsor N, Stamnes JJ, Gjessing Y. UV measurements in the 3000-5000 m altitude region in Tibet. J Geophys Res-Atmos. 2007;112:1–10.

40. Kerstiens G. Cuticular water permeability and its physiological significance. J Exp Bot. 199647:1813–32.

41. Tossi V, Lombardo C, Cassia R, Lamattina L. Nitric oxide and flavonoids are systemically induced by UV-B in maize leaves. Plant Sci. 2012;193:103–9.

42. Tohge T, Wendenburg R, Ishihara H, Nakabayashi R, Watanabe M, Sulpice R, Hoefgen R, Takayama H, Saito K, Stitt M, Fernie AR. Characterization of a recently evolved flavonol-phenylacyltransferase gene provides signatures of natural light selection in Brassicaceae. Nat Commun. 2016;7:12399.

43. Choi YE, Lim S, Kim HJ, Han JY, Lee MH, Yang Y, Kim JA, Kim YS. Tobacco *NtLTP1*, a glandular-specific lipid transfer protein, is required for lipid secretion from glandular trichomes. Plant J. 2012;70:480–91.

44. Mamrutha HM, Nataraja KN, Rama N, Kosma DK, Mogili T, Lakshmi KJ, Kumar MU, Jenks MA. Leaf surface wax composition of genetically diverse mulberry (*Morus* sp.) genotypes and its close association with expression of genes involved in wax metabolism. Curr Sci. 2017;112:759–66.

45. Trivedi P, Nguyen N, Klavins L, Kviesis J, Heinonen E, Remes J, Jokipii-Lukkari S, Klavins M, Karppinen K, Jaakola L, Haggman H. Analysis of composition, morphology, and biosynthesis of cuticular wax in wild type bilberry (*Vaccinium myrtillus* L.) and its glossy mutant. Food Chem. 2021;354:12957.

46. Gao HN, Jiang H, Lian XY, Cui JY, You CX, Hao YJ, Li YY. Identification and functional analysis of the *MdLTPG* gene family in apple. Crit. Rev. Biotechnol. 2021;163:338–47.

47. Emiliani J, Grotewold E, Ferreyra MLF, Casati P. Flavonols protect Arabidopsis plants against UV-B deleterious effects. Mol Plant. 2013;6:1376–9.

48. McKenzie R, Conner B, Bodeker G. Increased summertime UV radiation in New Zealand in response to ozone loss. Science. 1999;285:1709–11.

49. Sturm A, Lienhard S. Two isoforms of plant *RAD23* complement a UV-sensitive *RAD23* mutant in yeast. Plant J. 1998;13:815–21.

50. Lahari T, Lazaro J, Schroeder DF. *RAD4* and *RAD23/HMR* Contribute to Arabidopsis UV Tolerance. Genes. 2018;9:8.

51. Mohl P, von Buren R. S, Hiltbrunner E. Growth of alpine grasslandwill start and stop earlier under climate warming. Nat Commun. 2022;13:7398.

52. Korner C. Concepts in alpine plant ecology. Plants. 2023;12:2666.

53. Li YH, Zheng LY, Corke F, Smith C, Bevan MW. Control of final seed and organ size by the *DA1* gene family in *Arabidopsis thaliana*. Genes Dev. 2008;22:1331–6.

54. Titapiwatanakun B, Blakeslee JJ, Bandyopadhyay A, Yang H, Mravec J, Sauer M, Cheng Y, Adamec J, Nagashima A, Geisler M, et al. *ABCB19/PGP19* stabilises *PIN1* in membrane microdomains in *Arabidopsis*.

Plant J. 2009;57:27–44.

55. Gao, YS, Badejo AA, Sawa Y, Ishikawa T. Analysis of two l-Galactono-1,4-Lactone-Responsive genes with complementary expression during the development of *Arabidopsis thaliana*. Plant Cell Physiol. 2012;53:592–601.

56. Sun H, Niu Y, Chen YS, Song B, Liu CQ, Peng DL, Chen JG, Yang Y. Survival and reproduction of plant species in the Qinghai-Tibet Plateau. J Syst Evol. 2014;52:378–96.

57. Zhu JB, He HD, Zhang FW, Li HQ, Li YN, Yang YS, Wang CY, Zhang GR, Luo FL. Effect of temperature difference between day and night on NEE and its variation Characteristics in alpine shrubland in Qinghai-Tibetan Plateau. Research of Soil and Water Conservation. 2020;27:232–8.

58. Hu XP. The leaf stomatal characters of *Ligularia virgaurea* and their responses to alpine climate. Acta Agrestia Sinica. 2016;24:1283–9.

59. Abbas M, Sharma G, Dambire C, Marquez J, Alonso-Blanco C, Proano K, Holdsworth MJ. An oxygen-sensing mechanism for angiosperm adaptation to altitude. Nature. 2022;606:565–9.

60. Clouse SD, Sasse JM. Brassinosteroids: Essential regulators of plant growth and development. Annu Rev Plant Physiol, Plant Mol Biol. 1998;49:427–51.

61. Chaudhuri A, Halder K, Abdin MZ, Majee M, Datta A. Abiotic stress tolerance in plants: brassinosteroids navigate competently. Int J Mol Sci. 2022;23:14577.

62. Zhan HD, Lu MM, Luo Q, Tan F, Zhao ZW, Liu MQ, He YB. *OsCPD1* and *OsCPD2* are functional brassinosteroid biosynthesis genes in rice. Plant Sci. 2022;325:111482.

63. Zheng XY, Li P, Lu X. Research advances in cytochrome P450-catalysed pharmaceutical terpenoid biosynthesis in plants. J Exp Bot. 2019;70:4619–30.

64. Nakano T, Suzuki K, Fujimura T, Shinshi H. Genome-wide analysis of the ERF gene family in Arabidopsis and rice, Plant Physiol. 2006;140:411–432.

65. Thomashow MF. Plant cold acclimation: Freezing tolerance genes and regulatory mechanisms. Annu Rev Plant Physiol Plant Mol Biol. 1999;50:571–99.

66. Cao K, Zhang ZY, Fan H, Tan Y, Xu HW, Zhou XF. Comparative transcriptomic analysis reveals gene

expression in response to cold stress in *Rhododendron aureum* Georgi. Theor Exp Plant Physiol. 2022;34:347–66.

67. Liu T, Bin L, Duan JX, Xu ZQ, Jiang H, Wang QB. Geochemical characteristics of *Rhododendron nivale* Hook. f. and its indication for concealed Lithium deposits in Jiajika rare metal mining Area. Geological Journal of China Universities. 2022;28:32–9.

68. Zhang QY, Li Y, Cao K, Xu HW, Zhou XF. Transcriptome and proteome depth analysis indicate ABA, MAPK cascade and Ca^{2+} signaling co-regulate cold tolerance in *Rhododendron chrysanthum* Pall Front Plant Sci. 2023;14:1146663.

69. Song MJ, Potter BI, Doyle JJ, Coate JE. Gene balance predicts transcriptional responses immediately following ploidy change in *Arabidopsis thaliana*. Plant Cell. 2020;32:1434–48.

70. Zhou RC, Moshgabadi N, Adams KL. Extensive changes to alternative splicing patterns following allopolyploidy in natural and resynthesized polyploids. Proc. Natl. Acad Sci USA. 2011;108:16122–7.

71. Staiger D, Brown JWS. Alternative splicing at the intersection of biological timing, development, and stress responses. Plant Cell. 2013;25:3640–56.

72. Mao HT, Chen MY, Su YQ, Wu N, Yuan M, Yuan S, Brestic M, Zivcak M, Zhang HY, Chen Y. Comparison on photosynthesis and antioxidant defense systems in wheat with different ploidy levels and octoploid Triticale. Int J Mol Sci. 2018;19:3006.

73. Comai L. The advantages and disadvantages of being polyploid. Nat Rev Genet. 2005;6:836–46.

74. Singliarova B, Hojsgaard D, Muller-Scharer H, Mraz P. The novel expression of clonality following whole-genome multiplication compensates for reduced fertility in natural autopolyploids. Proc Biol Sci. 2023;290:20230389.

75. Morgan C, Zhang HK, Henry CE, Franklin FCH, Bomblies K. Derived alleles of two axis proteins affect meiotic traits in autotetraploid *Arabidopsis arenosa*. Proc Natl Acad Sci USA. 2020;117:8980–8988.

76. Seear PJ, France MG, Gregory CL, Heavens D, Schmickl R, Yant L, Higgins JD. A novel allele of *ASY3* is associated with greater meiotic stability in autotetraploid *Arabidopsis lyrata*. PLoS Genet. 2020;16:e1008900.

77. Marcais G, Kingsford C. A fast, lock-free approach for efficient parallel counting of occurrences of *k-mers*. Bioinformatics. 2011;27:764–70.

78. Ranallo-Benavidez TR, Jaron KS, Schatz MC. GenomeScope 2.0 and Smudgeplot for reference-free profiling of polyploid genomes. Nat Commun. 2020;11:1432.

79. Cheng HY, Concepcion T, Feng XW, Zhang HW, Li H. Haplotype-resolved de novo assembly using phased assembly graphs with hifiasm. Nat Methods. 2021;18:170–5.

80. Koren S, Rhie A, Walenz BP, Dilthey AT, Bickhart DM, Kingan SB, Hiendleder S, Williams JL, Smith TPL, Phillippy AM. De novo assembly of haplotype-resolved genomes with trio binning. Nat Biotechnol. 2018;36:1174–82.

81. Nurk S, Walenz BP, Rhie A, Vollger MR, Logsdon GA, Grothe R, Miga KH, Eichler EE, Phillippy AM, Koren S. HiCanu: accurate assembly of segmental duplications, satellites, and allelic variants from high-fidelity long reads. Genome Res. 2020;30:1291–305.

82. Zhang XT, Zhang SC, Zhao Q, Ming R, Tang HB. Assembly of allele-aware, chromosomal-scale autopolyploid genomes based on Hi-C data. Nat Plants. 2019;5:833–845. doi:10.1038/s41477-019-0487-8.

83. Durand NC, Robinson JT, Shamim S, Machol I, Mesirov P, Lander ES, Aiden EL. Juicebox provides a visualization system for Hi-C contact maps with unlimited zoom. Cell Syst. 2016;3:99–101.

84. Simao FA, Waterhouse RM, Ioannidis P, Kriventseva EV, Zdobnov EM. BUSCO: assessing genome assembly and annotation completeness with single-copy orthologs. Bioinformatics. 2015;31:3210–2.

85. Rhie A, Walenz BP, Koren S, Phillippy AM. Merqury: reference-free quality, completeness, and phasing assessment for genome assemblies. Genome Biol. 2020;21:245.

86. Li KP, Xu P, Wang JP, Yi X, Jiao YN. Identification of errors in draft genome assemblies at single-nucleotide resolution for quality assessment and improvement. Nat Commun. 2023;14:6556.

87. Li H, Durbin R. Fast and accurate short read alignment with Burrows-Wheeler transform. Bioinformatics. 2009;25:1754–60.

88. Li H, Handsaker B, Wysoker A, Fennell T, Ruan J, Homer N, Marth G, Abecasis G, Durbin R. The Sequence Alignment/Map format and SAMtools. Bioinformatics, 2009;25:2078–9.

89. Ellinghaus D, Kurtz S, Willhoeft U. LTRharvest, an efficient and flexible software for de novo detection of LTR retrotransposons. BMC Bioinformatics. 2008;9:18.

90. Zhao X, Wang H. LTR_FINDER: an efficient tool for the prediction of full-length LTR retrotransposons. Nucleic Acids Res. 2007;35:W265–W268.

91. Ou SJ, Jiang N. LTR_retriever: a highly accurate and sensitive program for identification of long terminal repeat retrotransposons. Plant Physiol. 2018;176:1410–22.

92. Tarailo-Graovac M, Chen NS. Using RepeatMasker to identify repetitive elements in genomic sequences. Current protocols in bioinformatics. 2009. Chapter 4:4.10.1–4.10.14. doi:10.1002/0471250953.bi0410s25

93. Lowe TM, Eddy SR. tRNAscan-SE: A program for improved detection of transfer RNA genes in genomic sequence. Nucleic Acids Res. 1997;25:955–64.

94. Lagesen K, Hallin P, Rodland EA, Staerfeldt HH, Rognes T, Ussery DW. RNAmmer: consistent and rapid annotation of ribosomal RNA genes. Nucleic Acids Res. 2007;35:3100–8.

95. Nawrocki EP, Burge SW, Bateman A, Daub J, Eberhardt RY, Eddy SR, Floden EW, Gardner PP, Jones TA, Tate J, et al. Rfam 12.0: updates to the RNA families database. Nucleic Acids Res. 2015;43:D130–D137.

96. Nawrocki EP, Eddy SR. Infernal 1.1: 100-fold faster RNA homology searches. Bioinformatics. 2013;29:2933–5.

97. Hoff KJ, Lange S, Lomsadze A, Borodovsky M, Stanke M. BRAKER1: Unsupervised RNA-Seq-Based fenome annotation with GeneMark-ET and AUGUSTUS. Bioinformatics. 2016;32:767–769.

98. Kim D, Paggi JM, Park C, Bennett C, Salzberg SL. Graph-based genome alignment and genotyping with HISAT2 and HISAT-genotype. Nat Biotechnol. 2019;37:907–15.

99. Haas BJ, Papanicolaou A, Yassour M, Grabherr M, Blood PD, Bowden J, Couger MB, Eccles D, Li B, Lieber M, et al. De novo transcript sequence reconstruction from RNA-seq using the Trinity platform for reference generation and analysis. Nat. Protoc. 2013;8:1494–512.

100. Pertea M, Pertea GM, Antonescu CM, Chang TC, Mendell JT, Salzberg SL. StringTie enables improved reconstruction of a transcriptome from RNA-seq reads. Nat Biotechnol. 2015;33:290–5.

101. Haas BJ, Delcher AL, Mount SM, Wortman JR, Smith RK, Hannick LI, Maiti R, Ronning CM, Rusch

DB, Town CD, et al. Improving the *Arabidopsis* genome annotation using maximal transcript alignment assemblies. Nucleic Acids Res. 2003;31:5654–66.

102. Korf I. Gene finding in novel genomes. BMC Bioinformatics. 2004;5:59. doi:10.1186/1471-2105-5-59.

103. Blanco E, Genis P, Roderic G. Using geneid to identify genes. Current protocols in bioinformatics. 2007. 4:4.3.1–4.3.28.

104. Keilwagen J, Hartung F, Grau J. GeMoMa: Homology-Based gene prediction utilizing intron position conservation and RNA-seq data. Methods Mol Biol. 2019;1962:161–77.

105. Haas BJ, Salzberg SL, Zhu W, Pertea M, Allen J. E, Orvis J, White O, Buell CR, Wortman JR. Automated eukaryotic gene structure annotation using EVidenceModeler and the program to assemble spliced alignments. Genome Biol. 2008;9:R7.

106. Huerta-Cepas J, Szklarczyk D, Heller D, Hernandez-Plaza A, Forslund SK, Cook H, Mende DR, Letunic I, Rattei T, Jensen LJ, et al. eggNOG 5.0: a hierarchical, functionally and phylogenetically annotated orthology resource based on 5090 organisms and 2502 viruses. Nucleic Acids Res. 2019;47:D309–D314.

107. McGinnis S, Madden TL. BLAST: at the core of a powerful and diverse set of sequence analysis tools. Nucleic Acids Res. 2004;32:W20–W25.

108. Nguyen LT, Schmidt HA, von Haeseler A, Minh BQ. IQ-TREE: A fast and effective stochastic algorithm for estimating maximum-likelihood phylogenies. Mol Biol Evol. 2015;32:268–74.

109. Emms DM, Kelly S. OrthoFinder: phylogenetic orthology inference for comparative genomics. Genome Biol. 2019;20:238.

110. Edgar RC. MUSCLE: multiple sequence alignment with high accuracy and high throughput. Nucleic Acids Res. 2004;32:1792–7.

111. Capella-Gutierrez S, Silla-Martinez JM, Gabaldon T. trimAl: a tool for automated alignment trimming in large-scale phylogenetic analyses. Bioinformatics. 2009;25:1972–3.

112. Yang ZH. PAML 4: Phylogenetic analysis by maximum likelihood. Mol. Biol. Evol. 2007;24:1586–91.

113. Ma YZ, Mao XX, Wang J, Zhang L, Jiang YZ, Geng YY, Ma T, Cai LM, Huang SQ, Hollingsworth P, et al. Pervasive hybridization during evolutionary radiation of *Rhododendron* subgenus *Hymenanthes* in

mountains of southwest China. Natl. Sci. Rev. 2022;9:nwac276.

114. Mendes FK, Vanderpool D, Fulton B, Hahn MW. CAFE 5 models variation in evolutionary rates among gene families. Bioinformatics. 2020;36:5516–8.

115. Wu TZ, Hu EQ, Xu SB, Chen MJ, Guo PF, Dai ZH, Feng TZ, Zhou L, Tang WL, Zhan L, et al. clusterProfiler 4.0:A universal enrichment tool for interpreting omics data. Innovation. 2021;2:100141.

116. Wang YP, Tang HB, DeBarry JD, Tan X, Li JP, Wang XY, Lee TH, Jin HZ, Marler B, Guo H, et al. MCScanX: a toolkit for detection and evolutionary analysis of gene synteny and collinearity. Nucleic Acids Res. 2012;40:e49.

117. Wang Dapeng, Zhang YB, Zhang Z, Zhu J, Yu J. KaKs_Calculator 2.0: a toolkit incorporating gamma-series methods and sliding window strategies. Genom Proteom Bioinf. 2010;8:77–80.

118. Zhang Z, Xiao JF, Wu JY, Zhang HY, Liu GM, Wang XM, Dai L. ParaAT: A parallel tool for constructing multiple protein-coding DNA alignments. Biochem Biophys Res Commun. 2012;419:779–881.

119. Yang FS, Nie S, Liu, H, Shi TL, Tian XC, Zhou SS, Bao YT, Jia KH, Gou JF, Zhao W, et al. Chromosome-level genome assembly of a parent species of widely cultivated azaleas. Nat Commun. 2020;11:5269.

120. Pond SLK, Frost SDW, Muse SV. HyPhy: hypothesis testing using phylogenies. Bioinformatics. 2005;21:676–9. doi:10.1093/bioinformatics/bti079.

121. Dobin A, Davis CA, Schlesinger F, Drenkow J, Zaleski C, Jha S, Batut P, Chaisson M, Gingeras TR. STAR: ultrafast universal RNA-seq aligner. Bioinformatics. 2013;29:15–21.

122. Li B, Dewey CN. RSEM: accurate transcript quantification from RNA-Seq data with or without a reference genome. BMC Bioinformatics. 2011;12:323.

123. Zhang JS, Zhang XT, Tang HB, Zhang Q, Hua XT, Ma XK, Zhu F, Jones T, Zhu XG, Bowers J, et al. Allele-defined genome of the autopolyploid sugarcane *Saccharum spontaneum* L. Nat Genet. 2018;50:1565–73.

124. Qiao X, Li QH, Yin H, Qi KJ, Li LT, Wang RZ, Zhang SL, Paterson AH. Gene duplication and evolution in recurring polyploidization-diploidization cycles in plants. Genome Biol. 2019;20:38.

125. Katoh K, Standley DM. MAFFT multiple sequence alignment software version 7: improvements in performance and usability. Mol Biol Evol. 2013;30:772–80.

126. Chen CJ, Chen H, Zhang Y, Thomas HR, Frank MH, He YH, Xia R. TBtools: an integrative toolkit developed for interactive analyses of big biological data. Mol Plant. 2020;13:1194–202.

127. Librado P, Vieira FG, Rozas J. BadiRate: estimating family turnover rates by likelihood-based methods. Bioinformatics. 2012;28:279–81.

Figure titles

Figure 1. Habitat and genomic characteristics of *R. nivale* subsp. *boreale*. A. habitat; B. habit; C. genome landscape, a, 52 pseudochromosomes, which belong to 13 homologous groups, and the length of the pseudochromosome; b, gene density; c, GC density; d, transposon element density; e, copia density; f, gypsy density; g, tandem repeat density; curved lines inside the circles linked syntenic genes between different pseudochromosomes, the synteny between haplotype 1 and haplotype 2 is in red, the synteny between haplotype 1 and haplotype 3 is in green, the synteny between haplotype 1 and haplotype 4 is in yellow. D. Hi-C heatmap for assembled pseudochromosomes; E. Smudgeplot analysis based on 21 *k-mers*.

Figure 2. Phylogenetic and comparative analysis between related species and haplotypes. A. Dot plot between *R. nivale* subsp. *boreale* and *R. ovatum*; B. syntenic blocks between four haplotypes; C. phylogenetic relationships of Subsect. *Lapponica*, yellow and green block showing *R. nivale* and sister clade of *R. nivale*, respectively, red block represents the data for this study and the downloaded species data were represented by blue block; D. gene families characteristics between four haplotypes.

Figure 3. Comparative genomic analysis. A. Phylogenetic tree showing the relationship of *R. nivale* subsp. *boreale* and 18 other species. Estimated divergence times (Mya, million years ago) are labeled at nodes in black. Bootstrap values are displayed on the nodes in circles (100%) and squares (≥ 95%). Expansion (orange) and contraction (blue) of gene families are shown on the branch, contraction and expansion of ancestors are represented by a pie chart, and extant species are displayed via numbers. The WGD and WGT events are marked with D and T, respectively. B. A number of other genes, unique, multicopy and single-copy in 19 species. C. *Ks* frequency distribution chart of seven species, including six Ericales (*Actinidia*, *Vaccinium* and Subg. *Hymenanthes*, Subg. *Furthermore*, Subg. *Rhododendron*, Subg. *Tsutsusi* one species each) and one *Vi. vinifera*, polyploidization events are represented by dotted lines. D. Homologous gene dot plots between *R. nivale* subsp. *boreale* and *Vi. vinifera*. The red box demonstrates an example of the orthologous ratio of 1:2 between *Vi. vinifera* and *R. nivale* subsp. *boreale*.

Figure 4. KEGG and GO enrichment and gene duplication analysis of *R. nivale* subsp. *boreale*. A. KEGG (left) and GO (right) enrichment of genes in significantly expanded gene families. B. Venn diagram showing the number of shared and specific gene duplications between the significantly expanded genes (SEGs) and five categories of duplications (DSD, dispersed duplications; PD, proximal duplications; TD, tandem duplications; TRD, transposed duplications; WGD, whole genome duplications). C. *Ka/Ks* ratios of the five types of duplications. D. KEGG pathway enrichment analysis of five duplication types.

Figure 5. Single-matched allelic expression analysis. A. The total amount of single-match allelic expression of 52 pseudochromosomes; the colors represent 13 homologous groups (HGs). B. Heatmap clustering analysis of single-match alleles in screening their expression levels in *R. nivale* subsp. *boreale*. Each row represents a set of alleles, and each column represents a haplotype. A heatmap shows a homologous group.

Figure 6. Identification and evolution of key family and genes for adaptation to mountaintop low temperature and hypoxia. A. Rootless phylogenetic tree based on ultrafast 1,000 bootstrap samplings showed a diversified AP2/ERF superfamily in 13 species, including 10 *Rhododendron* species and *Ar. thaliana*, Kiwifruit, *Va. darrowii*. The color of clades indicates 5 subfamilies of the AP2/ERF superfamily. The labels are differently colored according to species. B. Schematic diagram of the gain and loss of key genes in 12 species of Ericales;

pink and blue numbers depict *ERF VII* and *CBF* gene family turnover. The numbers in the rectangles and circles represent the number of genes in ancestors and existing species. The + and − signs represent the gain and loss of genes, respectively. C. Expression levels of *ERF VIIs* and *CBFs*. The number on the line of maximum value shows the number of genes expressed.

Figure 7. Characteristics of Ericales Cytochrome P450 (CYP). A. Phylogenetic tree showing the relationship of 10 clans (higher order groupings of CYP families). B. The number of clan members for each species is indicated by the heatmap. C. Phylogenetic tree of the CYP members of 13 species based on GTR (generalized time-reversible). Different clans are represented by different colors. D. Numbers of CYP genes produced by duplication events in 10 species of *Rhododendron*.

Figure 1

Click here to access/download;Figure;Fig1.pdf

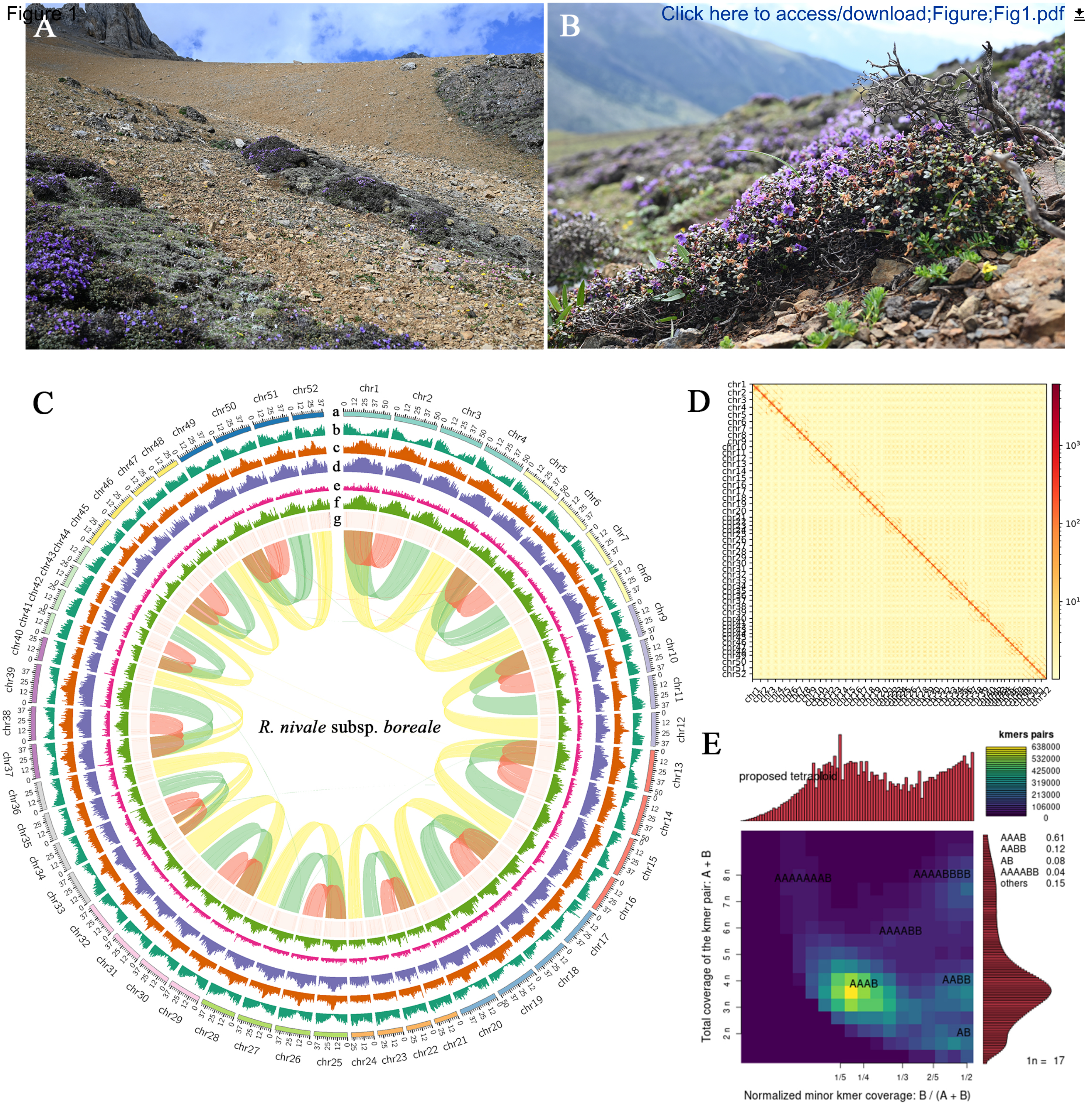

Figure 2

Click here to access/download;Figure;Fig2.pdf

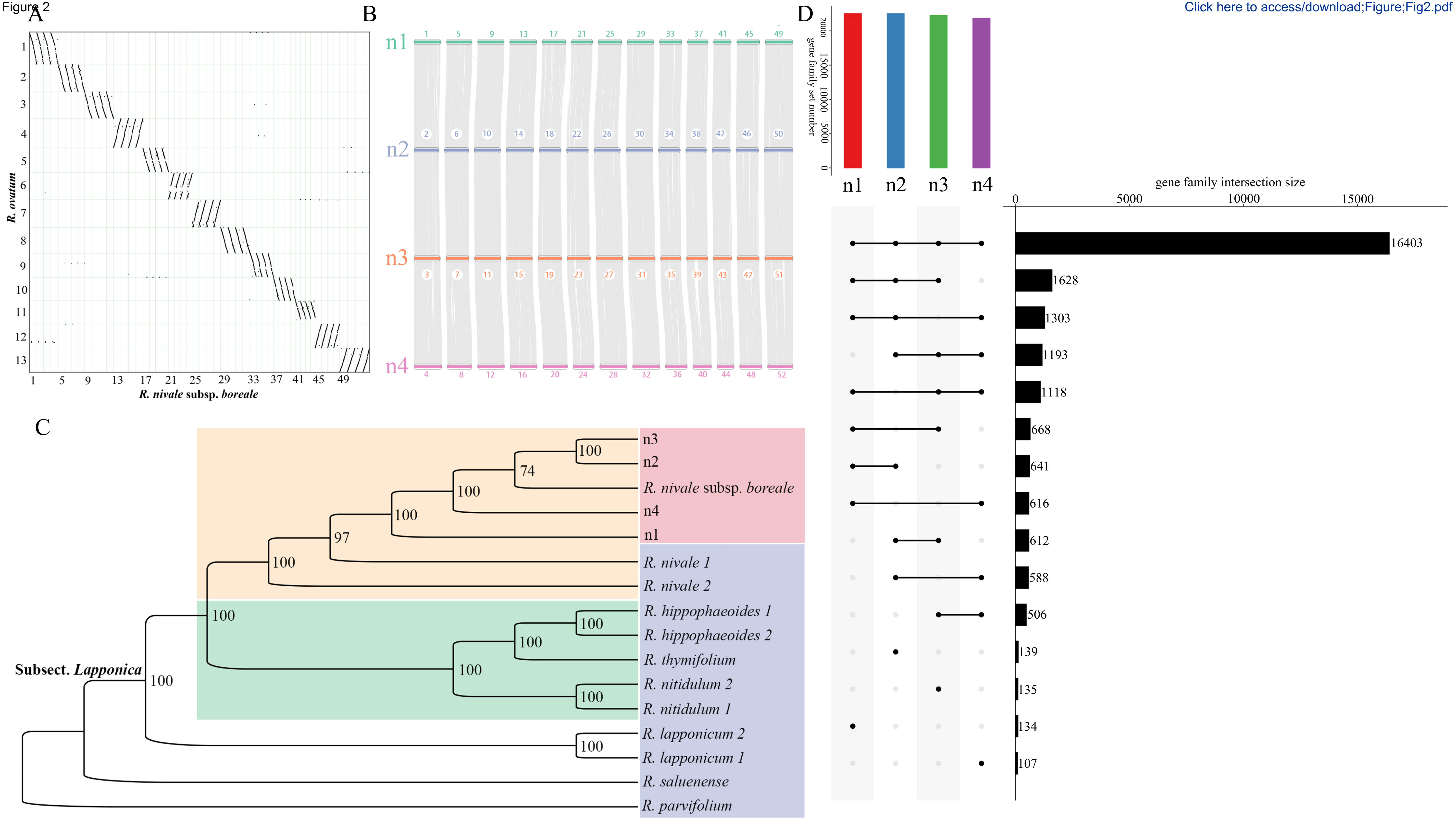

Figure 3

Click here to access/download;Figure;Fig3.pdf

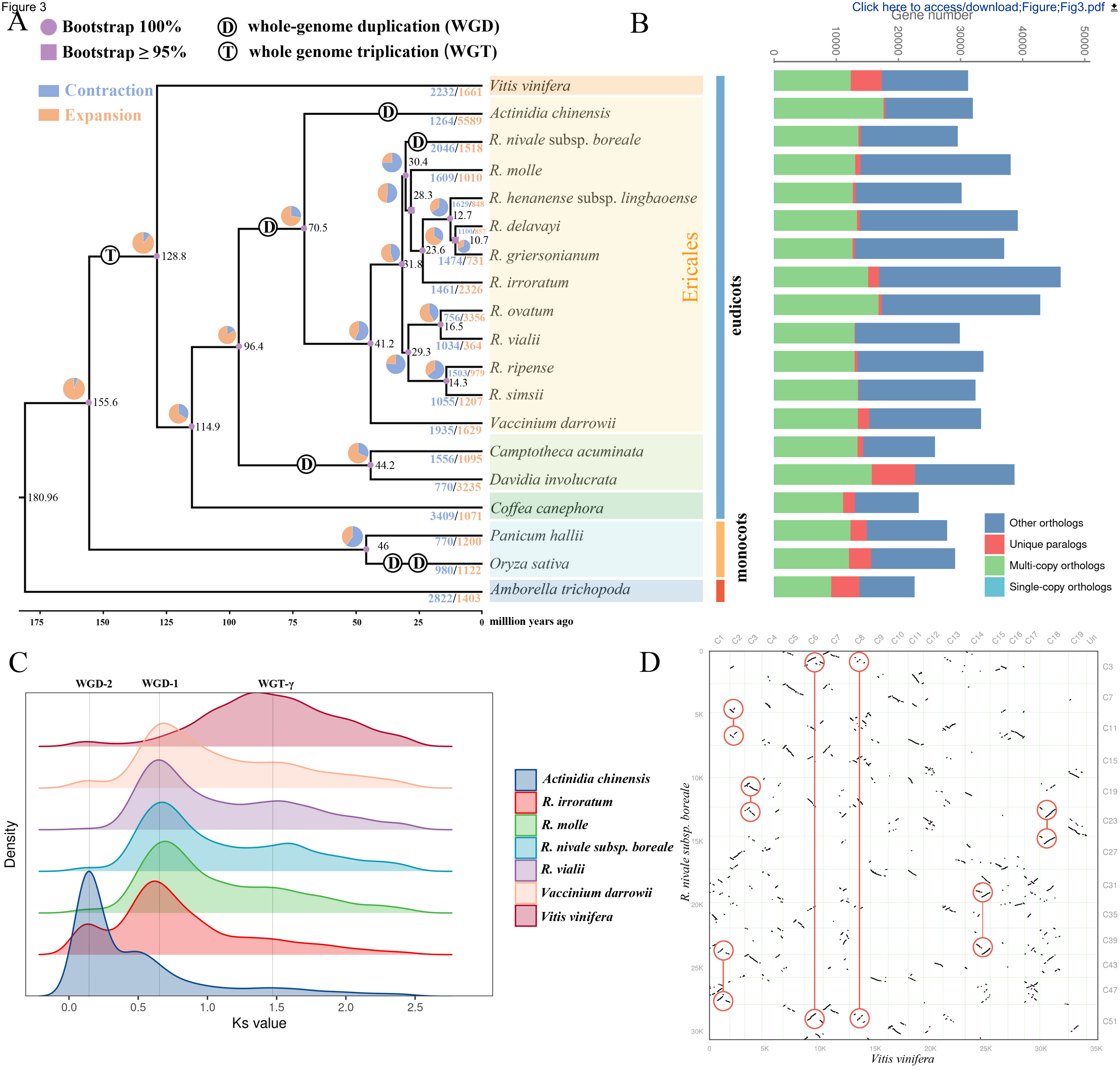

Figure 4

Click here to access/download;Figure;Fig4.pdf

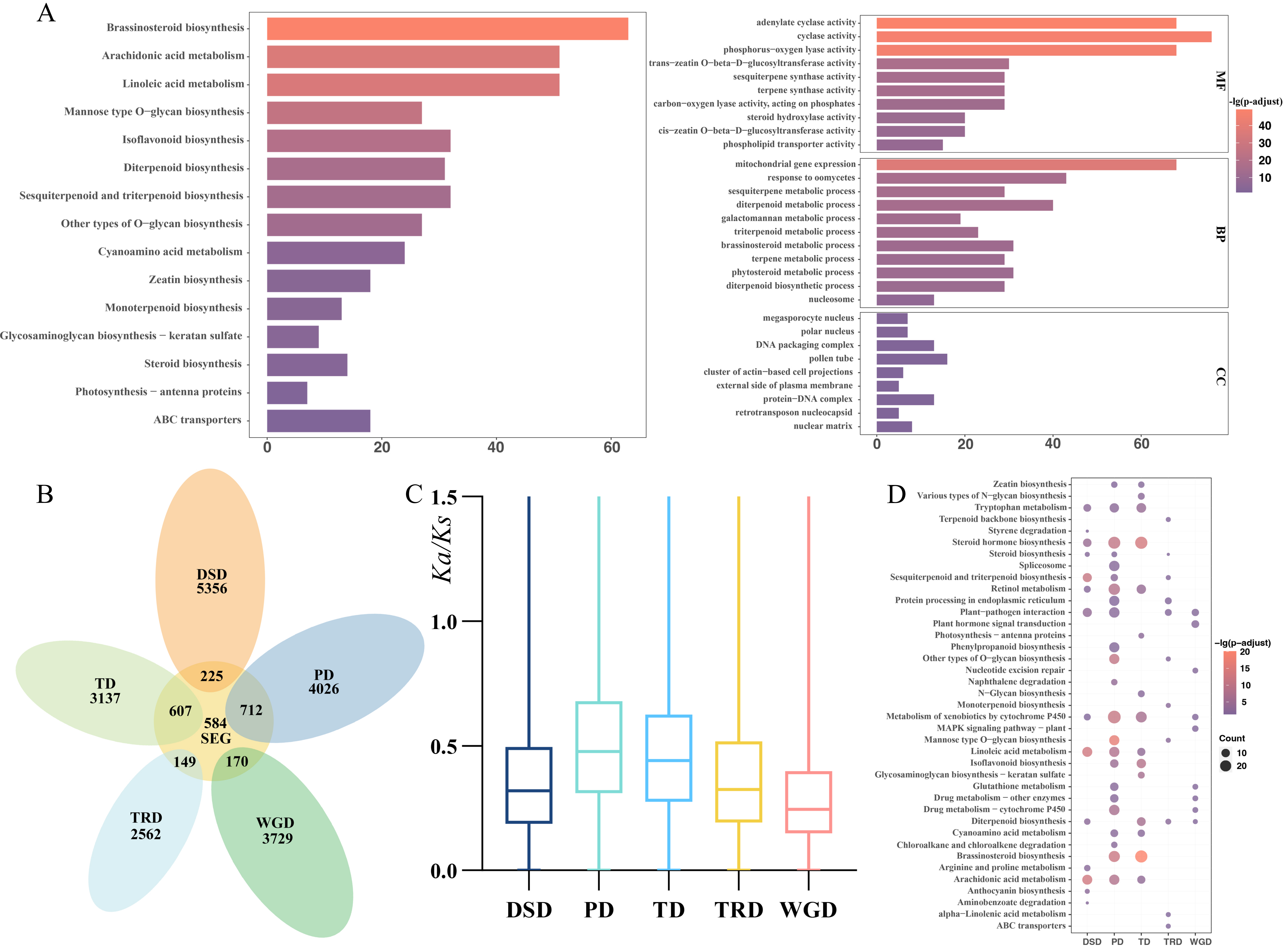

Figure 5

Click here to access/download;Figure;Fig5.pdf

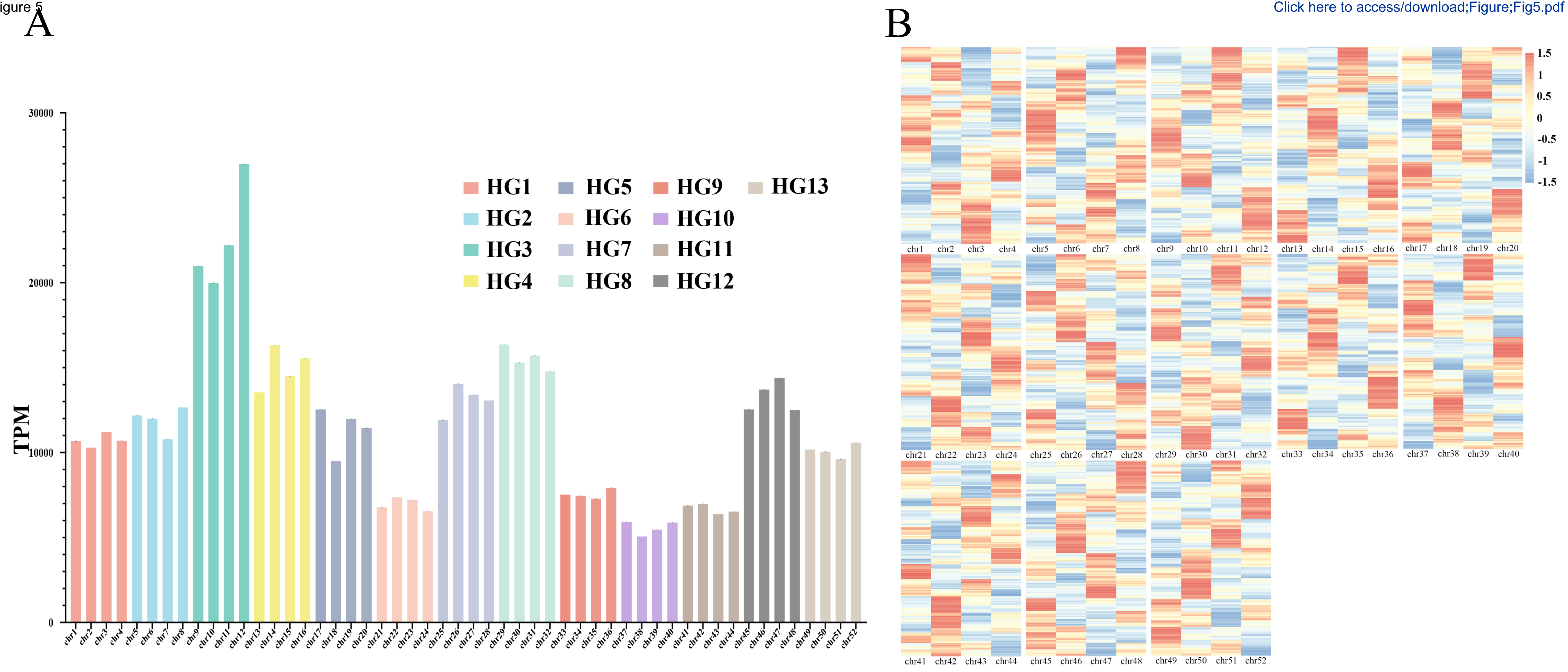

Figure 6

Click here to access/download;Figure;Fig6.pdf

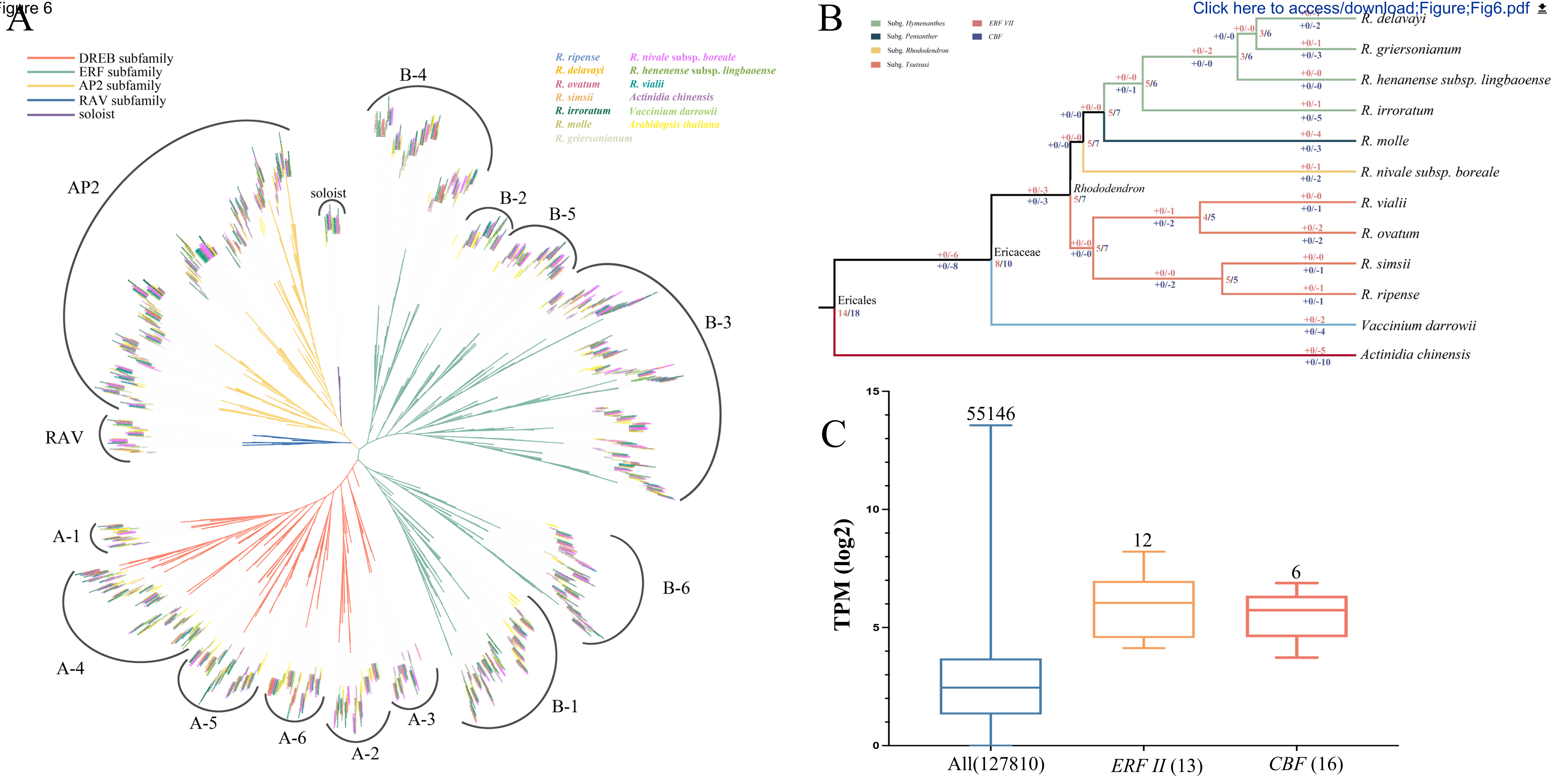

Figure 7

Click here to access/download;Figure;Fig7.pdf

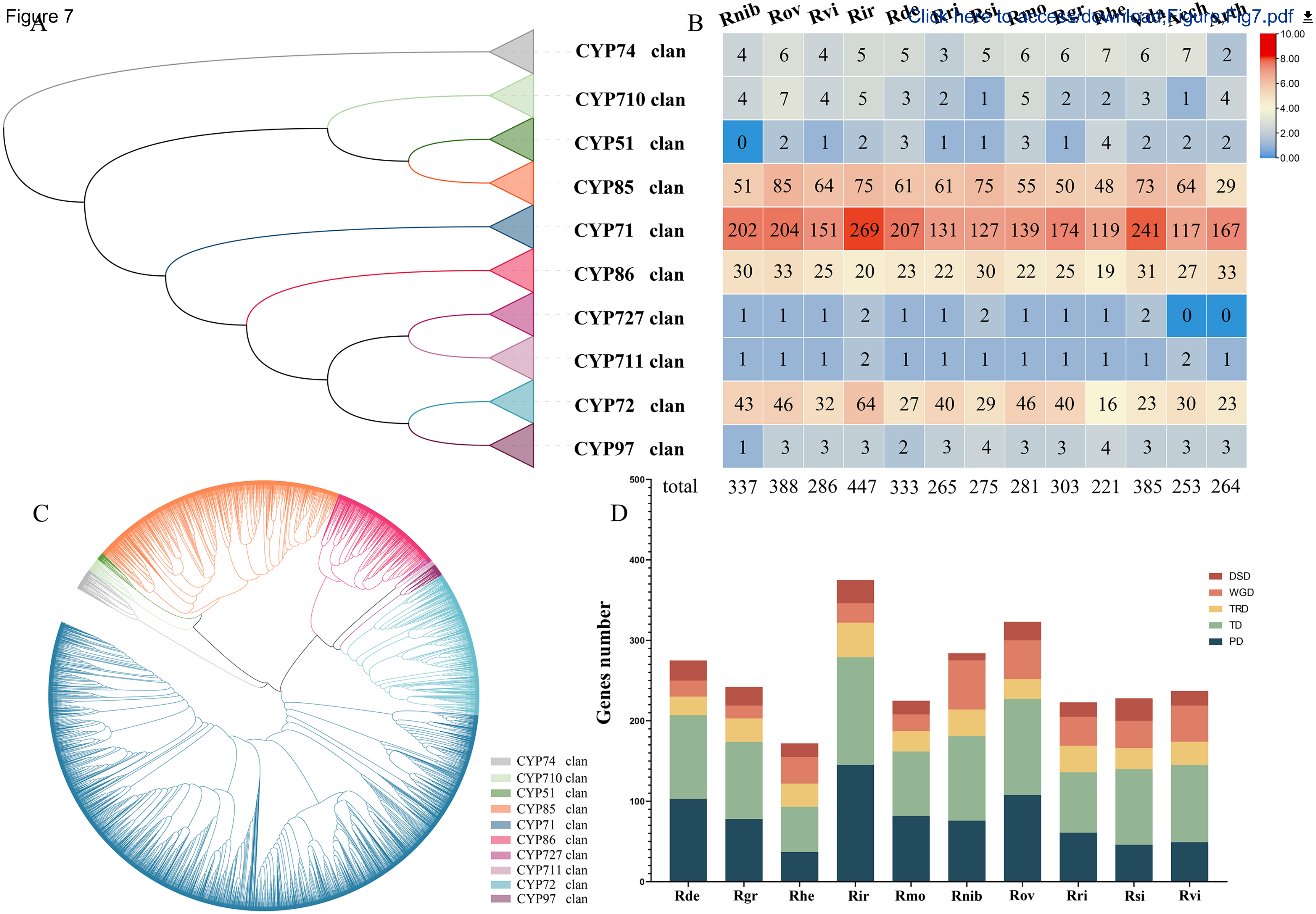

Click here to access/download
Supplementary Material
Supplememtary Figure.docx

Supplementary Table

Click here to access/download
Supplementary Material
Supplementary Table.xlsx

Personal Cover
Click here to access/download;Personal Cover;cover_letter.docx

December 18, 2023

Dear Editor,

With my co-workers, I would like to submit our manuscript entitled "The first high-altitude autotetraploid haplotype-resolved genome assembled (*Rhododendron nivale* subsp. *boreale*) provides new insights into mountaintop adaptation" to Gigascience.

The manuscript addresses the intricate relationship between high-altitude environments and polyploidization, with a focus on the woody flowering species *R. nivale* subsp. *boreale*. This species, thriving in the delicate subalpine to alpine transition zone, offers a unique opportunity to unravel the evolutionary mechanisms and adaptation strategies of alpine woody plants.

In our study, we present the comprehensive assembly and annotation of the first high-altitude autotetraploid haplotype-resolved genome of *Rhododendron nivale* subsp. *boreale* (scaffold N50=42.93 Mb; BUSCO=98.8%; QV=45.51; S-AQI=98.69), derived from the challenging terrain of the Qinghai-Tibet Plateau (4387.5 m). Leveraging a combination of comparative genomics, transcriptome analysis, and gene family scrutiny, we unveil profound insights into the evolutionary history, gene family dynamics, and the evolution of critical genes. **Notably, our investigation** 1) exposes special paleopolyploidy events dating back 78 million years. 2) The cell wall modification, flavonoid biosynthesis, DNA repair, inhibition of chlorophyll synthesis, auxin and brassinosteroid biosynthesis and transduction are the main high-altitude adaptation pathways. 3) Key genes, namely *ERF VIIs* and *CBFs*, exhibit consistent loss yet maintain high expression levels. 4) The expansion of gene families is intricately linked to brassinosteroid biosynthesis, while the expanded CYP family correlates with these evolutionary processes.

Novelty lies in 1) In a context where only a few autopolyploid genomes have been published, we provide a rare high-altitude autotetraploid haplotype-resolved genome; 2) we combined diverse methods (*k-mer*, collinearity and phylogeny) to establish the autotetraploid identity of *R. nivale* subsp. *boreale*. Simultaneously, 3) Predominance of polyploid expression and phytohormones (such as brassinosteroid) play an important role in adaptation of *R. nivale* subsp. *boreale* to high altitude. In addition, 4) our identification of positively selected genes, including *ASY3* associated with autotetraploid meiosis control, provides a pathway for a deeper understanding of the gametic formation mechanisms inherent in natural autopolyploids. Collectively, these results furnish valuable genetic resources, contributing significantly to our comprehension of the origin and

evolution of high-altitude polyploids. We believe our work not only contributes to domestication of high-altitude ornamentals but also aids in the conservation of fragile mountaintop ecosystems facing the challenges of global climate acceleration.

We have publicly disseminated our research findings through preprints in Authorea (doi: 10.22541/au.170047045.50747481/v1) to guarantee prompt accessibility for the academic community and to foster additional collaboration and discussion. There are no conflicts of interest, and our study does not involve human subjects. In order to improve the readability, we have asked a professional English language service for help and this manuscript has been edited by a native professional English speaker. The raw sequencing data of this study have been deposited in the Sequence Read Archive (SRA) under Bioproject number PRJNA1040959. The genome assembly and annotation data are available at figshare (https://doi.org/10.6084/m9.figshare.24565225.v1).

Thank you for considering our manuscript. Our study aligns with the journal's scope.

We look forward to the opportunity for this work to be reviewed for publication in Gigascience.

Best regards

Yours truly,

Shi-Kang Shen,

School of Ecology and Environmental Sciences, Yunnan University,

E-mail: ssk168@ynu.edu.cn
