# Supplementary material for: The first high-altitude autotetraploid haplotype-resolved genome assembled (Rhododendron nivale subsp. boreale) provides new insights into mountaintop adaptation
Source: Gigascience. 2024 Aug 7;13:giae052. doi: 10.1093/gigascience/giae052 (PMC11304948; doi:10.1093/gigascience/giae052)
Supplement: giae052_GIGA-D-23-00395_Revision_1 [file giae052_giga-d-23-00395_revision_1.pdf]

GigaScience

The first high-altitude autotetraploid haplotype-resolved genome assembled (Rhododendron nivale subsp. boreale) provides new insights into mountaintop adaptation

--Manuscript Draft--

Manuscript Number:	GIGA-D-23-00395R1
Full Title:	The first high-altitude autotetraploid haplotype-resolved genome assembled (Rhododendron nivale subsp. boreale) provides new insights into mountaintop adaptation
Article Type:	Data Note

Funding Information:

Funder	Recipient
Education Department of Yunnan, Scientific Research Fund Project (2024Y003)	Mr. Zhen-Yu Lyu
Science and Technology Development Fund of Guidance from the Central Government to Locals (202207AB110016)	Prof. Shi-Kang Shen
Major Program for Basic Research Project of Yunnan Province (202101BC070002)	Prof. Shi-Kang Shen
National Natural Science Foundation of China (31870529)	Prof. Shi-Kang Shen
Graduate Scientific Research Fund Project of Yunnan University (KC-22221373)	Mr. Zhen-Yu Lyu

Abstract:

Background

Rhododendron nivale subsp. boreale Philipson et M. N. Philipson is an alpine woody species with ornamental qualities that serve as the predominant species in mountainous scrub habitats found at an altitude of ~4200 m. As a high-altitude woody polyploid, this species plays a distinct role in the adaptability of alpine plants. Despite its ecological significance, the lack of genomic resources has hindered a comprehensive understanding of its evolutionary and adaptive characteristics in high-altitude mountainous environments.

Findings

We sequenced and assembled the genome of R. nivale subsp. boreale, an assembly of the first subgenus Rhododendron and the first high-altitude woody flowering tetraploid, contributing an important genomic resource for alpine woody flora. The assembly included 52 pseudochromosomes (scaffold N50=42.93 Mb; BUSCO=98.8%; QV=45.51; S-AQI=98.69), which belonged to 4 haplotypes, harboring 127,810 predicted protein-coding genes. Conjoint k-mer analysis, collinearity assessment, and phylogenetic investigation corroborated autotetraploid identity. Comparative genomic analysis revealed that R. nivale subsp. boreale originated as a neopolyploid of R. nivale and underwent two rounds of ancient polyploidy events. Transcriptional expression analysis showed that differences in expression between alleles were common and randomly distributed in the genome. We identified extended gene families and signatures of positive selection that are involved not only in adaptation to the mountaintop ecosystem (response to stress and developmental regulation) but also in autotetraploid reproduction (meiotic stabilization). Additionally, the expression levels of the ERF VIIs were significantly higher than the mean global gene expression. We suspect that these changes have enabled the success of this species at high altitudes.

Conclusions

We assembled the first high-altitude autopolyploid genome and achieved chromosome-level assembly within the subgenus Rhododendron. In addition, a high-altitude adaptation strategy of R. nivale subsp. boreale was reasonbly speculated. This study provides valuable data for the exploration of alpine mountaintop adaptations and the correlation between extreme environments and species polyploidization.

Corresponding Author: Shi-Kang Shen
Yunnan University

Powered by Editorial Manager® and ProduXion Manager® from Aries Systems Corporation

	Kunming, CHINA
**Corresponding Author Secondary Information:**	
**Corresponding Author's Institution:**	Yunnan University
**Corresponding Author's Secondary Institution:**	
**First Author:**	Zhen-Yu Lyu
**First Author Secondary Information:**	
**Order of Authors:**	Zhen-Yu Lyu
	Xiong-Li Zhou
	Si-Qi Wang
	Gao-Ming Yang
	Wen-Guang Sun
	Jie-Yu Zhang
	Rui Zhang
	Shi-Kang Shen
**Order of Authors Secondary Information:**	
**Response to Reviewers:**	Dear Editor and Reviewers,<br>Thank you for your email and the opportunity to revise our manuscript, “The first high-altitude autotetraploid haplotype-resolved genome assembled (Rhododendron nivale subsp. boreale) provides new insights into mountaintop adaptation” (GIGA-D-23-00395).<br>These comments are valuable and helpful for revising and improving the quality of our manuscript, as well as providing important guidance for our future research. We have checked the manuscript and have tried our best to revise it carefully according to these comments. The main changes are as follows:<br>(1) We have entrusted our paper to a professional institution for English language polishing to improve the language and logic of the manuscript.<br>(2) We have emphatically adjusted the tone to show that the alpine adaptation strategy in the manuscript is speculative and needs to be verified by further biological experiments.<br>(3) We found that a small portion of positive selection genes was recorded incorrectly, and we are very sorry about that. To identify the positive selection signal more accurately, we have updated the detection approach. In the revision, a multi-model cross-validation strategy was used to detect positive selection genes. Although the results produced by this approach are slightly different, the overall conclusion has not changed.<br>(4) We have also changed the submission section to DATA NOTE to emphasize that our main contribution is the provision of an important alpine plant genetic resource.<br>We have marked the changes with different colors in the revised manuscript to facilitate your review of the changes (Blue: Modifications based on review comments; Green: English grammar, logic and other modifications). We earnestly appreciate the thorough work of the Editors/Reviewers, and sincerely hope that you find our responses and modifications satisfactory. Thank you again for considering our work. We hope that this version of our manuscript is suitable for publication in GigaScience.<br><br>Best regards<br>Sincerely yours,<br>Shi-Kang Shen<br><br>Reviewer #1:<br>Thank you for your thorough evaluation of our manuscript and for the constructive comments provided. We appreciate the time you’ve taken to review our work. We acknowledge the issues you have raised regarding the clarity of language and the logical flow of the manuscript. We understand that these are crucial for effectively

communicating our research and ensuring that the scientific community can fully assess the quality and rigor of our work. To address your concerns, we made a complete revision of the manuscript:

Major Concern:
Comment: The paper is not yet ready for publication and requires a lot of writing improvement work to clarify the analyses and results.
Response: Thank you for your comment. For editors and reviewers to effectively evaluate our research, we conducted a careful examination of the manuscript, corrected typos, and improved the logic of the full text. In addition, we entrusted the manuscript to a professional agency to improve its readability.

Comment: I find that many of the claims that the authors suggest (especially concerning gene family expansion and contraction) are strongly worded and at the very least need to be toned down since they are only correlative at best.
Response: Thank you for your comment. We also realize that our study is preliminary and only speculates on high-altitude adaptation strategies. Therefore, we moderate our tone and show in the paper that our studies on adaptive strategies are speculative. In addition, we change the study to “Data Note”, showing that our study mainly provides an important genetic resource of high-altitude woody polyploid. We appreciate your attention to detail and guidance in enhancing the manuscript’s quality.

Other Concern:
Comment: Line 28 - the context and importance of R. nivale as the "first high-altitude woody flowering autotetraploid." Is missing. Why should the reader care about this very specific case study. I think some better lead up will help understand.
Response: Thank you for your comment. We have added a leading-in to the background of the abstract to make it easier for readers to quickly understand the issues of concern in this article. “As a high-altitude woody polyploid, this species occupies a distinctive role in the exploration of alpine plant adaptability.” (Line 24-25)

Comment: 30 - "harbor" change to "which harbor" or "harboring"
Response: Thank you for pointing out the mistake. We have revised the sentence to include “harboring” to properly connect the relative clause, providing a more precise and readable statement. (Line 33)

Comment: 37 - ERF VII are undefined yet as a gene class.
Response: Thank you for your comment. Group VII ERFs is a phylogenetic cluster, which is conserved across angiosperms. We acknowledge that the ERF VII gene class has not been explicitly defined in the current. However, ERF VII is a widely used gene group (Abbas et al. 2022; Fan et al. 2023; Zubrycka et al. 2023). Our research is predicated on the homology with the well-studied and defined ERF VII gene family in Arabidopsis thaliana. The proposed ERF VIIs is based on sequence similarity and functional characteristics of these known genes, particularly their role in response to hypoxic conditions.
Reference:
Abbas M, Sharma G, Dambire C, et al. An oxygen-sensing mechanism for angiosperm adaptation to altitude. Nature. 2022;606:565–9.
Fan B, Liao K, Wang LN, et al. Calcium-dependent activation of CPK12 facilitates its cytoplasm-to-nucleus translocation to potentiate plant hypoxia sensing by phosphorylating ERF-VII transcription factors. Mol Plant. 2023;16:979–98.
Zubrycka A, Dambire C, Carbonare DL, et al. ERFVII action and modulation through oxygen-sensing in Arabidopsis thaliana. Nat Commun. 2023;14:4665.

Comment: 38 - meanwhile is not the best connector word here and elsewhere.
Response: Thank you for pointing out the problem of improper use of connective words. To improve the readability of the full text, we have checked the connectives in the full text and modified them accordingly according to the context.

Comment: Line 50- what do you mean by core genus. Provide a better definition.
Response: Thank you for pointing out the problem about definition. In the reworked manuscript, we have replaced "core genus" with "largest genus". (Line 55)

Comment: Line 58-62- (1) what do we know about high-altitude polyploids in general?

	(2) Is there any evidence for adaptive benefits? (3) Why should we care?<br>Response: Thank you for your comment. All information related to polyploids is presented in the third paragraph of the introduction. (1) Following polyploidy events, rapid shifts in gene expression and epigenetic modifications can bestow the polyploid with an almost instant competitive edge. (2) Although the evidence of polyploid high altitude adaptation is not completely sufficient, many studies have proved that polyploid plants have stronger abiotic stress tolerance than diploid plants. Therefore, polyploidy tends to be ecologically advantageous and occurs in variable climatic regions, such as the Qinghai-Tibet Plateau alpine and Pan-Arctic regions. (3) Comprehending these adaptive mechanisms in high-altitude polyploids not only illustrates evolutionary dynamics but also informs conservation methodologies. (Line 86-100)<br><br>Comment: Line 78-79 : Limited is repeated twice<br>Response: Thank you for your comment. We have modified the sentence to solve the problem of word redundancy. “Currently, the assembly of autopolyploid genomes presents significant challenges, resulting in the publication of only a select number of such genomes, including those of Medicago sativa, Saccharum spontaneum, Solanum tuberosum, and Rheum officinale.” (Line 81-84)<br><br>Comment: 85 - polyploidy "event"<br>Response: Thank you for your carful checks. We have modified in manuscript. (Line 88)<br><br>Comment: 113- poor grammar and structure "Tetraploid was identified from the kmers present in sequencing reads (Fig. 1E)."<br>Response: Thank you for pointing out the problem. We have modified this sentence to "This species identified as a tetraploid based on the k-mers analysis." (Line 117-118)<br><br>Comment: 116 - great that you tried different assembly approaches. I think you should report some contig level statistics to show why you chose hifiasm. Minimum should report contig N50.<br>Response: Thank you for your recognition of our analytical methods. The results for each assembly approach are shown in Table S4.<br><br>Comment: 137 - LAI of reference suggests that the repeat content of the assembly is not well assembled. This can be improved by using HiFi pacbio reads. Although I understand that may not be in the scope of this manuscript however that would be an improvement for the assembly.<br>Response: Thank you for your suggestion to improve the assembly using HiFi PacBio reads. We agree that this approach could potentially enhance the assembly quality However, due to the extensive time and resources required for such an analysis, which includes not only closing gaps but also a complete re-run of all subsequent analyses, we are unable to include this in the current scope of our manuscript. To assess LAI more accurately, we have evaluated each haplotype independently, thereby reducing the negative impact of highly similar sequences between haplotypes on LAI regulation. The results showed that LAI of all haplotypes was greater than 14 (n1:14.78, n2:14.84, n3:14.35, n4:14.38). (Line 140-142) We have noted your recommendation as a valuable suggestion for future improvements to our assembly and will consider it for subsequent iterations of our work.<br><br>Comment: 159-163 the whole section about confirming the polyploidy using kmers should be explained better.<br>Response: Thank you for your comment. We have shown in the methods section how to determine auto and allotetraploid based kmers analysis. (Line 549-550). For more specific principles, please refer to the citation “[29] Ranallo-Benavidez TR, Jaron KS, Schatz MC. GenomeScope 2.0 and Smudgeplot for reference-free profiling of polyploid genomes. Nat Commun. 2020;11:1432.”<br><br>Comment: 167 - I am not familiar with the term "exerted synteny" I don't think it is being used the correct way.<br>Response: Thank you for your comment. Upon check, we agree that the term ‘exerted’ may not be the most appropriate in this context. We have revised the manuscript accordingly. The text now reads: “As expected, the dot plot and syntenic blocks indicated synteny among the four haplotypes (Fig. 2A), with 20,172 gene pairs showing

synteny between haplotypes 1 and 2, 20,249 between haplotypes 2 and 3, and 19,883 between haplotypes 3 and 4." (Line 169-172)

Comment: 169 - clarify what transcriptome data are being used here.
Response: Thank you for your comment. We provided relevant information of all the data in the manuscript in the Table S14, including species, data type, accession number and download address. The data generated in this study (n1, n2, n3, n4, and R. nivale subsp. boreale represent the four haplotypes and transcriptome of R. nivale subsp. boreale, respectively), are shown in Figure 2 title. (Line 969-971)

Comment: 214- incomplete sentence "In the dot plot between Vi. vinifera and R. nivale subsp. boreale (Fig. 3D, S7-8)."
Response: Thank you for pointing out the incomplete sentence. The sentence should be completed as follows: "The dot plot comparing Vi. vinifera and R. nivale subsp. boreale (Fig. 3D, S7–8), nearly every grape chromosome exhibited two highly compatible chromosomal regions in R. nivale subsp. boreale (orthologous ratio 1:2)." (Line 215-217).

Comment: 217 - the whole section on positive selection is not written well and was challenging to understand. Thus, the claims derived from these analyses are not well supported at this stage. There is no introduction to the section just an immediate dive into 44 genes. The reader is not prepared for that yet.
Response: Thank you for your valuable feedback regarding the section on positive selection. We apologize for any confusion caused by our initial presentation of the data and analysis. We acknowledge that the high-altitude adaptation mechanisms of R. nivale subsp. boreale cannot be fully resolved based on current data. In this manuscript, our aim is to provide important high-altitude plant genomes and speculate the high-altitude adaptation strategies of Rhododendron based on existing data, providing a scientific basis for future adaptation studies. To detect the true positive selection events as much as possible, we have updated the positive selection gene recognition pathway. On the basis of aBSREL detection, the Clade Model was used to exclude genes that may not be affected by selection pressure. Finally, the positive selection sites were identified based on the intersection of the MEME and the Contrast-FEL. A gene is considered a positive selection gene when it meets all model criteria. The multi-method detection approach reduces the probability of false positive gene identification. Finally, the results of the positive selection analysis from KaKs_Calculator are integrated to collectively speculate on the adaptation mechanism. Additionally, we have revised this section to improve clarity and ensure that the narrative is more accessible. I appreciate the opportunity to enhance the quality and rigor of our manuscript. (Line 219-234; 581-604; Tables S18-S20)

Comment: 230 - 233 - can be moved to discussion
Response: Thank you for your suggestion. We very much agree with this adjustment and have moved this section to the discussion section. (Line 369-374)

Comment: 282 - "More species had approximately 140 genes." Unclear sentence and context
Response: Thank you for comment. The intent of this statement was to convey that a greater number of species were found to have the gene count of AP2/ERF close to 140 in our study. To clarify this, we have revised the sentence to read: "The majority of species in our study had gene counts of approximately 140." (Line 283-284)

Comment: 291-295 - the whole section is missing citations and could be moved to the discussion.
Response: Thank you for your constructive feedback. We have added the appropriate references to ensure the arguments are well-founded. Additionally, we agree that the content may be better suited to the discussion part of the paper and have made the necessary adjustments to relocate the section. (Line 435-437)

Comment: 303 - citations.
Response: Thank you for your careful check. We have added the appropriate citation. The following revisions have been made to the manuscript: "Group VII ethylene response factor transcription factors (ERF VIIs) are associated with altitude adaptation [34]." (Line 291-292)

Powered by Editorial Manager® and ProduXion Manager® from Aries Systems Corporation

Comment: 307-310 conclusion is very strong for the evidence presented.
Response: Thank you for your comment. We understand your concern regarding the strength of the conclusion drawn from the observed gene expression. We would like to clarify that the high expression levels of these genes of R. nivale subsp. boreale are consistent with the plant’s adaptation to its natural habitat, which is characterized by low oxygen pressure. This inference is supported by the literature indicating that ERF VII genes probably are involved in the response to hypoxic conditions. However, we acknowledge that further experimental validation is needed to definitively establish the causal relationship. Therefore, we have moved it to discussion section and softened the language and said it was subject to experimental verification. We have revised the manuscript as follows: “The high expression levels of ERF VIIs in R. nivale subsp. boreale suggests a potential adaptation to low oxygen environments, which warrants further experimental investigation.” (Line 441-443)

Comment: 324 - typo : badiratE
Response: Thank you for pointing out the typo. We have corrected it. (Line 316)

Comment: 332- the distribution of the CYPs is shown? Reference a figure.
Response: Thank you for your comment. We have added the appropriate figures (S10-21), which visually represent the distribution of the AP2/ERFs, CYPs, ERF VIIs and CBFs in R. nivale subsp. boreale.

Comment: 336 - missing citations.
Response: Thank you for pointing out the missing citation. We have added the appropriate citation. (Line 330)

Comment: Overall the claims about positive selection of genes for altitude fitness should be toned down. The functional validation of the suggested genes is not clear and while the analysis might show they are under selection the role and fitness advantage is not supported by evidence presented herein.
Response: Thank you for your comment on genes for altitude fitness. We acknowledge that our results are preliminary and appreciate your suggestion to moderate the language used to describe these findings. To enhance the accuracy of analysis, we have modified our strategy of positive selection analysis to include multiple models for cross-validation, namely two branch site-based models (aBSREL and Clade Model) and two site-based models (MEME and Contrast-FEL). Additionally, we have revised the tone of the conclusion to reflect that it is a conjecture based on existing results, pending further experimental validation. Our aim is to provide valuable genetic resources and to formulate initial hypotheses regarding the high-altitude adaptation mechanisms in R. nivale subsp. boreale. In our latest revision, we have reclassified the submission as a DATA NOTE to emphasize that the primary contribution of our research is the provision of significant high-altitude genetic resources.

Comment: 431- what is anti-low temperature
Response: Thank you for your comment. We have modified it to “cold tolerance”. (Line 444-445)

Comment: 444- CFBs are lowly expressed? What are the conditions of sampling? These are cold response genes.
Response: Thank you for bringing this to our attention. We recognize that the expression of CBFs is typically induced by cold conditions, and therefore, their low expression under the conditions sampled may not fully reflect their role in cold response. Therefore, we have deleted this section.

Comment: 510 - what is this analysis for Modifit?
Response: We are sorry for our careless mistakes. Thank you for your reminder. The correct name for the software is ModFit LT 5.0, and its download address has been added to the manuscript. (Line 511) ModFit LT is the open-ended modeling software for cytometry data. We use ModFit LT to process flow cytometry results (Fig. S1). (Line 506-507)

Comment: 538 - Clarify the steps you took to annotate. which transcripts were used where and for what software downstream.

Powered by Editorial Manager® and ProduXion Manager® from Aries Systems Corporation

Response: Thank you for your comment. We have rewritten the section based on transcriptome annotations. During our annotation, the transcriptome data were assembled using Trinity and StringTie. Next, all assembly results were fed into BRAKER3 and PASA for gene prediction and to generate Ab initio gene predictor training sets. We have made the subsequent changes to the manuscript: “In our transcriptome-based strategies, we used HISAT2 v2.2.1 [92] to align clean reads of the transcriptome with the genome. Trinity v2.14.0 [93] and StringTie v2.2.1 [94] were used to assemble transcripts. BRAKER3 [95] and PASA v2.5.2 [96] were used to predict gene structure based on the assembled transcripts and to generate ab initio gene predictor training sets.” (Line 534-538)

Comment: 553 - You said you used smudgeplot at the top of the section not genomescope.
Response: Thank you for your comment. Genomescope2 and Smudgeplot are published in the same article, but they are completely different pipeline. Genomescope2 was used to estimate genome size and heterozygosity, and Smudgeplot was used to estimate genome ploidy. We used Genomescope2 to obtain an estimate of genomic heterozygosity (Table S3) and Smudgeplot to evaluate ploidy (Fig. 1E).

Comment: 556- JCVI has a better citation than the zenodo repo
Response: Thank you for your recommendation to provide a better citation for JCVI. We have updated the citation to reflect the primary literature associated with the JCVI utility libraries. The revised citation is as follows: “103. Tang H, Bowers JE, Wang X, Ming R, Alam M, Paterson AH. 2008. Synteny and collinearity in plant genomes. Science 320:486–488.”

Comment: 615- BadiTate typo
Response: Thank you for pointing out the typo. We have corrected it. (Line 631)

Comment: 629 - clarify the author contributions what does "Investigation" mean.
Response: Thank you for your comment. In this study, in addition to bioinformatics analysis, cytological experiments were used to estimate genome size (Fig. S1) and chromosome number. Gao-Ming Yang, Jie-Yu Zhang and Wen-Guang Sun provided experimental sites, reagents, and conducted experiments. Unfortunately, R. nivale subsp. boreale has a lot of small chromosomes, and despite numerous experiments, we have not yet obtained the desired results. We have modified “Investigation” to “Cytological experiment.” (Line 642-644)

Reviewer #2:
Thank you for your recognition of our work. Your suggestions are very important to us, both for improving the manuscript and our further research. We have studied these comments carefully and have made corrections as possible as we can. We hope these corrections could meet with approval. The point-by-point response is as follows:

Comment: Scientific names: Arabidopsis has been named as "Ar. thaliana" throughout the paper should be "A. thaliana".
Response: Thank you for your suggestion. We used Ar. thaliana to distinguish Ac. chinensis. Using the same abbreviation is likely to cause confusion.

Comment: Line 141: The completeness of protein coding genes were accessed by BUSCO. This is a good metric to access completeness, however sometimes BUSCO does not give us the complete picture. Hence I suggest, adding mono-exonic (single-exon) to multi- exonic (multiple-exon) ratio in the needed for further justification and support.
Response: Thank you for your valuable feedback. To address this, we calculated the ratio (0.245) of mono-exonic (single-exon) to multi- exonic (multiple-exon) genes and have added this information to the manuscript (Line 148-149). Additionally, we have provided more details about BUSCO in Table S8. This table contains BUSCO assessment results for different assembly approaches, different haplotypes, unitigs and protein-coding genes.

Comment: Line 289: The authors mention variable alpine climate for growth but do not

provide any metric data related to the statement. Growing conditions with metrics like average temp difference, oxygen level and average pressure needs to be provided for better context.
Response: Thank you for your valuable feedback. We have added relevant metrics for alpine climate, including average annual temperature, air pressure and diurnal temperature differences, which are presented in the discussion section. The revised text is as follows: “Low temperature (average annual temperature below 0 °C), low partial pressure of oxygen (for every 1,000 m increase in altitude, air pressure drops by about 11%) and rapid weather changes (annual average diurnal temperature exceeding 20 °C) are the main factors limiting alpine plant survival.” (Line 422-425)

Comment: Line 303: "Previous studies have shown that ERF VIIs play an extremely important role in adapting to high-altitude environments" This statement needs citation for support.
Response: Thank you for your carefully checks. The following revisions have been made to the manuscript: “Group VII ethylene response factor transcription factors (ERF VIIs) are associated with altitude adaptation [34].” (Line 291-292)

Comment: In the discussion section author has discusses in great detail about different pathways regulating different morphological traits. But a section describing the morphological traits and the phenotypic adaptation of Rhododendron nivale subsp. boreale is necessary to better relate these pathways in the contest of this species being studied.
Response: Thank you for your insightful suggestion. We agree that a section detailing the morphological traits and phenotypic adaptations of R. nivale subsp. boreale would greatly enhance the discussion. Consequently, we have speculated potential altitudinal adaptation traits in R. nivale subsp. boreale from existing studies and have incorporated this information into the manuscript. These characteristics include plant height, leaf size, flowering changes, and seed size. We have updated the manuscript as indicated below: “In alpine environments, plants have evolved myriad morphological and physiological adaptations to contend with the rigor of high-altitude conditions [50]. R. nivale subsp. boreale native to mountaintops, typically reaches heights of less than 30 cm, with leaves that seldom exceed 5 mm in both length and width, exhibits delayed flowering, and produces seeds that are nearly indiscernible. These characteristics are thought to be a response of Rhododendron to low temperatures at high altitudes, poor nutrition, and extremely short growth cycles [51].” (Line 408-413)

Comment: Line 444: Why CBFs and its transcription factors are relatively low expressed or absent? What does the author thinks is the reason? Further justification maybe necessary.
Response: Thank you for your comment. We are very sorry for ignoring CBFs as a class of cold response genes. The expression level of CBFs was closely related to our sampling conditions. It is possible that the gene expression observed may be influenced by various factors, such as the ambient temperature during sampling and the gene-dosage effect. Given these considerations, we cannot make reasonable speculation, and therefore, we have removed this section from the discussion.

Reviewer #3:
We would like to thank you for your professional review work, constructive comments, and valuable suggestions. Your insights have been instrumental in enhancing the quality of our research, and we have carefully considered each point to ensure our work meets high standards. We have revised our manuscript to clarify our claims and enhance the overall clarity of our work. We are confident that the revisions made will address your concerns. Followed by a point-by-point response:

Major Concern:
Comment: The authors logically and comprehensively describe their evidence to suspect these genes and pathways with relevant literature cited throughout and I applaud them for that. However, the evidence is not strong enough to support these final claims. They should be worded along the lines of, "we suspect these loci enabled the success of this species in high altitudes", or something of the sort rather than claiming they aid in survival or adaptability without having explicitly tested that fact.
Response: Thank you for acknowledging the logical structure and analytical comprehensiveness of our evidence presentation. We appreciate your suggestion to

Powered by Editorial Manager® and ProduXion Manager® from Aries Systems Corporation

moderate the language used to describe the influence of the identified loci on the success of the species at high altitudes. We agree that without explicit testing, it is more appropriate to use tentative language. Therefore, we have revised our claims to reflect that these loci are suspected of contributing to the species' success in high-altitude environments.

Other Concern:
Comment: Line 37: "Notably, highly expressed ERF VIIs aid survival in hypoxic mountaintop. Meanwhile, the extended families enriched in brassinosteroid biosynthesis, which enhanced adaptability to alpine weather and probably mediated by increased cytochrome P450 genes."
The authors present no experimental evidence for this claim. They did not explicitly test survival under hypoxic conditions, nor any physiological response to alpine weather.
Response: Thank you for your comment. We agree that all of these assumptions need to be tested experimentally. Therefore, we have revised the relevant sections to clarify that these are hypotheses based on gene expression patterns and known functions of these genes and gene families. The revised text now reads: "We identified extended gene families and signatures of positive selection that are involved not only in adaptation to the mountaintop ecosystem (response to stress and developmental regulation) but also in autotetraploid reproduction (meiotic stabilization). Additionally, the expression levels of the ERF VIIs were significantly higher than the mean global gene expression. We suspect that these changes have enabled the success of this species at high altitudes." (Line 38-43)

Comment: Line 100: "and determined which genes or gene families play a role in alpine adaptation and the survival of polyploids"
Qualifying language required that you have well supported candidates but do not know if they directly play a role in adaptation or survival without testing for these phenotypes.
Response: Thank you for your comment. We acknowledge the necessity of qualifying our language regarding the role of specific genes or gene families in alpine adaptation and the survival of polyploids. We have revised to reflect that, while we have identified well-supported candidate genes, their direct involvement in adaptation or survival remains to be empirically tested. The revised statement now reads: "Based on this assembly, we identified polyploid types, deciphered whole genome duplication (WGD) events, and investigated which genes or gene families are potential candidates involved in alpine mountaintop adaptation and the survival of polyploids." (Line 103-106)

Comment: Line 114: "The three initial assembly sizes were 2.48 Gb, 2.39 Gb and 2.40 Gb, which were assembled by Hifiasm, Canu v1.9 and Hicanu, respectively (Table S4). The assembled version of Hifiasm was used for subsequent analysis because it has higher integrity in genes and LTRs.
Thank you for providing some negative results! This helps benchmark software performance and gives the field some insight to how different assemblers handle autopolyploids.
Response: Thank you for your positive feedback. We are glad to contribute to this knowledge base.

Comment: Line 173: "A clade containing all four haplotypes of R. nivale subsp. boreale and two R. nivale was supported by 100% bootstrapping (Fig. 2 C)"
The placement of a 5th individual labeled "R. nivale subsp. boreale" was never explicitly mentioned. I assume this is all four haplotypes combined, but this needs mentioned
Response: We sincerely thank you for careful reading. The "R. nivale subsp. boreale" represents the transcriptome data generated in this study. And we have clarified in the figure title of Figure 2. (Line 969-971)

Comment: Line 175: "Therefore, four haploid chromosomes were considered to originate from the same species"
This claim is not supported by the evidence presented.
The observation of all four haplotypes clustering with one sample of R. nivale and not the other can arise from a few scenarios besides a single species origin. There could exist population structure within R. nivale from which multiple species have derived from one sub population and not the others.

Now is as good of a time as any but I do not believe in the technical distinction between auto and allo polyploids and especially not whether the progenitors belong to the same species being the delineation. I think differences between auto and allo are quantitative and the authors have done an excellent job layering analyses to address this question.
As it pertains to this claim of this genome deriving from a single species, the phylogenetic tree in Figure 2C is insufficient to claim this as a fact.
I recommend removing this statement.
Response: Thank you for recognizing our analysis of polyploid types. We completely agree with you. The difference between autopolyploids and allopolyploids should be quantitative. Otherwise, the type of polyploid hybrids in closely related species cannot be determined. Therefore, we have removed that statement.

Comment: Line 185: "1 basal angiosperm"
Though commonly referred to as a "basal" species, an extant species cannot be "basal". All extant species have been evolving for the same amount of time and contain their own derived characteristics. I recommend simply referring to this as "Amborella", or "1 species sister to all angiosperms" to keep the syntax consistent in that sentence.
Response: Thank you for your valuable feedback. We have revised to read “1 species sister to all other angiosperms (Amborella),” which accurately reflects the phylogenetic position of Amborella. (Line 188)

Comment: Line 191: "These species-specific genes were enriched in 12 KEGG pathways and 136 GO terms, such as arginine biosynthesis, nitrogen metabolism and flavonoid biosynthesis"
I cannot find where in the methods your GO enrichments are listed, but I did see not all genes were functionally annotated with a GO term. The background set to test these 369 R. nivale subsp. boreale specific genes should be all genes with a GO annotation rather than all genes in total. This is simply to test the robustness of your results which I do not expect to change, however reducing the background set is the more statistically appropriate test.
Response: Thank you for your meticulous review and valuable suggestion regarding the GO enrichment analysis. We used clusterProfiler for enrichment analysis. The background gene set used in the GO enrichment analysis was derived from the annotation result file (org.My.eg.db_1.0.tar.gz) produced in the annotation. This file contains all the genes that have been annotated by GO, and we have not made any changes. We present the analysis process as follows:

```
library(tidyverse)
library(clusterProfiler)

#KEGG
gene <- read.csv("~/enrichment /uni_gene.xls", sep="")
ee <-as.matrix(gene)
genev <-as.vector(ee)
gene2passway <- read.csv("~/enrichment / kegg_gene2passway.csv")
pathway2name <- read.delim("~/enrichment /kegg.pathway2name.tsv")
de_ekp <- enricher(genev, TERM2GENE = gene2passway, TERM2NAME =
pathway2name, pvalueCutoff = 0.05, qvalueCutoff = 0.05)
de_ekp_df <- as.data.frame(de_ekp)

#GO
dir.create('R_Library', recursive = T)
install.packages('~/enrichment/org.My.eg.db_1.0.tar.gz', repos = NULL, lib =
'R_Library')
library(org.My.eg.db, lib = 'R_Library')
de_ego <- enrichGO(gene = genev, OrgDb = org.My.eg.db, keyType = 'GID', ont =
'ALL', qvalueCutoff = 0.05, pvalueCutoff = 0.05)
de_ego_df <- as.data.frame(de_ego)
```

Comment: Line 211: "In addition, the recent peak at Ks ~0.65 indicated that another polyploidy of Rhododendron, Va. darrowii and Ac. chinensis occurred ~78 Mya (Fig. 3C, S9)."
It was unclear to me both in the text and in the figure legend for Fig 3C whether the Ks

distributions were of orthologs to R. nivale subsp. boreale or of paralogs within each genome. I can infer after reading some of the conclusions that these are intragenomic paralog Ks distributions but this needs made more clear in both the main text and in the figure legend.
Response: Thank you for your comment. Your inference is correct. We are very sorry for our unclear statement. We have clarified these in the text and in the figure title. The following revisions have been made to the manuscript: “In addition, the peak at Ks of the paralogs approximately 0.65 Mya suggests another polyploidization event in Rhododendron, Va. darrowii, and Ac. chinensis estimated to have occurred at approximately 78 Mya.” (Line 213-214; Line 980; Fig S9 title)

Comment: Line 215: "We identified nearly every grape chromosome with two highly compatible chromosome regions in R. nivale subsp. boreale (orthologous ratio 1:2) (Fig. 3D)"
For Fig 3D, the axis labels are ambiguous. Also I assume to dot plot is between genes and not chromosomes. I may have missed where this was stated but if its absent if must be included.
Response: Thank you for your comment. Dot plot analysis is based on homologous genes, and this statement is shown in the figure title of Fig 3. As follows: “D. Homologous gene dot plots between R. nivale subsp. boreale and Vi. vinifera. The red box exemplifies the orthologous ratio of 1:2 between Vi. vinifera and R. nivale subsp. boreale.” (Line 983-985)

Comment: Line 218: "A total of 44 genes were positively selected and functionally annotated based on Hyphy and kaks_calculator (Table S18; S19)"
I appreciate the inclusion of these results in the supplement but I recommend inclusion of quantitative values that indicated these genes were positively selected. I do not have experience with Hyphy so I'm not sure this is possible, but for kaks_calculator I think you can provide the Ka/Ks value for quantitative comparisons of these positively selected genes.
Response: Thank you for your suggestion to include more information for positively selected genes. We recognize that the lack of experimental validation is our key shortcoming. In the latest version of the manuscript, we have improved our analytical approach to detect positive selection events that are as real as possible. This approach eliminates possible false positive signals through cross-validation using multiple models. We used Hyphy for positive selection genetic testing based on an exploratory model. Next, we used the Clade Model to exclude genes not affected by selection pressure. Finally, we used MEME and Contrast-FEL to detect whether these potentially selected genes contain positive selection sites. Only when a gene meets all the model requirements do we consider it to be a valid positive selection gene. In addition, we have added the detailed results and methods of the selection analysis. We believe that the updated results are useful for better inferences of R. nivale subsp. boreale adaptation strategies to high altitude environments. (Line 219-234; Line 581-600; Tables S18-S20)

Comment: Line 233: "However, our results do not support this."
I think a statistical test is required to say whether the Ka/Ks distributions are not different within each homologous group for Fig S6. Otherwise the authors could rephrase this to "The distribution of Ka/Ks values do not appear different between our homologous groups (Fig S6)"
Response: Thank you for your suggestion. We have considered your recommendation to rephrase our statement, and have adjusted the text to “However, the distribution of Ka/Ks values did not differ between our homologous groups.” In addition, we moved this statement to the discussion. (Line 369-374)

Comment: Line 263: "the transcript expression of homologous Group 3 was higher than that of the other groups (Fig. 5A)"
These values need normalized by the number of genes on each chromosome or by the total length of CDS as this pattern may simply reflect the number of genes.
Also it would be interesting to investigate this pattern across tissue types.
Response: Thank you for your comment. We agree that normalizing the transcript expression levels by the number of genes on each chromosome would provide a more accurate reflection of gene expression patterns. We have now included normalization based on the number of genes in our analysis and have updated Figure 5A

accordingly. Additionally, we recognize the value in investigating expression patterns across different tissue types. While this was beyond the scope of our current study, we have noted it as a potential direction for future research.

Comment: Line 267: "Finally, 3,844 of the 6,388 (60.17%) single-match gene groups were identified as differential expression loci (DELs)"
(1) How were DEL determined?
(2) Were there biological replicates within each tissue?
(3) I also do not understand what conclusions are drawn from Fig 5B.
Response: We are very sorry that our unclear expression has caused your misunderstanding. (1) The identification strategy for DELs is shown in the method (Line 611-613). The method was published in the autotetraploid sugarcane genome study. Specifically, any two genes in a set of single-match genes with less than a two-fold difference in expression are defined as neutral; the other alleles are considered DELs. (2) Although our transcriptome comes from three replicates, samples are derived from a mixture of roots, stems, leaves, and buds. Expression levels in different tissues could not be analyzed. (3) As for Figure 5B, it illustrates the distribution of DELs across different homologous groups. As you mentioned in your comment, it is hard to tell the true haplotype origin of each chromosome. Thus, we used an independent analysis of each chromosome to compare differences in transcriptional expression level. In Figure 5B, we added labels for each heat map to indicate that our analysis was based on the chromosome level rather than haplotype. Each row of each heat map represents a set of single-match genes, while each column represents a chromosome. For example, in the heat map of Homologous group 1, 530 rows represent 530 sets of DEL alleles, and the colors represent differences in expression levels. The red and blue squares are scattered across different chromosomes within the homologous group, representing the distribution pattern of DELs.

Comment: Figure 6C:
What is the distribution of the "All" category when you removed genes that are not expressed. That should be a more appropriate comparison.
Response: Thank you for your advice. We have further clarified in figure 6 and the figure title. To address this, we have modified “All” to “Global Expression” to clearly show that the Figure 6C is drawn based on the expressed gene (Fig. 6C).

Comment: I was unable to access "PRJNA1040959" which is understandable as it should be private before publication but this led me to be unable to verify the availability of the data or if there were biological replicates for their expression analyses.
Response: Thank you for your comment. We provided a data access link from which the original data can be accessed (https://dataview.ncbi.nlm.nih.gov/object/PRJNA1040959?reviewer=46vvm122fdnc0s72kb0guu85fl). To obtain comprehensive transcriptional information, we collected and mixed samples from various tissues, ensuring three biological replicates.

Comment: Every phylogenetic tree in the main text and supplementary figures need explanations of how the trees were constructed including the program and methods (for example, maximum likelihood or parsimony?"
Response: Thank you for your comment. We have provided the construction methods of all the phylogenetic trees in the manuscript, including the figure title and the materials and methods section. These phylogenetic trees are constructed based on ML, and all are inferred using IQtree2 except for executing two large trees with thousands of sequences using Fasttree.

Comment: Some of conclusions of autopolyploid origin look at genome-wide patterns. I think some of them require a chromosome by chromosome approach like the expression analysis. Though the genome is phased, it is not phased across chromosomes, you do not know if homologous group 1 n1 is from the same parent as homologous group 5 n1.
Response: We could not agree with you more. We also expect to explore the origin of autopolyploids using a chromosome-by-chromosome approach. However, the lack of chromosome-level genomes in closely related species limits this approach. As the number of genetic resources increases, it will become more convenient and accurate to identify the origin of haplotypes in polyploids. For the level of gene expression, we

Powered by Editorial Manager® and ProduXion Manager® from Aries Systems Corporation

	have used a chromosome-by-chromosome process for analysis, such as allelic expression difference analysis (Fig. 5B).<br><br>Comment: Group 3 CBF are not monophyletic Fig S12, does this change any of your findings / is it biologically interesting?<br>Response: We are very sorry for our carelessness. Group III and Group IV should be combined. We have made changes accordingly (Fig. S24). Group III seem to have been split into two clades in the Ericaceae. Our modifications are as follows: "Phylogenetic analysis indicated that CBFs of Rhododendron were divided into 3 groups (Fig. S24)." (Line 313-314)<br><br>Comment: Line 457: "The presence of multiple alleles indicated that the expression level of R. nivale subsp. boreale is probably higher than that of the diploid ancestor"<br>I do not believe this result is supported by the data presented without being able to test the diploid ancestor. How do you know expression levels have not globally and proportionally reduced to compensate for the increase in copy number?<br>Response: Thank you for your comment. We acknowledge that our statement regarding the expression levels of R. nivale subsp. boreale compared to the diploid ancestor was speculative. Without direct expression data from the diploid ancestor, we cannot conclusively determine if the observed multiple alleles correlate with higher expression levels. Hence, we have removed this statement.
**Additional Information:**	
**Question**	**Response**
Are you submitting this manuscript to a special series or article collection?	No
**Experimental design and statistics**<br><br>Full details of the experimental design and statistical methods used should be given in the Methods section, as detailed in our Minimum Standards Reporting Checklist. Information essential to interpreting the data presented should be made available in the figure legends.<br><br>Have you included all the information requested in your manuscript?	Yes
**Resources**<br><br>A description of all resources used, including antibodies, cell lines, animals and software tools, with enough information to allow them to be uniquely identified, should be included in the Methods section. Authors are strongly encouraged to cite Research Resource Identifiers (RRIDs) for antibodies, model organisms and tools, where possible.	Yes

Have you included the information requested as detailed in our Minimum Standards Reporting Checklist?	
**Availability of data and materials**<br><br>All datasets and code on which the conclusions of the paper rely must be either included in your submission or deposited in publicly available repositories (where available and ethically appropriate), referencing such data using a unique identifier in the references and in the “Availability of Data and Materials” section of your manuscript.<br><br>Have you have met the above requirement as detailed in our Minimum Standards Reporting Checklist?	Yes

Powered by Editorial Manager® and ProduXion Manager® from Aries Systems Corporation

Manuscript

Click here to access/download;Manuscript;manuscript_with_revised_trace.d

Title: The first high-altitude autotetraploid haplotype-resolved genome assembled (*Rhododendron nivale* subsp. *boreale*) provides new insights into mountaintop adaptation

Authors: Zhen-Yu Lyu[1], Xiong-Li Zhou[1], Si-Qi Wang[1], Gao-Ming Yang[1], Wen-Guang Sun[2], Jie-Yu Zhang[2], Rui Zhang[1], Shi-Kang Shen[1*]

Affiliation:

[1]Ministry of Education Key Laboratory for Transboundary Ecosecurity of Southwest China, Yunnan Key Laboratory of Plant Reproductive Adaptation and Evolutionary Ecology, Institute of Biodiversity, School of Ecology and Environmental Science, Yunnan University, Kunming, 650504, Yunnan, China

[2]School of Life Sciences, Yunnan Normal University, Kunming, 650500, Yunnan, China

Corresponding authors: *Shi-Kang Shen, E-mail: ssk168@ynu.edu.cn; (ORCID: 0000-0002-0611-6763)

Telephone: +86-871-65933510; Fax: +86-871-65933510;

Postal address for corresponding authors: School of Ecology and Environmental Sciences, Yunnan University, No.2 Green Lake North road Kunming, Yunnan, 650091, China

Words: 7383 (excluding references)

Figures: 7 color figures.

Abstract

Background

Rhododendron nivale subsp. *boreale* Philipson et M. N. Philipson is an alpine woody species with ornamental qualities that serve as the predominant species in mountainous scrub habitats found at an altitude of ~4200 m. As a high-altitude woody polyploid, this species plays a distinct role in the adaptability of alpine plants. Despite its ecological significance, the lack of genomic resources has hindered a comprehensive understanding of its evolutionary and adaptive characteristics in high-altitude mountainous environments.

Findings

We sequenced and assembled the genome of *R. nivale* subsp. *boreale*, an assembly of the first subgenus *Rhododendron* and the first high-altitude woody flowering tetraploid, contributing an important genomic resource for alpine woody flora. The assembly included 52 pseudochromosomes (scaffold N50=42.93 Mb; BUSCO=98.8%; QV=45.51; S-AQI=98.69), which belonged to 4 haplotypes, harboring 127,810 predicted protein-coding genes. Conjoint *k-mer* analysis, collinearity assessment, and phylogenetic investigation corroborated autotetraploid identity. Comparative genomic analysis revealed that *R. nivale* subsp. *boreale* originated as a neopolyploid of *R. nivale* and underwent two rounds of ancient polyploidy events. Transcriptional expression analysis showed that differences in expression between alleles were common and randomly distributed in the genome. We identified extended gene families and signatures of positive selection that are involved not only in adaptation to the mountaintop ecosystem (response to stress and developmental regulation) but also in autotetraploid reproduction (meiotic stabilization). Additionally, the expression levels of the *ERF VIIs* were significantly higher than the mean global gene expression. We suspect that these

changes have enabled the success of this species at high altitudes.

Conclusions

We assembled the first high-altitude autopolyploid genome and achieved chromosome-level assembly within the subgenus *Rhododendron*. In addition, a high-altitude adaptation strategy of *R. nivale* subsp. *boreale* was reasonbly speculated. This study provides valuable data for the exploration of alpine mountaintop adaptations and the correlation between extreme environments and species polyploidization.

Key words: Autotetraploid, Evolutionary history, Harsh environment, Mountaintop adaptation, *Rhododendron*,

1. Context

Rhododendron L. is the largest genus in Ericaceae and the largest woody plant genus in the Northern Hemisphere, with more than 1,000 species. It is also representative of the highly diverse Sino-Himalayan Flora in East Asia, shaped by the topographic and climatic heterogeneity resulting from the uplift of the Qinghai-Tibet Plateau [1,2]. Furthermore, *Rhododendron* is one of the few woody flowering species that is dominant in plant communities found within the delicate subalpine to alpine transition zone and presents a perfect opportunity to explore the mechanisms behind the evolution and adaptation of alpine woody plants [3,4]. In *Rhododendron*, *R. nivale* subsp. *boreale* Philipson et M. N. Philipson is one of the few woody flowering plants discovered to be distributed at altitudes above 5000 m and is one of the few polyploid (2n=4x=52) woody plants in the Qinghai-Tibet Plateau [1,5,6]. *R. nivale* subsp. *boreale*, a member of the subg. *Rhododendron*, is a small-leaved, highly branched shrub distributed at high altitudes of mountaintops (up to alt. 5400 m) down to the mountainsides (~3200 m). This species demonstrates remarkable adaptability, as evidenced by its diverse habitats, including alpine meadows, forest edges, and metal mining areas [7]. Currently, *R. nivale* subsp. *boreale* is an important ornamental plant resource in mountainous plateau areas and is used in traditional Tibetan medicine [8,9]. Therefore, exploring the evolutionary patterns and adaptation mechanisms of *R. nivale* subsp. *boreale* not only promotes the understanding of alpine adaptation evolution in woody plants, but also establishes a basis for the commercial exploitation of high-altitude ornamental plants.

Genetic perspectives provide a better understanding of evolution and adaptive differentiation [10]. However, the paucity of genomic data is a significant impediment to

research advancement [11]. For example, in a recent high-altitude adaptation study, only seven alpine plant genomes were used, indicating that the genetic resources of alpine plants are far from sufficient compared to the diversity of the high-altitude flora [12]. Moreover, polyploidy likely enhanced the adaptability of alpine plants to harsh environments [13]. Unfortunately, acquiring polyploid genetic data remains challenging, particularly for autopolyploids with highly similar subgenomes. Currently, the assembly of autopolyploid genomes presents significant challenges, resulting in the publication of only a select number of such genomes, including those of *Medicago sativa*, *Saccharum spontaneum*, *Solanum tuberosum*, and *Rheum officinale* [14,15,16,17]. To further understand the evolution and adaptation of the alpine flora, additional genetic resources, particularly of polyploids, are essential.

Polyploidy has been theorized to be both a potential evolutionary roadblock and a catalyst for evolutionary breakthroughs and the proliferation of species [18]. On the one hand, following polyploidy events, rapid shifts in gene expression and epigenetic modifications can bestow the polyploid with an almost instant competitive edge, which is usually reflected in their broader geographical ranges compared with their diploid ancestors [19,20]. Therefore, polyploidy tends to be ecologically advantageous and occurs in variable climatic regions, such as the Qinghai-Tibet Plateau alpine and Pan-Arctic regions [21,22]. Comprehending these adaptive mechanisms in high-altitude polyploids not only clarifies evolutionary dynamics but also provides insights into conservation strategies. On the other hand, auto and allopolyploids face a significant obstacle: the accurate segregation of chromosomes during meiosis [23,24]. In recent years, our understanding of the molecular basis for polyploid adaptations to meiotic challenges has significantly increased; however, compared to allotetraploids, little is known

about the molecular mechanisms underlying the stabilization of autotetraploid meiosis [25]. Advances in molecular technology and subsequently, more genetic resources will provide new insights into the survival, evolution, adaptation, and conservation of polyploids.

Here, we present a haplotype-resolved tetraploid genome of mountaintop plant *R. nivale* subsp. *boreale* from an altitude of 4,287.5 m, which is the first chromosome-level genome assembly of the subgenus *Rhododendron*. Based on this assembly, we identified polyploid types, deciphered whole genome duplication (WGD) events, and investigated which genes or gene families are potential candidates involved in alpine mountaintop adaptation and the survival of polyploids. This genome not only establishes the groundwork for comprehending the evolution and adaptation of *Rhododendron* species, but also offers valuable genetic resources to investigate the origin, recombination, and differentiation of polyploid species.

2. Results

2.1 Genome estimation, sequencing, and assembly

R. nivale subsp. *boreale* samples were collected from the mountaintop, treated with liquid nitrogen, and sequenced (Fig. 1A). We obtained a total of 33.35 gigabases (Gb) of PacBio CCS long reads with an average length of 15.86 kb and an N50 length of 16.15 kb (Table S1). A genome survey was performed based on DNBseq short reads (95.61 Gb; Table S2), and the result revealed an estimated genome size of 2.48 Gb, which was consistent with that estimated by flow cytometry (Fig. S1, S2; Table S3). This species was identified as a tetraploid based on *k-mers* analysis (Fig. 1E). The three initial assembly sizes assembled using Hifiasm, Canu v1.9, and Hicanu were 2.48 Gb, 2.39 Gb, and 2.40 Gb, respectively (Table S4; S8). The assembled

version of Hifiasm was used for subsequent analysis, as it ensures higher integrity of both genes and long terminal repeats (LTRs). The long reads and NGS reads were mapped to the unitig-level assembly to assess the assembly quality. Long-read and whole-genome sequencing (WGS) reads were mapped to 99.87% and 99.59%, respectively, and RNA-seq reads exhibited mapping rates exceeding 94% (Table S5). The average GC content was 41.10%. We used the AllHiC algorithm to improve the genome assembly to the chromosome level using 138.98 Gb of Hi-C data. After manual checking, a total of 2.17 Gb of unitigs were anchored to 52 pseudochromosomes (scaffold N50 = 42.93 Mb), ranging from 23.70 to 59.70 megabase (Mb) in length, and containing four haplotypes (13 pseudochromosomes per haplotype) (Fig. 1C; Table S6). The Hi-C heatmap clearly showed the interactions of 13 homologous groups (Fig. 1D), with high similarity observed between the pseudochromosomes within each homologous group.

This genome was assembled with a high consensus quality value (QV = 45.51; error rate = 0.0028%) and high *k-mer* completeness (97.79%) (Table S7). BUSCO assessment indicated that the completeness of the conserved embryophyte genes was 98.8 % (Table S8). The quality of the genome structure at the reference genome level (assembly quality indicators of large structural fragments; S-AQI = 98.69) was assessed using CRAQ (Table S9).

2.2 Annotation

A repeat sequence of 1,549,068,457 bp was identified, accounting for 62.63% of the genome assembly (Table S10). The richest category of repeats was LTRs (43.12%), with *Gypsy* and *Copia* accounting for 33.66% and 5.88% of the repeats, respectively (Fig. S3). In addition, the LTR assembly index (LAI) was greater than 14 (n1:14.78, n2:14.84, n3:14.35, and n4:14.38)

based on LTR annotation, which indicated that the assembly met the reference category standards. By combining ab initio, homology, and transcriptome data predictions, 127,810 protein-coding genes were predicted, with an average gene length of 4,736.07 bp. The total length of the coding sequences (CDS) was 148,274,600 bp, and the average number of CDSs per gene was 4.8 (Table S11). The completeness of 98.6% of the annotated protein-coding genes of *R. nivale* subsp. *boreale* was assessed using BUSCO. Of the protein-coding genes, 96.86% were annotated functionally (Table S12). The ratio of mono-exonic (single-exon) to multi-exonic (multiple-exon) was 0.245. We annotated 17,049 candidate noncoding RNAs, including 703 microRNAs (miRNAs), 3,672 transfer RNAs (tRNAs), 5,373 small nuclear RNAs (snRNAs), and 7,301 ribosomal RNAs (rRNAs) (Table S13).

2.3 Confirmation of Autotetraploid

Polyploids are commonly found in plants. However, the origins of the polyploids differ and include both homologous and heterologous origins. Extensively studied allotetraploids such as peanuts, cotton, and wheat [26,27,28], exhibit significant subgenomic differences, allowing for their division into distinct subgenomes. In contrast to allotetraploids, the high similarity among haplotypes greatly increases the difficulty in assembling autotetraploids. To determine the polyploid type of *R. nivale* subsp. *boreale*, we employed *k-mer* analysis, collinearity analysis, and phylogenetic analysis for cross-validation. The 21 *k-mer* frequency analysis revealed four distinct peaks (located at 33, 68, 106, and 136) (Fig. S2), which was highly similar to the results for autotetraploids (*Medicago sativa* and *Saccharum spontaneum*). Nucleotide heterozygosity is an important criterion for determining polyploid types [29]. Nucleotide heterozygosity form analysis of *R. nivale* subsp. *boreale* showed 2.53% AAAB and 1.28%

AABB (Table S3), which was consistent with the expectation that the heterozygous rate of autotetraploid AAAB would be greater than that of AABB.

Nevertheless, these methods were insufficient to identify the polyploid type. For example, although genomic analysis indicated higher AAAB than AABB, *Artemisia argyi* was identified as an allotetraploid [30]. To further determine the polyploid type of *R. nivale* subsp. *boreale*, synteny analysis was performed based on syntenic blocks. As expected, the dot plot and syntenic blocks indicated synteny among the four haplotypes (Fig. 2A), with 20,172 gene pairs showing synteny between haplotypes 1 and 2, 20,249 between haplotypes 2 and 3, and 19,883 between haplotypes 3 and 4 (Fig. 2B, S4). Additionally, we downloaded transcriptome data from 11 samples of seven closely related species (Table S14) to infer the phylogenetic positions of the four haplotypes of *R. nivale* subsp. *boreale* and identified a monophyletic group consisting of six species (*R. nitidulum*, *R. hippophaeoides*, *R. thymifolium*, *R. nivale*, *R. nivale* subsp. *Boreale,* and *R. lapponicum*) of subsect. *Lapponica* with high support. A clade containing all *R. nivale* subsp. *boreale* (including the transcriptome and four haplotypes) and two *R. nivale* was supported by 100% bootstrapping (Fig. 2 C).

Moreover, 25,791 orthogroups were identified in the four haplotypes, with 16,403 shared by all, 5,242 shared by three, 3,631 shared by two, and only 515 (n1:134; n2:139; n3:135; n4:107) unique to each haplotype genome, showing high genetic similarity among haplotypes (Fig. 2D). Overall, the combined results of *k-mer* analysis, collinearity analysis, and phylogenetic analysis indicated that *R. nivale* subsp. *boreale* is an autotetraploid species.

2.4 Comparative analysis and recent polyploidization

The phylogenetic position and divergence times of *R. nivale* subsp. *boreale* were inferred

from 18 other species, including 12 species of Ericales (10 *Rhododendron*), two species of Cornales, one species of Gentianales, one species of Vitales, two species of monocotyledons, and one sister species to all angiosperms (*Amborella*). Altogether, 666,442 genes were used to infer orthology. A total of 625,661 genes (93.9%) clustered into 37,844 orthologous gene families, of which 6,547 were shared across all species (Fig. 2B; Table S15). In total, 209 single-copy gene families were identified. In total, 146 gene families, comprising 369 genes, were found to be specific to *R. nivale* subsp. *boreale* (Fig. S5). These species-specific genes were enriched in 12 Kyoto Encyclopedia of Genes and Genomes (KEGG) pathways and 136 gene ontology (GO) terms including arginine biosynthesis, nitrogen metabolism, and flavonoid biosynthesis (Tables S16 and S17).

In total, 209 single-copy orthologous genes were used to reconstruct phylogenetic relationships using IQ-TREE. All nodes were supported by high bootstrap values (> 95%). The results supported that *Rhododendron* is a monophyletic group, and the 10 species of *Rhododendron* were divided into four clades representing four subgenera (*Tsutsusi*, *Rhododendron*, *Pentanthera,* and *Hymenanthes*) (Fig. 3A). The time tree inferred from the MCMCtree suggested that the ancestor of *Rhododendron* separated from the common ancestor of *Rhododendron* and *Va. darrowii* approximately 41.2 Mya. The split between *R. nivale* subsp. *boreale* and the sister groups *(R. mole, R. henenense, R. delavayi, R. griersonianum, and R. irroratum*) was 30.4 Mya, while the divergence time of *R. molle* was 28.3 Mya (Fig. 3A).

The synonymous substitution rate (*Ks*) of orthologs and paralogs of seven species (four *Rhododendron* species, one *Vaccinium*, one *Actinidia* species, and one *Vitis*) was calculated to determine the WGD events that occurred in *R. nivale* subsp. *boreale*. Polyploidy analysis

indicated that *Rhododendron* and *Va. darrowii* experienced two rounds of ancient polyploidy events, whereas *Ac. chinensis* experienced three. Similar peaks were observed for all four *Rhododendron* species and *Va. darrowii*. And the farthest peak revealed an ancient γ whole-genome triplication (WGT-γ) event common to *Rhododendron* and other core eudicots, which was inferred to have occurred 122–164 Mya from a previous study [31]. In addition, the peak at *Ks* of the paralogs approximately 0.65 Mya suggests another polyploidization event in *Rhododendron*, *Va. darrowii,* and *Ac. chinensis* estimated to have occurred at approximately 78 Mya (Fig. 3C, S9). The dot plot comparing *Vi. vinifera* and *R. nivale* subsp. *boreale* (Fig. 3D, S7–8), nearly every grape chromosome exhibited two highly compatible chromosomal regions in *R. nivale* subsp. *boreale* (orthologous ratio 1:2) (Fig. 3D).

2.5 Analysis of positive selection

Using multiple models, we expected to provide more accurate detection of the selection signals of *R. nivale* subsp. *boreale*. First, adaptive Branch-Site Random Effects Likelihood (aBSREL) was used to test for positive selection in the high-altitude branch of each gene by examining 28 positively selected genes (PSGs). Subsequently, we employed clade model C (CmC) to further evaluate foreground and background selection differences compared with the null model M2a_rel to exclude genes not affected by selection pressure (Table. S18). Sixteen genes were identified as PSGs (Table. S19). Positive selection sites were detected using the Mixed Effects Model of Evolution (MEME) and the Contrast-FEL, with the number of positive selection sites per gene ranging from 0 to 19. These two genes exhibit no significant positive selection sites. In addition, 19 genes were identified as PSGs using KaKs_Calculator and were functionally annotated. (Table. S20). These PSGs are associated with various

biological processes, including meiosis recombination (*TOP3α*), nucleotide excision repair (*UVR8*, *RAD23B*), leaf surface wax metabolism (*LTG30*, *LTP1*), auxin transporter (*ABCB19*), signal transduction and regulation (*M3K1*, *CAGC1*), and biological clock regulation (*ESD4*). Additionally, we explored the selection pressure within 13 homologous groups where similar pressures were observed. (Fig. S6).

2.6 Gene duplication and family evolution

Based on the ultrametric tree, the gene family evolution of 19 species was compared with that of the most recent common ancestor (MRCA). Overall, 3,356 orthogroups expanded in *R. nivale* subsp. *boreale*, while only 756 orthogroups contracted. Among these, 375 and 79 orthogroups expanded and contracted significantly, respectively. GO and KEGG enrichment analyses suggested that the significantly expanded orthogroups were primarily enriched in pathways such as brassinosteroid (BR) biosynthesis, terpenoid biosynthesis, and isoflavonoid biosynthesis (Fig. 4A).

To explore the connection between gene duplication and gene family expansion, 20,673 duplicated genes were identified and classified into five categories: 3,899 whole-genome duplication (WGD; 18.86%), 2,711 transposed duplication (TRD; 13.11%), 3,744 tandem duplication (TD; 18.11%), 4,738 proximal duplications (PD; 22.92%), and 5,581 dispersed duplication (DSD; 27.00%) duplications (Fig. 4B). Among these, PD and TD contributed the most to the expansion of gene families. Moreover, ω (*Ka/Ks*) ratios of all duplication categories were calculated, revealing that PD and TD demonstrated superior ω scores compared to other types, while the lowest ω score was for WGD (Fig. 4C). KEGG functional enrichment analysis indicated that the functions of genes shared by the significantly expanded

orthogroups and five different duplication types were differentiated. WGD genes were enriched in plant hormone signal transduction and nucleotide excision repair, TRD duplications were implicated in plant-pathogen interactions and *O*-glycan biosynthesis, gene family expansions related to arachidonic acid metabolism and linoleic acid metabolism were mainly contributed by DSD, and duplications of TD and PD were associated with BR biosynthesis, cytochrome P450, and isoflavonoid biosynthesis (Fig. 4D; Table S21–S30).

2.7 Expression of alleles

Transcriptome data from the roots, stems, leaves, and buds of *R. nivale* subsp. *boreale* were used to explore allelic expression patterns. Overall, 77,892 genes, representing 60.94% of all the genes, were expressed in at least one tissue. In single matching four alleles (1:1:1:1), 12,642 out of the 14,550 alleles were expressed in at least one allele. The expression levels of chromosomes within each homologous group were similar, with homologous group 3 showing a higher transcript expression than the other groups (Fig. 5A). Homologous Group 10 had the lowest expression level. We selected genes with a transcripts per kilobase per million mapped reads (TPM) value $\geq$ 1 to compare the differences in expression levels between haplotypes, and 6,388 single-match gene groups were identified. Finally, 3,844 of the 6,388 (60.17%) single-match gene groups were identified as differentially expressed loci (DELs), with DEL ratios ranging from 56.14% to 63.93% per pseudochromosome. The DELs were randomly distributed across the genome (Fig. 5B).

2.8 Evolution of the APETALA2/ethylene responsive factor (AP2/ERF)

Plant APETALA2/ethylene responsive factors (AP2/ERFs) and cytochrome P450s (CYPs), which likely play important roles in the adaptation of plants to high altitudes, participate in a

multitude of biochemical pathways and fulfill various functions in the realms of growth and protection, including responses to UV irradiation, dehydration, and pathogens [32,33]. Therefore, AP2/ERF and CYP families were explored. We identified the AP2/ERF family in 10 *Rhododendron* and two related species (*Actinidia chinensis* and *Vaccinium darrowii*) using the HMMer method. In total, 2,397 genes were identified as belonging to the AP2/ERF family in 12 species after artificially confirming the presence of the AP2 domain (Table S31). For convenience, haplotypes were extracted from the haplotype-resolved assemblies of *R. nivale* subsp. *boreale* and *R. vialii* to compare the gene counts within the family. Among *Rhododendron* species, *R. ovatum* exhibited the highest gene count (163), while *R. irroratum* contained only 88 genes. The majority of species in our study had gene counts of approximately 140. These genes were randomly distributed across 13 pseudochromosomes (Fig. S10-21). To further understand the phylogenetic mechanisms of the AP2/ERF family in *Rhododendron*, proteins of *Ar. thaliana* and other 12 species were used to construct the phylogenetic tree. Consistent with previous research, 13 categories, including the AP2, ERF (B-1 to B-6), DREB (A-1 to A-6), RAV, and soloist subfamilies, were identified (Fig. 6A). Compared with *Ar. thaliana*, the categorization of B-3 was expanded in *Rhododendron*.

The alpine environment is variable, with significant temperature differences between day and night, strong ultraviolet radiation, and low oxygen partial pressure. Group VII ethylene response factor transcription factors (ERF VIIs) are associated with altitude adaptation [34]. Hence, based on the homology of *ERF VIIs* in *Ar. thaliana*, we identified *ERF VIIs* in *Rhododendron*. Ten species of *Rhododendron* contained one (*R. molle*) to five (*R. simsii*) *ERF VIIs* (Table S32). The phylogenetic tree topology showed that *ERF VIIs* were divided into four

groups. *ERF VIIs* of *Ar. thaliana* genes were found in Groups I, III, and IV (Fig. S22). Group II consisted solely of Ericales genes, and one clade contained the alpine species of *Rhododendron*. Based on motif analysis, similar gene structures within the groups showed phylogenetic reliability (Fig. S23). Interestingly, we estimated the turnover of *ERF VII* subfamilies using the maximum likelihood method and found a continuous decrease in the number of genes (Fig. 6B). However, among the 13 *ERF VII* genes, 12 were expressed in the roots, stems, leaves, and buds of *R. nivale* subsp. *boreale* and exhibited significantly higher expression levels than the global gene expression (Fig. 6C).

C-repeat binding factors/dehydration-responsive element binding protein 1 (*CBFs/DREB1s*) play a crucial role as transcription factors that regulate gene expression during cold acclimation. Consistent with previous studies, *CBFs* were categorized as A-1 of the dehydration-responsive element binding (DREB) subfamily, which is separated from A-4 of DREB [35]. We used the BLASTP program and two conserved sequences, PKRxAGRxKFxETRHPV and DSAWR, surrounding the AP2/ERF domain to accurately identify *CBF* genes. Sequence comparison with *Ar. thaliana* revealed that *CBFs* have a highly conserved domain. A total of 49 *CBFs* were identified from 10 *Rhododendron* species, of which *R. nivale* subsp. *boreale* contained 16 *CBFs* (12 *CBF* genes with four alleles and 2 *CBF* genes with two alleles) (Table S32). Phylogenetic analysis indicated that *CBFs* of *Rhododendrons* could be divided into three groups (Fig. S24). Similar conserved motifs were observed in each group, indicating the reliability of the relationship of *CBFs* (Fig. S25). In addition, family turnover based on BadiRate was re-validated, and *CBFs* were continuously lost without gain, resulting in the contraction of *CBF* genes (Fig. 6B). Approximately only half

(9/16) of the *CBFs* were expressed (Fig. 6C).

2.9 Evolution of the CYP family

Using the *R. nivale* subsp. *boreale* genome we assembled, along with the genomes of 12 other closely related species, we investigated the evolutionary pattern of the CYP family. The number of CYP family members in each species ranged from 221 to 447, as determined using local BLASTP, hmmsearch, and manual checks (Table S33). Among them, *R. irroratum,* which contains 447 CYPs, had the highest gene count. The identified CYP proteins varied in length, ranging from 303 to 621 amino acids. In addition, CYPs were unevenly located on different pseudochromosomes (Fig. S10-21).

To determine the phylogenetic relationship between CYPs, we constructed an unrooted maximum likelihood (ML) phylogenetic tree using protein alignments that primarily contained conserved domains and compared them with CYP superfamily members from *Ar. thaliana*. Following the classification system proposed [36], all CYPs were categorized into two distinct types: A-type, which comprises the CYP71 clan, and non-A-type, which comprises the CYP51, CYP72, CYP74, CYP85, CYP86, CYP97, CYP710, CYP711, and CYP727 clans. Our analysis confirmed monophyly within each clan, with the CYP71 clan being the most gene-rich, representing over half of all identified CYPs. In contrast, the CYP711 and CYP727 clans were the smallest (Fig. 7A–C). In comparison to *Ar. thaliana* and *Ac. chinensis*, we noted a species-specific increase in the CYP family across different *Rhododendron* species, particularly within the CYP71, CYP85, and CYP72 clans. These expansions are likely to play pivotal roles in species-specific adaptations. The CYP71 clan was mainly involved in the biosynthesis of alkaloids, sesquiterpenoids, cyclic terpenoids, and flavonoids, whereas the

CYP85 clan is implicated in the modification of cyclic terpenes and sterols in the BR, abscisic acid (ABA) and gibberellin (GA) pathways. CYP72 is involved in isoprenoid hormone catabolism. Unexpectedly, the CYP family of *R. henanense* subsp. *lingbaoense* was contracted, particularly the CYP72 clan (Fig. 7B). The patterns of duplicated gene pair identification showed that PD and TD remarkably contributed to the CYP family variation. In addition, WGD events accounted for a large proportion of duplications in *R. nivale* subsp. *boreale* and *R. ovatum* (Fig. 7D).

3. Discussion

Climate change is expected to significantly impact mountaintop ecosystems. Understanding the evolutionary patterns and survival strategies of mountaintop species is imperative to protect them [37]. Polyploidy is beneficial for the survival of species in harsh environments [21]. Therefore, it is essential to understand the evolution and adaptation of extremely high-altitude environmental species from the perspective of polyploidy. In this study, we provide a chromosome-scale and haplotype-resolved autotetraploid genome of *R. nivale* subsp. *boreale* using the DNBseq, PacBio CCS, and Hi-C sequencing platforms. The *R. nivale* subsp. *boreale* genome contains 52 pseudochromosomes divided into 13 homologous groups. Our assembly was estimated to be of high quality using four methods (Merqury, BUSCO, CRAQ, and LAI). In addition, we used various methods to determine autotetraploid identity. As the first autotetraploid genome of an alpine woody plant, the genome of *R. nivale* subsp. *boreale* has laid an important foundation for understanding the adaptation and evolution of woody plants in harsh environments at high altitudes. Consistent with previous systematics of *Rhododendron* studies, Subg. *Rhododendron,* to which *R. nivale* subsp. *boreale* belongs, is a

sister of Subg. *Pentanthera* and Subg. *Hymennanthes* [4]. Furthermore, our data suggest that the ancient WGD event in *R. nivale* subsp. *boreale* occurred approximately 78 Mya, which is probably shared with Ericaceae [38]. Overall, *R. nivale* subsp. *boreale* has recently experienced an additional WGD event, in addition to the WGT-γ event shared by the core eudicots and the WGD event shared by the Ericales. Recent polyploidy events, like those observed in other polyploids, are likely important factors for highly conserved pseudochromosomes without rearrangements [31]. The selection analysis revealed similar selection pressures in homologous groups (Fig. S6). The different subgenomes of allopolyploids were under significantly different selection pressures, and it has been speculated that different haplotypes of autopolyploids probably also faced different selection pressures [39]. However, the distribution of *Ka*/*Ks* values did not differ between our homologous groups (Fig. S6). Based on gene family evolution and positive selection analysis, we postulate the potential high-altitude adaptation strategies for *R. nivale* subsp. *boreale*.

Mountaintop ecosystems are exposed to high levels of UV radiation, low partial pressures of oxygen, and volatile temperatures and humidity [38]. Understanding the mechanisms of the adaptation of plants to high altitudes has long interested botanists. Altitude is positively correlated with UV radiation, with UV radiation rates increasing by 5.1–15% every 1,000 m increase in altitude [40]. Alpine plants employ various mechanisms to mitigate the effects of UV radiation, including cell wall surface modifications and the creation of a leaf cuticle consisting of cutin and cuticular waxes [41]. This cuticle serves as a protective shield against water loss and excessive UV radiation by forming a physical barrier between the plant surface and the environment [12]. Second, to enhance their tolerance to UV radiation and protect

themselves from UV damage, plants accumulate flavonoids that absorb UV radiation from strong light [42]. Several genes involved in cuticle and UV tolerance, such as *CER1*, *FARs,* and *MYB27*, are positively selected in high-altitude plants [12]. We identified a similar situation in *R. nivale* subsp. *boreale* living in a high-altitude mountaintop environment. *LTP1* and *LTPG30* have been identified as PSG, of which *LTP1* is associated with the biosynthesis and secretion of cuticular wax [43]. In wild-type bilberry (*Vaccinium myrtillus*), which is closely related to *Rhododendron*, expression of the *LTP* gene specific to the skin suggests its involvement in transporting wax compounds into the cuticle [44]. *LTPG30* performs similar functions [45]. Therefore, surface modification of the cell wall is likely to be the initial line of defense against UV damage in *R. nivale* subsp. *boreale*.

Flavonoids are widely recognized as important chemical compounds that protect plants from UV radiation [46]. We observed a significant expansion of the flavonoid gene family, which suggests that the absorption of UV radiation by flavonoid synthesis may be one of the key ways to reduce UV damage in *R. nivale* subsp. *boreale*. This finding was consistent with the conclusions of previous studies [12]. However, the creation of a wax barrier and biosynthesis of flavonoids that absorb UV radiation are insufficient to fully shield plant cells from the intense UV radiation found in mountainous environments. UV radiation not blocked by such barriers reaches deep into cells, damaging biological macromolecules such as DNA, thereby affecting the growth and development of various cells [47]. In this study, *UVR8* and *RAD23B*, which contribute to DNA repair, were positively selected. Specifically, *UVR8* enhances UV-B perception by interacting with the photomorphogenic repressor *COP1*, while *RAD23B* primarily collaborates with *RAD4* to facilitate nucleotide excision repair [48,49].

These interactions are likely to augment the UV tolerance in *R. nivale* subsp. *boreale.*

In alpine environments, plants have evolved myriad morphological and physiological adaptations to contend with the rigor of high-altitude conditions [50]. *R. nivale* subsp. *boreale* native to mountaintops, typically reaches heights of less than 30 cm, with leaves that seldom exceed 5 mm in both length and width, exhibits delayed flowering, and produces seeds that are nearly indiscernible. These characteristics are thought to be a response of *Rhododendron* to low temperatures at high altitudes, poor nutrition, and extremely short growth cycles [51]. Selection analysis revealed PSGs related to auxins, morphogenesis, and the biological clock. For instance, some PSGs identified are associated with auxin transport (*ABCB19*), seed size (*DA1*), and the biological clock (*ESD4*) [52,53,54]. Concurrently, the gene family for organ development, tissue development, and auxin polar transport expanded significantly. These genes and gene families, which are involved in growth and development, likely shape the special morphology of high-altitude plants such as *R. nivale* subsp. *boreale* and regulate the different stages of their developmental cycle in response to environmental changes, thus better adapting to the extreme spatial and temporal heterogeneity of mountaintop ecosystems [12,55].

Low temperature (average annual temperature below 0 °C), low partial pressure of oxygen (for every 1,000 m increase in altitude, air pressure drops by about 11%) and rapid weather changes (annual average diurnal temperature exceeding 20 °C) are the main factors limiting alpine plant survival [56,57,58]. Under harsh alpine conditions, plants adapt by modifying their morphology, producing specific metabolites, and changing the distribution patterns of biomass [55]. In *R. nivale* subsp. *boreale,* we observed a significant expansion in the gene family associated with BRs, which are pivotal for sustaining plant physiological functions and

significantly contribute to enhancing cold tolerance, drought resistance, and antioxidative capabilities [59,60]. Hence, the expansion of the BR gene family probably enhanced the ability of *R. nivale* subsp. *boreale* to adapt to dramatically changing environments. Moreover, we speculated that the expansion of CYPs, which are associated with stress response, could potentially facilitate the successful adaptation of *R. nivale* subsp. *boreale* at high altitudes. To further understand the adaptability of *R. nivale* subsp. *boreale*, we assessed the dynamics of an important family of transcription factors (AP2/ERF), which is an important group of transcription factors responsive to abiotic stress [61], including key members that adapt to alpine conditions such as *ERF VIIs* and *CBFs/DREB1s*. *ERF VIIs* and *CBFs* are important transcription factors that respond to low oxygen partial pressures and temperatures [34,62]. Unexpectedly, the genes of *Rhododendron* for *ERF VIIs* and *CBFs* continued to be lost lost in low-oxygen, cold alpine environments. *R. nivale* subsp. *boreale* distributed over the alt. 4000 m is no exception. The high expression levels of *ERF VIIs* in *R. nivale* subsp. *boreale* suggests a potential adaptation to low oxygen environments, which warrants further experimental investigation. In response to temperature changes, *Rhododendron* species rely on several pathways to enhance their low-temperature resistance, including *CBFs*-mediated cold tolerance and an integrated regulatory network of ABA, the MAPK cascade, and Ca^{2+} signaling [63,64]. Our positive selection analysis results suggested that *M3K1* and *CNGC1,* which are associated with the MAPK cascade and Ca^{2+} signal transduction, probably play important roles in low-temperature adaptation.

Polyploidy leads to rapid changes in gene expression and epigenetics, giving the polyploid a significant selective advantage over its diploid progenitors and serving as a crucial

mechanism for plants to swiftly adjust to severe environmental stress [6,18]. Moreover, differential splicing, which is a crucial mechanism in the eukaryotic stress response, changes rapidly after polyploidy and is associated with abiotic stress [65]. Certainly, the influence of genome doubling on phenotypes or life history traits can directly affect the likelihood of survival under challenging circumstances [18]. These include more viable seeds, more rapid growth, and stronger photosynthesis [19,66]. These characteristics provide plants with great advantages under adverse environmental conditions.

The decline in the fitness of autopolyploids, especially young autopolyploids, is usually attributed to multivalent chromosome pairing during meiosis and mutations in crossover (CO) frequency and distribution [23,67]. Consequently, autopolyploids experience significant disruptions in their developmental programs, resulting in a considerable reduction in seed production and a high incidence of aneuploid offspring [68]. Addressing these issues requires precise adaptive control of meiosis, such as reduced formation of multichromosome associations and reduced axis lengths [69]. The RECQ4a/4b (BLM)-TOP3α-RMI1 (BTR) complex plays a pivotal role in limiting CO outcomes and maintaining chromosome integrity [70,71]. Mutations of *RECQ4*, one of its members, significantly affect the stability of polyploid meiosis [72]. Notably, *TOP3α*, an important gene related to the BTR complex, exhibits positive selection in *R. nivale* subsp. *boreale* and likely serves as a key factor in promoting accurate chromosomal segregation during meiosis in autopolyploids.

Our results revealed that *R. nivale* subsp. *boreale* distributed on the mountaintop is an autotetraploid, which is probably mediated by the harsh environment at high altitudes. Paleopolyploid events are shared among the other 10 *Rhododendron* species of diploids. Our

conjecture regarding the alpine adaptation mechanisms of *R. nivale* subsp. *boreale* aligns with those of previous studies: cell wall modification, flavonoid biosynthesis, DNA repair, inhibition of chlorophyll synthesis, and auxin and BR biosynthesis and transduction are probably the main high-altitude adaptation pathways [12]. Polyploidization likely plays an important role in mountaintop survival because of its dominant gene expression pattern [18]. Notably, *TOP3α* is speculated to be an important gene during meiosis in autotetraploids, essential for the generation of normal gametes and implicated in the attenuation of CO events during meiosis. However, these hypotheses require verification through biological experiments. Moreover, the mechanism of the formation of natural polyploids remains to be fully elucidated, and the alpine environment, where polyploidy is concentrated, offers an ideal setting for investigation.

Overall, we assembled the first genome of Subge. *Rhododendron*, a rare high-altitude woody autotetraploid genome that provides an important resource for the domestication of high-altitude ornamentals and our understanding of polyploid origin and evolution in mountaintop ecosystems.

4. Materials and methods

4.1 Plant materials and sequencing

R. nivale subsp. *boreale* plant materials were collected from Baima Mountain, Dêqên County, Yunnan Province, China (99°4′13″E, 28°20′24″N, alt. 4287.5 m). The plant materials were immersed in liquid nitrogen immediately after collection and preserved at -80 °C. High-quality DNA isolated from young leaves was used to create the libraries. Long-read libraries

were constructed and sequenced using the PacBio Sequel II sequencing platform. To construct Hi-C libraries, genomic DNA was cross-linked with formaldehyde and digested using the MboI restriction enzyme into 300–500 bp fragments, which were sequenced on the BGI DNBseq sequencing platform. For short reads, DNA libraries were constructed and sequenced on the BGI DNBseq sequencing platform. Three biological replicates of roots, stems, leaves, and buds of *R. nivale* subsp. *boreale* were sampled. The cDNA libraries were constructed and sequenced on a BGI DNBseq sequencing platform.

4.2 Genome survey

Flow cytometry and *k-mer* analysis were used to evaluate the genome of *R. nivale* subsp. *boreale*. The following procedures were used for flow cytometry: preparation of nuclear suspension, DNA-specific staining, and testing. We selected *R. griersonianum* as an internal control. Graphical analysis was performed using ModFit LT 5.0 (www.vsh.com/products/mflt/index.asp) with a coefficient of variation (CV) controlled to within 5. For *k-mer* analysis, DNBseq short-reads clean data were used to count *k-mer* frequency with *k-mer* set to 21 using jellyfish v2.3.0 [73]. Genome size was estimated based on the 21 *k-mers* distribution. Ploidy was estimated using SmudgePlot v0.2.5 [29].

4.3 Genome assembly and scaffolding

The PacBio circular consensus sequencing (CCS) long-read data were assembled using Hifiasm v0.18.9 with Hi-C integration [74], Canu v1.9 [75], and HiCanu v2.2 [76]. We used the parameters of the genome of *Saccharum spontaneum* [16]. The integrity and continuity of the assembly were assessed separately, and the highest quality assembly was used for the scaffolding. The ALLHiC pipeline was used to improve assembly at the chromosomal level

based on five steps: pruning, partitioning, rescue, optimization, and construction [77]. Manual checks were conducted on potential misassemblies and corrected using Juicebox v1.11.08 [78]. Finally, the assembled genome was evaluated using Benchmarking Universal Single-Copy Orthologs (BUSCO) v5.4.6 [79], Merqury v1.3 [80], and Clipping information for Revealing Assembly Quality (CRAQ) v1.0.9 [81] using default parameters. Short reads mapped to the assembled genome using BWA v0.7.17-r1188 [82] and SAMtools v1.17 [83] were counted as properly paired.

4.4 Genome annotation

De novo prediction and homology alignment were used to identify whole-genome repeats. The LTRs were initially identified using LTRharvest [84] and LTR_Finder [85]. LTR_retriever v2.9.4 [86] was used to accurately identify LTR retrotransposons (LTR-RTs), generate a nonredundant LTR-RT library, and generate the LTR assembly index (LAI). A homology search was conducted to predict repeat elements using RepeatMasker v4.1.4 [87]. Transfer RNAs (tRNAs) were annotated using tRNAscan-SE v2.0.9 [88], and ribosomal RNAs (rRNAs) were identified using RNAmmer v1.2 [89]. Other noncoding RNAs, including miRNAs and snRNAs, were annotated by comparison using Infernal v1.1.4, with the Rfam database [90,91].

We combined ab initio, homolog and transcriptome-based strategies to predict the expression of high-quality protein-coding genes. In our transcriptome-based strategies, we used HISAT2 v2.2.1 [92] to align clean reads of the transcriptome with the genome. Trinity v2.14.0 [93] and StringTie v2.2.1 [94] were used to assemble transcripts. BRAKER3 [95] and PASA v2.5.2 [96] were used to predict gene structure based on the assembled transcripts and to generate ab initio gene predictor training sets. For ab initio, SNAP [97], GlimmerHMM v3.0.1,

and GeneID v1.4 [98] were used to annotate gene structures based on the training sets. For homology-based prediction, protein sequences from a total of eight species, namely, *Arabidopsis thaliana, Vitis vinifera, Glycine max, Nicotiana attenuata, Oryza sativa, R. ovatum, R. griersonianum,* and *R. mole*, were aligned with the genome of *R. nivale* subsp. *boreale* using GeMoMa v1.9 [99]. All gene structures annotated using the above approaches were integrated using the EVidenceModeler (EVM) [100]. Functional annotation of genes was performed using EggNOG v5.0 [101], and protein sequences were aligned to the UniProt database using BLAST v2.6.0 [102].

4.5 Identification of polyploid type

Used GenomeScope 2.0 [29] to count the proportion of nucleotide heterozygosity forms based on the 21 *k-mer* count distributions. AAAB < AABB indicates allotetraploidy, whereas AAAB > AABB indicates autotetraploidy. JCVI utility libraries [103] were used to analyze collinear relationships between haplotypes. To identify the relationship between different haplotypes and related species, 11 transcriptome datasets from seven related species from previous studies with four haplotypes and transcripts of *R. nivale* subsp. *boreale* were used to reconstruct the phylogenetic tree. StringTie v2.2.1 [94] was used to assemble transcripts. A maximum likelihood (ML) tree was reconstructed using IQ-TREE v2.2.2.2 [104] with 1000 ultrafast bootstrap replicates after single-copy orthologs were identified by OrthoFinder v2.5.4 [105].

4.6 Comparative genomics analysis

The genomes of *Actinidia chinensis, Amborella trichopoda, Camptotheca acuminata, Davidia involucrata, Oryza sativa, Panicum hallii, R. delavayi, R. griersonianum, R. henanense*

subsp. *lingbaoense, R. irroratum, R. molle, R. ovatum, R. ripense, R. simsii, R. vialii, Vaccinium darrowii,* and *Vitis vinifera* were used for comparative genomics analysis with our assembly of *R. nivale* subsp. *boreale*. Single-copy orthologs were identified based on protein sequences using OrthoFinder v2.5.4 [105]. The protein sequences in each single-copy orthogroup were aligned using MUSCLE v5.1 [106] and filtered using trimAI v1.4 [107] and used to construct a phylogenetic tree using IQ-TREE v2. 2.2.2 [104] with 1000 ultra-fast bootstrap replicates. The MCMCtree program in PAML v4.10 [108] was used to estimate the divergence times. Calibration times were obtained from the TimeTree database (http://timetree.org) and previous studies [4,109]. A total of four calibration points were used to calibrate age: angiosperms 168–194 Mya; monocots eudicots 142.1–163.5 Mya; *Rhododendron* crown 54.5 Mya; *Panicum hallii-Oryza sativa* 41.4–51.9 Mya. Based on the ultrametric tree, the expansion and contraction of gene families were estimated using CAFÉ 5 [110]. Functional enrichment analysis of Gene Ontology (GO) and Kyoto Encyclopedia of Genes and Genomes (KEGG) was performed using the R package clusterProfiler v4.8.3 [111]. Synteny between different species was identified and visualized using the MCscan pipeline in JCVI [103] and MCScanX [112] with default parameters. The *Ks* values of the ortholog and paralog pairs were calculated using KaKs_Calculator v2.0 [113] after alignment with ParaAT v2.0 [114]. WGD times were estimated as T=*Ks*/2r (T is the WGD time and r is the rate of divergence). The value of r was obtained from a previous study [115].

4.7 Selective analysis

Based on 1,122 single-copy conserved orthologs from Ericales (10 *Rhododendrons, Va. darrowii,* and *Ac. Chinensis*), we performed the positive selection analysis acting on the *R.*

nivale subsp. *boreale* clade by running separate aBSREL, Clade Model, MEME, and Contrast-FEL. aBSREL was implemented in HyPhy v2.5.48 [116] with exploratory analysis, representing an improved version of traditional "branch-site" models. The aBSREL test models both site-level and branch-level nonsynonymous-to-synonymous mutation ratio ω heterogeneity but does not test for selection at specific sites. To obtain more accurate PSGs, we used clade model C (CmC) to check the consistency of the model in PAML [108]. This model tested the differential selection pressure between the foreground branches and background for each gene. CmC was then compared with the null model M2a_rel using likelihood-ratio tests (LRT) [117].

To obtain information on specific sites during episodic selection, we applied MEME and Contrast-FEL. MEME tests [118] for sites that were subjected to episodic positive or diversifying selection were performed for each gene. The MEME employs a mixed-effects maximum likelihood approach to test the hypothesis that individual sites are subject to episodic, positive, or diversifying selection. For each site, MEME infers two ω rate classes and the corresponding weights representing the probability that the site evolves under each corresponding ω rate class at a given branch. Contrast-FEL [119] was used to estimate the difference in ω at each site between different branch sets in codon alignments. The false discovery rate (FDR) was used to correct for multiple comparisons.

To further understand the selection pressure characteristics of the high- and low-altitude genomes, we used KaKs_Calculator [113] to detect selected genes between *R. nivale* subsp. *boreale* (high altitude) and *R. ovatum* (low altitude). All genes with a P value < 0.05 and ω (Ka/Ks) > 1 were identified as candidate PSGs.

4.8 Gene expression analysis

Clean reads of the transcriptome were mapped to the genome using STAR and gene expression levels were estimated using STAR v2.7.10b [120]. Accurate quantification (transcripts per kilobase per million mapped reads) of genes was performed using RSEM v1.3.3 [121]. We selected the expression levels of single-match alleles to explore the differences in expression between alleles. The four alleles were compared pairwise to identify the differentially expressed alleles. Pairs of alleles exhibiting less than a twofold difference in expression were classified as neutral, whereas all other pairs were categorized as non-neutral, that is, DEL. [122]. We used the Kruskal–Wallis test to assess differences in median values among multiple independent samples. The level of significance was set at P value $<$ 0.05.

4.9 Identification of duplicate gene modes

Different modes of duplicated gene pairs were identified using the DupGen Finder pipeline [123]. The duplicated gene pairs were divided into five categories: whole-genome duplicates (WGD), tandem duplicates (TD), proximal duplicates (PD), transposed duplicates (TRD), and dispersed duplicates (DSD).

4.10 Identification and analysis of key gene families

The AP2/ERF and cytochrome P450 (CYP) gene families were identified using HMMER v3.3.2 (HMMER.org). The structural domain files corresponding to AP2/ERF (PF00847) and CYP (PF00067) were obtained from the Pfam database (https://www.ebi.ac.uk/interpro/). A domain file is used as the first template to search for a family. The filtered domain sequences were used as species-specific templates in the second scan. The Pfam and CDD databases (https://www.ncbi.nlm.nih.gov/cdd/) were used to verify conserved domains. Conserved

sequences containing the main domains were aligned using MAFFT v7.520 [124] and used to construct a phylogenetic tree of the gene family using FastTree v2.1.11 [125] with the GTR + CAT model. Phylogenetic analysis of *CBFs* and *ERF VIIs* was performed using IQ-TREE v2.2.2.2 with 1000 replicates [104]. Gene motifs were predicted using MEME software v5.5.1 and visualized using TBtools v2.003 [126]. BadiRate v1.35 [127] was used to estimate family turnover rates based on likelihood-based methods.

Data availability

The raw sequencing data of this study have been deposited in the Sequence Read Archive (SRA) under Bioproject number PRJNA1040959. The genome assembly and annotation data are available at figshare (https://doi.org/10.6084/m9.figshare.24565225.v1).

Author contribution statement

Zhen-Yu Lyu: Conceptualization, Methodology, Visualization, Formal analysis, Writing - Original Draft, Writing - Review & Editing. **Shi-Kang Shen:** Conceptualization, Methodology, Writing - Original Draft, Writing - Review & Editing. **Si-Qi Wang:** Resources. **Xiong-Li Zhou:** Resources. **Rui Zhang:** Resources. **Gao-Ming Yang:** Cytological experiment. **Jie-Yu Zhang:** Cytological experiment. **Wen-Guang Sun:** Cytological experiment.

Funding

This study was supported by the Science and Technology Development Fund of Guidance from the Central Government to Locals in Yunnan Province (202207AB110016), Major Program for Basic Research Project of Yunnan Province (202101BC070002), National Natural

Science Foundation of China (31870529), the Graduate Scientific Research Fund Project of Yunnan University (KC-22221373), and Education Department of Yunnan, Scientific Research Fund Project (2024Y003).

Conflict of Interest

The authors declare that they have no competing interests.

References:

1. Fang R, Min TL. The floristic study on the genus *Rhododendron*. Acta Botanica Yunnanica. 1995;17:359–79.

2. Chen YS, Deng T, Zhou Z, Sun H. Is the East Asian flora ancient or not? Natl Sci Rev. 2018;5:920–32.

3. Basnett S, Rengaian G. A Comprehensive Review on the taxonomy, ecology, reproductive biology, economic importance and conservation status of Indian Himalayan *Rhododendrons*. Bot Rev. 2022;88:505–44.

4. Xia XM, Yang MQ, Li CL, Huang SX, Jin WT, Shen TT, Wang F, Li XH, Yoichi W, Zhang LH, Zheng YR, Wang XQ. Spatiotemporal Evolution of the Global Species Diversity of *Rhododendron*. Mol Biol Evol. 2022;39:msab314.

5. Darlington CD, Wylie AP. Chromosome atlas of flowering plants. George Allen and Unwin Ltd. Londonn, UK; 1955. p. 217–8.

6. Zhang J, Peng HW, Xia FC, Wang W. A comparison of seed plants' polyploids between the Qinghai-Tibet Plateau alpine and the Pan-Arctic regions. Biodiversity Science. 2021;29:1470–80.

7. Liu B, Zhao FM, Zhou H, Xia YP, Wang XY. Photoprotection conferring plant tolerance to freezing stress through rescuing photosystem in evergreen *Rhododendron*. Plant Cell Environ. 2022;45:2093–108.

8. Popescu R, Kopp B. The genus *Rhododendron*: An ethnopharmacological and toxicological review. J Ethnopharmacol. 2013;147:42–62.

9. Guo X, Dong Z, Li Q, Wan DG, Zhong JB, Dong D, Huang MZ. Flavonoids from *Rhododendron nivale* Hook. f delay aging via modulation of gut microbiota and glutathione metabolism. Phytomedicine. 2022;104:154270.

10. Hu YB, Wang XP, Xu YC, Yang H, Tong ZY, Tian R, Xu SH, Yu L, Guo YL, Shi P, et al. Molecular mechanisms of adaptive evolution in wild animals and plants. Sci China Life Sci. 2023;66:453–95.

11. Marks R. A, Hotaling S, Frandsen P. B, VanBuren R. Representation and participation across 20 years of plant genome sequencing. Nat. Plants. 2021;7:1571–8.

12. Zhang X, Kuang TH, Dong WL, Qian ZH, Zhang HJ, Landis JB, Feng T, Li LJ, Sun YX, Huang JL, et al. Genomic convergence underlying high-altitude adaptation in alpine plants. J Integr Plant Biol. 2023;65:1620–35.

13. De Storme N, Geelen D. The impact of environmental stress on male reproductive development in plant: Biologival processes and molecular mechanisms. Plant Cell Environ. 2014;37:1–18.

14. Chen HT, Zeng Y, Yang YZ, Huang LL, Tang BL, Zhang H, Hao F, Li W, Li YH, Liu YB, et al. Allele-aware chromosome-level genome assembly and efficient transgene-free genome editing for the autotetraploid cultivated alfalfa. Nat Commun. 2020;11:2494.

15. Wang F, Xia ZQ, Zou ML, Zhao L, Jiang SR, Zhou Y, Zhang CJ, Ma YZ, Bao YT, Sun HH, et al. The autotetraploid potato genome provides insights into highly heterozygous species. Plant Biotechnol J. 2022;20:1996–2005.

16. Zhang Q, Qi YY, Pan HR, Tang HB, Wang G, Hua XT, Wang YJ, Lin LY, Li Z, Li YH, et al. Genomic insights into the recent chromosome reduction of autopolyploid sugarcane *Saccharum spontaneum*. Nat Genet. 2022;54:885–96.

17. Zhang HY, He Q, Xing LS, Wang RY, Wang Y, Liu Y, Zhou QH, Li XZ, Jia Z, Liu Z, et al. The haplotype-resolved genome assembly of autotetraploid rhubarb *Rheum officinale* provides insights into the genome evolution and massive accumulation of anthraquinones. Plant Commun. 2023;26:100677.

18. Van de Peer, Y, Ashman TL, Soltis PS, Soltis DE. Polyploidy: an evolutionary and ecological force in stressful times. Plant Cell. 2021;33:11–26.

19. Stevens AV, Nicotra AB, Godfree RC, Guja LK. Polyploidy affects the seed, dormancy and seedling characteristics of a perennial grass, conferring an advantage in stressful climates. Plant Biol. 2020;22:500–13.

20. Liu CJ, Wang YG. Does one subgenome become dominant in the formation and evolution of a polyploid? Ann Bot. 2023;131:11–6.

21. Wang KL, Deng PR, Yao Z, Dong JY, He Z, Yang P, Liu YB. Biogeographic patterns of polyploid species for the angiosperm flora in China. J Syst Evol. 2022;61:776–89.

22. Heslop-Harrison JS, Schwarzacher T, Liu Q. Polyploidy: its consequences and enabling role in plant diversification and evolution. Ann. Bot. 2023;131:1–10.

23. Cifuentes M, Grandont L, Moore G, Chevre AM, Jenczewski E. Genetic regulation of meiosis in polyploid species: new insights into an old question. New Phytol. 2010;186:29–36.

24. Grandont L, Jenczewski E, Lloyd A. Meiosis and its deviations in polyploid plants. Cytogenet. Genome Res. 2013;140:171–84.

25. Bomblies, K. Learning to tango with four (or more): the molecular basis of adaptation to polyploid meiosis. Plant Reprod. 2022;36:107–24.

26. Gou XW, Bian Y, Zhang A, Zhang HK, Wang B, Lv RL, Li JZ, Zhu B, Gong L, Liu B. Transgenerationally precipitated meiotic chromosome instability fuels rapid karyotypic evolution and phenotypic diversity in an artificially constructed allotetraploid wheat (AADD). Mol, Biol, Evol. 2018;35:1078–91.

27. Huang G, Wu Z, Percy RG, Bai MZ, Li Y, Frelichowski JE, Hu J, Wang K, Yu JZ, Zhu YX. Genome sequence of *Gossypium herbaceum* and genome updates of *Gossypium arboreum* and *Gossypium hirsutum* provide insights into cotton A-genome evolution. Nat. Genet. 2020;52:516–24.

28. Zhang X, Pandey MK, Wang, JP, Zhao KK, Ma XL, Li ZF, Zhao K, Gong FP, Guo BZ, Varshney R, et al. Chromatin spatial organization of wild type and mutant peanuts reveals high-resolution genomic architecture and interaction alterations. Genome Biol. 2021;22:315.

29. Ranallo-Benavidez TR, Jaron KS, Schatz MC. GenomeScope 2.0 and Smudgeplot for reference-free profiling of polyploid genomes. Nat Commun. 2020;11:1432.

30. Miao Y, Luo D, Zhao T, Du H, Liu Z, Xu Z, Guo L, Chen C, Peng S, Li JX, et al. Genome sequencing reveals chromosome fusion and extensive expansion of genes related to secondary metabolism in Artemisia argyi. Plant Biotechnol J. 2022;20:1902–1915.

31. Song AP, Su JS, Wang HB, Zhang ZR, Zhang XT, van de Peer Y, Chen F, Fang WM, Guan ZY, Zhang F, et al. Analyses of a chromosome-scale genome assembly reveal the origin and evolution of cultivated chrysanthemum. Nat Commun. 2023;14:2021.

32. Xu J, Wang XY, Guo WZ. The cytochrome P450 superfamily: key players in plant development and

defense. J Integr Agric. 2015;14:1673–86.

33. Feng K, Hou XL, Xing GM, Liu JX, Duan AQ, Xu ZS, Li MY, Zhuang J, Xiong AS. Advances in AP2/ERF super-family transcription factors in plant. Crit Rev Biotechnol. 2020;40:750–76.

34. Abbas M, Sharma G, Dambire C, Marquez J, Alonso-Blanco C, Proano K, Holdsworth MJ. An oxygen-sensing mechanism for angiosperm adaptation to altitude. Nature. 2022;606:565–9.

35. Nie YQ, Guo LY, Cui FQ, Shen YR, Ye XX, Deng DY, Wang S, Zhu JH, Wu, WW. Innovations and stepwise evolution of CBFs/DREB1s and their regulatory networks in angiosperms. J Integr Plant Biol. 2022;64: 2111–25.

36. Durst F, Nelson DR. 1995. Diversity and evolution of plant P450 and P450-reductases. Drug Metabol Drug Interact 12:189-206.

37. Shen SK, Zhou XL, Wang SQ, Lyu ZY, Zhang R, Liu Y, Long B. Protect fragile mountaintop ecosystems. Science. 2023;380:1114–5.

38. Wu XP, Zhang L, Wang XY, Zhang RA, Jin GH, Hu YT, Yang H, Wu ZZ, Ma YP, Zhang CJ, Wang JH. Evolutionary history of two evergreen *Rhododendron* species as revealed by chromosome-level genome assembly. Front. Plant Sci. 2023;14:1123707.

39. Xu P, Xu J, Liu G, Chen L, Zhou ZX, Peng WZ, Jiang YL, Zhao ZX, Jia ZY, Sun YH et al. The allotetraploid origin and asymmetrical genome evolution of the common carp *Cyprinus carpio*. Nat Commun. 2019;10:4625.

40. Blumthaler M, Ambach W, R Ellinger. Increase in solar UV radiation with altitude. J Photochem Photobiol B. 1997;39:130–4.

41. Kerstiens G. Cuticular water permeability and its physiological significance. J Exp Bot. 199647:1813–32.

42. Tossi V, Lombardo C, Cassia R, Lamattina L. Nitric oxide and flavonoids are systemically induced by UV-B in maize leaves. Plant Sci. 2012;193:103–9.

43. Choi YE, Lim S, Kim HJ, Han JY, Lee MH, Yang Y, Kim JA, Kim YS. Tobacco *NtLTP1*, a glandular-specific lipid transfer protein, is required for lipid secretion from glandular trichomes. Plant J. 2012;70:480–

91.

44. Trivedi P, Nguyen N, Klavins L, Kviesis J, Heinonen E, Remes J, Jokipii-Lukkari S, Klavins M, Karppinen K, Jaakola L, Haggman H. Analysis of composition, morphology, and biosynthesis of cuticular wax in wild type bilberry (*Vaccinium myrtillus* L.) and its glossy mutant. Food Chem. 2021;354:12957.

45. Gao HN, Jiang H, Lian XY, Cui JY, You CX, Hao YJ, Li YY. Identification and functional analysis of the *MdLTPG* gene family in apple. Crit. Rev. Biotechnol. 2021;163:338–47.

46. Emiliani J, Grotewold E, Ferreyra MLF, Casati P. Flavonols protect *Arabidopsis* plants against UV-B deleterious effects. Mol Plant. 2013;6:1376–9.

47. McKenzie R, Conner B, Bodeker G. Increased summertime UV radiation in New Zealand in response to ozone loss. Science. 1999;285:1709–11.

48. Lahari T, Lazaro J, Schroeder DF. *RAD4* and *RAD23/HMR* Contribute to *Arabidopsis* UV Tolerance. Genes. 2018;9:8.

49. Rai N, O'Hara A, Farkas D, Safronov O, Ratanasopa K, Wang F, Lindfors AV, Jenkins GI, Lehto T, Salojärvi J, et al. 2020. The photoreceptor *UVR8* mediates the perception of both UV-B and UV-A wavelengths up to 350 nm of sunlight with responsivity moderated by cryptochromes. Plant Cell and Environment 43:1513-1527.

50. Mohl P, von Buren R. S, Hiltbrunner E. Growth of alpine grasslandwill start and stop earlier under climate warming. Nat Commun. 2022;13:7398.

51. Liu JM, de Vos JM, Körner C, Yang Y. 2023. Phylogeny and phenotypic adjustments drive functional traits in across elevations in its diversity hot-spot in W-China. Alpine Botany 133:69-84.

52. Li YH, Zheng LY, Corke F, Smith C, Bevan MW. Control of final seed and organ size by the *DA1* gene family in *Arabidopsis thaliana*. Genes Dev. 2008;22:1331–6.

53. Titapiwatanakun B, Blakeslee JJ, Bandyopadhyay A, Yang H, Mravec J, Sauer M, Cheng Y, Adamec J, Nagashima A, Geisler M, et al. *ABCB19/PGP19* stabilises *PIN1* in membrane microdomains in *Arabidopsis*. Plant J. 2009;57:27–44.

54. Gao, YS, Badejo AA, Sawa Y, Ishikawa T. Analysis of two l-Galactono-1,4-Lactone-Responsive genes

with complementary expression during the development of Arabidopsis thaliana. Plant Cell Physiol. 2012;53:592–601.

55. Sun H, Niu Y, Chen YS, Song B, Liu CQ, Peng DL, Chen JG, Yang Y. Survival and reproduction of plant species in the Qinghai-Tibet Plateau. J Syst Evol. 2014;52:378–96.

56. Apte CV. Barometric Pressure at High Altitude: Revisiting West's Prediction Equation, and More. High Alt Med Biol. 2023;24:85–93.

57. Li XT, Guo W, Li SH, Zhang JZ, Ni XN. The different impacts of the daytime and nighttime land surface temperatures on the alpine grassland phenology. Ecosphere. 2021;12:e03578.

58. Wu QB, Liu YZ. Ground temperature monitoring and its recent change in Qinghai-Tibet Plateau. Cold Reg Sci Technol. 2004;38:85–92.

59. Clouse SD, Sasse JM. Brassinosteroids: Essential regulators of plant growth and development. Annu Rev Plant Physiol, Plant Mol Biol. 1998;49:427–51.

60. Chaudhuri A, Halder K, Abdin MZ, Majee M, Datta A. Abiotic stress tolerance in plants: brassinosteroids navigate competently. Int J Mol Sci. 2022;23:14577.

61. Riechmann JL, Meyerowitz EM. 1998. The AP2/EREBP family of plant transcription factors. Biol Chem 379:633-646.

62. Thomashow MF. Plant cold acclimation: Freezing tolerance genes and regulatory mechanisms. Annu Rev Plant Physiol Plant Mol Biol. 1999;50:571–99.

63. Cao K, Zhang ZY, Fan H, Tan Y, Xu HW, Zhou XF. Comparative transcriptomic analysis reveals gene expression in response to cold stress in *Rhododendron aureum* Georgi. Theor Exp Plant Physiol. 2022;34:347–66.

64. Zhang QY, Li Y, Cao K, Xu HW, Zhou XF. Transcriptome and proteome depth analysis indicate ABA, MAPK cascade and Ca^{2+} signaling co-regulate cold tolerance in *Rhododendron chrysanthum* Pall. Front Plant Sci. 2023;14:1146663.

65. Staiger D, Brown JWS. Alternative splicing at the intersection of biological timing, development, and stress responses. Plant Cell. 2013;25:3640–56.

66. Mao HT, Chen MY, Su YQ, Wu N, Yuan M, Yuan S, Brestic M, Zivcak M, Zhang HY, Chen Y. Comparison on photosynthesis and antioxidant defense systems in wheat with different ploidy levels and octoploid Triticale. Int J Mol Sci. 2018;19:3006.

67. Parra-Nunez P, Fernández-Jiménez N, Pachon-Penalba M, Sanchez-Moran E, Pradillo M, Santos JL. 2024. Synthetically induced autotetraploids provide insights into the analysis of meiotic mutants with altered crossover frequency. New Phytologist 241:197-208.

68. Singliarova B, Hojsgaard D, Muller-Scharer H, Mraz P. The novel expression of clonality following whole-genome multiplication compensates for reduced fertility in natural autopolyploids. Proc Biol Sci. 2023;290:20230389.

69. Morgan C, Zhang HK, Henry CE, Franklin FCH, Bomblies K. Derived alleles of two axis proteins affect meiotic traits in autotetraploid *Arabidopsis arenosa*. Proc Natl Acad Sci USA. 2020;117:8980–8988.

70. Séguéla-Arnaud M, Choinard S, Larchevêque C, Girard C, Froger N, Crismani W, Mercier R. 2016. *RMI1* and *TOP3α* limit meiotic CO formation through their C-terminal domains. Nucleic Acids Research 45:1860-1871.

71. Séguéla-Arnaud M, Crismani W, Larchevêque C, Mazel J, Froger N, Choinard S, Lemhemdi A, Macaisne N, Van Leene J, Gevaert K, et al. 2015. Multiple mechanisms limit meiotic crossovers: TOP3alpha and two BLM homologs antagonize crossovers in parallel to FANCM. Proc Natl Acad Sci U S A 112:4713-4718.

72. Bazile J, Nadaud I, Lasserre-Zuber P, Kitt J, De Oliveira R, Choulet F, Sourdille P. 2024. *TaRECQ4* contributes to maintain both homologous and homoeologous recombination during wheat meiosis. Frontiers in Plant Science 14.

73. Marcais G, Kingsford C. A fast, lock-free approach for efficient parallel counting of occurrences of k-mers. Bioinformatics. 2011;27:764–70.

74. Cheng HY, Concepcion T, Feng XW, Zhang HW, Li H. Haplotype-resolved de novo assembly using phased assembly graphs with hifiasm. Nat Methods. 2021;18:170–5.

75. Koren S, Rhie A, Walenz BP, Dilthey AT, Bickhart DM, Kingan SB, Hiendleder S, Williams JL, Smith TPL, Phillippy AM. De novo assembly of haplotype-resolved genomes with trio binning. Nat Biotechnol. 2018;36:1174–82.

76. Nurk S, Walenz BP, Rhie A, Vollger MR, Logsdon GA, Grothe R, Miga KH, Eichler EE, Phillippy AM, Koren S. HiCanu: accurate assembly of segmental duplications, satellites, and allelic variants from high-fidelity long reads. Genome Res. 2020;30:1291–305.

77. Zhang XT, Zhang SC, Zhao Q, Ming R, Tang HB. Assembly of allele-aware, chromosomal-scale autopolyploid genomes based on Hi-C data. Nat Plants. 2019;5:833–845. doi:10.1038/s41477-019-0487-8.

78. Durand NC, Robinson JT, Shamim S, Machol I, Mesirov P, Lander ES, Aiden EL. Juicebox provides a visualization system for Hi-C contact maps with unlimited zoom. Cell Syst. 2016;3:99–101.

79. Simao FA, Waterhouse RM, Ioannidis P, Kriventseva EV, Zdobnov EM. BUSCO: assessing genome assembly and annotation completeness with single-copy orthologs. Bioinformatics. 2015;31:3210–2.

80. Rhie A, Walenz BP, Koren S, Phillippy AM. Merqury: reference-free quality, completeness, and phasing assessment for genome assemblies. Genome Biol. 2020;21:245.

81. Li KP, Xu P, Wang JP, Yi X, Jiao YN. Identification of errors in draft genome assemblies at single-nucleotide resolution for quality assessment and improvement. Nat Commun. 2023;14:6556.

82. Li H, Durbin R. Fast and accurate short read alignment with Burrows-Wheeler transform. Bioinformatics. 2009;25:1754–60.

83. Li H, Handsaker B, Wysoker A, Fennell T, Ruan J, Homer N, Marth G, Abecasis G, Durbin R. The Sequence Alignment/Map format and SAMtools. Bioinformatics, 2009;25:2078–9.

84. Ellinghaus D, Kurtz S, Willhoeft U. LTRharvest, an efficient and flexible software for de novo detection of LTR retrotransposons. BMC Bioinformatics. 2008;9:18.

85. Zhao X, Wang H. LTR_FINDER: an efficient tool for the prediction of full-length LTR retrotransposons. Nucleic Acids Res. 2007;35:W265–W268.

86. Ou SJ, Jiang N. LTR_retriever: a highly accurate and sensitive program for identification of long terminal repeat retrotransposons. Plant Physiol. 2018;176:1410–22.

87. Tarailo-Graovac M, Chen NS. Using RepeatMasker to identify repetitive elements in genomic sequences. Current protocols in bioinformatics. 2009. Chapter 4:4.10.1–4.10.14. doi:10.1002/0471250953.bi0410s25

88. Lowe TM, Eddy SR. tRNAscan-SE: A program for improved detection of transfer RNA genes in genomic

sequence. Nucleic Acids Res. 1997;25:955–64.

89. Lagesen K, Hallin P, Rodland EA, Staerfeldt HH, Rognes T, Ussery DW. RNAmmer: consistent and rapid annotation of ribosomal RNA genes. Nucleic Acids Res. 2007;35:3100–8.

90. Nawrocki EP, Burge SW, Bateman A, Daub J, Eberhardt RY, Eddy SR, Floden EW, Gardner PP, Jones TA, Tate J, et al. Rfam 12.0: updates to the RNA families database. Nucleic Acids Res. 2015;43:D130–D137.

91. Nawrocki EP, Eddy SR. Infernal 1.1: 100-fold faster RNA homology searches. Bioinformatics. 2013;29:2933–5.

92. Kim D, Paggi JM, Park C, Bennett C, Salzberg SL. Graph-based genome alignment and genotyping with HISAT2 and HISAT-genotype. Nat Biotechnol. 2019;37:907–15.

93. Haas BJ, Papanicolaou A, Yassour M, Grabherr M, Blood PD, Bowden J, Couger MB, Eccles D, Li B, Lieber M, et al. De novo transcript sequence reconstruction from RNA-seq using the Trinity platform for reference generation and analysis. Nat. Protoc. 2013;8:1494–512.

94. Pertea M, Pertea GM, Antonescu CM, Chang TC, Mendell JT, Salzberg SL. StringTie enables improved reconstruction of a transcriptome from RNA-seq reads. Nat Biotechnol. 2015;33:290–5.

95. Hoff KJ, Lange S, Lomsadze A, Borodovsky M, Stanke M. BRAKER1: Unsupervised RNA-Seq-Based fenome annotation with GeneMark-ET and AUGUSTUS. Bioinformatics. 2016;32:767–769.

96. Haas BJ, Delcher AL, Mount SM, Wortman JR, Smith RK, Hannick LI, Maiti R, Ronning CM, Rusch DB, Town CD, et al. Improving the *Arabidopsis* genome annotation using maximal transcript alignment assemblies. Nucleic Acids Res. 2003;31:5654–66.

97. Korf I. Gene finding in novel genomes. BMC Bioinformatics. 2004;5:59. doi:10.1186/1471-2105-5-59.

98. Blanco E, Genis P, Roderic G. Using geneid to identify genes. Current protocols in bioinformatics. 2007. 4:4.3.1–4.3.28.

99. Keilwagen J, Hartung F, Grau J. GeMoMa: Homology-Based gene prediction utilizing intron position conservation and RNA-seq data. Methods Mol Biol. 2019;1962:161–77.

100. Haas BJ, Salzberg SL, Zhu W, Pertea M, Allen J. E, Orvis J, White O, Buell CR, Wortman JR. Automated eukaryotic gene structure annotation using EVidenceModeler and the program to assemble spliced

alignments. Genome Biol. 2008;9:R7.

101. Huerta-Cepas J, Szklarczyk D, Heller D, Hernandez-Plaza A, Forslund SK, Cook H, Mende DR, Letunic I, Rattei T, Jensen LJ, et al. eggNOG 5.0: a hierarchical, functionally and phylogenetically annotated orthology resource based on 5090 organisms and 2502 viruses. Nucleic Acids Res. 2019;47:D309–D314.

102. McGinnis S, Madden TL. BLAST: at the core of a powerful and diverse set of sequence analysis tools. Nucleic Acids Res. 2004;32:W20–W25.

103. Tang H, Bowers JE, Wang X, Ming R, Alam M, Paterson AH. 2008. Synteny and collinearity in plant genomes. Science 320:486–488.

104. Nguyen LT, Schmidt HA, von Haeseler A, Minh BQ. IQ-TREE: A fast and effective stochastic algorithm for estimating maximum-likelihood phylogenies. Mol Biol Evol. 2015;32:268–74.

105. Emms DM, Kelly S. OrthoFinder: phylogenetic orthology inference for comparative genomics. Genome Biol. 2019;20:238.

106. Edgar RC. MUSCLE: multiple sequence alignment with high accuracy and high throughput. Nucleic Acids Res. 2004;32:1792–7.

107. Capella-Gutierrez S, Silla-Martinez JM, Gabaldon T. trimAl: a tool for automated alignment trimming in large-scale phylogenetic analyses. Bioinformatics. 2009;25:1972–3.

108. Yang ZH. PAML 4: Phylogenetic analysis by maximum likelihood. Mol. Biol. Evol. 2007;24:1586–91.

109. Ma YZ, Mao XX, Wang J, Zhang L, Jiang YZ, Geng YY, Ma T, Cai LM, Huang SQ, Hollingsworth P, et al. Pervasive hybridization during evolutionary radiation of *Rhododendron* subgenus *Hymenanthes* in mountains of southwest China. Natl. Sci. Rev. 2022;9:nwac276.

110. Mendes FK, Vanderpool D, Fulton B, Hahn MW. CAFE 5 models variation in evolutionary rates among gene families. Bioinformatics. 2020;36:5516–8.

111. Wu TZ, Hu EQ, Xu SB, Chen MJ, Guo PF, Dai ZH, Feng TZ, Zhou L, Tang WL, Zhan L, et al. clusterProfiler 4.0:A universal enrichment tool for interpreting omics data. Innovation. 2021;2:100141.

112. Wang YP, Tang HB, DeBarry JD, Tan X, Li JP, Wang XY, Lee TH, Jin HZ, Marler B, Guo H, et al. MCScanX: a toolkit for detection and evolutionary analysis of gene synteny and collinearity. Nucleic Acids

Res. 2012;40:e49.

113. Wang Dapeng, Zhang YB, Zhang Z, Zhu J, Yu J. KaKs_Calculator 2.0: a toolkit incorporating gamma-series methods and sliding window strategies. Genom Proteom Bioinf. 2010;8:77–80.

114. Zhang Z, Xiao JF, Wu JY, Zhang HY, Liu GM, Wang XM, Dai L. ParaAT: A parallel tool for constructing multiple protein-coding DNA alignments. Biochem Biophys Res Commun. 2012;419:779–881.

115. Yang FS, Nie S, Liu, H, Shi TL, Tian XC, Zhou SS, Bao YT, Jia KH, Gou JF, Zhao W, et al. Chromosome-level genome assembly of a parent species of widely cultivated azaleas. Nat Commun. 2020;11:5269.

116. Pond SLK, Frost SDW, Muse SV. HyPhy: hypothesis testing using phylogenies. Bioinformatics. 2005;21:676–9. doi:10.1093/bioinformatics/bti079.

117. Smith MD, Wertheim JO, Weaver S, Murrell B, Scheffler K, Pond SLK. Less Is More: An Adaptive Branch-Site Random Effects Model for Efficient Detection of Episodic Diversifying Selection. Mol Biol Evol. 2015;32:1342–53.

118. Murrell B, Wertheim JO, Moola S, Weighill T, Scheffler K, Pond SLK. 2012. Detecting Individual Sites Subject to Episodic Diversifying Selection. PLoS Genet;8:e1002764..

119. Pond SLK, Wisotsky SR, Escalante A, Magalis BR, Weaver S. Contrast-FEL-A Test for Differences in Selective Pressures at Individual Sites among Clades and Sets of Branches. Mol Biol Evol. 2021;38:1184–98.

120. Dobin A, Davis CA, Schlesinger F, Drenkow J, Zaleski C, Jha S, Batut P, Chaisson M, Gingeras TR. STAR: ultrafast universal RNA-seq aligner. Bioinformatics. 2013;29:15–21.

121. Li B, Dewey CN. RSEM: accurate transcript quantification from RNA-Seq data with or without a reference genome. BMC Bioinformatics. 2011;12:323.

122. Zhang JS, Zhang XT, Tang HB, Zhang Q, Hua XT, Ma XK, Zhu F, Jones T, Zhu XG, Bowers J, et al. Allele-defined genome of the autopolyploid sugarcane *Saccharum spontaneum* L. Nat Genet. 2018;50:1565–73.

123. Qiao X, Li QH, Yin H, Qi KJ, Li LT, Wang RZ, Zhang SL, Paterson AH. Gene duplication and evolution

in recurring polyploidization-diploidization cycles in plants. Genome Biol. 2019;20:38.

124. Katoh K, Standley DM. MAFFT multiple sequence alignment software version 7: improvements in performance and usability. Mol Biol Evol. 2013;30:772–80.

125. Price MN, Dehal PS, Arkin AP. 2010. FastTree 2--approximately maximum-likelihood trees for large alignments. Plos One 5:e9490.

126. Chen CJ, Chen H, Zhang Y, Thomas HR, Frank MH, He YH, Xia R. TBtools: an integrative toolkit developed for interactive analyses of big biological data. Mol Plant. 2020;13:1194–202.

127. Librado P, Vieira FG, Rozas J. BadiRate: estimating family turnover rates by likelihood-based methods. Bioinformatics. 2012;28:279–81.

Figure titles

Figure 1. Habitat and genomic characteristics of *R. nivale* subsp. *boreale*. A. habitat; B. habit; C. genome landscape, a, 52 pseudochromosomes, which belong to 13 homologous groups, and the length of the pseudochromosome; b, gene density; c, GC density; d, transposon element density; e, copia density; f, gypsy density; g, tandem repeat density; curved lines inside the circles link syntenic genes between different pseudochromosomes, the synteny between haplotype 1 and haplotype 2 is indicated in red, the synteny between haplotype 1 and haplotype 3 is indicated in green, the synteny between haplotype 1 and haplotype 4 is indicated in yellow. D. Hi-C heatmap for assembled pseudochromosomes; E. Smudgeplot analysis based on 21 *k-mers*.

Figure 2. Phylogenetic and comparative analysis between related species and haplotypes. A. Dot plot between *R. nivale* subsp. *boreale* and *R. ovatum*. B. Syntenic blocks between four haplotypes. C. phylogenetic relationships of Subsect. *Lapponica* based on the maximum likelihood (ML) analysis; yellow and green blocks show *R. nivale* and the sister clade of *R. nivale*, respectively; red block represents the data generated in this study (n1, n2, n3, n4, and *R. nivale* subsp. *boreale* represent the four haplotypes and transcriptome of *R. nivale* subsp. *Boreale,* respectively) and the blue block represents downloaded species data. D. Gene family characteristics between four haplotypes.

Figure 3. Comparative genomic analysis. A. ML phylogenetic tree showing the relationship between *R. nivale* subsp. *boreale* and 18 other species. Estimated divergence times (Mya, million years ago) are labeled at nodes in black. Bootstrap values are displayed on the nodes in circles (100%) and squares (≥ 95%). Expansion (orange) and contraction (blue) of gene families are shown on the branch, contraction and expansion of ancestors are represented by a pie chart, and extant species are indicated by numbers. WGD and WGT events are marked with D and T, respectively. B. A number of other orthologs, unique paralogs, multicopy orthologs and single-copy orthologs in 19 species. C. *Ks* of paralogs frequency distribution chart of seven species, namely, six Ericales (*Actinidia*, *Vaccinium* and Subg. *Hymenanthes*, Subg. *Furthermore*, Subg. *Rhododendron*, Subg. *Tsutsusi* one species each) and one *Vi. vinifera*, polyploidization events are represented by dotted lines. D. Homologous gene dot plots between *R. nivale* subsp. *boreale* and *Vi. vinifera*. The red box exemplifies the orthologous ratio of 1:2 between *Vi. vinifera* and *R. nivale* subsp. *boreale*.

Figure 4. KEGG and GO enrichment and gene duplication analysis of *R. nivale* subsp. *boreale*. A. KEGG (left) and GO (right) enrichment of genes in significantly expanded gene families. B. Venn diagram showing the number of shared and specific gene duplications between the significantly expanded genes (SEGs) and five categories of duplications (DSD, dispersed duplications; PD, proximal duplications; TD, tandem duplications; TRD, transposed duplications; WGD, whole genome duplications). C. *Ka/Ks* ratios of the five types of duplications. D. KEGG pathway enrichment analysis of the five duplication types.

Figure 5. Single-matched allelic expression analysis. A. The total amount of single-match allelic expression of 52 pseudochromosomes; the colors represent 13 homologous groups (HGs). B. Heatmap clustering analysis of single-match alleles in screening the position of DELs in *R. nivale* subsp. *boreale*. Each row represents a set of differentially expressed alleles, and each column represents a chromosome. The heatmap shows a homologous group.

Figure 6. Identification and evolution of key family and genes for adaptation to low mountaintop temperature and hypoxia. A. Rootless ML phylogenetic tree based on ultrafast 1,000 bootstrap samplings showed diversified AP2/ERF superfamily in 13 species, including 10 *Rhododendron* species and *Ar. thaliana*, Kiwifruit, *Va. darrowii.* The color of the clades indicates five subfamilies of the AP2/ERF superfamily. The labels are differently colored according to species. B. Schematic diagram of the gain and loss of key genes in 12 species of Ericales; numbers in pink and blue depict *ERF VII* and *CBF* gene family turnover. The numbers in the rectangles and circles represent the number of genes in ancestral and existing species. The + and − signs represent the gain and loss of genes, respectively. C. Expression levels of *ERF VIIs* and *CBFs*. The numbers in parentheses indicate the number of genes that are expressed. P represents the adjusted P value.

Figure 7. Characteristics of Ericales Cytochrome P450 (CYP). A. ML phylogenetic tree showing the relationship between 10 CYP clans (higher order groupings of CYP families). B. Heatmap showing the number of clan members for each species. C. Phylogenetic tree of the CYP members of 13 species based on GTR (generalized time-reversible). Different clans are represented by different colors. D. Number of CYP genes produced by duplication events in 10 species of *Rhododendron*.

Manuscript

Click here to access/download;Manuscript;manuscript_without_revised_trac

Title: The first high-altitude autotetraploid haplotype-resolved genome assembled (*Rhododendron nivale* subsp. *boreale*) provides new insights into mountaintop adaptation

Authors: Zhen-Yu Lyu[1], Xiong-Li Zhou[1], Si-Qi Wang[1], Gao-Ming Yang[1], Wen-Guang Sun[2], Jie-Yu Zhang[2], Rui Zhang[1], Shi-Kang Shen[1*]

Affiliation:

[1]Ministry of Education Key Laboratory for Transboundary Ecosecurity of Southwest China, Yunnan Key Laboratory of Plant Reproductive Adaptation and Evolutionary Ecology, Institute of Biodiversity, School of Ecology and Environmental Science, Yunnan University, Kunming, 650504, Yunnan, China

[2]School of Life Sciences, Yunnan Normal University, Kunming, 650500, Yunnan, China

Corresponding authors: *Shi-Kang Shen, E-mail: ssk168@ynu.edu.cn; (ORCID: 0000-0002-0611-6763)

Telephone: +86-871-65933510; Fax: +86-871-65933510;

Postal address for corresponding authors: School of Ecology and Environmental Sciences, Yunnan University, No.2 Green Lake North road Kunming, Yunnan, 650091, China

Words: 7383 (excluding references)

Figures: 7 color figures.

Abstract

Background

Rhododendron nivale subsp. *boreale* Philipson et M. N. Philipson is an alpine woody species with ornamental qualities that serve as the predominant species in mountainous scrub habitats found at an altitude of ~4200 m. As a high-altitude woody polyploid, this species plays a distinct role in the adaptability of alpine plants. Despite its ecological significance, the lack of genomic resources has hindered a comprehensive understanding of its evolutionary and adaptive characteristics in high-altitude mountainous environments.

Findings

We sequenced and assembled the genome of *R. nivale* subsp. *boreale*, an assembly of the first subgenus *Rhododendron* and the first high-altitude woody flowering tetraploid, contributing an important genomic resource for alpine woody flora. The assembly included 52 pseudochromosomes (scaffold N50=42.93 Mb; BUSCO=98.8%; QV=45.51; S-AQI=98.69), which belonged to 4 haplotypes, harboring 127,810 predicted protein-coding genes. Conjoint *k-mer* analysis, collinearity assessment, and phylogenetic investigation corroborated autotetraploid identity. Comparative genomic analysis revealed that *R. nivale* subsp. *boreale* originated as a neopolyploid of *R. nivale* and underwent two rounds of ancient polyploidy events. Transcriptional expression analysis showed that differences in expression between alleles were common and randomly distributed in the genome. We identified extended gene families and signatures of positive selection that are involved not only in adaptation to the mountaintop ecosystem (response to stress and developmental regulation) but also in autotetraploid reproduction (meiotic stabilization). Additionally, the expression levels of the *ERF VIIs* were significantly higher than the mean global gene expression. We suspect that these

changes have enabled the success of this species at high altitudes.

Conclusions

We assembled the first high-altitude autopolyploid genome and achieved chromosome-level assembly within the subgenus *Rhododendron*. In addition, a high-altitude adaptation strategy of *R. nivale* subsp. *boreale* was reasonbly speculated. This study provides valuable data for the exploration of alpine mountaintop adaptations and the correlation between extreme environments and species polyploidization.

Key words: Autotetraploid, Evolutionary history, Harsh environment, Mountaintop adaptation, *Rhododendron*,

1. Context

Rhododendron L. is the largest genus in Ericaceae and the largest woody plant genus in the Northern Hemisphere, with more than 1,000 species. It is also representative of the highly diverse Sino-Himalayan Flora in East Asia, shaped by the topographic and climatic heterogeneity resulting from the uplift of the Qinghai-Tibet Plateau [1,2]. Furthermore, *Rhododendron* is one of the few woody flowering species that is dominant in plant communities found within the delicate subalpine to alpine transition zone and presents a perfect opportunity to explore the mechanisms behind the evolution and adaptation of alpine woody plants [3,4]. In *Rhododendron*, *R. nivale* subsp. *boreale* Philipson et M. N. Philipson is one of the few woody flowering plants discovered to be distributed at altitudes above 5000 m and is one of the few polyploid (2n=4x=52) woody plants in the Qinghai-Tibet Plateau [1,5,6]. *R. nivale* subsp. *boreale*, a member of the subg. *Rhododendron*, is a small-leaved, highly branched shrub distributed at high altitudes of mountaintops (up to alt. 5400 m) down to the mountainsides (~3200 m). This species demonstrates remarkable adaptability, as evidenced by its diverse habitats, including alpine meadows, forest edges, and metal mining areas [7]. Currently, *R. nivale* subsp. *boreale* is an important ornamental plant resource in mountainous plateau areas and is used in traditional Tibetan medicine [8,9]. Therefore, exploring the evolutionary patterns and adaptation mechanisms of *R. nivale* subsp. *boreale* not only promotes the understanding of alpine adaptation evolution in woody plants, but also establishes a basis for the commercial exploitation of high-altitude ornamental plants.

Genetic perspectives provide a better understanding of evolution and adaptive differentiation [10]. However, the paucity of genomic data is a significant impediment to

research advancement [11]. For example, in a recent high-altitude adaptation study, only seven alpine plant genomes were used, indicating that the genetic resources of alpine plants are far from sufficient compared to the diversity of the high-altitude flora [12]. Moreover, polyploidy likely enhanced the adaptability of alpine plants to harsh environments [13]. Unfortunately, acquiring polyploid genetic data remains challenging, particularly for autopolyploids with highly similar subgenomes. Currently, the assembly of autopolyploid genomes presents significant challenges, resulting in the publication of only a select number of such genomes, including those of *Medicago sativa*, *Saccharum spontaneum*, *Solanum tuberosum*, and *Rheum officinale* [14,15,16,17]. To further understand the evolution and adaptation of the alpine flora, additional genetic resources, particularly of polyploids, are essential.

Polyploidy has been theorized to be both a potential evolutionary roadblock and a catalyst for evolutionary breakthroughs and the proliferation of species [18]. On the one hand, following polyploidy events, rapid shifts in gene expression and epigenetic modifications can bestow the polyploid with an almost instant competitive edge, which is usually reflected in their broader geographical ranges compared with their diploid ancestors [19,20]. Therefore, polyploidy tends to be ecologically advantageous and occurs in variable climatic regions, such as the Qinghai-Tibet Plateau alpine and Pan-Arctic regions [21,22]. Comprehending these adaptive mechanisms in high-altitude polyploids not only clarifies evolutionary dynamics but also provides insights into conservation strategies. On the other hand, auto and allopolyploids face a significant obstacle: the accurate segregation of chromosomes during meiosis [23,24]. In recent years, our understanding of the molecular basis for polyploid adaptations to meiotic challenges has significantly increased; however, compared to allotetraploids, little is known

about the molecular mechanisms underlying the stabilization of autotetraploid meiosis [25]. Advances in molecular technology and subsequently, more genetic resources will provide new insights into the survival, evolution, adaptation, and conservation of polyploids.

Here, we present a haplotype-resolved tetraploid genome of mountaintop plant *R. nivale* subsp. *boreale* from an altitude of 4,287.5 m, which is the first chromosome-level genome assembly of the subgenus *Rhododendron*. Based on this assembly, we identified polyploid types, deciphered whole genome duplication (WGD) events, and investigated which genes or gene families are potential candidates involved in alpine mountaintop adaptation and the survival of polyploids. This genome not only establishes the groundwork for comprehending the evolution and adaptation of *Rhododendron* species, but also offers valuable genetic resources to investigate the origin, recombination, and differentiation of polyploid species.

2. Results

2.1 Genome estimation, sequencing, and assembly

R. nivale subsp. *boreale* samples were collected from the mountaintop, treated with liquid nitrogen, and sequenced (Fig. 1A). We obtained a total of 33.35 gigabases (Gb) of PacBio CCS long reads with an average length of 15.86 kb and an N50 length of 16.15 kb (Table S1). A genome survey was performed based on DNBseq short reads (95.61 Gb; Table S2), and the result revealed an estimated genome size of 2.48 Gb, which was consistent with that estimated by flow cytometry (Fig. S1, S2; Table S3). This species was identified as a tetraploid based on *k-mers* analysis (Fig. 1E). The three initial assembly sizes assembled using Hifiasm, Canu v1.9, and Hicanu were 2.48 Gb, 2.39 Gb, and 2.40 Gb, respectively (Table S4; S8). The assembled

version of Hifiasm was used for subsequent analysis, as it ensures higher integrity of both genes and long terminal repeats (LTRs). The long reads and NGS reads were mapped to the unitig-level assembly to assess the assembly quality. Long-read and whole-genome sequencing (WGS) reads were mapped to 99.87% and 99.59%, respectively, and RNA-seq reads exhibited mapping rates exceeding 94% (Table S5). The average GC content was 41.10%. We used the AllHiC algorithm to improve the genome assembly to the chromosome level using 138.98 Gb of Hi-C data. After manual checking, a total of 2.17 Gb of unitigs were anchored to 52 pseudochromosomes (scaffold N50 = 42.93 Mb), ranging from 23.70 to 59.70 megabase (Mb) in length, and containing four haplotypes (13 pseudochromosomes per haplotype) (Fig. 1C; Table S6). The Hi-C heatmap clearly showed the interactions of 13 homologous groups (Fig. 1D), with high similarity observed between the pseudochromosomes within each homologous group.

This genome was assembled with a high consensus quality value (QV = 45.51; error rate = 0.0028%) and high *k-mer* completeness (97.79%) (Table S7). BUSCO assessment indicated that the completeness of the conserved embryophyte genes was 98.8 % (Table S8). The quality of the genome structure at the reference genome level (assembly quality indicators of large structural fragments; S-AQI = 98.69) was assessed using CRAQ (Table S9).

2.2 Annotation

A repeat sequence of 1,549,068,457 bp was identified, accounting for 62.63% of the genome assembly (Table S10). The richest category of repeats was LTRs (43.12%), with *Gypsy* and *Copia* accounting for 33.66% and 5.88% of the repeats, respectively (Fig. S3). In addition, the LTR assembly index (LAI) was greater than 14 (n1:14.78, n2:14.84, n3:14.35, and n4:14.38)

based on LTR annotation, which indicated that the assembly met the reference category standards. By combining ab initio, homology, and transcriptome data predictions, 127,810 protein-coding genes were predicted, with an average gene length of 4,736.07 bp. The total length of the coding sequences (CDS) was 148,274,600 bp, and the average number of CDSs per gene was 4.8 (Table S11). The completeness of 98.6% of the annotated protein-coding genes of *R. nivale* subsp. *boreale* was assessed using BUSCO. Of the protein-coding genes, 96.86% were annotated functionally (Table S12). The ratio of mono-exonic (single-exon) to multi-exonic (multiple-exon) was 0.245. We annotated 17,049 candidate noncoding RNAs, including 703 microRNAs (miRNAs), 3,672 transfer RNAs (tRNAs), 5,373 small nuclear RNAs (snRNAs), and 7,301 ribosomal RNAs (rRNAs) (Table S13).

2.3 Confirmation of Autotetraploid

Polyploids are commonly found in plants. However, the origins of the polyploids differ and include both homologous and heterologous origins. Extensively studied allotetraploids such as peanuts, cotton, and wheat [26,27,28], exhibit significant subgenomic differences, allowing for their division into distinct subgenomes. In contrast to allotetraploids, the high similarity among haplotypes greatly increases the difficulty in assembling autotetraploids. To determine the polyploid type of *R. nivale* subsp. *boreale*, we employed *k-mer* analysis, collinearity analysis, and phylogenetic analysis for cross-validation. The 21 *k-mer* frequency analysis revealed four distinct peaks (located at 33, 68, 106, and 136) (Fig. S2), which was highly similar to the results for autotetraploids (*Medicago sativa* and *Saccharum spontaneum*). Nucleotide heterozygosity is an important criterion for determining polyploid types [29]. Nucleotide heterozygosity form analysis of *R. nivale* subsp. *boreale* showed 2.53% AAAB and 1.28%

AABB (Table S3), which was consistent with the expectation that the heterozygous rate of autotetraploid AAAB would be greater than that of AABB.

Nevertheless, these methods were insufficient to identify the polyploid type. For example, although genomic analysis indicated higher AAAB than AABB, *Artemisia argyi* was identified as an allotetraploid [30]. To further determine the polyploid type of *R. nivale* subsp. *boreale*, synteny analysis was performed based on syntenic blocks. As expected, the dot plot and syntenic blocks indicated synteny among the four haplotypes (Fig. 2A), with 20,172 gene pairs showing synteny between haplotypes 1 and 2, 20,249 between haplotypes 2 and 3, and 19,883 between haplotypes 3 and 4 (Fig. 2B, S4). Additionally, we downloaded transcriptome data from 11 samples of seven closely related species (Table S14) to infer the phylogenetic positions of the four haplotypes of *R. nivale* subsp. *boreale* and identified a monophyletic group consisting of six species (*R. nitidulum*, *R. hippophaeoides*, *R. thymifolium*, *R. nivale*, *R. nivale* subsp. *Boreale,* and *R. lapponicum*) of subsect. *Lapponica* with high support. A clade containing all *R. nivale* subsp. *boreale* (including the transcriptome and four haplotypes) and two *R. nivale* was supported by 100% bootstrapping (Fig. 2 C).

Moreover, 25,791 orthogroups were identified in the four haplotypes, with 16,403 shared by all, 5,242 shared by three, 3,631 shared by two, and only 515 (n1:134; n2:139; n3:135; n4:107) unique to each haplotype genome, showing high genetic similarity among haplotypes (Fig. 2D). Overall, the combined results of *k-mer* analysis, collinearity analysis, and phylogenetic analysis indicated that *R. nivale* subsp. *boreale* is an autotetraploid species.

2.4 Comparative analysis and recent polyploidization

The phylogenetic position and divergence times of *R. nivale* subsp. *boreale* were inferred

from 18 other species, including 12 species of Ericales (10 *Rhododendron*), two species of Cornales, one species of Gentianales, one species of Vitales, two species of monocotyledons, and one sister species to all angiosperms (*Amborella*). Altogether, 666,442 genes were used to infer orthology. A total of 625,661 genes (93.9%) clustered into 37,844 orthologous gene families, of which 6,547 were shared across all species (Fig. 2B; Table S15). In total, 209 single-copy gene families were identified. In total, 146 gene families, comprising 369 genes, were found to be specific to *R. nivale* subsp. *boreale* (Fig. S5). These species-specific genes were enriched in 12 Kyoto Encyclopedia of Genes and Genomes (KEGG) pathways and 136 gene ontology (GO) terms including arginine biosynthesis, nitrogen metabolism, and flavonoid biosynthesis (Tables S16 and S17).

In total, 209 single-copy orthologous genes were used to reconstruct phylogenetic relationships using IQ-TREE. All nodes were supported by high bootstrap values (> 95%). The results supported that *Rhododendron* is a monophyletic group, and the 10 species of *Rhododendron* were divided into four clades representing four subgenera (*Tsutsusi*, *Rhododendron*, *Pentanthera,* and *Hymenanthes*) (Fig. 3A). The time tree inferred from the MCMCtree suggested that the ancestor of *Rhododendron* separated from the common ancestor of *Rhododendron* and *Va. darrowii* approximately 41.2 Mya. The split between *R. nivale* subsp. *boreale* and the sister groups *(R. mole, R. henenense, R. delavayi, R. griersonianum, and R. irroratum*) was 30.4 Mya, while the divergence time of *R. molle* was 28.3 Mya (Fig. 3A).

The synonymous substitution rate (*Ks*) of orthologs and paralogs of seven species (four *Rhododendron* species, one *Vaccinium*, one *Actinidia* species, and one *Vitis*) was calculated to determine the WGD events that occurred in *R. nivale* subsp. *boreale*. Polyploidy analysis

indicated that *Rhododendron* and *Va. darrowii* experienced two rounds of ancient polyploidy events, whereas *Ac. chinensis* experienced three. Similar peaks were observed for all four *Rhododendron* species and *Va. darrowii*. And the farthest peak revealed an ancient γ whole-genome triplication (WGT-γ) event common to *Rhododendron* and other core eudicots, which was inferred to have occurred 122–164 Mya from a previous study [31]. In addition, the peak at *Ks* of the paralogs approximately 0.65 Mya suggests another polyploidization event in *Rhododendron*, *Va. darrowii,* and *Ac. chinensis* estimated to have occurred at approximately 78 Mya (Fig. 3C, S9). The dot plot comparing *Vi. vinifera* and *R. nivale* subsp. *boreale* (Fig. 3D, S7–8), nearly every grape chromosome exhibited two highly compatible chromosomal regions in *R. nivale* subsp. *boreale* (orthologous ratio 1:2) (Fig. 3D).

2.5 Analysis of positive selection

Using multiple models, we expected to provide more accurate detection of the selection signals of *R. nivale* subsp. *boreale*. First, adaptive Branch-Site Random Effects Likelihood (aBSREL) was used to test for positive selection in the high-altitude branch of each gene by examining 28 positively selected genes (PSGs). Subsequently, we employed clade model C (CmC) to further evaluate foreground and background selection differences compared with the null model M2a_rel to exclude genes not affected by selection pressure (Table. S18). Sixteen genes were identified as PSGs (Table. S19). Positive selection sites were detected using the Mixed Effects Model of Evolution (MEME) and the Contrast-FEL, with the number of positive selection sites per gene ranging from 0 to 19. These two genes exhibit no significant positive selection sites. In addition, 19 genes were identified as PSGs using KaKs_Calculator and were functionally annotated. (Table. S20). These PSGs are associated with various

biological processes, including meiosis recombination (*TOP3α*), nucleotide excision repair (*UVR8*, *RAD23B*), leaf surface wax metabolism (*LTG30*, *LTP1*), auxin transporter (*ABCB19*), signal transduction and regulation (*M3K1*, *CAGC1*), and biological clock regulation (*ESD4*). Additionally, we explored the selection pressure within 13 homologous groups where similar pressures were observed. (Fig. S6).

2.6 Gene duplication and family evolution

Based on the ultrametric tree, the gene family evolution of 19 species was compared with that of the most recent common ancestor (MRCA). Overall, 3,356 orthogroups expanded in *R. nivale* subsp. *boreale*, while only 756 orthogroups contracted. Among these, 375 and 79 orthogroups expanded and contracted significantly, respectively. GO and KEGG enrichment analyses suggested that the significantly expanded orthogroups were primarily enriched in pathways such as brassinosteroid (BR) biosynthesis, terpenoid biosynthesis, and isoflavonoid biosynthesis (Fig. 4A).

To explore the connection between gene duplication and gene family expansion, 20,673 duplicated genes were identified and classified into five categories: 3,899 whole-genome duplication (WGD; 18.86%), 2,711 transposed duplication (TRD; 13.11%), 3,744 tandem duplication (TD; 18.11%), 4,738 proximal duplications (PD; 22.92%), and 5,581 dispersed duplication (DSD; 27.00%) duplications (Fig. 4B). Among these, PD and TD contributed the most to the expansion of gene families. Moreover, ω (*Ka/Ks*) ratios of all duplication categories were calculated, revealing that PD and TD demonstrated superior ω scores compared to other types, while the lowest ω score was for WGD (Fig. 4C). KEGG functional enrichment analysis indicated that the functions of genes shared by the significantly expanded

orthogroups and five different duplication types were differentiated. WGD genes were enriched in plant hormone signal transduction and nucleotide excision repair, TRD duplications were implicated in plant-pathogen interactions and *O*-glycan biosynthesis, gene family expansions related to arachidonic acid metabolism and linoleic acid metabolism were mainly contributed by DSD, and duplications of TD and PD were associated with BR biosynthesis, cytochrome P450, and isoflavonoid biosynthesis (Fig. 4D; Table S21–S30).

2.7 Expression of alleles

Transcriptome data from the roots, stems, leaves, and buds of *R. nivale* subsp. *boreale* were used to explore allelic expression patterns. Overall, 77,892 genes, representing 60.94% of all the genes, were expressed in at least one tissue. In single matching four alleles (1:1:1:1), 12,642 out of the 14,550 alleles were expressed in at least one allele. The expression levels of chromosomes within each homologous group were similar, with homologous group 3 showing a higher transcript expression than the other groups (Fig. 5A). Homologous Group 10 had the lowest expression level. We selected genes with a transcripts per kilobase per million mapped reads (TPM) value ≥ 1 to compare the differences in expression levels between haplotypes, and 6,388 single-match gene groups were identified. Finally, 3,844 of the 6,388 (60.17%) single-match gene groups were identified as differentially expressed loci (DELs), with DEL ratios ranging from 56.14% to 63.93% per pseudochromosome. The DELs were randomly distributed across the genome (Fig. 5B).

2.8 Evolution of the APETALA2/ethylene responsive factor (AP2/ERF)

Plant APETALA2/ethylene responsive factors (AP2/ERFs) and cytochrome P450s (CYPs), which likely play important roles in the adaptation of plants to high altitudes, participate in a

multitude of biochemical pathways and fulfill various functions in the realms of growth and protection, including responses to UV irradiation, dehydration, and pathogens [32,33]. Therefore, AP2/ERF and CYP families were explored. We identified the AP2/ERF family in 10 *Rhododendron* and two related species (*Actinidia chinensis* and *Vaccinium darrowii*) using the HMMer method. In total, 2,397 genes were identified as belonging to the AP2/ERF family in 12 species after artificially confirming the presence of the AP2 domain (Table S31). For convenience, haplotypes were extracted from the haplotype-resolved assemblies of *R. nivale* subsp. *boreale* and *R. vialii* to compare the gene counts within the family. Among *Rhododendron* species, *R. ovatum* exhibited the highest gene count (163), while *R. irroratum* contained only 88 genes. The majority of species in our study had gene counts of approximately 140. These genes were randomly distributed across 13 pseudochromosomes (Fig. S10-21). To further understand the phylogenetic mechanisms of the AP2/ERF family in *Rhododendron*, proteins of *Ar. thaliana* and other 12 species were used to construct the phylogenetic tree. Consistent with previous research, 13 categories, including the AP2, ERF (B-1 to B-6), DREB (A-1 to A-6), RAV, and soloist subfamilies, were identified (Fig. 6A). Compared with *Ar. thaliana*, the categorization of B-3 was expanded in *Rhododendron*.

The alpine environment is variable, with significant temperature differences between day and night, strong ultraviolet radiation, and low oxygen partial pressure. Group VII ethylene response factor transcription factors (ERF VIIs) are associated with altitude adaptation [34]. Hence, based on the homology of *ERF VIIs* in *Ar. thaliana*, we identified *ERF VIIs* in *Rhododendron*. Ten species of *Rhododendron* contained one (*R. molle*) to five (*R. simsii*) *ERF VIIs* (Table S32). The phylogenetic tree topology showed that *ERF VIIs* were divided into four

groups. *ERF VIIs* of *Ar. thaliana* genes were found in Groups I, III, and IV (Fig. S22). Group II consisted solely of Ericales genes, and one clade contained the alpine species of *Rhododendron*. Based on motif analysis, similar gene structures within the groups showed phylogenetic reliability (Fig. S23). Interestingly, we estimated the turnover of *ERF VII* subfamilies using the maximum likelihood method and found a continuous decrease in the number of genes (Fig. 6B). However, among the 13 *ERF VII* genes, 12 were expressed in the roots, stems, leaves, and buds of *R. nivale* subsp. *boreale* and exhibited significantly higher expression levels than the global gene expression (Fig. 6C).

C-repeat binding factors/dehydration-responsive element binding protein 1 (*CBFs/DREB1s*) play a crucial role as transcription factors that regulate gene expression during cold acclimation. Consistent with previous studies, *CBFs* were categorized as A-1 of the dehydration-responsive element binding (DREB) subfamily, which is separated from A-4 of DREB [35]. We used the BLASTP program and two conserved sequences, PKRxAGRxKFxETRHPV and DSAWR, surrounding the AP2/ERF domain to accurately identify *CBF* genes. Sequence comparison with *Ar. thaliana* revealed that *CBFs* have a highly conserved domain. A total of 49 *CBFs* were identified from 10 *Rhododendron* species, of which *R. nivale* subsp. *boreale* contained 16 *CBFs* (12 *CBF* genes with four alleles and 2 *CBF* genes with two alleles) (Table S32). Phylogenetic analysis indicated that *CBFs* of *Rhododendrons* could be divided into three groups (Fig. S24). Similar conserved motifs were observed in each group, indicating the reliability of the relationship of *CBFs* (Fig. S25). In addition, family turnover based on BadiRate was re-validated, and *CBFs* were continuously lost without gain, resulting in the contraction of *CBF* genes (Fig. 6B). Approximately only half

(9/16) of the *CBFs* were expressed (Fig. 6C).

2.9 Evolution of the CYP family

Using the *R. nivale* subsp. *boreale* genome we assembled, along with the genomes of 12 other closely related species, we investigated the evolutionary pattern of the CYP family. The number of CYP family members in each species ranged from 221 to 447, as determined using local BLASTP, hmmsearch, and manual checks (Table S33). Among them, *R. irroratum,* which contains 447 CYPs, had the highest gene count. The identified CYP proteins varied in length, ranging from 303 to 621 amino acids. In addition, CYPs were unevenly located on different pseudochromosomes (Fig. S10-21).

To determine the phylogenetic relationship between CYPs, we constructed an unrooted maximum likelihood (ML) phylogenetic tree using protein alignments that primarily contained conserved domains and compared them with CYP superfamily members from *Ar. thaliana*. Following the classification system proposed [36], all CYPs were categorized into two distinct types: A-type, which comprises the CYP71 clan, and non-A-type, which comprises the CYP51, CYP72, CYP74, CYP85, CYP86, CYP97, CYP710, CYP711, and CYP727 clans. Our analysis confirmed monophyly within each clan, with the CYP71 clan being the most gene-rich, representing over half of all identified CYPs. In contrast, the CYP711 and CYP727 clans were the smallest (Fig. 7A–C). In comparison to *Ar. thaliana* and *Ac. chinensis*, we noted a species-specific increase in the CYP family across different *Rhododendron* species, particularly within the CYP71, CYP85, and CYP72 clans. These expansions are likely to play pivotal roles in species-specific adaptations. The CYP71 clan was mainly involved in the biosynthesis of alkaloids, sesquiterpenoids, cyclic terpenoids, and flavonoids, whereas the

CYP85 clan is implicated in the modification of cyclic terpenes and sterols in the BR, abscisic acid (ABA) and gibberellin (GA) pathways. CYP72 is involved in isoprenoid hormone catabolism. Unexpectedly, the CYP family of *R. henanense* subsp. *lingbaoense* was contracted, particularly the CYP72 clan (Fig. 7B). The patterns of duplicated gene pair identification showed that PD and TD remarkably contributed to the CYP family variation. In addition, WGD events accounted for a large proportion of duplications in *R. nivale* subsp. *boreale* and *R. ovatum* (Fig. 7D).

3. Discussion

Climate change is expected to significantly impact mountaintop ecosystems. Understanding the evolutionary patterns and survival strategies of mountaintop species is imperative to protect them [37]. Polyploidy is beneficial for the survival of species in harsh environments [21]. Therefore, it is essential to understand the evolution and adaptation of extremely high-altitude environmental species from the perspective of polyploidy. In this study, we provide a chromosome-scale and haplotype-resolved autotetraploid genome of *R. nivale* subsp. *boreale* using the DNBseq, PacBio CCS, and Hi-C sequencing platforms. The *R. nivale* subsp. *boreale* genome contains 52 pseudochromosomes divided into 13 homologous groups. Our assembly was estimated to be of high quality using four methods (Merqury, BUSCO, CRAQ, and LAI). In addition, we used various methods to determine autotetraploid identity. As the first autotetraploid genome of an alpine woody plant, the genome of *R. nivale* subsp. *boreale* has laid an important foundation for understanding the adaptation and evolution of woody plants in harsh environments at high altitudes. Consistent with previous systematics of *Rhododendron* studies, Subg. *Rhododendron,* to which *R. nivale* subsp. *boreale* belongs, is a

sister of Subg. *Pentanthera* and Subg. *Hymennanthes* [4]. Furthermore, our data suggest that the ancient WGD event in *R. nivale* subsp. *boreale* occurred approximately 78 Mya, which is probably shared with Ericaceae [38]. Overall, *R. nivale* subsp. *boreale* has recently experienced an additional WGD event, in addition to the WGT-γ event shared by the core eudicots and the WGD event shared by the Ericales. Recent polyploidy events, like those observed in other polyploids, are likely important factors for highly conserved pseudochromosomes without rearrangements [31]. The selection analysis revealed similar selection pressures in homologous groups (Fig. S6). The different subgenomes of allopolyploids were under significantly different selection pressures, and it has been speculated that different haplotypes of autopolyploids probably also faced different selection pressures [39]. However, the distribution of *Ka*/*Ks* values did not differ between our homologous groups (Fig. S6). Based on gene family evolution and positive selection analysis, we postulate the potential high-altitude adaptation strategies for *R. nivale* subsp. *boreale*.

Mountaintop ecosystems are exposed to high levels of UV radiation, low partial pressures of oxygen, and volatile temperatures and humidity [38]. Understanding the mechanisms of the adaptation of plants to high altitudes has long interested botanists. Altitude is positively correlated with UV radiation, with UV radiation rates increasing by 5.1–15% every 1,000 m increase in altitude [40]. Alpine plants employ various mechanisms to mitigate the effects of UV radiation, including cell wall surface modifications and the creation of a leaf cuticle consisting of cutin and cuticular waxes [41]. This cuticle serves as a protective shield against water loss and excessive UV radiation by forming a physical barrier between the plant surface and the environment [12]. Second, to enhance their tolerance to UV radiation and protect

themselves from UV damage, plants accumulate flavonoids that absorb UV radiation from strong light [42]. Several genes involved in cuticle and UV tolerance, such as *CER1*, *FARs,* and *MYB27*, are positively selected in high-altitude plants [12]. We identified a similar situation in *R. nivale* subsp. *boreale* living in a high-altitude mountaintop environment. *LTP1* and *LTPG30* have been identified as PSG, of which *LTP1* is associated with the biosynthesis and secretion of cuticular wax [43]. In wild-type bilberry (*Vaccinium myrtillus*), which is closely related to *Rhododendron*, expression of the *LTP* gene specific to the skin suggests its involvement in transporting wax compounds into the cuticle [44]. *LTPG30* performs similar functions [45]. Therefore, surface modification of the cell wall is likely to be the initial line of defense against UV damage in *R. nivale* subsp. *boreale*.

Flavonoids are widely recognized as important chemical compounds that protect plants from UV radiation [46]. We observed a significant expansion of the flavonoid gene family, which suggests that the absorption of UV radiation by flavonoid synthesis may be one of the key ways to reduce UV damage in *R. nivale* subsp. *boreale*. This finding was consistent with the conclusions of previous studies [12]. However, the creation of a wax barrier and biosynthesis of flavonoids that absorb UV radiation are insufficient to fully shield plant cells from the intense UV radiation found in mountainous environments. UV radiation not blocked by such barriers reaches deep into cells, damaging biological macromolecules such as DNA, thereby affecting the growth and development of various cells [47]. In this study, *UVR8* and *RAD23B*, which contribute to DNA repair, were positively selected. Specifically, *UVR8* enhances UV-B perception by interacting with the photomorphogenic repressor *COP1*, while *RAD23B* primarily collaborates with *RAD4* to facilitate nucleotide excision repair [48,49].

These interactions are likely to augment the UV tolerance in *R. nivale* subsp. *boreale.*

In alpine environments, plants have evolved myriad morphological and physiological adaptations to contend with the rigor of high-altitude conditions [50]. *R. nivale* subsp. *boreale* native to mountaintops, typically reaches heights of less than 30 cm, with leaves that seldom exceed 5 mm in both length and width, exhibits delayed flowering, and produces seeds that are nearly indiscernible. These characteristics are thought to be a response of *Rhododendron* to low temperatures at high altitudes, poor nutrition, and extremely short growth cycles [51]. Selection analysis revealed PSGs related to auxins, morphogenesis, and the biological clock. For instance, some PSGs identified are associated with auxin transport (*ABCB19*), seed size (*DA1*), and the biological clock (*ESD4*) [52,53,54]. Concurrently, the gene family for organ development, tissue development, and auxin polar transport expanded significantly. These genes and gene families, which are involved in growth and development, likely shape the special morphology of high-altitude plants such as *R. nivale* subsp. *boreale* and regulate the different stages of their developmental cycle in response to environmental changes, thus better adapting to the extreme spatial and temporal heterogeneity of mountaintop ecosystems [12,55].

Low temperature (average annual temperature below 0 °C), low partial pressure of oxygen (for every 1,000 m increase in altitude, air pressure drops by about 11%) and rapid weather changes (annual average diurnal temperature exceeding 20 °C) are the main factors limiting alpine plant survival [56,57,58]. Under harsh alpine conditions, plants adapt by modifying their morphology, producing specific metabolites, and changing the distribution patterns of biomass [55]. In *R. nivale* subsp. *boreale,* we observed a significant expansion in the gene family associated with BRs, which are pivotal for sustaining plant physiological functions and

significantly contribute to enhancing cold tolerance, drought resistance, and antioxidative capabilities [59,60]. Hence, the expansion of the BR gene family probably enhanced the ability of *R. nivale* subsp. *boreale* to adapt to dramatically changing environments. Moreover, we speculated that the expansion of CYPs, which are associated with stress response, could potentially facilitate the successful adaptation of *R. nivale* subsp. *boreale* at high altitudes. To further understand the adaptability of *R. nivale* subsp. *boreale*, we assessed the dynamics of an important family of transcription factors (AP2/ERF), which is an important group of transcription factors responsive to abiotic stress [61], including key members that adapt to alpine conditions such as *ERF VIIs* and *CBFs/DREB1s*. *ERF VIIs* and *CBFs* are important transcription factors that respond to low oxygen partial pressures and temperatures [34,62]. Unexpectedly, the genes of *Rhododendron* for *ERF VIIs* and *CBFs* continued to be lost lost in low-oxygen, cold alpine environments. *R. nivale* subsp. *boreale* distributed over the alt. 4000 m is no exception. The high expression levels of *ERF VIIs* in *R. nivale* subsp. *boreale* suggests a potential adaptation to low oxygen environments, which warrants further experimental investigation. In response to temperature changes, *Rhododendron* species rely on several pathways to enhance their low-temperature resistance, including *CBFs*-mediated cold tolerance and an integrated regulatory network of ABA, the MAPK cascade, and Ca^{2+} signaling [63,64]. Our positive selection analysis results suggested that *M3K1* and *CNGC1,* which are associated with the MAPK cascade and Ca^{2+} signal transduction, probably play important roles in low-temperature adaptation.

Polyploidy leads to rapid changes in gene expression and epigenetics, giving the polyploid a significant selective advantage over its diploid progenitors and serving as a crucial

mechanism for plants to swiftly adjust to severe environmental stress [6,18]. Moreover, differential splicing, which is a crucial mechanism in the eukaryotic stress response, changes rapidly after polyploidy and is associated with abiotic stress [65]. Certainly, the influence of genome doubling on phenotypes or life history traits can directly affect the likelihood of survival under challenging circumstances [18]. These include more viable seeds, more rapid growth, and stronger photosynthesis [19,66]. These characteristics provide plants with great advantages under adverse environmental conditions.

The decline in the fitness of autopolyploids, especially young autopolyploids, is usually attributed to multivalent chromosome pairing during meiosis and mutations in crossover (CO) frequency and distribution [23,67]. Consequently, autopolyploids experience significant disruptions in their developmental programs, resulting in a considerable reduction in seed production and a high incidence of aneuploid offspring [68]. Addressing these issues requires precise adaptive control of meiosis, such as reduced formation of multichromosome associations and reduced axis lengths [69]. The RECQ4a/4b (BLM)-TOP3α-RMI1 (BTR) complex plays a pivotal role in limiting CO outcomes and maintaining chromosome integrity [70,71]. Mutations of *RECQ4*, one of its members, significantly affect the stability of polyploid meiosis [72]. Notably, *TOP3α*, an important gene related to the BTR complex, exhibits positive selection in *R. nivale* subsp. *boreale* and likely serves as a key factor in promoting accurate chromosomal segregation during meiosis in autopolyploids.

Our results revealed that *R. nivale* subsp. *boreale* distributed on the mountaintop is an autotetraploid, which is probably mediated by the harsh environment at high altitudes. Paleopolyploid events are shared among the other 10 *Rhododendron* species of diploids. Our

conjecture regarding the alpine adaptation mechanisms of *R. nivale* subsp. *boreale* aligns with those of previous studies: cell wall modification, flavonoid biosynthesis, DNA repair, inhibition of chlorophyll synthesis, and auxin and BR biosynthesis and transduction are probably the main high-altitude adaptation pathways [12]. Polyploidization likely plays an important role in mountaintop survival because of its dominant gene expression pattern [18]. Notably, *TOP3α* is speculated to be an important gene during meiosis in autotetraploids, essential for the generation of normal gametes and implicated in the attenuation of CO events during meiosis. However, these hypotheses require verification through biological experiments. Moreover, the mechanism of the formation of natural polyploids remains to be fully elucidated, and the alpine environment, where polyploidy is concentrated, offers an ideal setting for investigation.

Overall, we assembled the first genome of Subge. *Rhododendron*, a rare high-altitude woody autotetraploid genome that provides an important resource for the domestication of high-altitude ornamentals and our understanding of polyploid origin and evolution in mountaintop ecosystems.

4. Materials and methods

4.1 Plant materials and sequencing

R. nivale subsp. *boreale* plant materials were collected from Baima Mountain, Dêqên County, Yunnan Province, China (99°4′13″E, 28°20′24″N, alt. 4287.5 m). The plant materials were immersed in liquid nitrogen immediately after collection and preserved at -80 °C. High-quality DNA isolated from young leaves was used to create the libraries. Long-read libraries

were constructed and sequenced using the PacBio Sequel II sequencing platform. To construct Hi-C libraries, genomic DNA was cross-linked with formaldehyde and digested using the MboI restriction enzyme into 300–500 bp fragments, which were sequenced on the BGI DNBseq sequencing platform. For short reads, DNA libraries were constructed and sequenced on the BGI DNBseq sequencing platform. Three biological replicates of roots, stems, leaves, and buds of *R. nivale* subsp. *boreale* were sampled. The cDNA libraries were constructed and sequenced on a BGI DNBseq sequencing platform.

4.2 Genome survey

Flow cytometry and *k-mer* analysis were used to evaluate the genome of *R. nivale* subsp. *boreale*. The following procedures were used for flow cytometry: preparation of nuclear suspension, DNA-specific staining, and testing. We selected *R. griersonianum* as an internal control. Graphical analysis was performed using ModFit LT 5.0 (www.vsh.com/products/mflt/index.asp) with a coefficient of variation (CV) controlled to within 5. For *k-mer* analysis, DNBseq short-reads clean data were used to count *k-mer* frequency with *k-mer* set to 21 using jellyfish v2.3.0 [73]. Genome size was estimated based on the 21 *k-mers* distribution. Ploidy was estimated using SmudgePlot v0.2.5 [29].

4.3 Genome assembly and scaffolding

The PacBio circular consensus sequencing (CCS) long-read data were assembled using Hifiasm v0.18.9 with Hi-C integration [74], Canu v1.9 [75], and HiCanu v2.2 [76]. We used the parameters of the genome of *Saccharum spontaneum* [16]. The integrity and continuity of the assembly were assessed separately, and the highest quality assembly was used for the scaffolding. The ALLHiC pipeline was used to improve assembly at the chromosomal level

based on five steps: pruning, partitioning, rescue, optimization, and construction [77]. Manual checks were conducted on potential misassemblies and corrected using Juicebox v1.11.08 [78]. Finally, the assembled genome was evaluated using Benchmarking Universal Single-Copy Orthologs (BUSCO) v5.4.6 [79], Merqury v1.3 [80], and Clipping information for Revealing Assembly Quality (CRAQ) v1.0.9 [81] using default parameters. Short reads mapped to the assembled genome using BWA v0.7.17-r1188 [82] and SAMtools v1.17 [83] were counted as properly paired.

4.4 Genome annotation

De novo prediction and homology alignment were used to identify whole-genome repeats. The LTRs were initially identified using LTRharvest [84] and LTR_Finder [85]. LTR_retriever v2.9.4 [86] was used to accurately identify LTR retrotransposons (LTR-RTs), generate a nonredundant LTR-RT library, and generate the LTR assembly index (LAI). A homology search was conducted to predict repeat elements using RepeatMasker v4.1.4 [87]. Transfer RNAs (tRNAs) were annotated using tRNAscan-SE v2.0.9 [88], and ribosomal RNAs (rRNAs) were identified using RNAmmer v1.2 [89]. Other noncoding RNAs, including miRNAs and snRNAs, were annotated by comparison using Infernal v1.1.4, with the Rfam database [90,91].

We combined ab initio, homolog and transcriptome-based strategies to predict the expression of high-quality protein-coding genes. In our transcriptome-based strategies, we used HISAT2 v2.2.1 [92] to align clean reads of the transcriptome with the genome. Trinity v2.14.0 [93] and StringTie v2.2.1 [94] were used to assemble transcripts. BRAKER3 [95] and PASA v2.5.2 [96] were used to predict gene structure based on the assembled transcripts and to generate ab initio gene predictor training sets. For ab initio, SNAP [97], GlimmerHMM v3.0.1,

and GeneID v1.4 [98] were used to annotate gene structures based on the training sets. For homology-based prediction, protein sequences from a total of eight species, namely, *Arabidopsis thaliana, Vitis vinifera, Glycine max, Nicotiana attenuata, Oryza sativa, R. ovatum, R. griersonianum,* and *R. mole*, were aligned with the genome of *R. nivale* subsp. *boreale* using GeMoMa v1.9 [99]. All gene structures annotated using the above approaches were integrated using the EVidenceModeler (EVM) [100]. Functional annotation of genes was performed using EggNOG v5.0 [101], and protein sequences were aligned to the UniProt database using BLAST v2.6.0 [102].

4.5 Identification of polyploid type

Used GenomeScope 2.0 [29] to count the proportion of nucleotide heterozygosity forms based on the 21 *k-mer* count distributions. AAAB < AABB indicates allotetraploidy, whereas AAAB > AABB indicates autotetraploidy. JCVI utility libraries [103] were used to analyze collinear relationships between haplotypes. To identify the relationship between different haplotypes and related species, 11 transcriptome datasets from seven related species from previous studies with four haplotypes and transcripts of *R. nivale* subsp. *boreale* were used to reconstruct the phylogenetic tree. StringTie v2.2.1 [94] was used to assemble transcripts. A maximum likelihood (ML) tree was reconstructed using IQ-TREE v2.2.2.2 [104] with 1000 ultrafast bootstrap replicates after single-copy orthologs were identified by OrthoFinder v2.5.4 [105].

4.6 Comparative genomics analysis

The genomes of *Actinidia chinensis, Amborella trichopoda, Camptotheca acuminata, Davidia involucrata, Oryza sativa, Panicum hallii, R. delavayi, R. griersonianum, R. henanense*

subsp. *lingbaoense, R. irroratum, R. molle, R. ovatum, R. ripense, R. simsii, R. vialii, Vaccinium darrowii,* and *Vitis vinifera* were used for comparative genomics analysis with our assembly of *R. nivale* subsp. *boreale*. Single-copy orthologs were identified based on protein sequences using OrthoFinder v2.5.4 [105]. The protein sequences in each single-copy orthogroup were aligned using MUSCLE v5.1 [106] and filtered using trimAI v1.4 [107] and used to construct a phylogenetic tree using IQ-TREE v2. 2.2.2 [104] with 1000 ultra-fast bootstrap replicates. The MCMCtree program in PAML v4.10 [108] was used to estimate the divergence times. Calibration times were obtained from the TimeTree database (http://timetree.org) and previous studies [4,109]. A total of four calibration points were used to calibrate age: angiosperms 168–194 Mya; monocots eudicots 142.1–163.5 Mya; *Rhododendron* crown 54.5 Mya; *Panicum hallii-Oryza sativa* 41.4–51.9 Mya. Based on the ultrametric tree, the expansion and contraction of gene families were estimated using CAFÉ 5 [110]. Functional enrichment analysis of Gene Ontology (GO) and Kyoto Encyclopedia of Genes and Genomes (KEGG) was performed using the R package clusterProfiler v4.8.3 [111]. Synteny between different species was identified and visualized using the MCscan pipeline in JCVI [103] and MCScanX [112] with default parameters. The *Ks* values of the ortholog and paralog pairs were calculated using KaKs_Calculator v2.0 [113] after alignment with ParaAT v2.0 [114]. WGD times were estimated as T=*Ks*/2r (T is the WGD time and r is the rate of divergence). The value of r was obtained from a previous study [115].

4.7 Selective analysis

Based on 1,122 single-copy conserved orthologs from Ericales (10 *Rhododendrons, Va. darrowii,* and *Ac. Chinensis*), we performed the positive selection analysis acting on the *R.*

nivale subsp. *boreale* clade by running separate aBSREL, Clade Model, MEME, and Contrast-FEL. aBSREL was implemented in HyPhy v2.5.48 [116] with exploratory analysis, representing an improved version of traditional "branch-site" models. The aBSREL test models both site-level and branch-level nonsynonymous-to-synonymous mutation ratio ω heterogeneity but does not test for selection at specific sites. To obtain more accurate PSGs, we used clade model C (CmC) to check the consistency of the model in PAML [108]. This model tested the differential selection pressure between the foreground branches and background for each gene. CmC was then compared with the null model M2a_rel using likelihood-ratio tests (LRT) [117].

To obtain information on specific sites during episodic selection, we applied MEME and Contrast-FEL. MEME tests [118] for sites that were subjected to episodic positive or diversifying selection were performed for each gene. The MEME employs a mixed-effects maximum likelihood approach to test the hypothesis that individual sites are subject to episodic, positive, or diversifying selection. For each site, MEME infers two ω rate classes and the corresponding weights representing the probability that the site evolves under each corresponding ω rate class at a given branch. Contrast-FEL [119] was used to estimate the difference in ω at each site between different branch sets in codon alignments. The false discovery rate (FDR) was used to correct for multiple comparisons.

To further understand the selection pressure characteristics of the high- and low-altitude genomes, we used KaKs_Calculator [113] to detect selected genes between *R. nivale* subsp. *boreale* (high altitude) and *R. ovatum* (low altitude). All genes with a P value < 0.05 and ω (Ka/Ks) > 1 were identified as candidate PSGs.

4.8 Gene expression analysis

Clean reads of the transcriptome were mapped to the genome using STAR and gene expression levels were estimated using STAR v2.7.10b [120]. Accurate quantification (transcripts per kilobase per million mapped reads) of genes was performed using RSEM v1.3.3 [121]. We selected the expression levels of single-match alleles to explore the differences in expression between alleles. The four alleles were compared pairwise to identify the differentially expressed alleles. Pairs of alleles exhibiting less than a twofold difference in expression were classified as neutral, whereas all other pairs were categorized as non-neutral, that is, DEL. [122]. We used the Kruskal–Wallis test to assess differences in median values among multiple independent samples. The level of significance was set at P value < 0.05.

4.9 Identification of duplicate gene modes

Different modes of duplicated gene pairs were identified using the DupGen Finder pipeline [123]. The duplicated gene pairs were divided into five categories: whole-genome duplicates (WGD), tandem duplicates (TD), proximal duplicates (PD), transposed duplicates (TRD), and dispersed duplicates (DSD).

4.10 Identification and analysis of key gene families

The AP2/ERF and cytochrome P450 (CYP) gene families were identified using HMMER v3.3.2 (HMMER.org). The structural domain files corresponding to AP2/ERF (PF00847) and CYP (PF00067) were obtained from the Pfam database (https://www.ebi.ac.uk/interpro/). A domain file is used as the first template to search for a family. The filtered domain sequences were used as species-specific templates in the second scan. The Pfam and CDD databases (https://www.ncbi.nlm.nih.gov/cdd/) were used to verify conserved domains. Conserved

sequences containing the main domains were aligned using MAFFT v7.520 [124] and used to construct a phylogenetic tree of the gene family using FastTree v2.1.11 [125] with the GTR + CAT model. Phylogenetic analysis of *CBFs* and *ERF VIIs* was performed using IQ-TREE v2.2.2.2 with 1000 replicates [104]. Gene motifs were predicted using MEME software v5.5.1 and visualized using TBtools v2.003 [126]. BadiRate v1.35 [127] was used to estimate family turnover rates based on likelihood-based methods.

Data availability

The raw sequencing data of this study have been deposited in the Sequence Read Archive (SRA) under Bioproject number PRJNA1040959. The genome assembly and annotation data are available at figshare (https://doi.org/10.6084/m9.figshare.24565225.v1).

Author contribution statement

Zhen-Yu Lyu: Conceptualization, Methodology, Visualization, Formal analysis, Writing - Original Draft, Writing - Review & Editing. **Shi-Kang Shen:** Conceptualization, Methodology, Writing - Original Draft, Writing - Review & Editing. **Si-Qi Wang:** Resources. **Xiong-Li Zhou:** Resources. **Rui Zhang:** Resources. **Gao-Ming Yang:** Cytological experiment. **Jie-Yu Zhang:** Cytological experiment. **Wen-Guang Sun:** Cytological experiment.

Funding

This study was supported by the Science and Technology Development Fund of Guidance from the Central Government to Locals in Yunnan Province (202207AB110016), Major Program for Basic Research Project of Yunnan Province (202101BC070002), National Natural

Science Foundation of China (31870529), the Graduate Scientific Research Fund Project of Yunnan University (KC-22221373), and Education Department of Yunnan, Scientific Research Fund Project (2024Y003).

Conflict of Interest

The authors declare that they have no competing interests.

References:

1. Fang R, Min TL. The floristic study on the genus *Rhododendron*. Acta Botanica Yunnanica. 1995;17:359–79.

2. Chen YS, Deng T, Zhou Z, Sun H. Is the East Asian flora ancient or not? Natl Sci Rev. 2018;5:920–32.

3. Basnett S, Rengaian G. A Comprehensive Review on the taxonomy, ecology, reproductive biology, economic importance and conservation status of Indian Himalayan *Rhododendrons*. Bot Rev. 2022;88:505–44.

4. Xia XM, Yang MQ, Li CL, Huang SX, Jin WT, Shen TT, Wang F, Li XH, Yoichi W, Zhang LH, Zheng YR, Wang XQ. Spatiotemporal Evolution of the Global Species Diversity of *Rhododendron*. Mol Biol Evol. 2022;39:msab314.

5. Darlington CD, Wylie AP. Chromosome atlas of flowering plants. George Allen and Unwin Ltd. Londonn, UK; 1955. p. 217–8.

6. Zhang J, Peng HW, Xia FC, Wang W. A comparison of seed plants' polyploids between the Qinghai-Tibet Plateau alpine and the Pan-Arctic regions. Biodiversity Science. 2021;29:1470–80.

7. Liu B, Zhao FM, Zhou H, Xia YP, Wang XY. Photoprotection conferring plant tolerance to freezing stress through rescuing photosystem in evergreen *Rhododendron*. Plant Cell Environ. 2022;45:2093–108.

8. Popescu R, Kopp B. The genus *Rhododendron*: An ethnopharmacological and toxicological review. J Ethnopharmacol. 2013;147:42–62.

9. Guo X, Dong Z, Li Q, Wan DG, Zhong JB, Dong D, Huang MZ. Flavonoids from *Rhododendron nivale* Hook. f delay aging via modulation of gut microbiota and glutathione metabolism. Phytomedicine. 2022;104:154270.

10. Hu YB, Wang XP, Xu YC, Yang H, Tong ZY, Tian R, Xu SH, Yu L, Guo YL, Shi P, et al. Molecular mechanisms of adaptive evolution in wild animals and plants. Sci China Life Sci. 2023;66:453–95.

11. Marks R. A, Hotaling S, Frandsen P. B, VanBuren R. Representation and participation across 20 years of plant genome sequencing. Nat. Plants. 2021;7:1571–8.

12. Zhang X, Kuang TH, Dong WL, Qian ZH, Zhang HJ, Landis JB, Feng T, Li LJ, Sun YX, Huang JL, et al. Genomic convergence underlying high-altitude adaptation in alpine plants. J Integr Plant Biol. 2023;65:1620–35.

13. De Storme N, Geelen D. The impact of environmental stress on male reproductive development in plant: Biologival processes and molecular mechanisms. Plant Cell Environ. 2014;37:1–18.

14. Chen HT, Zeng Y, Yang YZ, Huang LL, Tang BL, Zhang H, Hao F, Li W, Li YH, Liu YB, et al. Allele-aware chromosome-level genome assembly and efficient transgene-free genome editing for the autotetraploid cultivated alfalfa. Nat Commun. 2020;11:2494.

15. Wang F, Xia ZQ, Zou ML, Zhao L, Jiang SR, Zhou Y, Zhang CJ, Ma YZ, Bao YT, Sun HH, et al. The autotetraploid potato genome provides insights into highly heterozygous species. Plant Biotechnol J. 2022;20:1996–2005.

16. Zhang Q, Qi YY, Pan HR, Tang HB, Wang G, Hua XT, Wang YJ, Lin LY, Li Z, Li YH, et al. Genomic insights into the recent chromosome reduction of autopolyploid sugarcane *Saccharum spontaneum*. Nat Genet. 2022;54:885–96.

17. Zhang HY, He Q, Xing LS, Wang RY, Wang Y, Liu Y, Zhou QH, Li XZ, Jia Z, Liu Z, et al. The haplotype-resolved genome assembly of autotetraploid rhubarb *Rheum officinale* provides insights into the genome evolution and massive accumulation of anthraquinones. Plant Commun. 2023;26:100677.

18. Van de Peer, Y, Ashman TL, Soltis PS, Soltis DE. Polyploidy: an evolutionary and ecological force in stressful times. Plant Cell. 2021;33:11–26.

19. Stevens AV, Nicotra AB, Godfree RC, Guja LK. Polyploidy affects the seed, dormancy and seedling characteristics of a perennial grass, conferring an advantage in stressful climates. Plant Biol. 2020;22:500–13.

20. Liu CJ, Wang YG. Does one subgenome become dominant in the formation and evolution of a polyploid? Ann Bot. 2023;131:11–6.

21. Wang KL, Deng PR, Yao Z, Dong JY, He Z, Yang P, Liu YB. Biogeographic patterns of polyploid species for the angiosperm flora in China. J Syst Evol. 2022;61:776–89.

22. Heslop-Harrison JS, Schwarzacher T, Liu Q. Polyploidy: its consequences and enabling role in plant diversification and evolution. Ann. Bot. 2023;131:1–10.

23. Cifuentes M, Grandont L, Moore G, Chevre AM, Jenczewski E. Genetic regulation of meiosis in polyploid species: new insights into an old question. New Phytol. 2010;186:29–36.

24. Grandont L, Jenczewski E, Lloyd A. Meiosis and its deviations in polyploid plants. Cytogenet. Genome Res. 2013;140:171–84.

25. Bomblies, K. Learning to tango with four (or more): the molecular basis of adaptation to polyploid meiosis. Plant Reprod. 2022;36:107–24.

26. Gou XW, Bian Y, Zhang A, Zhang HK, Wang B, Lv RL, Li JZ, Zhu B, Gong L, Liu B. Transgenerationally precipitated meiotic chromosome instability fuels rapid karyotypic evolution and phenotypic diversity in an artificially constructed allotetraploid wheat (AADD). Mol, Biol, Evol. 2018;35:1078–91.

27. Huang G, Wu Z, Percy RG, Bai MZ, Li Y, Frelichowski JE, Hu J, Wang K, Yu JZ, Zhu YX. Genome sequence of *Gossypium herbaceum* and genome updates of *Gossypium arboreum* and *Gossypium hirsutum* provide insights into cotton A-genome evolution. Nat. Genet. 2020;52:516–24.

28. Zhang X, Pandey MK, Wang, JP, Zhao KK, Ma XL, Li ZF, Zhao K, Gong FP, Guo BZ, Varshney R, et al. Chromatin spatial organization of wild type and mutant peanuts reveals high-resolution genomic architecture and interaction alterations. Genome Biol. 2021;22:315.

29. Ranallo-Benavidez TR, Jaron KS, Schatz MC. GenomeScope 2.0 and Smudgeplot for reference-free profiling of polyploid genomes. Nat Commun. 2020;11:1432.

30. Miao Y, Luo D, Zhao T, Du H, Liu Z, Xu Z, Guo L, Chen C, Peng S, Li JX, et al. Genome sequencing reveals chromosome fusion and extensive expansion of genes related to secondary metabolism in Artemisia argyi. Plant Biotechnol J. 2022;20:1902–1915.

31. Song AP, Su JS, Wang HB, Zhang ZR, Zhang XT, van de Peer Y, Chen F, Fang WM, Guan ZY, Zhang F, et al. Analyses of a chromosome-scale genome assembly reveal the origin and evolution of cultivated chrysanthemum. Nat Commun. 2023;14:2021.

32. Xu J, Wang XY, Guo WZ. The cytochrome P450 superfamily: key players in plant development and

defense. J Integr Agric. 2015;14:1673–86.

33. Feng K, Hou XL, Xing GM, Liu JX, Duan AQ, Xu ZS, Li MY, Zhuang J, Xiong AS. Advances in AP2/ERF super-family transcription factors in plant. Crit Rev Biotechnol. 2020;40:750–76.

34. Abbas M, Sharma G, Dambire C, Marquez J, Alonso-Blanco C, Proano K, Holdsworth MJ. An oxygen-sensing mechanism for angiosperm adaptation to altitude. Nature. 2022;606:565–9.

35. Nie YQ, Guo LY, Cui FQ, Shen YR, Ye XX, Deng DY, Wang S, Zhu JH, Wu, WW. Innovations and stepwise evolution of CBFs/DREB1s and their regulatory networks in angiosperms. J Integr Plant Biol. 2022;64: 2111–25.

36. Durst F, Nelson DR. 1995. Diversity and evolution of plant P450 and P450-reductases. Drug Metabol Drug Interact 12:189-206.

37. Shen SK, Zhou XL, Wang SQ, Lyu ZY, Zhang R, Liu Y, Long B. Protect fragile mountaintop ecosystems. Science. 2023;380:1114–5.

38. Wu XP, Zhang L, Wang XY, Zhang RA, Jin GH, Hu YT, Yang H, Wu ZZ, Ma YP, Zhang CJ, Wang JH. Evolutionary history of two evergreen *Rhododendron* species as revealed by chromosome-level genome assembly. Front. Plant Sci. 2023;14:1123707.

39. Xu P, Xu J, Liu G, Chen L, Zhou ZX, Peng WZ, Jiang YL, Zhao ZX, Jia ZY, Sun YH et al. The allotetraploid origin and asymmetrical genome evolution of the common carp *Cyprinus carpio*. Nat Commun. 2019;10:4625.

40. Blumthaler M, Ambach W, R Ellinger. Increase in solar UV radiation with altitude. J Photochem Photobiol B. 1997;39:130–4.

41. Kerstiens G. Cuticular water permeability and its physiological significance. J Exp Bot. 199647:1813–32.

42. Tossi V, Lombardo C, Cassia R, Lamattina L. Nitric oxide and flavonoids are systemically induced by UV-B in maize leaves. Plant Sci. 2012;193:103–9.

43. Choi YE, Lim S, Kim HJ, Han JY, Lee MH, Yang Y, Kim JA, Kim YS. Tobacco *NtLTP1*, a glandular-specific lipid transfer protein, is required for lipid secretion from glandular trichomes. Plant J. 2012;70:480–

91.

44. Trivedi P, Nguyen N, Klavins L, Kviesis J, Heinonen E, Remes J, Jokipii-Lukkari S, Klavins M, Karppinen K, Jaakola L, Haggman H. Analysis of composition, morphology, and biosynthesis of cuticular wax in wild type bilberry (*Vaccinium myrtillus* L.) and its glossy mutant. Food Chem. 2021;354:12957.

45. Gao HN, Jiang H, Lian XY, Cui JY, You CX, Hao YJ, Li YY. Identification and functional analysis of the *MdLTPG* gene family in apple. Crit. Rev. Biotechnol. 2021;163:338–47.

46. Emiliani J, Grotewold E, Ferreyra MLF, Casati P. Flavonols protect *Arabidopsis* plants against UV-B deleterious effects. Mol Plant. 2013;6:1376–9.

47. McKenzie R, Conner B, Bodeker G. Increased summertime UV radiation in New Zealand in response to ozone loss. Science. 1999;285:1709–11.

48. Lahari T, Lazaro J, Schroeder DF. *RAD4* and *RAD23/HMR* Contribute to *Arabidopsis* UV Tolerance. Genes. 2018;9:8.

49. Rai N, O'Hara A, Farkas D, Safronov O, Ratanasopa K, Wang F, Lindfors AV, Jenkins GI, Lehto T, Salojärvi J, et al. 2020. The photoreceptor *UVR8* mediates the perception of both UV-B and UV-A wavelengths up to 350 nm of sunlight with responsivity moderated by cryptochromes. Plant Cell and Environment 43:1513-1527.

50. Mohl P, von Buren R. S, Hiltbrunner E. Growth of alpine grasslandwill start and stop earlier under climate warming. Nat Commun. 2022;13:7398.

51. Liu JM, de Vos JM, Körner C, Yang Y. 2023. Phylogeny and phenotypic adjustments drive functional traits in across elevations in its diversity hot-spot in W-China. Alpine Botany 133:69-84.

52. Li YH, Zheng LY, Corke F, Smith C, Bevan MW. Control of final seed and organ size by the *DA1* gene family in *Arabidopsis thaliana*. Genes Dev. 2008;22:1331–6.

53. Titapiwatanakun B, Blakeslee JJ, Bandyopadhyay A, Yang H, Mravec J, Sauer M, Cheng Y, Adamec J, Nagashima A, Geisler M, et al. *ABCB19/PGP19* stabilises *PIN1* in membrane microdomains in *Arabidopsis*. Plant J. 2009;57:27–44.

54. Gao, YS, Badejo AA, Sawa Y, Ishikawa T. Analysis of two l-Galactono-1,4-Lactone-Responsive genes

with complementary expression during the development of Arabidopsis thaliana. Plant Cell Physiol. 2012;53:592–601.

55. Sun H, Niu Y, Chen YS, Song B, Liu CQ, Peng DL, Chen JG, Yang Y. Survival and reproduction of plant species in the Qinghai-Tibet Plateau. J Syst Evol. 2014;52:378–96.

56. Apte CV. Barometric Pressure at High Altitude: Revisiting West's Prediction Equation, and More. High Alt Med Biol. 2023;24:85–93.

57. Li XT, Guo W, Li SH, Zhang JZ, Ni XN. The different impacts of the daytime and nighttime land surface temperatures on the alpine grassland phenology. Ecosphere. 2021;12:e03578.

58. Wu QB, Liu YZ. Ground temperature monitoring and its recent change in Qinghai-Tibet Plateau. Cold Reg Sci Technol. 2004;38:85–92.

59. Clouse SD, Sasse JM. Brassinosteroids: Essential regulators of plant growth and development. Annu Rev Plant Physiol, Plant Mol Biol. 1998;49:427–51.

60. Chaudhuri A, Halder K, Abdin MZ, Majee M, Datta A. Abiotic stress tolerance in plants: brassinosteroids navigate competently. Int J Mol Sci. 2022;23:14577.

61. Riechmann JL, Meyerowitz EM. 1998. The AP2/EREBP family of plant transcription factors. Biol Chem 379:633-646.

62. Thomashow MF. Plant cold acclimation: Freezing tolerance genes and regulatory mechanisms. Annu Rev Plant Physiol Plant Mol Biol. 1999;50:571–99.

63. Cao K, Zhang ZY, Fan H, Tan Y, Xu HW, Zhou XF. Comparative transcriptomic analysis reveals gene expression in response to cold stress in *Rhododendron aureum* Georgi. Theor Exp Plant Physiol. 2022;34:347–66.

64. Zhang QY, Li Y, Cao K, Xu HW, Zhou XF. Transcriptome and proteome depth analysis indicate ABA, MAPK cascade and Ca^{2+} signaling co-regulate cold tolerance in *Rhododendron chrysanthum* Pall. Front Plant Sci. 2023;14:1146663.

65. Staiger D, Brown JWS. Alternative splicing at the intersection of biological timing, development, and stress responses. Plant Cell. 2013;25:3640–56.

66. Mao HT, Chen MY, Su YQ, Wu N, Yuan M, Yuan S, Brestic M, Zivcak M, Zhang HY, Chen Y. Comparison on photosynthesis and antioxidant defense systems in wheat with different ploidy levels and octoploid Triticale. Int J Mol Sci. 2018;19:3006.

67. Parra-Nunez P, Fernández-Jiménez N, Pachon-Penalba M, Sanchez-Moran E, Pradillo M, Santos JL. 2024. Synthetically induced autotetraploids provide insights into the analysis of meiotic mutants with altered crossover frequency. New Phytologist 241:197-208.

68. Singliarova B, Hojsgaard D, Muller-Scharer H, Mraz P. The novel expression of clonality following whole-genome multiplication compensates for reduced fertility in natural autopolyploids. Proc Biol Sci. 2023;290:20230389.

69. Morgan C, Zhang HK, Henry CE, Franklin FCH, Bomblies K. Derived alleles of two axis proteins affect meiotic traits in autotetraploid *Arabidopsis arenosa*. Proc Natl Acad Sci USA. 2020;117:8980–8988.

70. Séguéla-Arnaud M, Choinard S, Larchevêque C, Girard C, Froger N, Crismani W, Mercier R. 2016. *RMI1* and *TOP3α* limit meiotic CO formation through their C-terminal domains. Nucleic Acids Research 45:1860-1871.

71. Séguéla-Arnaud M, Crismani W, Larchevêque C, Mazel J, Froger N, Choinard S, Lemhemdi A, Macaisne N, Van Leene J, Gevaert K, et al. 2015. Multiple mechanisms limit meiotic crossovers: TOP3alpha and two BLM homologs antagonize crossovers in parallel to FANCM. Proc Natl Acad Sci U S A 112:4713-4718.

72. Bazile J, Nadaud I, Lasserre-Zuber P, Kitt J, De Oliveira R, Choulet F, Sourdille P. 2024. *TaRECQ4* contributes to maintain both homologous and homoeologous recombination during wheat meiosis. Frontiers in Plant Science 14.

73. Marcais G, Kingsford C. A fast, lock-free approach for efficient parallel counting of occurrences of k-mers. Bioinformatics. 2011;27:764–70.

74. Cheng HY, Concepcion T, Feng XW, Zhang HW, Li H. Haplotype-resolved de novo assembly using phased assembly graphs with hifiasm. Nat Methods. 2021;18:170–5.

75. Koren S, Rhie A, Walenz BP, Dilthey AT, Bickhart DM, Kingan SB, Hiendleder S, Williams JL, Smith TPL, Phillippy AM. De novo assembly of haplotype-resolved genomes with trio binning. Nat Biotechnol. 2018;36:1174–82.

76. Nurk S, Walenz BP, Rhie A, Vollger MR, Logsdon GA, Grothe R, Miga KH, Eichler EE, Phillippy AM, Koren S. HiCanu: accurate assembly of segmental duplications, satellites, and allelic variants from high-fidelity long reads. Genome Res. 2020;30:1291–305.

77. Zhang XT, Zhang SC, Zhao Q, Ming R, Tang HB. Assembly of allele-aware, chromosomal-scale autopolyploid genomes based on Hi-C data. Nat Plants. 2019;5:833–845. doi:10.1038/s41477-019-0487-8.

78. Durand NC, Robinson JT, Shamim S, Machol I, Mesirov P, Lander ES, Aiden EL. Juicebox provides a visualization system for Hi-C contact maps with unlimited zoom. Cell Syst. 2016;3:99–101.

79. Simao FA, Waterhouse RM, Ioannidis P, Kriventseva EV, Zdobnov EM. BUSCO: assessing genome assembly and annotation completeness with single-copy orthologs. Bioinformatics. 2015;31:3210–2.

80. Rhie A, Walenz BP, Koren S, Phillippy AM. Merqury: reference-free quality, completeness, and phasing assessment for genome assemblies. Genome Biol. 2020;21:245.

81. Li KP, Xu P, Wang JP, Yi X, Jiao YN. Identification of errors in draft genome assemblies at single-nucleotide resolution for quality assessment and improvement. Nat Commun. 2023;14:6556.

82. Li H, Durbin R. Fast and accurate short read alignment with Burrows-Wheeler transform. Bioinformatics. 2009;25:1754–60.

83. Li H, Handsaker B, Wysoker A, Fennell T, Ruan J, Homer N, Marth G, Abecasis G, Durbin R. The Sequence Alignment/Map format and SAMtools. Bioinformatics, 2009;25:2078–9.

84. Ellinghaus D, Kurtz S, Willhoeft U. LTRharvest, an efficient and flexible software for de novo detection of LTR retrotransposons. BMC Bioinformatics. 2008;9:18.

85. Zhao X, Wang H. LTR_FINDER: an efficient tool for the prediction of full-length LTR retrotransposons. Nucleic Acids Res. 2007;35:W265–W268.

86. Ou SJ, Jiang N. LTR_retriever: a highly accurate and sensitive program for identification of long terminal repeat retrotransposons. Plant Physiol. 2018;176:1410–22.

87. Tarailo-Graovac M, Chen NS. Using RepeatMasker to identify repetitive elements in genomic sequences. Current protocols in bioinformatics. 2009. Chapter 4:4.10.1–4.10.14. doi:10.1002/0471250953.bi0410s25

88. Lowe TM, Eddy SR. tRNAscan-SE: A program for improved detection of transfer RNA genes in genomic

sequence. Nucleic Acids Res. 1997;25:955–64.

89. Lagesen K, Hallin P, Rodland EA, Staerfeldt HH, Rognes T, Ussery DW. RNAmmer: consistent and rapid annotation of ribosomal RNA genes. Nucleic Acids Res. 2007;35:3100–8.

90. Nawrocki EP, Burge SW, Bateman A, Daub J, Eberhardt RY, Eddy SR, Floden EW, Gardner PP, Jones TA, Tate J, et al. Rfam 12.0: updates to the RNA families database. Nucleic Acids Res. 2015;43:D130–D137.

91. Nawrocki EP, Eddy SR. Infernal 1.1: 100-fold faster RNA homology searches. Bioinformatics. 2013;29:2933–5.

92. Kim D, Paggi JM, Park C, Bennett C, Salzberg SL. Graph-based genome alignment and genotyping with HISAT2 and HISAT-genotype. Nat Biotechnol. 2019;37:907–15.

93. Haas BJ, Papanicolaou A, Yassour M, Grabherr M, Blood PD, Bowden J, Couger MB, Eccles D, Li B, Lieber M, et al. De novo transcript sequence reconstruction from RNA-seq using the Trinity platform for reference generation and analysis. Nat. Protoc. 2013;8:1494–512.

94. Pertea M, Pertea GM, Antonescu CM, Chang TC, Mendell JT, Salzberg SL. StringTie enables improved reconstruction of a transcriptome from RNA-seq reads. Nat Biotechnol. 2015;33:290–5.

95. Hoff KJ, Lange S, Lomsadze A, Borodovsky M, Stanke M. BRAKER1: Unsupervised RNA-Seq-Based fenome annotation with GeneMark-ET and AUGUSTUS. Bioinformatics. 2016;32:767–769.

96. Haas BJ, Delcher AL, Mount SM, Wortman JR, Smith RK, Hannick LI, Maiti R, Ronning CM, Rusch DB, Town CD, et al. Improving the *Arabidopsis* genome annotation using maximal transcript alignment assemblies. Nucleic Acids Res. 2003;31:5654–66.

97. Korf I. Gene finding in novel genomes. BMC Bioinformatics. 2004;5:59. doi:10.1186/1471-2105-5-59.

98. Blanco E, Genis P, Roderic G. Using geneid to identify genes. Current protocols in bioinformatics. 2007. 4:4.3.1–4.3.28.

99. Keilwagen J, Hartung F, Grau J. GeMoMa: Homology-Based gene prediction utilizing intron position conservation and RNA-seq data. Methods Mol Biol. 2019;1962:161–77.

100. Haas BJ, Salzberg SL, Zhu W, Pertea M, Allen J. E, Orvis J, White O, Buell CR, Wortman JR. Automated eukaryotic gene structure annotation using EVidenceModeler and the program to assemble spliced

alignments. Genome Biol. 2008;9:R7.

101. Huerta-Cepas J, Szklarczyk D, Heller D, Hernandez-Plaza A, Forslund SK, Cook H, Mende DR, Letunic I, Rattei T, Jensen LJ, et al. eggNOG 5.0: a hierarchical, functionally and phylogenetically annotated orthology resource based on 5090 organisms and 2502 viruses. Nucleic Acids Res. 2019;47:D309–D314.

102. McGinnis S, Madden TL. BLAST: at the core of a powerful and diverse set of sequence analysis tools. Nucleic Acids Res. 2004;32:W20–W25.

103. Tang H, Bowers JE, Wang X, Ming R, Alam M, Paterson AH. 2008. Synteny and collinearity in plant genomes. Science 320:486–488.

104. Nguyen LT, Schmidt HA, von Haeseler A, Minh BQ. IQ-TREE: A fast and effective stochastic algorithm for estimating maximum-likelihood phylogenies. Mol Biol Evol. 2015;32:268–74.

105. Emms DM, Kelly S. OrthoFinder: phylogenetic orthology inference for comparative genomics. Genome Biol. 2019;20:238.

106. Edgar RC. MUSCLE: multiple sequence alignment with high accuracy and high throughput. Nucleic Acids Res. 2004;32:1792–7.

107. Capella-Gutierrez S, Silla-Martinez JM, Gabaldon T. trimAl: a tool for automated alignment trimming in large-scale phylogenetic analyses. Bioinformatics. 2009;25:1972–3.

108. Yang ZH. PAML 4: Phylogenetic analysis by maximum likelihood. Mol. Biol. Evol. 2007;24:1586–91.

109. Ma YZ, Mao XX, Wang J, Zhang L, Jiang YZ, Geng YY, Ma T, Cai LM, Huang SQ, Hollingsworth P, et al. Pervasive hybridization during evolutionary radiation of *Rhododendron* subgenus *Hymenanthes* in mountains of southwest China. Natl. Sci. Rev. 2022;9:nwac276.

110. Mendes FK, Vanderpool D, Fulton B, Hahn MW. CAFE 5 models variation in evolutionary rates among gene families. Bioinformatics. 2020;36:5516–8.

111. Wu TZ, Hu EQ, Xu SB, Chen MJ, Guo PF, Dai ZH, Feng TZ, Zhou L, Tang WL, Zhan L, et al. clusterProfiler 4.0:A universal enrichment tool for interpreting omics data. Innovation. 2021;2:100141.

112. Wang YP, Tang HB, DeBarry JD, Tan X, Li JP, Wang XY, Lee TH, Jin HZ, Marler B, Guo H, et al. MCScanX: a toolkit for detection and evolutionary analysis of gene synteny and collinearity. Nucleic Acids

Res. 2012;40:e49.

113. Wang Dapeng, Zhang YB, Zhang Z, Zhu J, Yu J. KaKs_Calculator 2.0: a toolkit incorporating gamma-series methods and sliding window strategies. Genom Proteom Bioinf. 2010;8:77–80.

114. Zhang Z, Xiao JF, Wu JY, Zhang HY, Liu GM, Wang XM, Dai L. ParaAT: A parallel tool for constructing multiple protein-coding DNA alignments. Biochem Biophys Res Commun. 2012;419:779–881.

115. Yang FS, Nie S, Liu, H, Shi TL, Tian XC, Zhou SS, Bao YT, Jia KH, Gou JF, Zhao W, et al. Chromosome-level genome assembly of a parent species of widely cultivated azaleas. Nat Commun. 2020;11:5269.

116. Pond SLK, Frost SDW, Muse SV. HyPhy: hypothesis testing using phylogenies. Bioinformatics. 2005;21:676–9. doi:10.1093/bioinformatics/bti079.

117. Smith MD, Wertheim JO, Weaver S, Murrell B, Scheffler K, Pond SLK. Less Is More: An Adaptive Branch-Site Random Effects Model for Efficient Detection of Episodic Diversifying Selection. Mol Biol Evol. 2015;32:1342–53.

118. Murrell B, Wertheim JO, Moola S, Weighill T, Scheffler K, Pond SLK. 2012. Detecting Individual Sites Subject to Episodic Diversifying Selection. PLoS Genet;8:e1002764..

119. Pond SLK, Wisotsky SR, Escalante A, Magalis BR, Weaver S. Contrast-FEL-A Test for Differences in Selective Pressures at Individual Sites among Clades and Sets of Branches. Mol Biol Evol. 2021;38:1184–98.

120. Dobin A, Davis CA, Schlesinger F, Drenkow J, Zaleski C, Jha S, Batut P, Chaisson M, Gingeras TR. STAR: ultrafast universal RNA-seq aligner. Bioinformatics. 2013;29:15–21.

121. Li B, Dewey CN. RSEM: accurate transcript quantification from RNA-Seq data with or without a reference genome. BMC Bioinformatics. 2011;12:323.

122. Zhang JS, Zhang XT, Tang HB, Zhang Q, Hua XT, Ma XK, Zhu F, Jones T, Zhu XG, Bowers J, et al. Allele-defined genome of the autopolyploid sugarcane *Saccharum spontaneum* L. Nat Genet. 2018;50:1565–73.

123. Qiao X, Li QH, Yin H, Qi KJ, Li LT, Wang RZ, Zhang SL, Paterson AH. Gene duplication and evolution

in recurring polyploidization-diploidization cycles in plants. Genome Biol. 2019;20:38.

124. Katoh K, Standley DM. MAFFT multiple sequence alignment software version 7: improvements in performance and usability. Mol Biol Evol. 2013;30:772–80.

125. Price MN, Dehal PS, Arkin AP. 2010. FastTree 2--approximately maximum-likelihood trees for large alignments. Plos One 5:e9490.

126. Chen CJ, Chen H, Zhang Y, Thomas HR, Frank MH, He YH, Xia R. TBtools: an integrative toolkit developed for interactive analyses of big biological data. Mol Plant. 2020;13:1194–202.

127. Librado P, Vieira FG, Rozas J. BadiRate: estimating family turnover rates by likelihood-based methods. Bioinformatics. 2012;28:279–81.

Figure titles

Figure 1. Habitat and genomic characteristics of *R. nivale* subsp. *boreale*. A. habitat; B. habit; C. genome landscape, a, 52 pseudochromosomes, which belong to 13 homologous groups, and the length of the pseudochromosome; b, gene density; c, GC density; d, transposon element density; e, copia density; f, gypsy density; g, tandem repeat density; curved lines inside the circles link syntenic genes between different pseudochromosomes, the synteny between haplotype 1 and haplotype 2 is indicated in red, the synteny between haplotype 1 and haplotype 3 is indicated in green, the synteny between haplotype 1 and haplotype 4 is indicated in yellow. D. Hi-C heatmap for assembled pseudochromosomes; E. Smudgeplot analysis based on 21 *k-mers*.

Figure 2. Phylogenetic and comparative analysis between related species and haplotypes. A. Dot plot between *R. nivale* subsp. *boreale* and *R. ovatum*. B. Syntenic blocks between four haplotypes. C. phylogenetic relationships of Subsect. *Lapponica* based on the maximum likelihood (ML) analysis; yellow and green blocks show *R. nivale* and the sister clade of *R. nivale*, respectively; red block represents the data generated in this study (n1, n2, n3, n4, and *R. nivale* subsp. *boreale* represent the four haplotypes and transcriptome of *R. nivale* subsp. *Boreale,* respectively) and the blue block represents downloaded species data. D. Gene family characteristics between four haplotypes.

Figure 3. Comparative genomic analysis. A. ML phylogenetic tree showing the relationship between *R. nivale* subsp. *boreale* and 18 other species. Estimated divergence times (Mya, million years ago) are labeled at nodes in black. Bootstrap values are displayed on the nodes in circles (100%) and squares (≥ 95%). Expansion (orange) and contraction (blue) of gene families are shown on the branch, contraction and expansion of ancestors are represented by a pie chart, and extant species are indicated by numbers. WGD and WGT events are marked with D and T, respectively. B. A number of other orthologs, unique paralogs, multicopy orthologs and single-copy orthologs in 19 species. C. *Ks* of paralogs frequency distribution chart of seven species, namely, six Ericales (*Actinidia*, *Vaccinium* and Subg. *Hymenanthes*, Subg. *Furthermore*, Subg. *Rhododendron*, Subg. *Tsutsusi* one species each) and one *Vi. vinifera*, polyploidization events are represented by dotted lines. D. Homologous gene dot plots between *R. nivale* subsp. *boreale* and *Vi. vinifera*. The red box exemplifies the orthologous ratio of 1:2 between *Vi. vinifera* and *R. nivale* subsp. *boreale*.

Figure 4. KEGG and GO enrichment and gene duplication analysis of *R. nivale* subsp. *boreale*. A. KEGG (left) and GO (right) enrichment of genes in significantly expanded gene families. B. Venn diagram showing the number of shared and specific gene duplications between the significantly expanded genes (SEGs) and five categories of duplications (DSD, dispersed duplications; PD, proximal duplications; TD, tandem duplications; TRD, transposed duplications; WGD, whole genome duplications). C. *Ka/Ks* ratios of the five types of duplications. D. KEGG pathway enrichment analysis of the five duplication types.

Figure 5. Single-matched allelic expression analysis. A. The total amount of single-match allelic expression of 52 pseudochromosomes; the colors represent 13 homologous groups (HGs). B. Heatmap clustering analysis of single-match alleles in screening the position of DELs in *R. nivale* subsp. *boreale*. Each row represents a set of differentially expressed alleles, and each column represents a chromosome. The heatmap shows a homologous group.

Figure 6. Identification and evolution of key family and genes for adaptation to low mountaintop temperature and hypoxia. A. Rootless ML phylogenetic tree based on ultrafast 1,000 bootstrap samplings showed diversified AP2/ERF superfamily in 13 species, including 10 *Rhododendron* species and *Ar. thaliana*, Kiwifruit, *Va. darrowii.* The color of the clades indicates five subfamilies of the AP2/ERF superfamily. The labels are differently colored according to species. B. Schematic diagram of the gain and loss of key genes in 12 species of Ericales; numbers in pink and blue depict *ERF VII* and *CBF* gene family turnover. The numbers in the rectangles and circles represent the number of genes in ancestral and existing species. The + and − signs represent the gain and loss of genes, respectively. C. Expression levels of *ERF VIIs* and *CBFs*. The numbers in parentheses indicate the number of genes that are expressed. P represents the adjusted P value.

Figure 7. Characteristics of Ericales Cytochrome P450 (CYP). A. ML phylogenetic tree showing the relationship between 10 CYP clans (higher order groupings of CYP families). B. Heatmap showing the number of clan members for each species. C. Phylogenetic tree of the CYP members of 13 species based on GTR (generalized time-reversible). Different clans are represented by different colors. D. Number of CYP genes produced by duplication events in 10 species of *Rhododendron*.

Figure 1

Click here to access/download;Figure;Fig1.pdf

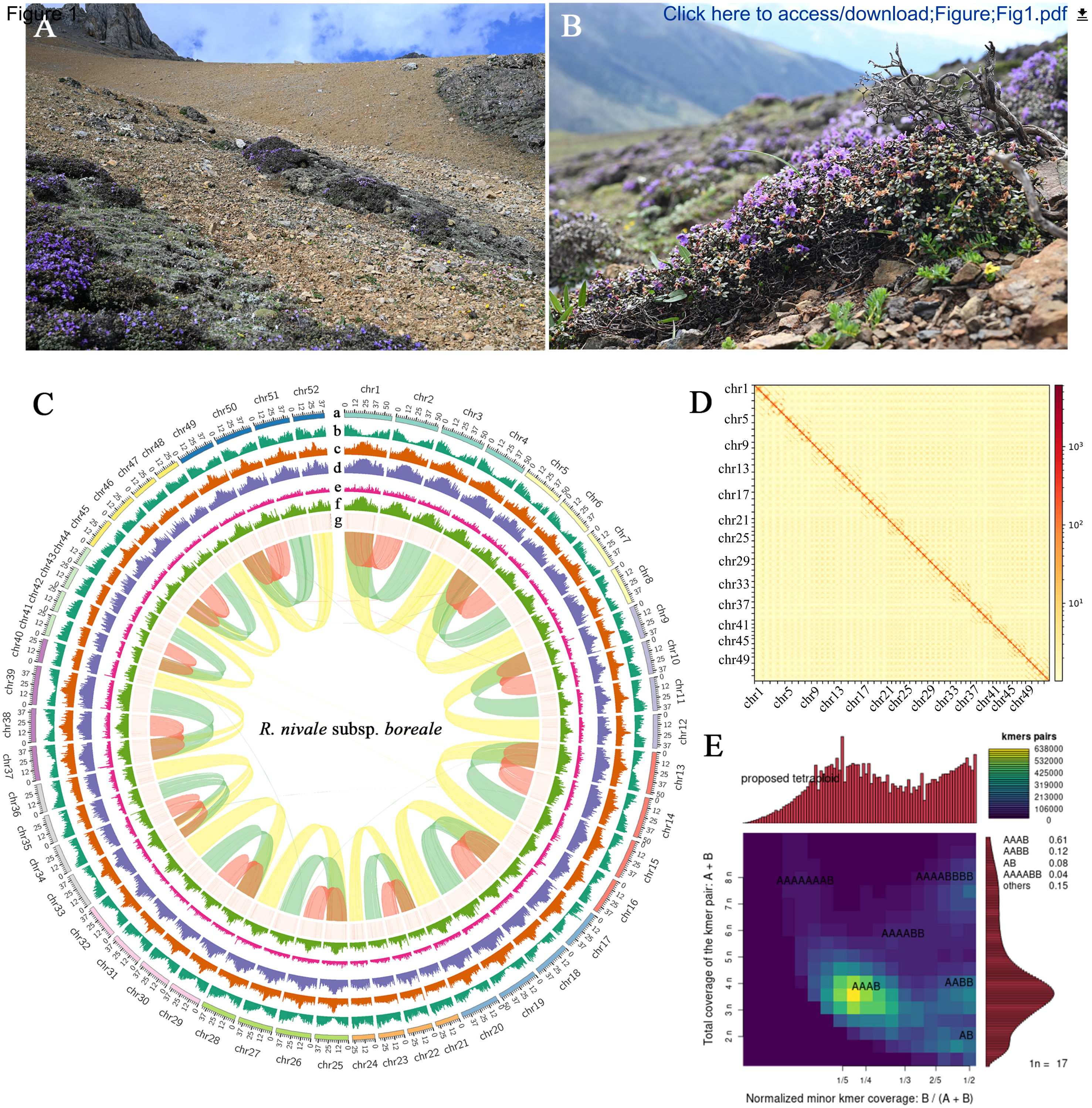

Click here to access/download;Figure;Fig2.pdf

Figure 2

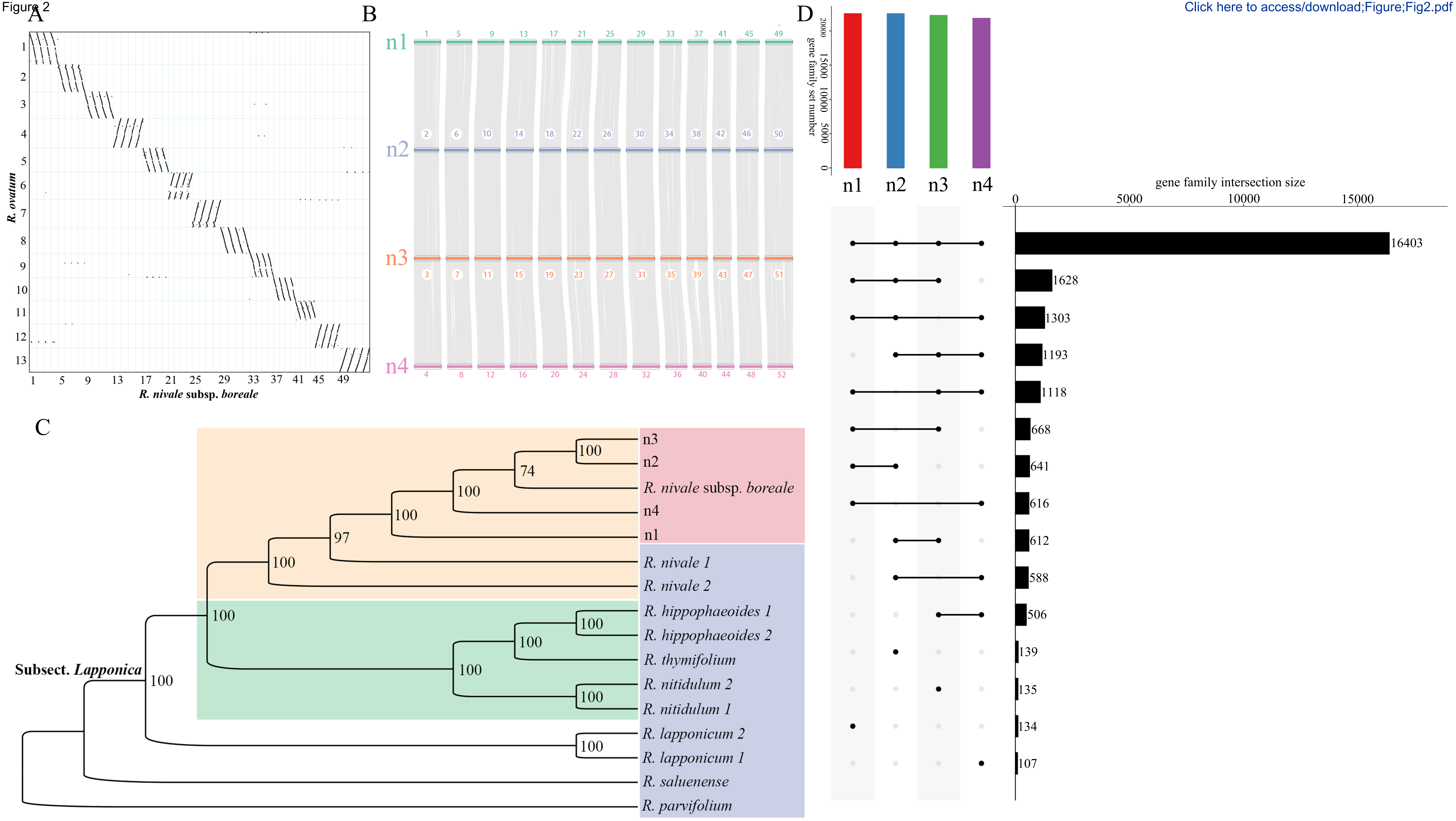

Figure 3

Click here to access/download;Figure;Fig3.pdf

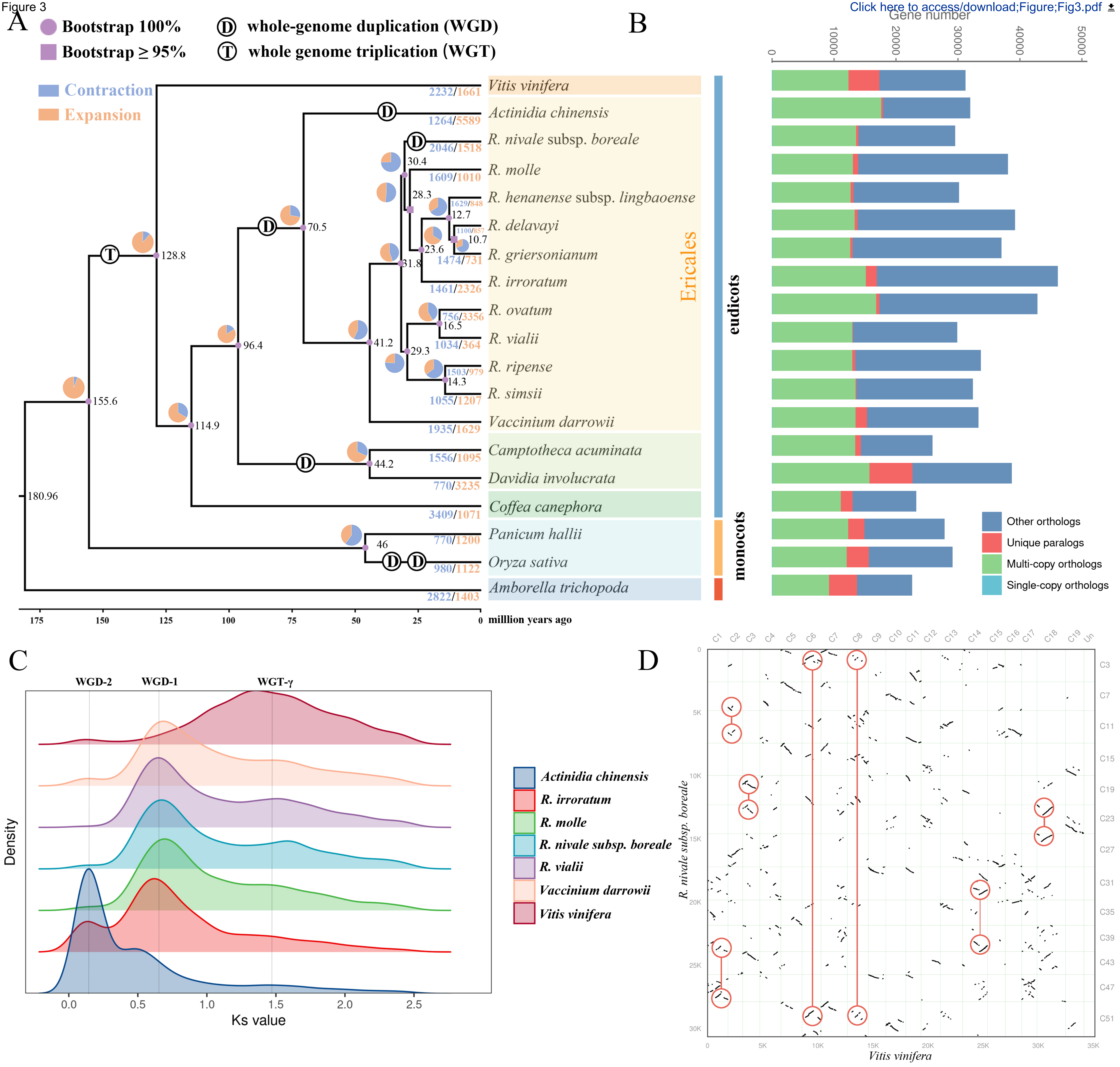

Figure 4

Click here to access/download;Figure;Fig4.pdf

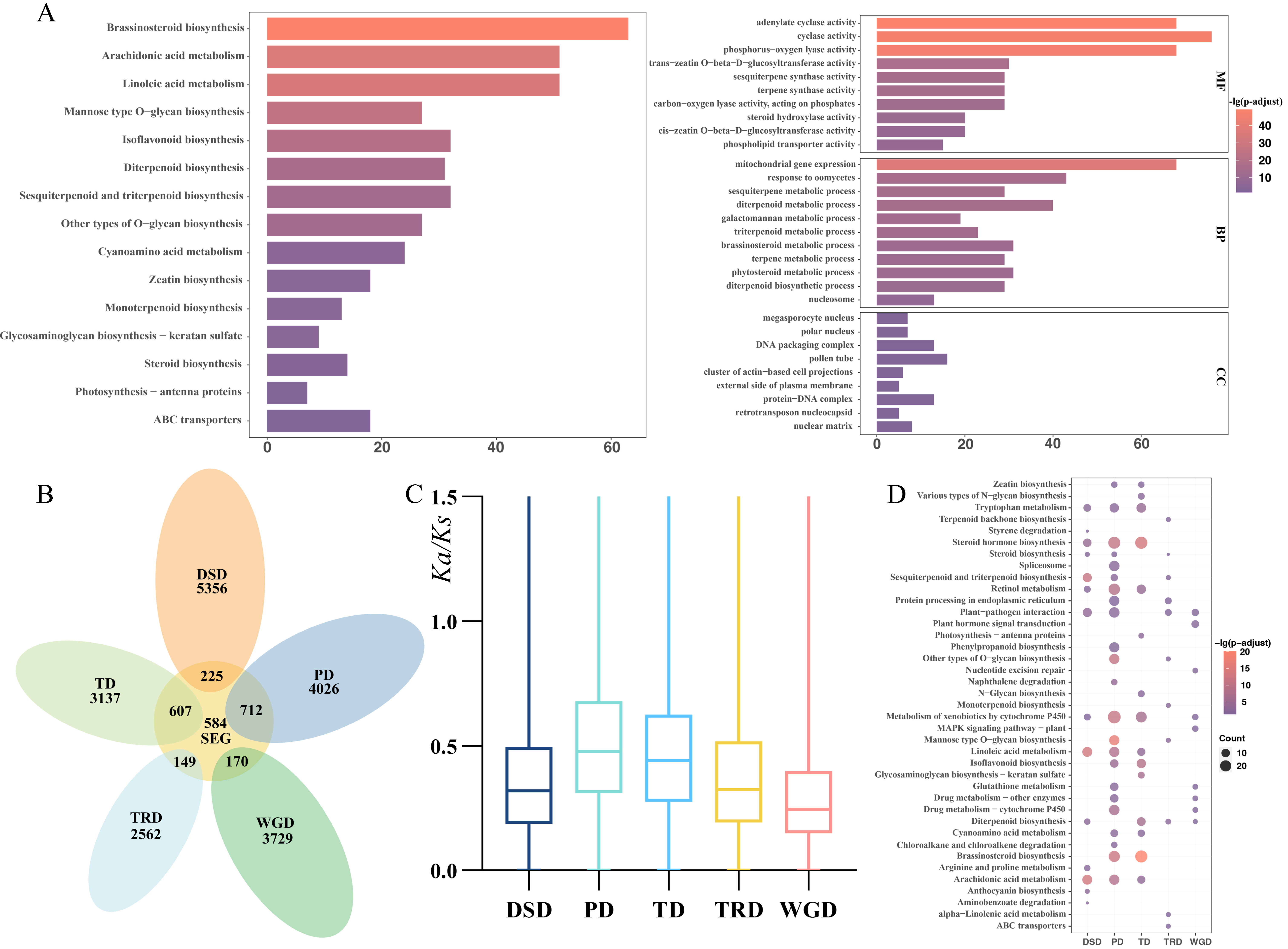

Figure 5

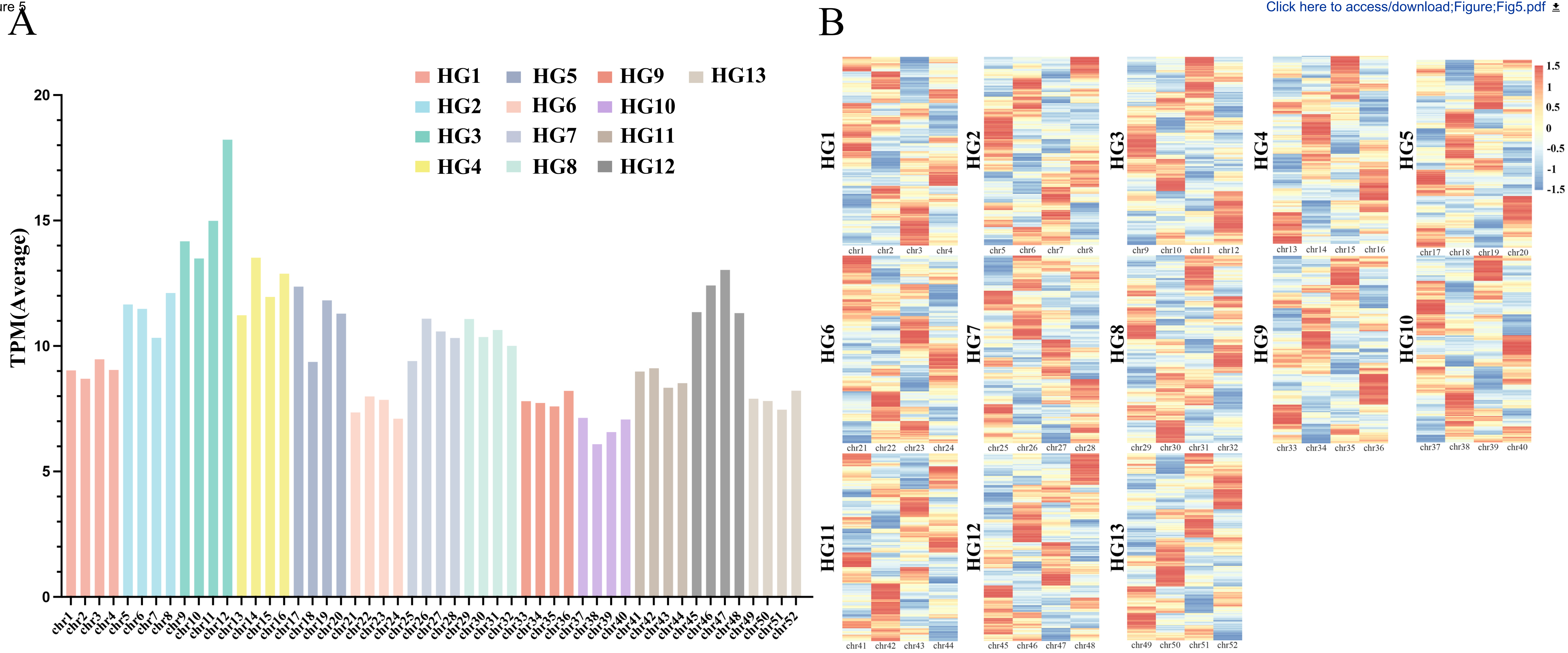

Click here to access/download;Figure;Fig5.pdf

Figure 6

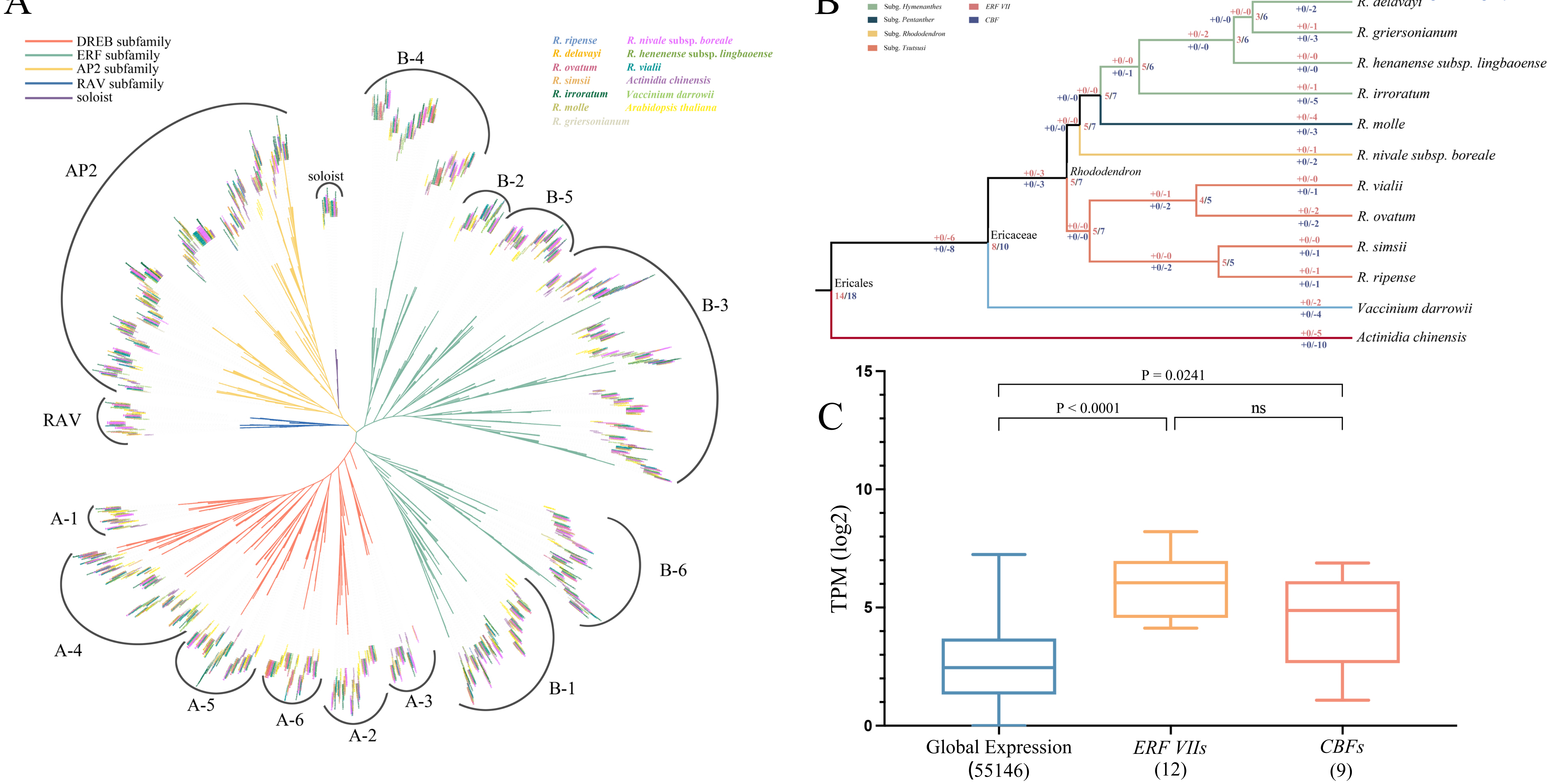
A
DREB subfamily
ERF subfamily
AP2 subfamily
RAV subfamily
soloist
R. ripense
R. delavayi
R. ovatum
R. simsii
R. irroratum
R. molle
R. griersonianum
R. nivale subsp. boreale
R. henenense subsp. lingbaoense
R. vialii
Actinidia chinensis
Vaccinium darrowii
Arabidopsis thaliana
AP2
soloist
B-4
B-2
B-5
B-3
RAV
A-1
A-4
A-5
A-6
A-2
A-3
B-1
B-6
B
Subg. Hymenanthes
Subg. Pentanther
Subg. Rhododendron
Subg. Tsutsusi
ERF VII
CBF
R. delavayi
R. griersonianum
R. henanense subsp. lingbaoense
R. irroratum
R. molle
R. nivale subsp. boreale
R. vialii
R. ovatum
R. simsii
R. ripense
Vaccinium darrowii
Actinidia chinensis
Rhododendron
Ericaceae
Ericales
14/18
8/10
C
TPM (log2)
P = 0.0241
P < 0.0001
ns
Global Expression (55146)
ERF VIIs (12)
CBFs (9)

Figure 7

Click here to access/download;Figure;Fig7.pdf

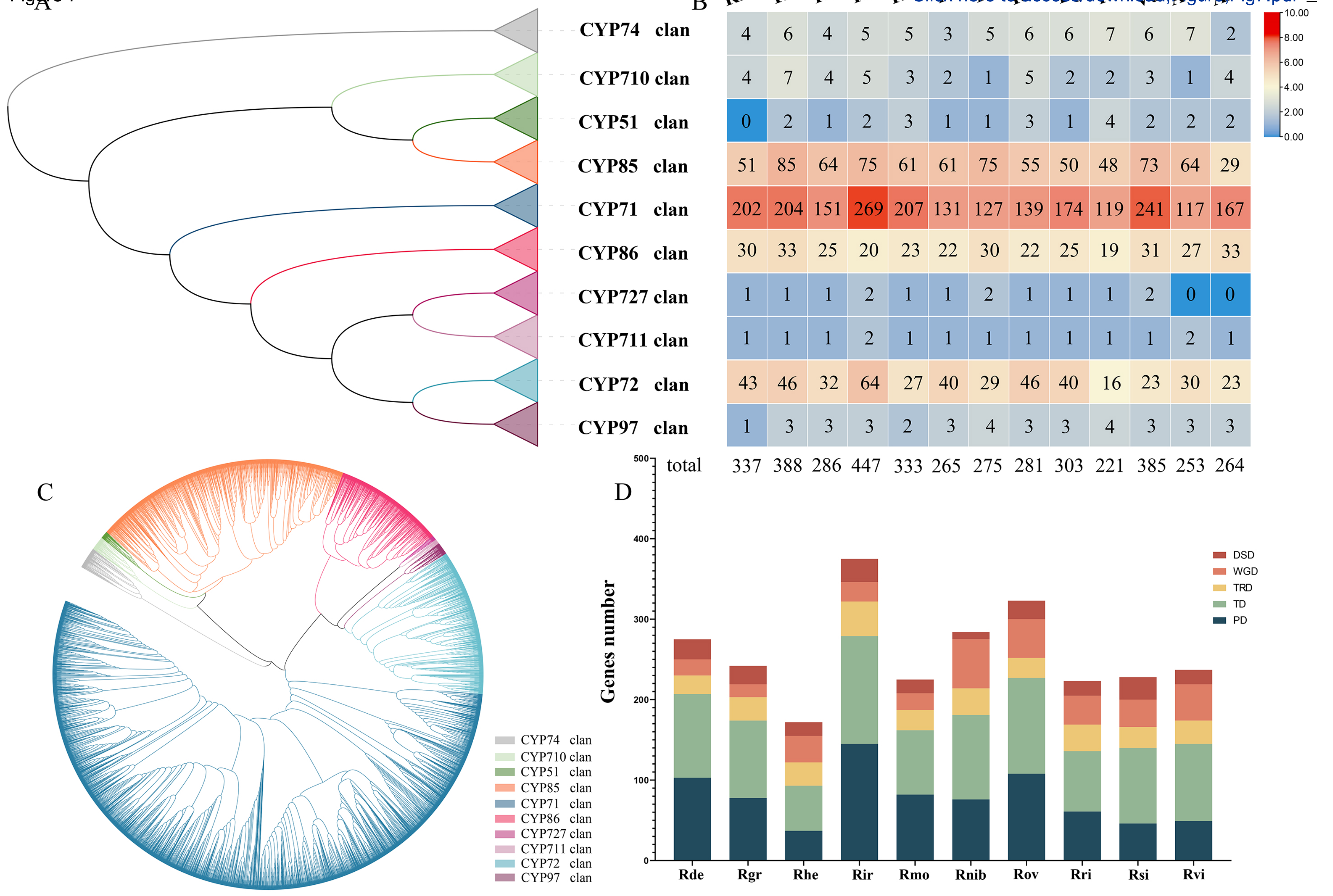

Supplementary Figure

Click here to access/download
Supplementary Material
Supplementary Figure.docx

Supplementary Table

Click here to access/download
Supplementary Material
Supplementary Table.xls

Personal Cover
Click here to access/download;Personal Cover;Respond_to_editor_and_reviewers.docx

April 25, 2024

Dear Editor and Reviewers,

Thank you for your email and the opportunity to revise our manuscript, "The first high-altitude autotetraploid haplotype-resolved genome assembled (*Rhododendron nivale* subsp. *boreale*) provides new insights into mountaintop adaptation" (GIGA-D-23-00395).

These comments are valuable and helpful for revising and improving the quality of our manuscript, as well as providing important guidance for our future research. We have checked the manuscript and have tried our best to revise it carefully according to these comments. The main changes are as follows:

(1) We have entrusted our paper to a professional institution for English language polishing to improve the language and logic of the manuscript.

(2) We have emphatically adjusted the tone to show that the alpine adaptation strategy in the manuscript is speculative and needs to be verified by further biological experiments.

(3) We found that a small portion of positive selection genes was recorded incorrectly, and we are very sorry about that. To identify the positive selection signal more accurately, we have updated the detection approach. In the revision, a multi-model cross-validation strategy was used to detect positive selection genes. Although the results produced by this approach are slightly different, the overall conclusion has not changed.

(4) We have also changed the submission section to DATA NOTE to emphasize that our main contribution is the provision of an important alpine plant genetic resource.

We have marked the changes with different colors in the revised manuscript to facilitate your review of the changes (Blue: Modifications based on review comments; Green: English grammar, logic and other modifications). We earnestly appreciate the thorough work of the Editors/Reviewers, and sincerely hope that you find our responses and modifications satisfactory. Thank you again for considering our work. We hope that this version of our manuscript is suitable for publication in GigaScience.

Best regards

Sincerely yours,

Shi-Kang Shen

Reviewer #1:

Thank you for your thorough evaluation of our manuscript and for the constructive comments provided. We appreciate the time you've taken to review our work. We acknowledge the issues you have raised regarding the clarity of language and the logical flow of the manuscript. We understand that these are crucial for effectively communicating our research and ensuring that the scientific community can fully assess the quality and rigor of our work. To address your concerns, we made a complete revision of the manuscript:

Major Concern:

Comment: The paper is not yet ready for publication and requires a lot of writing improvement work to clarify the analyses and results.

Response: Thank you for your comment. For editors and reviewers to effectively evaluate our research, we conducted a careful examination of the manuscript, corrected typos, and improved the logic of the full text. In addition, we entrusted the manuscript to a professional agency to improve its readability.

Comment: I find that many of the claims that the authors suggest (especially concerning gene family expansion and contraction) are strongly worded and at the very least need to be toned down since they are only correlative at best.

Response: Thank you for your comment. We also realize that our study is preliminary and only speculates on high-altitude adaptation strategies. Therefore, we moderate our tone and show in the paper that our studies on adaptive strategies are speculative. In addition, we change the study to "Data Note", showing that our study mainly provides an important genetic resource of high-altitude woody polyploid. We appreciate your attention to detail and guidance in enhancing the manuscript's quality.

Other Concern:

Comment: Line 28 - the context and importance of *R. nivale* as the "first high-altitude woody flowering autotetraploid." Is missing. Why should the reader care about this

very specific case study. I think some better lead up will help understand.

Response: Thank you for your comment. We have added a leading-in to the background of the abstract to make it easier for readers to quickly understand the issues of concern in this article. “As a high-altitude woody polyploid, this species occupies a distinctive role in the exploration of alpine plant adaptability.” (Line 24-25)

Comment: 30 - "harbor" change to "which harbor" or "harboring"

Response: Thank you for pointing out the mistake. We have revised the sentence to include “harboring” to properly connect the relative clause, providing a more precise and readable statement. (Line 33)

Comment: 37 - ERF VII are undefined yet as a gene class.

Response: Thank you for your comment. Group VII ERFs is a phylogenetic cluster, which is conserved across angiosperms. We acknowledge that the *ERF VII* gene class has not been explicitly defined in the current. However, *ERF VII* is a widely used gene group (Abbas et al. 2022; Fan et al. 2023; Zubrycka et al. 2023). Our research is predicated on the homology with the well-studied and defined *ERF VII* gene family in *Arabidopsis thaliana*. The proposed *ERF VIIs* is based on sequence similarity and functional characteristics of these known genes, particularly their role in response to hypoxic conditions.

Reference:

Abbas M, Sharma G, Dambire C, et al. An oxygen-sensing mechanism for angiosperm adaptation to altitude. Nature. 2022;606:565–9.

Fan B, Liao K, Wang LN, et al. Calcium-dependent activation of CPK12 facilitates its cytoplasm-to-nucleus translocation to potentiate plant hypoxia sensing by phosphorylating ERF-VII transcription factors. Mol Plant. 2023;16:979–98.

Zubrycka A, Dambire C, Carbonare DL, et al. ERFVII action and modulation through oxygen-sensing in Arabidopsis thaliana. Nat Commun. 2023;14:4665.

Comment: 38 - meanwhile is not the best connector word here and elsewhere.

Response: Thank you for pointing out the problem of improper use of connective words. To improve the readability of the full text, we have checked the connectives in the full text and modified them accordingly according to the context.

Comment: Line 50- what do you mean by core genus. Provide a better definition.

Response: Thank you for pointing out the problem about definition. In the reworked manuscript, we have replaced "core genus" with "largest genus". (Line 55)

Comment: Line 58-62- (1) what do we know about high-altitude polyploids in general? (2) Is there any evidence for adaptive benefits? (3) Why should we care?

Response: Thank you for your comment. All information related to polyploids is presented in the third paragraph of the introduction. (1) Following polyploidy events, rapid shifts in gene expression and epigenetic modifications can bestow the polyploid with an almost instant competitive edge. (2) Although the evidence of polyploid high altitude adaptation is not completely sufficient, many studies have proved that polyploid plants have stronger abiotic stress tolerance than diploid plants. Therefore, polyploidy tends to be ecologically advantageous and occurs in variable climatic regions, such as the Qinghai-Tibet Plateau alpine and Pan-Arctic regions. (3) Comprehending these adaptive mechanisms in high-altitude polyploids not only illustrates evolutionary dynamics but also informs conservation methodologies. (Line 86-100)

Comment: Line 78-79 : Limited is repeated twice

Response: Thank you for your comment. We have modified the sentence to solve the problem of word redundancy. "Currently, the assembly of autopolyploid genomes presents significant challenges, resulting in the publication of only a select number of such genomes, including those of *Medicago sativa*, *Saccharum spontaneum*, *Solanum tuberosum*, and *Rheum officinale*." (Line 81-84)

Comment: 85 - polyploidy "event"

Response: Thank you for your carful checks. We have modified in manuscript. (Line 88)

Comment: 113- poor grammar and structure "Tetraploid was identified from the *kmers* present in sequencing reads (Fig. 1E)."

Response: Thank you for pointing out the problem. We have modified this sentence to "This species identified as a tetraploid based on the *k-mers* analysis." (Line 117-118)

Comment: 116 - great that you tried different assembly approaches. I think you should report some contig level statistics to show why you chose hifiasm. Minimum should report contig N50.

Response: Thank you for your recognition of our analytical methods. The results for each assembly approach are shown in Table S4.

Comment: 137 - LAI of reference suggests that the repeat content of the assembly is not well assembled. This can be improved by using HiFi pacbio reads. Although I understand that may not be in the scope of this manuscript however that would be an improvement for the assembly.

Response: Thank you for your suggestion to improve the assembly using HiFi PacBio reads. We agree that this approach could potentially enhance the assembly quality However, due to the extensive time and resources required for such an analysis, which includes not only closing gaps but also a complete re-run of all subsequent analyses, we are unable to include this in the current scope of our manuscript. To assess LAI more accurately, we have evaluated each haplotype independently, thereby reducing the negative impact of highly similar sequences between haplotypes on LAI regulation. The results showed that LAI of all haplotypes was greater than 14 (n1:14.78, n2:14.84, n3:14.35, n4:14.38). (Line 140-142) We have noted your recommendation as a valuable suggestion for future improvements to our assembly and will consider it for subsequent iterations of our work.

Comment: 159-163 the whole section about confirming the polyploidy using kmers should be explained better.

Response: Thank you for your comment. We have shown in the methods section how to determine auto and allotetraploid based *kmers* analysis. (Line 549-550). For more specific principles, please refer to the citation "[29] Ranallo-Benavidez TR, Jaron KS, Schatz MC. GenomeScope 2.0 and Smudgeplot for reference-free profiling of polyploid genomes. Nat Commun. 2020;11:1432."

Comment: 167 - I am not familiar with the term "exerted synteny" I don't think it is being used the correct way.

Response: Thank you for your comment. Upon check, we agree that the term 'exerted' may not be the most appropriate in this context. We have revised the manuscript accordingly. The text now reads: "As expected, the dot plot and syntenic blocks indicated synteny among the four haplotypes (Fig. 2A), with 20,172 gene pairs showing synteny between haplotypes 1 and 2, 20,249 between haplotypes 2 and 3, and 19,883 between haplotypes 3 and 4." (Line 169-172)

Comment: 169 - clarify what transcriptome data are being used here.

Response: Thank you for your comment. We provided relevant information of all the data in the manuscript in the Table S14, including species, data type, accession number and download address. The data generated in this study (n1, n2, n3, n4, and *R. nivale* subsp. *boreale* represent the four haplotypes and transcriptome of *R. nivale* subsp. *boreale*, respectively), are shown in Figure 2 title. (Line 969-971)

Comment: 214- incomplete sentence "In the dot plot between *Vi. vinifera* and *R. nivale* subsp. *boreale* (Fig. 3D, S7-8)."

Response: Thank you for pointing out the incomplete sentence. The sentence should be completed as follows: "The dot plot comparing *Vi. vinifera* and *R. nivale* subsp. *boreale* (Fig. 3D, S7–8), nearly every grape chromosome exhibited two highly compatible chromosomal regions in *R. nivale* subsp. *boreale* (orthologous ratio 1:2)."

(Line 215-217).

Comment: 217 - the whole section on positive selection is not written well and was challenging to understand. Thus, the claims derived from these analyses are not well supported at this stage. There is no introduction to the section just an immediate dive into 44 genes. The reader is not prepared for that yet.

Response: Thank you for your valuable feedback regarding the section on positive selection. We apologize for any confusion caused by our initial presentation of the data and analysis. We acknowledge that the high-altitude adaptation mechanisms of *R. nivale* subsp. *boreale* cannot be fully resolved based on current data. In this manuscript, our aim is to provide important high-altitude plant genomes and speculate the high-altitude adaptation strategies of *Rhododendron* based on existing data, providing a scientific basis for future adaptation studies. To detect the true positive selection events as much as possible, we have updated the positive selection gene recognition pathway. On the basis of aBSREL detection, the Clade Model was used to exclude genes that may not be affected by selection pressure. Finally, the positive selection sites were identified based on the intersection of the MEME and the Contrast-FEL. A gene is considered a positive selection gene when it meets all model criteria. The multi-method detection approach reduces the probability of false positive gene identification. Finally, the results of the positive selection analysis from KaKs_Calculator are integrated to collectively speculate on the adaptation mechanism. Additionally, we have revised this section to improve clarity and ensure that the narrative is more accessible. I appreciate the opportunity to enhance the quality and rigor of our manuscript. (Line 219-234; 581-604; Tables S18-S20)

Comment: 230 - 233 - can be moved to discussion

Response: Thank you for your suggestion. We very much agree with this adjustment and have moved this section to the discussion section. (Line 369-374)

Comment: 282 - "More species had approximately 140 genes." Unclear sentence and

context

Response: Thank you for comment. The intent of this statement was to convey that a greater number of species were found to have the gene count of AP2/ERF close to 140 in our study. To clarify this, we have revised the sentence to read: "The majority of species in our study had gene counts of approximately 140." (Line 283-284)

Comment: 291-295 - the whole section is missing citations and could be moved to the discussion.

Response: Thank you for your constructive feedback. We have added the appropriate references to ensure the arguments are well-founded. Additionally, we agree that the content may be better suited to the discussion part of the paper and have made the necessary adjustments to relocate the section. (Line 435-437)

Comment: 303 - citations.

Response: Thank you for your careful check. We have added the appropriate citation. The following revisions have been made to the manuscript: "Group VII ethylene response factor transcription factors (ERF VIIs) are associated with altitude adaptation [34]." (Line 291-292)

Comment: 307-310 conclusion is very strong for the evidence presented.

Response: Thank you for your comment. We understand your concern regarding the strength of the conclusion drawn from the observed gene expression. We would like to clarify that the high expression levels of these genes of *R. nivale* subsp. *boreale* are consistent with the plant's adaptation to its natural habitat, which is characterized by low oxygen pressure. This inference is supported by the literature indicating that ERF VII genes probably are involved in the response to hypoxic conditions. However, we acknowledge that further experimental validation is needed to definitively establish the causal relationship. Therefore, we have moved it to discussion section and softened the language and said it was subject to experimental verification. We have revised the manuscript as follows: "The high expression levels of *ERF VIIs* in *R. nivale* subsp.

boreale suggests a potential adaptation to low oxygen environments, which warrants further experimental investigation." (Line 441-443)

Comment: 324 - typo : badiratE

Response: Thank you for pointing out the typo. We have corrected it. (Line 316)

Comment: 332- the distribution of the CYPs is shown? Reference a figure.

Response: Thank you for your comment. We have added the appropriate figures (S10-21), which visually represent the distribution of the AP2/ERFs, CYPs, *ERF VIIs* and *CBFs* in *R. nivale* subsp. *boreale*.

Comment: 336 - missing citations.

Response: Thank you for pointing out the missing citation. We have added the appropriate citation. (Line 330)

Comment: Overall the claims about positive selection of genes for altitude fitness should be toned down. The functional validation of the suggested genes is not clear and while the analysis might show they are under selection the role and fitness advantage is not supported by evidence presented herein.

Response: Thank you for your comment on genes for altitude fitness. We acknowledge that our results are preliminary and appreciate your suggestion to moderate the language used to describe these findings. To enhance the accuracy of analysis, we have modified our strategy of positive selection analysis to include multiple models for cross-validation, namely two branch site-based models (aBSREL and Clade Model) and two site-based models (MEME and Contrast-FEL). Additionally, we have revised the tone of the conclusion to reflect that it is a conjecture based on existing results, pending further experimental validation. Our aim is to provide valuable genetic resources and to formulate initial hypotheses regarding the high-altitude adaptation mechanisms in *R. nivale* subsp. *boreale*. In our latest revision, we have reclassified the submission as a DATA NOTE to emphasize that the primary contribution of our research is the provision

of significant high-altitude genetic resources.

Comment: 431- what is anti-low temperature

Response: Thank you for your comment. We have modified it to "cold tolerance". (Line 444-445)

Comment: 444- CFBs are lowly expressed? What are the conditions of sampling? These are cold response genes.

Response: Thank you for bringing this to our attention. We recognize that the expression of *CBFs* is typically induced by cold conditions, and therefore, their low expression under the conditions sampled may not fully reflect their role in cold response. Therefore, we have deleted this section.

Comment: 510 - what is this analysis for Modifit?

Response: We are sorry for our careless mistakes. Thank you for your reminder. The correct name for the software is ModFit LT 5.0, and its download address has been added to the manuscript. (Line 511) ModFit LT is the open-ended modeling software for cytometry data. We use ModFit LT to process flow cytometry results (Fig. S1). (Line 506-507)

Comment: 538 - Clarify the steps you took to annotate. which transcripts were used where and for what software downstream.

Response: Thank you for your comment. We have rewritten the section based on transcriptome annotations. During our annotation, the transcriptome data were assembled using Trinity and StringTie. Next, all assembly results were fed into BRAKER3 and PASA for gene prediction and to generate Ab initio gene predictor training sets. We have made the subsequent changes to the manuscript: "In our transcriptome-based strategies, we used HISAT2 v2.2.1 [92] to align clean reads of the transcriptome with the genome. Trinity v2.14.0 [93] and StringTie v2.2.1 [94] were used to assemble transcripts. BRAKER3 [95] and PASA v2.5.2 [96] were used to

predict gene structure based on the assembled transcripts and to generate ab initio gene predictor training sets.” (Line 534-538)

Comment: 553 - You said you used smudgeplot at the top of the section not genomescope.

Response: Thank you for your comment. Genomescope2 and Smudgeplot are published in the same article, but they are completely different pipeline. Genomescope2 was used to estimate genome size and heterozygosity, and Smudgeplot was used to estimate genome ploidy. We used Genomescope2 to obtain an estimate of genomic heterozygosity (Table S3) and Smudgeplot to evaluate ploidy (Fig. 1E).

Comment: 556- JCVI has a better citation than the zenodo repo

Response: Thank you for your recommendation to provide a better citation for JCVI. We have updated the citation to reflect the primary literature associated with the JCVI utility libraries. The revised citation is as follows: “103. Tang H, Bowers JE, Wang X, Ming R, Alam M, Paterson AH. 2008. Synteny and collinearity in plant genomes. Science 320:486–488.”

Comment: 615- BadiTate typo

Response: Thank you for pointing out the typo. We have corrected it. (Line 631)

Comment: 629 - clarify the author contributions what does "Investigation" mean.

Response: Thank you for your comment. In this study, in addition to bioinformatics analysis, cytological experiments were used to estimate genome size (Fig. S1) and chromosome number. Gao-Ming Yang, Jie-Yu Zhang and Wen-Guang Sun provided experimental sites, reagents, and conducted experiments. Unfortunately, *R. nivale* subsp. *boreale* has a lot of small chromosomes, and despite numerous experiments, we have not yet obtained the desired results. We also attach a chromosome count below, indicating that the number of chromosomes is much higher than that of the diploid *Rhododendron*. We have modified “Investigation” to “Cytological experiment.” (Line

642-644)

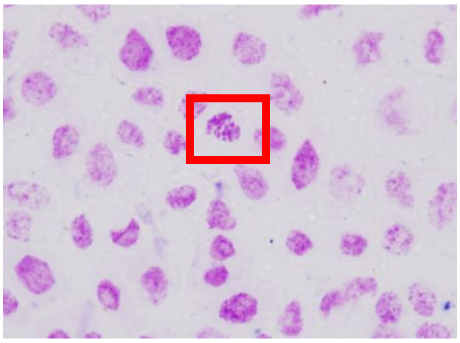

R. nivale subsp. *boreale* chromosomes

Reviewer #2:

Thank you for your recognition of our work. Your suggestions are very important to us, both for improving the manuscript and our further research. We have studied these comments carefully and have made corrections as possible as we can. We hope these corrections could meet with approval. The point-by-point response is as follows:

Comment: Scientific names: *Arabidopsis* has been named as "*Ar. thaliana*" throughout the paper should be "*A. thaliana*".

Response: Thank you for your suggestion. We used *Ar. thaliana* to distinguish *Ac. chinensis*. Using the same abbreviation is likely to cause confusion.

Comment: Line 141: The completeness of protein coding genes were accessed by BUSCO. This is a good metric to access completeness, however sometimes BUSCO does not give us the complete picture. Hence I suggest, adding mono-exonic (single-exon) to multi- exonic (multiple-exon) ratio in the needed for further justification and support.

Response: Thank you for your valuable feedback. To address this, we calculated the ratio (0.245) of mono-exonic (single-exon) to multi- exonic (multiple-exon) genes and have added this information to the manuscript (Line 148-149). Additionally, we have provided more details about BUSCO in Table S8. This table contains BUSCO assessment results for different assembly approaches, different haplotypes, unitigs and protein-coding genes.

Comment: Line 289: The authors mention variable alpine climate for growth but do not provide any metric data related to the statement. Growing conditions with metrics like average temp difference, oxygen level and average pressure needs to be provided for better context.

Response: Thank you for your valuable feedback. We have added relevant metrics for alpine climate, including average annual temperature, air pressure and diurnal temperature differences, which are presented in the discussion section. The revised text

is as follows: “Low temperature (average annual temperature below 0 °C), low partial pressure of oxygen (for every 1,000 m increase in altitude, air pressure drops by about 11%) and rapid weather changes (annual average diurnal temperature exceeding 20 °C) are the main factors limiting alpine plant survival.” (Line 422-425)

Comment: Line 303: "Previous studies have shown that *ERF VIIs* play an extremely important role in adapting to high-altitude environments" This statement needs citation for support.

Response: Thank you for your carefully checks. The following revisions have been made to the manuscript: “Group VII ethylene response factor transcription factors (ERF VIIs) are associated with altitude adaptation [34].” (Line 291-292)

Comment: In the discussion section author has discusses in great detail about different pathways regulating different morphological traits. But a section describing the morphological traits and the phenotypic adaptation of *Rhododendron nivale* subsp. *boreale* is necessary to better relate these pathways in the contest of this species being studied.

Response: Thank you for your insightful suggestion. We agree that a section detailing the morphological traits and phenotypic adaptations of *R. nivale* subsp. *boreale* would greatly enhance the discussion. Consequently, we have speculated potential altitudinal adaptation traits in *R. nivale* subsp. *boreale* from existing studies and have incorporated this information into the manuscript. These characteristics include plant height, leaf size, flowering changes, and seed size. We have updated the manuscript as indicated below: “In alpine environments, plants have evolved myriad morphological and physiological adaptations to contend with the rigor of high-altitude conditions [50]. *R. nivale* subsp. *boreale* native to mountaintops, typically reaches heights of less than 30 cm, with leaves that seldom exceed 5 mm in both length and width, exhibits delayed flowering, and produces seeds that are nearly indiscernible. These characteristics are thought to be a response of *Rhododendron* to low temperatures at high altitudes, poor nutrition, and extremely short growth cycles [51].” (Line 408-413)

Comment: Line 444: Why CBFs and its transcription factors are relatively low expressed or absent? What does the author thinks is the reason? Further justification maybe necessary.

Response: Thank you for your comment. We are very sorry for ignoring *CBFs* as a class of cold response genes. The expression level of *CBFs* was closely related to our sampling conditions. It is possible that the gene expression observed may be influenced by various factors, such as the ambient temperature during sampling and the gene-dosage effect. Given these considerations, we cannot make reasonable speculation, and therefore, we have removed this section from the discussion.

Reviewer #3:

We would like to thank you for your professional review work, constructive comments, and valuable suggestions. Your insights have been instrumental in enhancing the quality of our research, and we have carefully considered each point to ensure our work meets high standards. We have revised our manuscript to clarify our claims and enhance the overall clarity of our work. We are confident that the revisions made will address your concerns. Followed by a point-by-point response:

Major Concern:

Comment: The authors logically and comprehensively describe their evidence to suspect these genes and pathways with relevant literature cited throughout and I applaud them for that. However, the evidence is not strong enough to support these final claims. They should be worded along the lines of, "we suspect these loci enabled the success of this species in high altitudes", or something of the sort rather than claiming they aid in survival or adaptability without having explicitly tested that fact.

Response: Thank you for acknowledging the logical structure and analytical comprehensiveness of our evidence presentation. We appreciate your suggestion to moderate the language used to describe the influence of the identified loci on the success of the species at high altitudes. We agree that without explicit testing, it is more appropriate to use tentative language. Therefore, we have revised our claims to reflect that these loci are suspected of contributing to the species' success in high-altitude environments.

Other Concern:

Comment: Line 37: "Notably, highly expressed *ERF VIIs* aid survival in hypoxic mountaintop. Meanwhile, the extended families enriched in brassinosteroid biosynthesis, which enhanced adaptability to alpine weather and probably mediated by increased cytochrome P450 genes."

The authors present no experimental evidence for this claim. They did not explicitly test survival under hypoxic conditions, nor any physiological response to alpine weather.

Response: Thank you for your comment. We agree that all of these assumptions need to be tested experimentally. Therefore, we have revised the relevant sections to clarify that these are hypotheses based on gene expression patterns and known functions of these genes and gene families. The revised text now reads: "We identified extended gene families and signatures of positive selection that are involved not only in adaptation to the mountaintop ecosystem (response to stress and developmental regulation) but also in autotetraploid reproduction (meiotic stabilization). Additionally, the expression levels of the *ERF VIIs* were significantly higher than the mean global gene expression. We suspect that these changes have enabled the success of this species at high altitudes." (Line 38-43)

Comment: Line 100: "and determined which genes or gene families play a role in alpine adaptation and the survival of polyploids"

Qualifying language required that you have well supported candidates but do not know if they directly play a role in adaptation or survival without testing for these phenotypes.

Response: Thank you for your comment. We acknowledge the necessity of qualifying our language regarding the role of specific genes or gene families in alpine adaptation and the survival of polyploids. We have revised to reflect that, while we have identified well-supported candidate genes, their direct involvement in adaptation or survival remains to be empirically tested. The revised statement now reads: "Based on this assembly, we identified polyploid types, deciphered whole genome duplication (WGD) events, and investigated which genes or gene families are potential candidates involved in alpine mountaintop adaptation and the survival of polyploids." (Line 103-106)

Comment: Line 114: "The three initial assembly sizes were 2.48 Gb, 2.39 Gb and 2.40 Gb, which were assembled by Hifiasm, Canu v1.9 and Hicanu, respectively (Table S4). The assembled version of Hifiasm was used for subsequent analysis because it has higher integrity in genes and LTRs.

Thank you for providing some negative results! This helps benchmark software performance and gives the field some insight to how different assemblers handle

autopolyploids.

Response: Thank you for your positive feedback. We are glad to contribute to this knowledge base.

Comment: Line 173: "A clade containing all four haplotypes of *R. nivale* subsp. *boreale* and two *R. nivale* was supported by 100% bootstrapping (Fig. 2 C)"

The placement of a 5th individual labeled "*R. nivale* subsp. *boreale*" was never explicitly mentioned. I assume this is all four haplotypes combined, but this needs mentioned

Response: We sincerely thank you for careful reading. The "*R. nivale* subsp. *boreale*" represents the transcriptome data generated in this study. And we have clarified in the figure title of Figure 2. (Line 969-971)

Comment: Line 175: "Therefore, four haploid chromosomes were considered to originate from the same species"

This claim is not supported by the evidence presented.

The observation of all four haplotypes clustering with one sample of *R. nivale* and not the other can arise from a few scenarios besides a single species origin. There could exist population structure within *R. nivale* from which multiple species have derived from one sub population and not the others.

Now is as good of a time as any but I do not believe in the technical distinction between auto and allo polyploids and especially not whether the progenitors belong to the same species being the delineation. I think differences between auto and allo are quantitative and the authors have done an excellent job layering analyses to address this question.

As it pertains to this claim of this genome deriving from a single species, the phylogenetic tree in Figure 2C is insufficient to claim this as a fact.

I recommend removing this statement.

Response: Thank you for recognizing our analysis of polyploid types. We completely agree with you. The difference between autopolyploids and allopolyploids should be quantitative. Otherwise, the type of polyploid hybrids in closely related species cannot

be determined. Therefore, we have removed that statement.

Comment: Line 185: "1 basal angiosperm"

Though commonly referred to as a "basal" species, an extant species cannot be "basal". All extant species have been evolving for the same amount of time and contain their own derived characteristics. I recommend simply referring to this as "Amborella", or "1 species sister to all angiosperms" to keep the syntax consistent in that sentence.

Response: Thank you for your valuable feedback. We have revised to read "1 species sister to all other angiosperms (*Amborella*)," which accurately reflects the phylogenetic position of *Amborella*. (Line 188)

Comment: Line 191: "These species-specific genes were enriched in 12 KEGG pathways and 136 GO terms, such as arginine biosynthesis, nitrogen metabolism and flavonoid biosynthesis"

I cannot find where in the methods your GO enrichments are listed, but I did see not all genes were functionally annotated with a GO term. The background set to test these 369 *R. nivale* subsp. *boreale* specific genes should be all genes with a GO annotation rather than all genes in total. This is simply to test the robustness of your results which I do not expect to change, however reducing the background set is the more statistically appropriate test.

Response: Thank you for your meticulous review and valuable suggestion regarding the GO enrichment analysis. We used clusterProfiler for enrichment analysis. The background gene set used in the GO enrichment analysis was derived from the annotation result file (org.My.eg.db_1.0.tar.gz) produced in the annotation. This file contains all the genes that have been annotated by GO, and we have not made any changes. We present the analysis process as follows:

```
library(tidyverse)
library(clusterProfiler)
```

```
#KEGG
gene <- read.csv("~/enrichment /uni_gene.xls", sep="")
ee <-as.matrix(gene)
genev <-as.vector(ee)
gene2passway <- read.csv("~/enrichment / kegg_gene2passway.csv")
pathway2name <- read.delim("~/enrichment /kegg.pathway2name.tsv")
de_ekp <- enricher(genev, TERM2GENE = gene2passway, TERM2NAME = pathway2name, pvalueCutoff = 0.05, qvalueCutoff = 0.05)
de_ekp_df <- as.data.frame(de_ekp)

#GO
dir.create('R_Library', recursive = T)
install.packages('~/enrichment/org.My.eg.db_1.0.tar.gz', repos = NULL, lib = 'R_Library')
library(org.My.eg.db, lib = 'R_Library')
de_ego <- enrichGO(gene = genev, OrgDb = org.My.eg.db, keyType = 'GID', ont = 'ALL', qvalueCutoff = 0.05, pvalueCutoff = 0.05)
de_ego_df <- as.data.frame(de_ego)
```

Comment: Line 211: "In addition, the recent peak at *Ks* ~0.65 indicated that another polyploidy of *Rhododendron*, *Va. darrowii* and *Ac. chinensis* occurred ~78 Mya (Fig. 3C, S9)."

It was unclear to me both in the text and in the figure legend for Fig 3C whether the *Ks* distributions were of orthologs to *R. nivale* subsp. *boreale* or of paralogs within each genome. I can infer after reading some of the conclusions that these are intragenomic paralog *Ks* distributions but this needs made more clear in both the main text and in the figure legend.

Response: Thank you for your comment. Your inference is correct. We are very sorry for our unclear statement. We have clarified these in the text and in the figure title. The following revisions have been made to the manuscript: “In addition, the peak at *Ks* of

the paralogs approximately 0.65 Mya suggests another polyploidization event in *Rhododendron*, *Va. darrowii*, and *Ac. chinensis* estimated to have occurred at approximately 78 Mya." (Line 213-214; Line 980; Fig S9 title)

Comment: Line 215: "We identified nearly every grape chromosome with two highly compatible chromosome regions in *R. nivale* subsp. *boreale* (orthologous ratio 1:2) (Fig. 3D)"

For Fig 3D, the axis labels are ambiguous. Also I assume to dot plot is between genes and not chromosomes. I may have missed where this was stated but if its absent if must be included.

Response: Thank you for your comment. Dot plot analysis is based on homologous genes, and this statement is shown in the figure title of Fig 3. As follows: "D. Homologous gene dot plots between *R. nivale* subsp. *boreale* and *Vi. vinifera*. The red box exemplifies the orthologous ratio of 1:2 between *Vi. vinifera* and *R. nivale* subsp. *boreale*." (Line 983-985)

Comment: Line 218: "A total of 44 genes were positively selected and functionally annotated based on Hyphy and kaks_calculator (Table S18; S19)"

I appreciate the inclusion of these results in the supplement but I recommend inclusion of quantitative values that indicated these genes were positively selected. I do not have experience with Hyphy so I'm not sure this is possible, but for kaks_calculator I think you can provide the *Ka/Ks* value for quantitative comparisons of these positively selected genes.

Response: Thank you for your suggestion to include more information for positively selected genes. We recognize that the lack of experimental validation is our key shortcoming. In the latest version of the manuscript, we have improved our analytical approach to detect positive selection events that are as real as possible. This approach eliminates possible false positive signals through cross-validation using multiple models. We used Hyphy for positive selection genetic testing based on an exploratory model. Next, we used the Clade Model to exclude genes not affected by selection

pressure. Finally, we used MEME and Contrast-FEL to detect whether these potentially selected genes contain positive selection sites. Only when a gene meets all the model requirements do we consider it to be a valid positive selection gene. In addition, we have added the detailed results and methods of the selection analysis. We believe that the updated results are useful for better inferences of *R. nivale* subsp. *boreale* adaptation strategies to high altitude environments. (Line 219-234; Line 581-600; Tables S18-S20)

Comment: Line 233: "However, our results do not support this."

I think a statistical test is required to say whether the Ka/Ks distributions are not different within each homologous group for Fig S6. Otherwise the authors could rephrase this to "The distribution of Ka/Ks values do not appear different between our homologous groups (Fig S6)"

Response: Thank you for your suggestion. We have considered your recommendation to rephrase our statement, and have adjusted the text to "However, the distribution of Ka/Ks values did not differ between our homologous groups." In addition, we moved this statement to the discussion. (Line 369-374)

Comment: Line 263: "the transcript expression of homologous Group 3 was higher than that of the other groups (Fig. 5A)"

These values need normalized by the number of genes on each chromosome or by the total length of CDS as this pattern may simply reflect the number of genes.

Also it would be interesting to investigate this pattern across tissue types.

Response: Thank you for your comment. We agree that normalizing the transcript expression levels by the number of genes on each chromosome would provide a more accurate reflection of gene expression patterns. We have now included normalization based on the number of genes in our analysis and have updated Figure 5A accordingly. Additionally, we recognize the value in investigating expression patterns across different tissue types. While this was beyond the scope of our current study, we have noted it as a potential direction for future research.

Comment: Line 267: "Finally, 3,844 of the 6,388 (60.17%) single-match gene groups were identified as differential expression loci (DELs)"

(1) How were DEL determined?

(2) Were there biological replicates within each tissue?

(3) I also do not understand what conclusions are drawn from Fig 5B.

Response: We are very sorry that our unclear expression has caused your misunderstanding. (1) The identification strategy for DELs is shown in the method (Line 611-613). The method was published in the autotetraploid sugarcane genome study. Specifically, any two genes in a set of single-match genes with less than a two-fold difference in expression are defined as neutral; the other alleles are considered DELs. (2) Although our transcriptome comes from three replicates, samples are derived from a mixture of roots, stems, leaves, and buds. Expression levels in different tissues could not be analyzed. (3) As for Figure 5B, it illustrates the distribution of DELs across different homologous groups. As you mentioned in your comment, it is hard to tell the true haplotype origin of each chromosome. Thus, we used an independent analysis of each chromosome to compare differences in transcriptional expression level. In Figure 5B, we added labels for each heat map to indicate that our analysis was based on the chromosome level rather than haplotype. Each row of each heat map represents a set of single-match genes, while each column represents a chromosome. For example, in the heat map of Homologous group 1, 530 rows represent 530 sets of DEL alleles, and the colors represent differences in expression levels. The red and blue squares are scattered across different chromosomes within the homologous group, representing the distribution pattern of DELs.

Comment: Figure 6C:

What is the distribution of the "All" category when you removed genes that are not expressed. That should be a more appropriate comparison.

Response: Thank you for your advice. We have further clarified in figure 6 and the figure title. To address this, we have modified “All” to “Global Expression” to clearly show that the Figure 6C is drawn based on the expressed gene (Fig. 6C).

Comment: I was unable to access "PRJNA1040959" which is understandable as it should be private before publication but this led me to be unable to verify the availability of the data or if there were biological replicates for their expression analyses.

Response: Thank you for your comment. We provided a data access link from which the original data can be accessed (https://dataview.ncbi.nlm.nih.gov/object/PRJNA1040959?reviewer=46vvm122fdnc0s72kb0guu85fl). To obtain comprehensive transcriptional information, we collected and mixed samples from various tissues, ensuring three biological replicates.

Comment: Every phylogenetic tree in the main text and supplementary figures need explanations of how the trees were constructed including the program and methods (for example, maximum likelihood or parsimony?"

Response: Thank you for your comment. We have provided the construction methods of all the phylogenetic trees in the manuscript, including the figure title and the materials and methods section. These phylogenetic trees are constructed based on ML, and all are inferred using IQtree2 except for executing two large trees with thousands of sequences using Fasttree.

Comment: Some of conclusions of autopolyploid origin look at genome-wide patterns. I think some of them require a chromosome by chromosome approach like the expression analysis. Though the genome is phased, it is not phased across chromosomes, you do not know if homologous group 1 n1 is from the same parent as homologous group 5 n1.

Response: We could not agree with you more. We also expect to explore the origin of autopolyploids using a chromosome-by-chromosome approach. However, the lack of chromosome-level genomes in closely related species limits this approach. As the number of genetic resources increases, it will become more convenient and accurate to identify the origin of haplotypes in polyploids. For the level of gene expression, we have used a chromosome-by-chromosome process for analysis, such as allelic

expression difference analysis (Fig. 5B).

Comment: Group 3 *CBF* are not monophyletic Fig S12, does this change any of your findings / is it biologically interesting?

Response: We are very sorry for our carelessness. Group III and Group IV should be combined. We have made changes accordingly (Fig. S24). Group III seem to have been split into two clades in the Ericaceae. Our modifications are as follows: "Phylogenetic analysis indicated that *CBFs* of *Rhododendron* were divided into 3 groups (Fig. S24)." (Line 313-314)

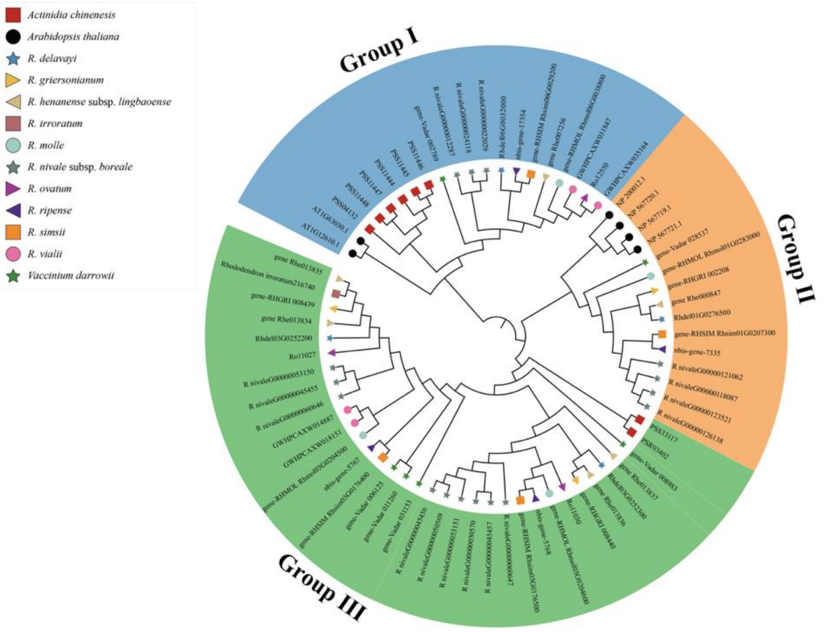

Fig. S24 Phylogenetic analysis of the CBF proteins in the 13 species. Group I to III were distinguished by four colors.

Comment: Line 457: "The presence of multiple alleles indicated that the expression level of *R. nivale* subsp. *boreale* is probably higher than that of the diploid ancestor"

I do not believe this result is supported by the data presented without being able to test the diploid ancestor. How do you know expression levels have not globally and proportionally reduced to compensate for the increase in copy number?

Response: Thank you for your comment. We acknowledge that our statement regarding the expression levels of *R. nivale* subsp. *boreale* compared to the diploid ancestor was

speculative. Without direct expression data from the diploid ancestor, we cannot conclusively determine if the observed multiple alleles correlate with higher expression levels. Hence, we have removed this statement.
