# Supplementary material for: The first high-altitude autotetraploid haplotype-resolved genome assembled (Rhododendron nivale subsp. boreale) provides new insights into mountaintop adaptation
Source: Gigascience. 2024 Aug 7;13:giae052. doi: 10.1093/gigascience/giae052 (PMC11304948; doi:10.1093/gigascience/giae052)
Supplement: giae052_GIGA-D-23-00395_Revision_2 [file giae052_giga-d-23-00395_revision_2.pdf]

GigaScience

The first high-altitude autotetraploid haplotype-resolved genome assembled (Rhododendron nivale subsp. boreale) provides new insights into mountaintop adaptation

--Manuscript Draft--

**Manuscript Number:**	GIGA-D-23-00395R2
**Full Title:**	The first high-altitude autotetraploid haplotype-resolved genome assembled (Rhododendron nivale subsp. boreale) provides new insights into mountaintop adaptation
**Article Type:**	Data Note
**Funding Information:**	Science and Technology Development Fund of Guidance from the Central Government to Locals (202207AB110016) — Prof. Shi-Kang Shen<br>Major Program for Basic Research Project of Yunnan Province (202101BC070002) — Prof. Shi-Kang Shen<br>National Natural Science Foundation of China (31870529) — Prof. Shi-Kang Shen<br>Graduate Scientific Research Fund Project of Yunnan University (KC-22221373) — Mr. Zhen-Yu Lyu<br>Scientific Research Fund of Yunnan Provincial Education Department (2024Y003) — Mr. Zhen-Yu Lyu
**Abstract:**	Background<br>Rhododendron nivale subsp. boreale Philipson et M. N. Philipson is an alpine woody species with ornamental qualities that serve as the predominant species in mountainous scrub habitats found at an altitude of ~4200 m. As a high-altitude woody polyploid, this species may serve as a model to understand how plants adapt to alpine environments. Despite its ecological significance, the lack of genomic resources has hindered a comprehensive understanding of its evolutionary and adaptive characteristics in high-altitude mountainous environments.<br>Findings<br>We sequenced and assembled the genome of R. nivale subsp. boreale, an assembly of the first subgenus Rhododendron and the first high-altitude woody flowering tetraploid, contributing an important genomic resource for alpine woody flora. The assembly included 52 pseudochromosomes (scaffold N50=42.93 Mb; BUSCO=98.8%; QV=45.51; S-AQI=98.69), which belonged to 4 haplotypes, harboring 127,810 predicted protein-coding genes. Conjoint k-mer analysis, collinearity assessment, and phylogenetic investigation corroborated autotetraploid identity. Comparative genomic analysis revealed that R. nivale subsp. boreale originated as a neopolyploid of R. nivale and underwent two rounds of ancient polyploidy events. Transcriptional expression analysis showed that differences in expression between alleles were common and randomly distributed in the genome. We identified extended gene families and signatures of positive selection that are involved not only in adaptation to the mountaintop ecosystem (response to stress and developmental regulation) but also in autotetraploid reproduction (meiotic stabilization). Additionally, the expression levels of the ERF VIIs were significantly higher than the mean global gene expression. We suspect that these changes have enabled the success of this species at high altitudes.<br>Conclusions<br>We assembled the first high-altitude autopolyploid genome and achieved chromosome-level assembly within the subgenus Rhododendron. In addition, a high-altitude adaptation strategy of R. nivale subsp. boreale was reasonbly speculated. This study provides valuable data for the exploration of alpine mountaintop adaptations and the correlation between extreme environments and species polyploidization.
**Corresponding Author:**	Shi-Kang Shen<br>Yunnan University

Powered by Editorial Manager® and ProduXion Manager® from Aries Systems Corporation

	Kunming, CHINA
**Corresponding Author Secondary Information:**	
**Corresponding Author's Institution:**	Yunnan University
**Corresponding Author's Secondary Institution:**	
**First Author:**	Zhen-Yu Lyu
**First Author Secondary Information:**	
**Order of Authors:**	Zhen-Yu Lyu
	Xiong-Li Zhou
	Si-Qi Wang
	Gao-Ming Yang
	Wen-Guang Sun
	Jie-Yu Zhang
	Rui Zhang
	Shi-Kang Shen
**Order of Authors Secondary Information:**	
**Response to Reviewers:**	Dear Editor and Reviewers,<br><br>Thank you for the feedback on our manuscript "The first high-altitude autotetraploid haplotype-resolved genome assembled (Rhododendron nivale subsp. boreale) provides new insights into mountaintop adaptation " (GIGA-D-23-00395R1).<br>In this version, we double-checked all the data and conclusions and improved our manuscript based on reviewer comments. Additionally, we have submitted the genome sequences to the National Genomics Data Center (NGDC) and included the accession number (GWHETKQ00000000.1) in the Data Availability section.<br>We have marked the changes in blue in the revised manuscript to facilitate your review. Thank you again for considering our work. We hope this manuscript meets the requirements for publication at Gigascience.<br><br>Best regards<br>Sincerely yours,<br>Shi-Kang Shen<br><br>Reviewer #1:<br>Thank you for reviewing our manuscript. We have made improvements to the manuscript in response to your comments. The point-by-point response follows:<br><br>> Comment: line 42 - Spell out the abbreviation for Group VII Ethylene Response Factors in the abstract.<br>>> Response: Done. "Additionally, the expression levels of the (Group VII ethylene response factor transcription factors) ERF VIIs were significantly higher than the mean global gene expression." (line 42)<br><br>> Comment: line 112- what mountain top, I know this is in the methods but needs clarifying here too.<br>>> Response: We have clarified this part. "R. nivale subsp. boreale samples were collected from the alpine region at an altitude of 4300 m, treated with liquid nitrogen, and sequenced (Fig. 1A). " (line 113)<br><br>> Comment: Line 119 - The total size of the assembly doesn't differ much between the different strategies - report the contig N50 in the main text which will tell you reader how well the assemblers performed. Looking at table S4 HiCanu might have performed better than Hifiasm. Even though I also prefer Hifiasm, why did you choose that over the longer and less contigs in hicanu? you say better integrity of genes and LTRs but

you don't present the results for this or point to supplement here.
>> Response: Thank you for your comment. Our LAI assessment of assemblies using different approaches was based on the whole genome, including four haplotypes. Although such an assessment may be inaccurate due to the influence of identical sequences between subgenomes, we believe it still reflects the continuity of the assemblies. We have included these results in the Supplementary Table and described the datasets used. The LAI evaluations for the assemblies using different approaches are as follows: Hifiasm (LAI=12.19), HiCanu (LAI=9.27), Canu (LAI=11.31). (Table S4)

> Comment: Line 148 - the ratio of single exonic needs the word "genes".
>> Response: Done. "The ratio of mono-exonic (single-exon) genes to multi-exonic (multiple-exon) genes was 0.245." (line 149-150)

> Comment: line 215- "In" the dot plot...
>> Response: Done. "In the dot plot comparing Vi. vinifera and R. nivale subsp. boreale (Fig. 3D, S7–8) …" (line 216)

> Comment: Line 227 -These two genes? Which genes? I think something is missing here.
>> Response: We have revised this sentence. Apologies for the oversight, there are four genes here, not two. The corrected text is as follows: "Four genes exhibited no significant positive selection sites. Finally, 16 genes met all model criteria and were identified as PSGs (Table. S19)". (line 228-230)

> Comment: line 287 - consistent with previous research - citation needed
>> Response: Done. "Consistent with previous research [33] …" (line 289)

> Comment: Line 447 - probably play important roles? I think "might" is more appropriate
>> Response: We have changed "probably" to "might". "Our positive selection analysis results suggested that M3K1 and CNGC1, which are associated with the MAPK cascade and Ca^{2+} signal transduction, might play important roles in low-temperature adaptation." (line 448-450)

> Comment: line 468 - change "likely serves" to "may serve"
>> Response: We have replaced "likely serves" with "may serve". "Notably, TOP3α, an important gene related to the BTR complex, exhibits positive selection in R. nivale subsp. boreale and may serve as a key factor in promoting accurate chromosomal segregation during meiosis in autopolyploids." (line 469-471)

> Comment: line 470 - grammar. and too strong tone.
>> Response: We have checked the grammar and softened the tone. The corrected text is as follows: "Our results revealed that R. nivale subsp. boreale distributed on the mountaintop is an autotetraploid, which might be mediated by the harsh environment at high altitudes." (line 472-473)

> Comment: line 476 - what do you mean here by dominant gene expression pattern? clarify.
>> Response: Thank you for your comment. We have clarified this sentence. The revised version is as follows: "Polyploidization likely plays an important role in mountaintop survival because increased gene dosage can lead to enhanced stress tolerance, greater genetic diversity, and the potential for novel traits that enhance adaptability [18]." (line 478-481)

Reviewer #3:
Thank you for reviewing our manuscript. We have made improvements to the manuscript in response to your comments. The point-by-point response follows:

> Comment: Abstract: "As a high-altitude woody polyploid, this species plays a distinct role in the adaptability of alpine plants"
- This plant does not affect the adaptability of other plants, it can tell us something about how plants adapt to alpine environments however. I'm sure thats what the authors intended. Suggested revision:
"As a high-altitude woody polyploid, this species may serve as a model to understand

Powered by Editorial Manager® and ProduXion Manager® from Aries Systems Corporation

	how plants adapt to alpine environments"<br>>> Response: Thank you for your suggestion. We have made the modification. (line 24-25)<br><br>> Comment: Line 118: "k-mers" should just be "k-mer"<br>>> Response: Done. "This species was identified as a tetraploid based on k-mer analysis (Fig. 1E)." (line 119)
**Additional Information:**	
**Question**	**Response**
Are you submitting this manuscript to a special series or article collection?	No
**Experimental design and statistics**<br><br>Full details of the experimental design and statistical methods used should be given in the Methods section, as detailed in our Minimum Standards Reporting Checklist. Information essential to interpreting the data presented should be made available in the figure legends.<br><br>Have you included all the information requested in your manuscript?	Yes
**Resources**<br><br>A description of all resources used, including antibodies, cell lines, animals and software tools, with enough information to allow them to be uniquely identified, should be included in the Methods section. Authors are strongly encouraged to cite Research Resource Identifiers (RRIDs) for antibodies, model organisms and tools, where possible.<br><br>Have you included the information requested as detailed in our Minimum Standards Reporting Checklist?	Yes
**Availability of data and materials**<br><br>All datasets and code on which the conclusions of the paper rely must be either included in your submission or deposited in publicly available repositories	Yes

Powered by Editorial Manager® and ProduXion Manager® from Aries Systems Corporation

(where available and ethically appropriate), referencing such data using a unique identifier in the references and in the “Availability of Data and Materials” section of your manuscript.<br><br>Have you have met the above requirement as detailed in our Minimum Standards Reporting Checklist?	

Powered by Editorial Manager® and ProduXion Manager® from Aries Systems Corporation

Manuscript
Click here to access/download;Manuscript;Rhodo_for_production.docx

Title: The first high-altitude autotetraploid haplotype-resolved genome assembled (*Rhododendron nivale* subsp. *boreale*) provides new insights into mountaintop adaptation

Authors: Zhen-Yu Lyu[1], Xiong-Li Zhou[1], Si-Qi Wang[1], Gao-Ming Yang[1], Wen-Guang Sun[2], Jie-Yu Zhang[2], Rui Zhang[1], Shi-Kang Shen[1*]

Affiliation:

[1]Ministry of Education Key Laboratory for Transboundary Ecosecurity of Southwest China, Yunnan Key Laboratory of Plant Reproductive Adaptation and Evolutionary Ecology, Institute of Biodiversity, School of Ecology and Environmental Science, Yunnan University, Kunming, 650504, Yunnan, China

[2]School of Life Sciences, Yunnan Normal University, Kunming, 650500, Yunnan, China

ORCID iDs: Zhen-Yu Lyu [0009-0003-8309-9199]; Shi-Kang Shen [0000-0002-0611-6763];

Corresponding author: *Shi-Kang Shen, E-mail: ssk168@ynu.edu.cn; (ORCID: 0000-0002-0611-6763)

Telephone: +86-871-65933510; Fax: +86-871-65933510;

Postal address for corresponding authors: School of Ecology and Environmental Sciences, Yunnan University, No.2 Green Lake North road Kunming, Yunnan, 650091, China

Words: 7383 (excluding references)

Figures: 7 color figures.

Abstract

Background

Rhododendron nivale subsp. *boreale* Philipson et M. N. Philipson is an alpine woody species with ornamental qualities that serve as the predominant species in mountainous scrub habitats found at an altitude of ~4200 m. As a high-altitude woody polyploid, this species may serve as a model to understand how plants adapt to alpine environments. Despite its ecological significance, the lack of genomic resources has hindered a comprehensive understanding of its evolutionary and adaptive characteristics in high-altitude mountainous environments.

Findings

We sequenced and assembled the genome of *R. nivale* subsp. *boreale*, an assembly of the first subgenus *Rhododendron* and the first high-altitude woody flowering tetraploid, contributing an important genomic resource for alpine woody flora. The assembly included 52 pseudochromosomes (scaffold N50=42.93 Mb; BUSCO=98.8%; QV=45.51; S-AQI=98.69), which belonged to 4 haplotypes, harboring 127,810 predicted protein-coding genes. Conjoint *k-mer* analysis, collinearity assessment, and phylogenetic investigation corroborated autotetraploid identity. Comparative genomic analysis revealed that *R. nivale* subsp. *boreale* originated as a neopolyploid of *R. nivale* and underwent two rounds of ancient polyploidy events. Transcriptional expression analysis showed that differences in expression between alleles were common and randomly distributed in the genome. We identified extended gene families and signatures of positive selection that are involved not only in adaptation to the mountaintop ecosystem (response to stress and developmental regulation) but also in autotetraploid reproduction (meiotic stabilization). Additionally, the expression levels of the (Group VII ethylene response factor transcription factors) *ERF VIIs* were significantly higher

than the mean global gene expression. We suspect that these changes have enabled the success of this species at high altitudes.

Conclusions

We assembled the first high-altitude autopolyploid genome and achieved chromosome-level assembly within the subgenus *Rhododendron*. In addition, a high-altitude adaptation strategy of *R. nivale* subsp. *boreale* was reasonbly speculated. This study provides valuable data for the exploration of alpine mountaintop adaptations and the correlation between extreme environments and species polyploidization.

Key words: Autotetraploid, Evolutionary history, Harsh environment, Mountaintop adaptation, *Rhododendron*,

1. Context

Rhododendron L. is the largest genus in Ericaceae and the largest woody plant genus in the Northern Hemisphere, with more than 1,000 species. It is also representative of the highly diverse Sino-Himalayan Flora in East Asia, shaped by the topographic and climatic heterogeneity resulting from the uplift of the Qinghai-Tibet Plateau [1,2]. Furthermore, *Rhododendron* is one of the few woody flowering species that is dominant in plant communities found within the delicate subalpine to alpine transition zone and presents a perfect opportunity to explore the mechanisms behind the evolution and adaptation of alpine woody plants [3,4]. In *Rhododendron*, *R. nivale* subsp. *boreale* Philipson et M. N. Philipson is one of the few woody flowering plants discovered to be distributed at altitudes above 5000 m and is one of the few polyploid (2n=4x=52) woody plants in the Qinghai-Tibet Plateau [1,5,6]. *R. nivale* subsp. *boreale*, a member of the subg. *Rhododendron*, is a small-leaved, highly branched shrub distributed at high altitudes of mountaintops (up to alt. 5400 m) down to the mountainsides (~3200 m). This species demonstrates remarkable adaptability, as evidenced by its diverse habitats, including alpine meadows, forest edges, and metal mining areas [7]. Currently, *R. nivale* subsp. *boreale* is an important ornamental plant resource in mountainous plateau areas and is used in traditional Tibetan medicine [8,9]. Therefore, exploring the evolutionary patterns and adaptation mechanisms of *R. nivale* subsp. *boreale* not only promotes the understanding of alpine adaptation evolution in woody plants, but also establishes a basis for the commercial exploitation of high-altitude ornamental plants.

Genetic perspectives provide a better understanding of evolution and adaptive differentiation [10]. However, the paucity of genomic data is a significant impediment to

research advancement [11]. For example, in a recent high-altitude adaptation study, only seven alpine plant genomes were used, indicating that the genetic resources of alpine plants are far from sufficient compared to the diversity of the high-altitude flora [12]. Moreover, polyploidy likely enhanced the adaptability of alpine plants to harsh environments [13]. Unfortunately, acquiring polyploid genetic data remains challenging, particularly for autopolyploids with highly similar subgenomes. Currently, the assembly of autopolyploid genomes presents significant challenges, resulting in the publication of only a select number of such genomes, including those of *Medicago sativa*, *Saccharum spontaneum*, *Solanum tuberosum*, and *Rheum officinale* [14,15,16,17]. To further understand the evolution and adaptation of the alpine flora, additional genetic resources, particularly of polyploids, are essential.

Polyploidy has been theorized to be both a potential evolutionary roadblock and a catalyst for evolutionary breakthroughs and the proliferation of species [18]. On the one hand, following polyploidy events, rapid shifts in gene expression and epigenetic modifications can bestow the polyploid with an almost instant competitive edge, which is usually reflected in their broader geographical ranges compared with their diploid ancestors [19,20]. Therefore, polyploidy tends to be ecologically advantageous and occurs in variable climatic regions, such as the Qinghai-Tibet Plateau alpine and Pan-Arctic regions [21,22]. Comprehending these adaptive mechanisms in high-altitude polyploids not only clarifies evolutionary dynamics but also provides insights into conservation strategies. On the other hand, auto and allopolyploids face a significant obstacle: the accurate segregation of chromosomes during meiosis [23,24]. In recent years, our understanding of the molecular basis for polyploid adaptations to meiotic challenges has significantly increased; however, compared to allotetraploids, little is known

about the molecular mechanisms underlying the stabilization of autotetraploid meiosis [25]. Advances in molecular technology and subsequently, more genetic resources will provide new insights into the survival, evolution, adaptation, and conservation of polyploids.

Here, we present a haplotype-resolved tetraploid genome of mountaintop plant *R. nivale* subsp. *boreale* from an altitude of 4,287.5 m, which is the first chromosome-level genome assembly of the subgenus *Rhododendron*. Based on this assembly, we identified polyploid types, deciphered whole genome duplication (WGD) events, and investigated which genes or gene families are potential candidates involved in alpine mountaintop adaptation and the survival of polyploids. This genome not only establishes the groundwork for comprehending the evolution and adaptation of *Rhododendron* species, but also offers valuable genetic resources to investigate the origin, recombination, and differentiation of polyploid species.

2. Results

2.1 Genome estimation, sequencing, and assembly

R. nivale subsp. *Boreale* samples were collected from the alpine region at an altitude of 4300 m, treated with liquid nitrogen, and sequenced (Fig. 1A). We obtained a total of 33.35 gigabases (Gb) of PacBio CCS long reads with an average length of 15.86 kb and an N50 length of 16.15 kb (Table S1). A genome survey was performed based on DNBseq short reads (95.61 Gb; Table S2), and the result revealed an estimated genome size of 2.48 Gb, which was consistent with that estimated by flow cytometry (Fig. S1, S2; Table S3). This species was identified as a tetraploid based on *k-mer* analysis (Fig. 1E). The three initial assembly sizes assembled using Hifiasm, Canu v1.9, and HiCanu were 2.48 Gb, 2.39 Gb, and 2.40 Gb,

respectively (Table S4; S8). The assembled version of Hifiasm was used for subsequent analysis, as it ensures higher integrity of both genes and long terminal repeats (LTRs). The long reads and NGS reads were mapped to the unitig-level assembly to assess the assembly quality. Long-read and whole-genome sequencing (WGS) reads were mapped to 99.87% and 99.59%, respectively, and RNA-seq reads exhibited mapping rates exceeding 94% (Table S5). The average GC content was 41.10%. We used the AllHiC algorithm to improve the genome assembly to the chromosome level using 138.98 Gb of Hi-C data. After manual checking, a total of 2.17 Gb of unitigs were anchored to 52 pseudochromosomes (scaffold N50 = 42.93 Mb), ranging from 23.70 to 59.70 megabase (Mb) in length, and containing four haplotypes (13 pseudochromosomes per haplotype) (Fig. 1C; Table S6). The Hi-C heatmap clearly showed the interactions of 13 homologous groups (Fig. 1D), with high similarity observed between the pseudochromosomes within each homologous group.

This genome was assembled with a high consensus quality value (QV = 45.51; error rate = 0.0028%) and high *k-mer* completeness (97.79%) (Table S7). BUSCO assessment indicated that the completeness of the conserved embryophyte genes was 98.8 % (Table S8). The quality of the genome structure at the reference genome level (assembly quality indicators of large structural fragments; S-AQI = 98.69) was assessed using CRAQ (Table S9).

2.2 Annotation

A repeat sequence of 1,549,068,457 bp was identified, accounting for 62.63% of the genome assembly (Table S10). The richest category of repeats was LTRs (43.12%), with *Gypsy* and *Copia* accounting for 33.66% and 5.88% of the repeats, respectively (Fig. S3). In addition, the LTR assembly index (LAI) was greater than 14 (n1:14.78, n2:14.84, n3:14.35, and n4:14.38)

based on LTR annotation, which indicated that the assembly met the reference category standards. By combining ab initio, homology, and transcriptome data predictions, 127,810 protein-coding genes were predicted, with an average gene length of 4,736.07 bp. The total length of the coding sequences (CDS) was 148,274,600 bp, and the average number of CDSs per gene was 4.8 (Table S11). The completeness of 98.6% of the annotated protein-coding genes of *R. nivale* subsp. *boreale* was assessed using BUSCO. Of the protein-coding genes, 96.86% were annotated functionally (Table S12). The ratio of mono-exonic (single-exon) genes to multi-exonic (multiple-exon) genes was 0.245. We annotated 17,049 candidate noncoding RNAs, including 703 microRNAs (miRNAs), 3,672 transfer RNAs (tRNAs), 5,373 small nuclear RNAs (snRNAs), and 7,301 ribosomal RNAs (rRNAs) (Table S13).

2.3 Confirmation of Autotetraploid

Polyploids are commonly found in plants. However, the origins of the polyploids differ and include both homologous and heterologous origins. Extensively studied allotetraploids such as peanuts, cotton, and wheat [26,27,28], exhibit significant subgenomic differences, allowing for their division into distinct subgenomes. In contrast to allotetraploids, the high similarity among haplotypes greatly increases the difficulty in assembling autotetraploids. To determine the polyploid type of *R. nivale* subsp. *boreale*, we employed *k-mer* analysis, collinearity analysis, and phylogenetic analysis for cross-validation. The 21 *k-mer* frequency analysis revealed four distinct peaks (located at 33, 68, 106, and 136) (Fig. S2), which was highly similar to the results for autotetraploids (*Medicago sativa* and *Saccharum spontaneum*). Nucleotide heterozygosity is an important criterion for determining polyploid types [29]. Nucleotide heterozygosity form analysis of *R. nivale* subsp. *boreale* showed 2.53% AAAB and 1.28%

AABB (Table S3), which was consistent with the expectation that the heterozygous rate of autotetraploid AAAB would be greater than that of AABB.

Nevertheless, these methods were insufficient to identify the polyploid type. For example, although genomic analysis indicated higher AAAB than AABB, *Artemisia argyi* was identified as an allotetraploid [30]. To further determine the polyploid type of *R. nivale* subsp. *boreale*, synteny analysis was performed based on syntenic blocks. As expected, the dot plot and syntenic blocks indicated synteny among the four haplotypes (Fig. 2A), with 20,172 gene pairs showing synteny between haplotypes 1 and 2, 20,249 between haplotypes 2 and 3, and 19,883 between haplotypes 3 and 4 (Fig. 2B, S4). Additionally, we downloaded transcriptome data from 11 samples of seven closely related species (Table S14) to infer the phylogenetic positions of the four haplotypes of *R. nivale* subsp. *boreale* and identified a monophyletic group consisting of six species (*R. nitidulum*, *R. hippophaeoides*, *R. thymifolium*, *R. nivale*, *R. nivale* subsp. *Boreale,* and *R. lapponicum*) of subsect. *Lapponica* with high support. A clade containing all *R. nivale* subsp. *boreale* (including the transcriptome and four haplotypes) and two *R. nivale* was supported by 100% bootstrapping (Fig. 2 C).

Moreover, 25,791 orthogroups were identified in the four haplotypes, with 16,403 shared by all, 5,242 shared by three, 3,631 shared by two, and only 515 (n1:134; n2:139; n3:135; n4:107) unique to each haplotype genome, showing high genetic similarity among haplotypes (Fig. 2D). Overall, the combined results of *k-mer* analysis, collinearity analysis, and phylogenetic analysis indicated that *R. nivale* subsp. *boreale* is an autotetraploid species.

2.4 Comparative analysis and recent polyploidization

The phylogenetic position and divergence times of *R. nivale* subsp. *boreale* were inferred

from 18 other species, including 12 species of Ericales (10 *Rhododendron*), two species of Cornales, one species of Gentianales, one species of Vitales, two species of monocotyledons, and one sister species to all angiosperms (*Amborella*). Altogether, 666,442 genes were used to infer orthology. A total of 625,661 genes (93.9%) clustered into 37,844 orthologous gene families, of which 6,547 were shared across all species (Fig. 2B; Table S15). In total, 209 single-copy gene families were identified. In total, 146 gene families, comprising 369 genes, were found to be specific to *R. nivale* subsp. *boreale* (Fig. S5). These species-specific genes were enriched in 12 Kyoto Encyclopedia of Genes and Genomes (KEGG) pathways and 136 gene ontology (GO) terms including arginine biosynthesis, nitrogen metabolism, and flavonoid biosynthesis (Tables S16 and S17).

In total, 209 single-copy orthologous genes were used to reconstruct phylogenetic relationships using IQ-TREE. All nodes were supported by high bootstrap values (> 95%). The results supported that *Rhododendron* is a monophyletic group, and the 10 species of *Rhododendron* were divided into four clades representing four subgenera (*Tsutsusi*, *Rhododendron*, *Pentanthera,* and *Hymenanthes*) (Fig. 3A). The time tree inferred from the MCMCtree suggested that the ancestor of *Rhododendron* separated from the common ancestor of *Rhododendron* and *Va. darrowii* approximately 41.2 Mya. The split between *R. nivale* subsp. *boreale* and the sister groups *(R. mole, R. henenense, R. delavayi, R. griersonianum, and R. irroratum*) was 30.4 Mya, while the divergence time of *R. molle* was 28.3 Mya (Fig. 3A).

The synonymous substitution rate (*Ks*) of orthologs and paralogs of seven species (four *Rhododendron* species, one *Vaccinium*, one *Actinidia* species, and one *Vitis*) was calculated to determine the WGD events that occurred in *R. nivale* subsp. *boreale*. Polyploidy analysis

indicated that *Rhododendron* and *Va. darrowii* experienced two rounds of ancient polyploidy events, whereas *Ac. chinensis* experienced three. Similar peaks were observed for all four *Rhododendron* species and *Va. darrowii*. And the farthest peak revealed an ancient γ whole-genome triplication (WGT-γ) event common to *Rhododendron* and other core eudicots, which was inferred to have occurred 122–164 Mya from a previous study [31]. In addition, the peak at *Ks* of the paralogs approximately 0.65 Mya suggests another polyploidization event in *Rhododendron*, *Va. darrowii,* and *Ac. chinensis* estimated to have occurred at approximately 78 Mya (Fig. 3C, S9). In the dot plot comparing *Vi. vinifera* and *R. nivale* subsp. *boreale* (Fig. 3D, S7–8), nearly every grape chromosome exhibited two highly compatible chromosomal regions in *R. nivale* subsp. *boreale* (orthologous ratio 1:2) (Fig. 3D).

2.5 Analysis of positive selection

Using multiple models, we expected to provide more accurate detection of the selection signals of *R. nivale* subsp. *boreale*. First, adaptive Branch-Site Random Effects Likelihood (aBSREL) was used to test for positive selection in the high-altitude branch of each gene by examining positively selected genes (PSGs). Subsequently, we employed clade model C (CmC) to further evaluate foreground and background selection differences compared with the null model M2a_rel to exclude genes not affected by selection pressure. Twenty potential PSGs were identified by both models (Table. S18). Positive selection sites were detected using the Mixed Effects Model of Evolution (MEME) and the Contrast-FEL, with the number of positive selection sites per gene ranging from 0 to 19 (Table. S19). Four genes exhibited no significant positive selection sites. Finally, 16 genes met all model criteria and were identified as PSGs (Table. S19). In addition, 19 genes were identified as PSGs using KaKs_Calculator

and were functionally annotated. (Table. S20). These PSGs are associated with various biological processes, including meiosis recombination (*TOP3α*), nucleotide excision repair (*UVR8*, *RAD23B*), leaf surface wax metabolism (*LTG30*, *LTP1*), auxin transporter (*ABCB19*), signal transduction and regulation (*M3K1*, *CAGC1*), and biological clock regulation (*ESD4*). Additionally, we explored the selection pressure within 13 homologous groups where similar pressures were observed. (Fig. S6).

2.6 Gene duplication and family evolution

Based on the ultrametric tree, the gene family evolution of 19 species was compared with that of the most recent common ancestor (MRCA). Overall, 3,356 orthogroups expanded in *R. nivale* subsp. *boreale*, while only 756 orthogroups contracted. Among these, 375 and 79 orthogroups expanded and contracted significantly, respectively. GO and KEGG enrichment analyses suggested that the significantly expanded orthogroups were primarily enriched in pathways such as brassinosteroid (BR) biosynthesis, terpenoid biosynthesis, and isoflavonoid biosynthesis (Fig. 4A).

To explore the connection between gene duplication and gene family expansion, 20,673 duplicated genes were identified and classified into five categories: 3,899 whole-genome duplication (WGD; 18.86%), 2,711 transposed duplication (TRD; 13.11%), 3,744 tandem duplication (TD; 18.11%), 4,738 proximal duplications (PD; 22.92%), and 5,581 dispersed duplication (DSD; 27.00%) duplications (Fig. 4B). Among these, PD and TD contributed the most to the expansion of gene families. Moreover, ω (*Ka/Ks*) ratios of all duplication categories were calculated, revealing that PD and TD demonstrated superior ω scores compared to other types, while the lowest ω score was for WGD (Fig. 4C). KEGG functional

enrichment analysis indicated that the functions of genes shared by the significantly expanded orthogroups and five different duplication types were differentiated. WGD genes were enriched in plant hormone signal transduction and nucleotide excision repair, TRD duplications were implicated in plant-pathogen interactions and *O*-glycan biosynthesis, gene family expansions related to arachidonic acid metabolism and linoleic acid metabolism were mainly contributed by DSD, and duplications of TD and PD were associated with BR biosynthesis, cytochrome P450, and isoflavonoid biosynthesis (Fig. 4D; Table S21–S30).

2.7 Expression of alleles

Transcriptome data from the roots, stems, leaves, and buds of *R. nivale* subsp. *boreale* were used to explore allelic expression patterns. Overall, 77,892 genes, representing 60.94% of all the genes, were expressed in at least one tissue. In single matching four alleles (1:1:1:1), 12,642 out of the 14,550 alleles were expressed in at least one allele. The expression levels of chromosomes within each homologous group were similar, with homologous group 3 showing a higher transcript expression than the other groups (Fig. 5A). Homologous Group 10 had the lowest expression level. We selected genes with a transcripts per kilobase per million mapped reads (TPM) value ≥ 1 to compare the differences in expression levels between haplotypes, and 6,388 single-match gene groups were identified. Finally, 3,844 of the 6,388 (60.17%) single-match gene groups were identified as differentially expressed loci (DELs), with DEL ratios ranging from 56.14% to 63.93% per pseudochromosome. The DELs were randomly distributed across the genome (Fig. 5B).

2.8 Evolution of the APETALA2/ethylene responsive factor (AP2/ERF)

Plant APETALA2/ethylene responsive factors (AP2/ERFs) and cytochrome P450s (CYPs),

which likely play important roles in the adaptation of plants to high altitudes, participate in a multitude of biochemical pathways and fulfill various functions in the realms of growth and protection, including responses to UV irradiation, dehydration, and pathogens [32,33]. Therefore, AP2/ERF and CYP families were explored. We identified the AP2/ERF family in 10 *Rhododendron* and two related species (*Actinidia chinensis* and *Vaccinium darrowii*) using the HMMer method. In total, 2,397 genes were identified as belonging to the AP2/ERF family in 12 species after artificially confirming the presence of the AP2 domain (Table S31). For convenience, haplotypes were extracted from the haplotype-resolved assemblies of *R. nivale* subsp. *boreale* and *R. vialii* to compare the gene counts within the family. Among *Rhododendron* species, *R. ovatum* exhibited the highest gene count (163), while *R. irroratum* contained only 88 genes. The majority of species in our study had gene counts of approximately 140. These genes were randomly distributed across 13 pseudochromosomes (Fig. S10-21). To further understand the phylogenetic mechanisms of the AP2/ERF family in *Rhododendron*, proteins of *Ar. thaliana* and other 12 species were used to construct the phylogenetic tree. Consistent with previous research [33], 13 categories, including the AP2, ERF (B-1 to B-6), DREB (A-1 to A-6), RAV, and soloist subfamilies, were identified (Fig. 6A). Compared with *Ar. thaliana*, the categorization of B-3 was expanded in *Rhododendron*.

The alpine environment is variable, with significant temperature differences between day and night, strong ultraviolet radiation, and low oxygen partial pressure. Group VII ethylene response factor transcription factors (*ERF VII*s) are associated with altitude adaptation [34]. Hence, based on the homology of *ERF VIIs* in *Ar. thaliana*, we identified *ERF VIIs* in *Rhododendron*. Ten species of *Rhododendron* contained one (*R. molle*) to five (*R. simsii*) *ERF*

VIIs (Table S32). The phylogenetic tree topology showed that *ERF VIIs* were divided into four groups. *ERF VIIs* of *Ar. thaliana* genes were found in Groups I, III, and IV (Fig. S22). Group II consisted solely of Ericales genes, and one clade contained the alpine species of *Rhododendron*. Based on motif analysis, similar gene structures within the groups showed phylogenetic reliability (Fig. S23). Interestingly, we estimated the turnover of *ERF VII* subfamilies using the maximum likelihood method and found a continuous decrease in the number of genes (Fig. 6B). However, among the 13 *ERF VII* genes, 12 were expressed in the roots, stems, leaves, and buds of *R. nivale* subsp. *boreale* and exhibited significantly higher expression levels than the global gene expression (Fig. 6C).

C-repeat binding factors/dehydration-responsive element binding protein 1 (*CBFs/DREB1s*) play a crucial role as transcription factors that regulate gene expression during cold acclimation. Consistent with previous studies, *CBFs* were categorized as A-1 of the dehydration-responsive element binding (DREB) subfamily, which is separated from A-4 of DREB [35]. We used the BLASTP program and two conserved sequences, PKRxAGRxKFxETRHPV and DSAWR, surrounding the AP2/ERF domain to accurately identify *CBF* genes. Sequence comparison with *Ar. thaliana* revealed that *CBFs* have a highly conserved domain. A total of 49 *CBFs* were identified from 10 *Rhododendron* species, of which *R. nivale* subsp. *boreale* contained 16 *CBFs* (12 *CBF* genes with four alleles and 2 *CBF* genes with two alleles) (Table S32). Phylogenetic analysis indicated that *CBFs* of *Rhododendrons* could be divided into three groups (Fig. S24). Similar conserved motifs were observed in each group, indicating the reliability of the relationship of *CBFs* (Fig. S25). In addition, family turnover based on BadiRate was re-validated, and *CBFs* were continuously

lost without gain, resulting in the contraction of *CBF* genes (Fig. 6B). Approximately only half (9/16) of the *CBFs* were expressed (Fig. 6C).

2.9 Evolution of the CYP family

Using the *R. nivale* subsp. *boreale* genome we assembled, along with the genomes of 12 other closely related species, we investigated the evolutionary pattern of the CYP family. The number of CYP family members in each species ranged from 221 to 447, as determined using local BLASTP, hmmsearch, and manual checks (Table S33). Among them, *R. irroratum,* which contains 447 CYPs, had the highest gene count. The identified CYP proteins varied in length, ranging from 303 to 621 amino acids. In addition, CYPs were unevenly located on different pseudochromosomes (Fig. S10-21).

To determine the phylogenetic relationship between CYPs, we constructed an unrooted maximum likelihood (ML) phylogenetic tree using protein alignments that primarily contained conserved domains and compared them with CYP superfamily members from *Ar. thaliana*. Following the classification system proposed [36], all CYPs were categorized into two distinct types: A-type, which comprises the CYP71 clan, and non-A-type, which comprises the CYP51, CYP72, CYP74, CYP85, CYP86, CYP97, CYP710, CYP711, and CYP727 clans. Our analysis confirmed monophyly within each clan, with the CYP71 clan being the most gene-rich, representing over half of all identified CYPs. In contrast, the CYP711 and CYP727 clans were the smallest (Fig. 7A–C). In comparison to *Ar. thaliana* and *Ac. chinensis*, we noted a species-specific increase in the CYP family across different *Rhododendron* species, particularly within the CYP71, CYP85, and CYP72 clans. These expansions are likely to play pivotal roles in species-specific adaptations. The CYP71 clan was mainly involved in the

biosynthesis of alkaloids, sesquiterpenoids, cyclic terpenoids, and flavonoids, whereas the CYP85 clan is implicated in the modification of cyclic terpenes and sterols in the BR, abscisic acid (ABA) and gibberellin (GA) pathways. CYP72 is involved in isoprenoid hormone catabolism. Unexpectedly, the CYP family of *R. henanense* subsp. *lingbaoense* was contracted, particularly the CYP72 clan (Fig. 7B). The patterns of duplicated gene pair identification showed that PD and TD remarkably contributed to the CYP family variation. In addition, WGD events accounted for a large proportion of duplications in *R. nivale* subsp. *boreale* and *R. ovatum* (Fig. 7D).

3. Discussion

Climate change is expected to significantly impact mountaintop ecosystems. Understanding the evolutionary patterns and survival strategies of mountaintop species is imperative to protect them [37]. Polyploidy is beneficial for the survival of species in harsh environments [21]. Therefore, it is essential to understand the evolution and adaptation of extremely high-altitude environmental species from the perspective of polyploidy. In this study, we provide a chromosome-scale and haplotype-resolved autotetraploid genome of *R. nivale* subsp. *boreale* using the DNBseq, PacBio CCS, and Hi-C sequencing platforms. The *R. nivale* subsp. *boreale* genome contains 52 pseudochromosomes divided into 13 homologous groups. Our assembly was estimated to be of high quality using four methods (Merqury, BUSCO, CRAQ, and LAI). In addition, we used various methods to determine autotetraploid identity. As the first autotetraploid genome of an alpine woody plant, the genome of *R. nivale* subsp. *boreale* has laid an important foundation for understanding the adaptation and evolution of woody plants in harsh environments at high altitudes. Consistent with previous systematics of

Rhododendron studies, Subg. *Rhododendron,* to which *R. nivale* subsp. *boreale* belongs, is a sister of Subg. *Pentanthera* and Subg. *Hymennanthes* [4]. Furthermore, our data suggest that the ancient WGD event in *R. nivale* subsp. *boreale* occurred approximately 78 Mya, which is probably shared with Ericaceae [38]. Overall, *R. nivale* subsp. *boreale* has recently experienced an additional WGD event, in addition to the WGT-γ event shared by the core eudicots and the WGD event shared by the Ericales. Recent polyploidy events, like those observed in other polyploids, are likely important factors for highly conserved pseudochromosomes without rearrangements [31]. The selection analysis revealed similar selection pressures in homologous groups (Fig. S6). The different subgenomes of allopolyploids were under significantly different selection pressures, and it has been speculated that different haplotypes of autopolyploids probably also faced different selection pressures [39]. However, the distribution of *Ka*/*Ks* values did not differ between our homologous groups (Fig. S6). Based on gene family evolution and positive selection analysis, we postulate the potential high-altitude adaptation strategies for *R. nivale* subsp. *boreale*.

Mountaintop ecosystems are exposed to high levels of UV radiation, low partial pressures of oxygen, and volatile temperatures and humidity [38]. Understanding the mechanisms of the adaptation of plants to high altitudes has long interested botanists. Altitude is positively correlated with UV radiation, with UV radiation rates increasing by 5.1–15% every 1,000 m increase in altitude [40]. Alpine plants employ various mechanisms to mitigate the effects of UV radiation, including cell wall surface modifications and the creation of a leaf cuticle consisting of cutin and cuticular waxes [41]. This cuticle serves as a protective shield against water loss and excessive UV radiation by forming a physical barrier between the plant surface

and the environment [12]. Second, to enhance their tolerance to UV radiation and protect themselves from UV damage, plants accumulate flavonoids that absorb UV radiation from strong light [42]. Several genes involved in cuticle and UV tolerance, such as *CER1*, *FARs,* and *MYB27*, are positively selected in high-altitude plants [12]. We identified a similar situation in *R. nivale* subsp. *boreale* living in a high-altitude mountaintop environment. *LTP1* and *LTPG30* have been identified as PSG, of which *LTP1* is associated with the biosynthesis and secretion of cuticular wax [43]. In wild-type bilberry (*Vaccinium myrtillus*), which is closely related to *Rhododendron*, expression of the *LTP* gene specific to the skin suggests its involvement in transporting wax compounds into the cuticle [44]. *LTPG30* performs similar functions [45]. Therefore, surface modification of the cell wall is likely to be the initial line of defense against UV damage in *R. nivale* subsp. *boreale*.

Flavonoids are widely recognized as important chemical compounds that protect plants from UV radiation [46]. We observed a significant expansion of the flavonoid gene family, which suggests that the absorption of UV radiation by flavonoid synthesis may be one of the key ways to reduce UV damage in *R. nivale* subsp. *boreale*. This finding was consistent with the conclusions of previous studies [12]. However, the creation of a wax barrier and biosynthesis of flavonoids that absorb UV radiation are insufficient to fully shield plant cells from the intense UV radiation found in mountainous environments. UV radiation not blocked by such barriers reaches deep into cells, damaging biological macromolecules such as DNA, thereby affecting the growth and development of various cells [47]. In this study, *UVR8* and *RAD23B*, which contribute to DNA repair, were positively selected. Specifically, *UVR8* enhances UV-B perception by interacting with the photomorphogenic repressor *COP1*, while

RAD23B primarily collaborates with *RAD4* to facilitate nucleotide excision repair [48,49]. These interactions are likely to augment the UV tolerance in *R. nivale* subsp. *boreale.*

In alpine environments, plants have evolved myriad morphological and physiological adaptations to contend with the rigor of high-altitude conditions [50]. *R. nivale* subsp. *boreale* native to mountaintops, typically reaches heights of less than 30 cm, with leaves that seldom exceed 5 mm in both length and width, exhibits delayed flowering, and produces seeds that are nearly indiscernible. These characteristics are thought to be a response of *Rhododendron* to low temperatures at high altitudes, poor nutrition, and extremely short growth cycles [51]. Selection analysis revealed PSGs related to auxins, morphogenesis, and the biological clock. For instance, some PSGs identified are associated with auxin transport (*ABCB19*), seed size (*DA1*), and the biological clock (*ESD4*) [52,53,54]. Concurrently, the gene family for organ development, tissue development, and auxin polar transport expanded significantly. These genes and gene families, which are involved in growth and development, likely shape the special morphology of high-altitude plants such as *R. nivale* subsp. *boreale* and regulate the different stages of their developmental cycle in response to environmental changes, thus better adapting to the extreme spatial and temporal heterogeneity of mountaintop ecosystems [12,55].

Low temperature (average annual temperature below 0 °C), low partial pressure of oxygen (for every 1,000 m increase in altitude, air pressure drops by about 11%) and rapid weather changes (annual average diurnal temperature exceeding 20 °C) are the main factors limiting alpine plant survival [56,57,58]. Under harsh alpine conditions, plants adapt by modifying their morphology, producing specific metabolites, and changing the distribution patterns of biomass [55]. In *R. nivale* subsp. *boreale,* we observed a significant expansion in the gene

family associated with BRs, which are pivotal for sustaining plant physiological functions and significantly contribute to enhancing cold tolerance, drought resistance, and antioxidative capabilities [59,60]. Hence, the expansion of the BR gene family probably enhanced the ability of *R. nivale* subsp. *boreale* to adapt to dramatically changing environments. Moreover, we speculated that the expansion of CYPs, which are associated with stress response, could potentially facilitate the successful adaptation of *R. nivale* subsp. *boreale* at high altitudes. To further understand the adaptability of *R. nivale* subsp. *boreale*, we assessed the dynamics of an important family of transcription factors (AP2/ERF), which is an important group of transcription factors responsive to abiotic stress [61], including key members that adapt to alpine conditions such as *ERF VIIs* and *CBFs/DREB1s*. *ERF VIIs* and *CBFs* are important transcription factors that respond to low oxygen partial pressures and temperatures [34,62]. Unexpectedly, the genes of *Rhododendron* for *ERF VIIs* and *CBFs* continued to be lost lost in low-oxygen, cold alpine environments. *R. nivale* subsp. *boreale* distributed over the alt. 4000 m is no exception. The high expression levels of *ERF VIIs* in *R. nivale* subsp. *boreale* suggests a potential adaptation to low oxygen environments, which warrants further experimental investigation. In response to temperature changes, *Rhododendron* species rely on several pathways to enhance their low-temperature resistance, including *CBFs*-mediated cold tolerance and an integrated regulatory network of ABA, the MAPK cascade, and Ca^{2+} signaling [63,64]. Our positive selection analysis results suggested that *M3K1* and *CNGC1,* which are associated with the MAPK cascade and Ca^{2+} signal transduction, might play important roles in low-temperature adaptation.

Polyploidy leads to rapid changes in gene expression and epigenetics, giving the polyploid

a significant selective advantage over its diploid progenitors and serving as a crucial mechanism for plants to swiftly adjust to severe environmental stress [6,18]. Moreover, differential splicing, which is a crucial mechanism in the eukaryotic stress response, changes rapidly after polyploidy and is associated with abiotic stress [65]. Certainly, the influence of genome doubling on phenotypes or life history traits can directly affect the likelihood of survival under challenging circumstances [18]. These include more viable seeds, more rapid growth, and stronger photosynthesis [19,66]. These characteristics provide plants with great advantages under adverse environmental conditions.

The decline in the fitness of autopolyploids, especially young autopolyploids, is usually attributed to multivalent chromosome pairing during meiosis and mutations in crossover (CO) frequency and distribution [23,67]. Consequently, autopolyploids experience significant disruptions in their developmental programs, resulting in a considerable reduction in seed production and a high incidence of aneuploid offspring [68]. Addressing these issues requires precise adaptive control of meiosis, such as reduced formation of multichromosome associations and reduced axis lengths [69]. The RECQ4a/4b (BLM)-TOP3α-RMI1 (BTR) complex plays a pivotal role in limiting CO outcomes and maintaining chromosome integrity [70,71]. Mutations of *RECQ4*, one of its members, significantly affect the stability of polyploid meiosis [72]. Notably, *TOP3α*, an important gene related to the BTR complex, exhibits positive selection in *R. nivale* subsp. *boreale* and may serve as a key factor in promoting accurate chromosomal segregation during meiosis in autopolyploids.

Our results revealed that *R. nivale* subsp. *boreale* distributed on the mountaintop is an autotetraploid, which might be mediated by the harsh environment at high altitudes.

Paleopolyploid events are shared among the other 10 *Rhododendron* species of diploids. Our conjecture regarding the alpine adaptation mechanisms of *R. nivale* subsp. *boreale* aligns with those of previous studies: cell wall modification, flavonoid biosynthesis, DNA repair, inhibition of chlorophyll synthesis, and auxin and BR biosynthesis and transduction are probably the main high-altitude adaptation pathways [12]. Polyploidization likely plays an important role in mountaintop survival because increased gene dosage can lead to enhanced stress tolerance, greater genetic diversity, and the potential for novel traits that enhance adaptability [18]. Notably, *TOP3α* is speculated to be an important gene during meiosis in autotetraploids, essential for the generation of normal gametes and implicated in the attenuation of CO events during meiosis. However, these hypotheses require verification through biological experiments. Moreover, the mechanism of the formation of natural polyploids remains to be fully elucidated, and the alpine environment, where polyploidy is concentrated, offers an ideal setting for investigation.

Overall, we assembled the first genome of Subge. *Rhododendron*, a rare high-altitude woody autotetraploid genome that provides an important resource for the domestication of high-altitude ornamentals and our understanding of polyploid origin and evolution in mountaintop ecosystems.

4. Materials and methods

4.1 Plant materials and sequencing

R. nivale subsp. *boreale* (NCBI:txid1701214) plant materials were collected from Baima Mountain, Dêqên County, Yunnan Province, China (99°4′13″E, 28°20′24″N, alt. 4287.5 m).

The plant materials were immersed in liquid nitrogen immediately after collection and preserved at -80 °C. High-quality DNA isolated from young leaves was used to create the libraries. Long-read libraries were constructed and sequenced using the PacBio Sequel II sequencing platform. To construct Hi-C libraries, genomic DNA was cross-linked with formaldehyde and digested using the MboI restriction enzyme into 300–500 bp fragments, which were sequenced on the BGI DNBseq sequencing platform. For short reads, DNA libraries were constructed and sequenced on the BGI DNBseq sequencing platform. Three biological replicates of roots, stems, leaves, and buds of *R. nivale* subsp. *boreale* were sampled. The cDNA libraries were constructed and sequenced on a BGI DNBseq sequencing platform.

4.2 Genome survey

Flow cytometry and *k-mer* analysis were used to evaluate the genome of *R. nivale* subsp. *boreale*. The following procedures were used for flow cytometry: preparation of nuclear suspension, DNA-specific staining, and testing. We selected *R. griersonianum* as an internal control. Graphical analysis was performed using ModFit LT 5.0 (www.vsh.com/products/mflt/index.asp) with a coefficient of variation (CV) controlled to within 5. For *k-mer* analysis, DNBseq short-reads clean data were used to count *k-mer* frequency with *k-mer* set to 21 using jellyfish v2.3.0 [73]. Genome size was estimated based on the 21 *k-mers* distribution. Ploidy was estimated using SmudgePlot v0.2.5 [29].

4.3 Genome assembly and scaffolding

The PacBio circular consensus sequencing (CCS) long-read data were assembled using Hifiasm v0.18.9 with Hi-C integration [74], Canu v1.9 [75], and HiCanu v2.2 [76]. We used the parameters of the genome of *Saccharum spontaneum* [16]. The integrity and continuity of

the assembly were assessed separately, and the highest quality assembly was used for the scaffolding. The ALLHiC pipeline was used to improve assembly at the chromosomal level based on five steps: pruning, partitioning, rescue, optimization, and construction [77]. Manual checks were conducted on potential misassemblies and corrected using Juicebox v1.11.08 [78]. Finally, the assembled genome was evaluated using Benchmarking Universal Single-Copy Orthologs (BUSCO) v5.4.6 [79], Merqury v1.3 [80], and Clipping information for Revealing Assembly Quality (CRAQ) v1.0.9 [81] using default parameters. Short reads mapped to the assembled genome using BWA v0.7.17-r1188 [82] and SAMtools v1.17 [83] were counted as properly paired.

4.4 Genome annotation

De novo prediction and homology alignment were used to identify whole-genome repeats. The LTRs were initially identified using LTRharvest (RRID:SCR_018970) [84] and LTR_Finder (RRID:SCR_015247) [85]. LTR_retriever v2.9.4 [86] was used to accurately identify LTR retrotransposons (LTR-RTs), generate a nonredundant LTR-RT library, and generate the LTR assembly index (LAI). A homology search was conducted to predict repeat elements using RepeatMasker v4.1.4 [87]. Transfer RNAs (tRNAs) were annotated using tRNAscan-SE (RRID:SCR_008637) v2.0.9 [88], and ribosomal RNAs (rRNAs) were identified using RNAmmer (RRID:SCR_017075) v1.2 [89]. Other noncoding RNAs, including miRNAs and snRNAs, were annotated by comparison using Infernal v1.1.4, with the Rfam database [90,91].

We combined ab initio, homolog and transcriptome-based strategies to predict the expression of high-quality protein-coding genes. In our transcriptome-based strategies, we used

HISAT2 (RRID:SCR_015530) v2.2.1 [92] to align clean reads of the transcriptome with the genome. Trinity (RRID:SCR_013048) v2.14.0 [93] and StringTie (RRID:SCR_016323) v2.2.1 [94] were used to assemble transcripts. BRAKER3 [95] and PASA (RRID:SCR_014656) v2.5.2 [96] were used to predict gene structure based on the assembled transcripts and to generate ab initio gene predictor training sets. For ab initio, SNAP (RRID:SCR_007936) [97], GlimmerHMM (RRID:SCR_002654) v3.0.1, and GeneID v1.4 [98] were used to annotate gene structures based on the training sets. For homology-based prediction, protein sequences from a total of eight species, namely, *Arabidopsis thaliana, Vitis vinifera, Glycine max, Nicotiana attenuata, Oryza sativa, R. ovatum, R. griersonianum,* and *R. mole*, were aligned with the genome of *R. nivale* subsp. *boreale* using GeMoMa (RRID:SCR_017646) v1.9 [99]. All gene structures annotated using the above approaches were integrated using the EVidenceModeler (EVM) [100]. Functional annotation of genes was performed using EggNOG (RRID:SCR_002456) v5.0 [101], and protein sequences were aligned to the UniProt database using BLAST v2.6.0 [102].

4.5 Identification of polyploid type

GenomeScope 2.0 [29] was used to count the proportion of nucleotide heterozygosity forms based on the 21 *k-mer* count distributions. AAAB < AABB indicates allotetraploidy, whereas AAAB > AABB indicates autotetraploidy. JCVI utility libraries [103] were used to analyze collinear relationships between haplotypes. To identify the relationship between different haplotypes and related species, 11 transcriptome datasets from seven related species from previous studies with four haplotypes and transcripts of *R. nivale* subsp. *boreale* were used to reconstruct the phylogenetic tree. StringTie v2.2.1 [94] was used to assemble transcripts. A

maximum likelihood (ML) tree was reconstructed using IQ-TREE v2.2.2.2 [104] with 1000 ultrafast bootstrap replicates after single-copy orthologs were identified by OrthoFinder v2.5.4 [105].

4.6 Comparative genomics analysis

The genomes of *Actinidia chinensis, Amborella trichopoda, Camptotheca acuminata, Davidia involucrata, Oryza sativa, Panicum hallii, R. delavayi, R. griersonianum, R. henanense* subsp. *lingbaoense, R. irroratum, R. molle, R. ovatum, R. ripense, R. simsii, R. vialii, Vaccinium darrowii,* and *Vitis vinifera* were used for comparative genomics analysis with our assembly of *R. nivale* subsp. *boreale*. Single-copy orthologs were identified based on protein sequences using OrthoFinder (RRID:SCR_017118) v2.5.4 [105]. The protein sequences in each single-copy orthogroup were aligned using MUSCLE (RRID:SCR_011812) v5.1 [106] and filtered using trimAI v1.4 [107] and used to construct a phylogenetic tree using IQ-TREE v2. 2.2.2 [104] with 1000 ultra-fast bootstrap replicates. The MCMCtree program in PAML (RRID:SCR_014932) v4.10 [108] was used to estimate the divergence times. Calibration times were obtained from the TimeTree database (http://timetree.org) and previous studies [4,109]. A total of four calibration points were used to calibrate age: angiosperms 168–194 Mya; monocots eudicots 142.1–163.5 Mya; *Rhododendron* crown 54.5 Mya; *Panicum hallii-Oryza sativa* 41.4–51.9 Mya. Based on the ultrametric tree, the expansion and contraction of gene families were estimated using CAFÉ 5 [110]. Functional enrichment analysis of Gene Ontology (GO) and Kyoto Encyclopedia of Genes and Genomes (KEGG) was performed using the R package clusterProfiler v4.8.3 [111]. Synteny between different species was identified and visualized using the MCscan pipeline in JCVI [103] and MCScanX [112] with default parameters. The *Ks*

values of the ortholog and paralog pairs were calculated using KaKs_Calculator v2.0 [113] after alignment with ParaAT v2.0 [114]. WGD times were estimated as T=*Ks*/2r (T is the WGD time and r is the rate of divergence). The value of r was obtained from a previous study [115].

4.7 Selective analysis

Based on 1,122 single-copy conserved orthologs from Ericales (10 *Rhododendrons, Va. darrowii,* and *Ac. Chinensis*), we performed the positive selection analysis acting on the *R. nivale* subsp. *boreale* clade by running separate aBSREL, Clade Model, MEME, and Contrast-FEL. A gene was considered a PSG when it met all model criteria. aBSREL was implemented in HyPhy v2.5.48 [116] with exploratory analysis, representing an improved version of traditional "branch-site" models. The aBSREL test models both site-level and branch-level nonsynonymous-to-synonymous mutation ratio ω heterogeneity but does not test for selection at specific sites. To obtain more accurate PSGs, we used clade model C (CmC) to check the consistency of the model in PAML [108]. This model tested the differential selection pressure between the foreground branches and background for each gene. CmC was then compared with the null model M2a_rel using likelihood-ratio tests (LRT) [117].

To obtain information on specific sites during episodic selection, we applied MEME and Contrast-FEL. MEME tests [118] for sites that were subjected to episodic positive or diversifying selection were performed for each gene. The MEME employs a mixed-effects maximum likelihood approach to test the hypothesis that individual sites are subject to episodic, positive, or diversifying selection. For each site, MEME infers two ω rate classes and the corresponding weights representing the probability that the site evolves under each corresponding ω rate class at a given branch. Contrast-FEL [119] was used to estimate the

difference in ω at each site between different branch sets in codon alignments. The false discovery rate (FDR) was used to correct for multiple comparisons.

To further understand the selection pressure characteristics of the high- and low-altitude genomes, we used KaKs_Calculator [113] to detect selected genes between *R. nivale* subsp. *boreale* (high altitude) and *R. ovatum* (low altitude). All genes with a P value < 0.05 and ω (Ka/Ks) > 1 were identified as candidate PSGs.

4.8 Gene expression analysis

Clean reads of the transcriptome were mapped to the genome using STAR and gene expression levels were estimated using STAR v2.7.10b [120]. Accurate quantification (transcripts per kilobase per million mapped reads) of genes was performed using RSEM v1.3.3 [121]. We selected the expression levels of single-match alleles to explore the differences in expression between alleles. The four alleles were compared pairwise to identify the differentially expressed alleles. Pairs of alleles exhibiting less than a twofold difference in expression were classified as neutral, whereas all other pairs were categorized as non-neutral, that is, DEL. [122]. We used the Kruskal–Wallis test to assess differences in median values among multiple independent samples. The level of significance was set at P value < 0.05.

4.9 Identification of duplicate gene modes

Different modes of duplicated gene pairs were identified using the DupGen Finder pipeline [123]. The duplicated gene pairs were divided into five categories: whole-genome duplicates (WGD), tandem duplicates (TD), proximal duplicates (PD), transposed duplicates (TRD), and dispersed duplicates (DSD).

4.10 Identification and analysis of key gene families

The AP2/ERF and cytochrome P450 (CYP) gene families were identified using HMMER (RRID:SCR_005305) v3.3.2 (HMMER.org). The structural domain files corresponding to AP2/ERF (PF00847) and CYP (PF00067) were obtained from the Pfam database (https://www.ebi.ac.uk/interpro/). A domain file is used as the first template to search for a family. The filtered domain sequences were used as species-specific templates in the second scan. The Pfam and CDD databases (https://www.ncbi.nlm.nih.gov/cdd/) were used to verify conserved domains. Conserved sequences containing the main domains were aligned using MAFFT (RRID:SCR_011811) v7.520 [124] and used to construct a phylogenetic tree of the gene family using FastTree v2.1.11 [125] with the GTR + CAT model. Phylogenetic analysis of *CBFs* and *ERF VIIs* was performed using IQ-TREE v2.2.2.2 with 1000 replicates [104]. Gene motifs were predicted using MEME software v5.5.1 and visualized using TBtools v2.003 [126]. BadiRate v1.35 [127] was used to estimate family turnover rates based on likelihood-based methods.

Data availability

The raw sequencing data of this study have been deposited in the Sequence Read Archive (SRA) at NCBI under Bioproject number PRJNA1040959. The genome assembly and annotation data are available on Figshare [128] and in the Genome Warehouse (GWH) at National Genomics Data Center (NGDC) under accession number GWHETKQ00000000.1. Supporting data is also available via the *GigaScience* database, GigaDB [129].

Author contribution statement

Zhen-Yu Lyu: Conceptualization, Methodology, Visualization, Formal analysis, Writing

- Original Draft, Writing - Review & Editing. **Shi-Kang Shen:** Conceptualization, Methodology, Writing - Original Draft, Writing - Review & Editing. **Si-Qi Wang:** Resources. **Xiong-Li Zhou:** Resources. **Rui Zhang:** Resources. **Gao-Ming Yang:** Cytological experiment. **Jie-Yu Zhang:** Cytological experiment. **Wen-Guang Sun:** Cytological experiment.

Funding

This study was supported by the Science and Technology Development Fund of Guidance from the Central Government to Locals in Yunnan Province (202207AB110016), Major Program for Basic Research Project of Yunnan Province (202101BC070002), National Natural Science Foundation of China (31870529), the Graduate Scientific Research Fund Project of Yunnan University (KC-22221373), and the Scientific Research Fund of Yunnan Provincial Education Department (2024Y003).

Conflict of Interest

The authors declare that they have no competing interests.

References:

1. Fang R, Min TL. The floristic study on the genus *Rhododendron*. Acta Botanica Yunnanica. 1995;17:359–79.

2. Chen YS, Deng T, Zhou Z, Sun H. Is the East Asian flora ancient or not? Natl Sci Rev. 2018;5:920–32.

3. Basnett S, Rengaian G. A Comprehensive Review on the taxonomy, ecology, reproductive biology, economic importance and conservation status of Indian Himalayan *Rhododendrons*. Bot Rev. 2022;88:505–44.

4. Xia XM, Yang MQ, Li CL, Huang SX, Jin WT, Shen TT, Wang F, Li XH, Yoichi W, Zhang LH, Zheng YR, Wang XQ. Spatiotemporal Evolution of the Global Species Diversity of *Rhododendron*. Mol Biol Evol. 2022;39:msab314.

5. Darlington CD, Wylie AP. Chromosome atlas of flowering plants. George Allen and Unwin Ltd. Londonn, UK; 1955. p. 217–8.

6. Zhang J, Peng HW, Xia FC, Wang W. A comparison of seed plants' polyploids between the Qinghai-Tibet Plateau alpine and the Pan-Arctic regions. Biodiversity Science. 2021;29:1470–80.

7. Liu B, Zhao FM, Zhou H, Xia YP, Wang XY. Photoprotection conferring plant tolerance to freezing stress through rescuing photosystem in evergreen *Rhododendron*. Plant Cell Environ. 2022;45:2093–108.

8. Popescu R, Kopp B. The genus *Rhododendron*: An ethnopharmacological and toxicological review. J Ethnopharmacol. 2013;147:42–62.

9. Guo X, Dong Z, Li Q, Wan DG, Zhong JB, Dong D, Huang MZ. Flavonoids from *Rhododendron nivale* Hook. f delay aging via modulation of gut microbiota and glutathione metabolism. Phytomedicine. 2022;104:154270.

10. Hu YB, Wang XP, Xu YC, Yang H, Tong ZY, Tian R, Xu SH, Yu L, Guo YL, Shi P, et al. Molecular mechanisms of adaptive evolution in wild animals and plants. Sci China Life Sci. 2023;66:453–95.

11. Marks R. A, Hotaling S, Frandsen P. B, VanBuren R. Representation and participation across 20 years of plant genome sequencing. Nat. Plants. 2021;7:1571–8.

12. Zhang X, Kuang TH, Dong WL, Qian ZH, Zhang HJ, Landis JB, Feng T, Li LJ, Sun YX, Huang JL, et al. Genomic convergence underlying high-altitude adaptation in alpine plants. J Integr Plant Biol. 2023;65:1620–35.

13. De Storme N, Geelen D. The impact of environmental stress on male reproductive development in plant: Biologival processes and molecular mechanisms. Plant Cell Environ. 2014;37:1–18.

14. Chen HT, Zeng Y, Yang YZ, Huang LL, Tang BL, Zhang H, Hao F, Li W, Li YH, Liu YB, et al. Allele-aware chromosome-level genome assembly and efficient transgene-free genome editing for the autotetraploid cultivated alfalfa. Nat Commun. 2020;11:2494.

15. Wang F, Xia ZQ, Zou ML, Zhao L, Jiang SR, Zhou Y, Zhang CJ, Ma YZ, Bao YT, Sun HH, et al. The autotetraploid potato genome provides insights into highly heterozygous species. Plant Biotechnol J. 2022;20:1996–2005.

16. Zhang Q, Qi YY, Pan HR, Tang HB, Wang G, Hua XT, Wang YJ, Lin LY, Li Z, Li YH, et al. Genomic insights into the recent chromosome reduction of autopolyploid sugarcane *Saccharum spontaneum*. Nat Genet. 2022;54:885–96.

17. Zhang HY, He Q, Xing LS, Wang RY, Wang Y, Liu Y, Zhou QH, Li XZ, Jia Z, Liu Z, et al. The haplotype-resolved genome assembly of autotetraploid rhubarb *Rheum officinale* provides insights into the genome evolution and massive accumulation of anthraquinones. Plant Commun. 2023;26:100677.

18. Van de Peer, Y, Ashman TL, Soltis PS, Soltis DE. Polyploidy: an evolutionary and ecological force in stressful times. Plant Cell. 2021;33:11–26.

19. Stevens AV, Nicotra AB, Godfree RC, Guja LK. Polyploidy affects the seed, dormancy and seedling characteristics of a perennial grass, conferring an advantage in stressful climates. Plant Biol. 2020;22:500–13.

20. Liu CJ, Wang YG. Does one subgenome become dominant in the formation and evolution of a polyploid? Ann Bot. 2023;131:11–6.

21. Wang KL, Deng PR, Yao Z, Dong JY, He Z, Yang P, Liu YB. Biogeographic patterns of polyploid species for the angiosperm flora in China. J Syst Evol. 2022;61:776–89.

22. Heslop-Harrison JS, Schwarzacher T, Liu Q. Polyploidy: its consequences and enabling role in plant diversification and evolution. Ann. Bot. 2023;131:1–10.

23. Cifuentes M, Grandont L, Moore G, Chevre AM, Jenczewski E. Genetic regulation of meiosis in polyploid species: new insights into an old question. New Phytol. 2010;186:29–36.

24. Grandont L, Jenczewski E, Lloyd A. Meiosis and its deviations in polyploid plants. Cytogenet. Genome Res. 2013;140:171–84.

25. Bomblies, K. Learning to tango with four (or more): the molecular basis of adaptation to polyploid meiosis. Plant Reprod. 2022;36:107–24.

26. Gou XW, Bian Y, Zhang A, Zhang HK, Wang B, Lv RL, Li JZ, Zhu B, Gong L, Liu B. Transgenerationally precipitated meiotic chromosome instability fuels rapid karyotypic evolution and phenotypic diversity in an artificially constructed allotetraploid wheat (AADD). Mol, Biol, Evol. 2018;35:1078–91.

27. Huang G, Wu Z, Percy RG, Bai MZ, Li Y, Frelichowski JE, Hu J, Wang K, Yu JZ, Zhu YX. Genome sequence of *Gossypium herbaceum* and genome updates of *Gossypium arboreum* and *Gossypium hirsutum* provide insights into cotton A-genome evolution. Nat. Genet. 2020;52:516–24.

28. Zhang X, Pandey MK, Wang, JP, Zhao KK, Ma XL, Li ZF, Zhao K, Gong FP, Guo BZ, Varshney R, et al. Chromatin spatial organization of wild type and mutant peanuts reveals high-resolution genomic architecture and interaction alterations. Genome Biol. 2021;22:315.

29. Ranallo-Benavidez TR, Jaron KS, Schatz MC. GenomeScope 2.0 and Smudgeplot for reference-free profiling of polyploid genomes. Nat Commun. 2020;11:1432.

30. Miao Y, Luo D, Zhao T, Du H, Liu Z, Xu Z, Guo L, Chen C, Peng S, Li JX, et al. Genome sequencing reveals chromosome fusion and extensive expansion of genes related to secondary metabolism in Artemisia argyi. Plant Biotechnol J. 2022;20:1902–1915.

31. Song AP, Su JS, Wang HB, Zhang ZR, Zhang XT, van de Peer Y, Chen F, Fang WM, Guan ZY, Zhang F, et al. Analyses of a chromosome-scale genome assembly reveal the origin and evolution of cultivated chrysanthemum. Nat Commun. 2023;14:2021.

32. Xu J, Wang XY, Guo WZ. The cytochrome P450 superfamily: key players in plant development and

defense. J Integr Agric. 2015;14:1673–86.

33. Feng K, Hou XL, Xing GM, Liu JX, Duan AQ, Xu ZS, Li MY, Zhuang J, Xiong AS. Advances in AP2/ERF super-family transcription factors in plant. Crit Rev Biotechnol. 2020;40:750–76.

34. Abbas M, Sharma G, Dambire C, Marquez J, Alonso-Blanco C, Proano K, Holdsworth MJ. An oxygen-sensing mechanism for angiosperm adaptation to altitude. Nature. 2022;606:565–9.

35. Nie YQ, Guo LY, Cui FQ, Shen YR, Ye XX, Deng DY, Wang S, Zhu JH, Wu, WW. Innovations and stepwise evolution of CBFs/DREB1s and their regulatory networks in angiosperms. J Integr Plant Biol. 2022;64: 2111–25.

36. Durst F, Nelson DR. 1995. Diversity and evolution of plant P450 and P450-reductases. Drug Metabol Drug Interact 12:189-206.

37. Shen SK, Zhou XL, Wang SQ, Lyu ZY, Zhang R, Liu Y, Long B. Protect fragile mountaintop ecosystems. Science. 2023;380:1114–5.

38. Wu XP, Zhang L, Wang XY, Zhang RA, Jin GH, Hu YT, Yang H, Wu ZZ, Ma YP, Zhang CJ, Wang JH. Evolutionary history of two evergreen *Rhododendron* species as revealed by chromosome-level genome assembly. Front. Plant Sci. 2023;14:1123707.

39. Xu P, Xu J, Liu G, Chen L, Zhou ZX, Peng WZ, Jiang YL, Zhao ZX, Jia ZY, Sun YH et al. The allotetraploid origin and asymmetrical genome evolution of the common carp *Cyprinus carpio*. Nat Commun. 2019;10:4625.

40. Blumthaler M, Ambach W, R Ellinger. Increase in solar UV radiation with altitude. J Photochem Photobiol B. 1997;39:130–4.

41. Kerstiens G. Cuticular water permeability and its physiological significance. J Exp Bot. 199647:1813–32.

42. Tossi V, Lombardo C, Cassia R, Lamattina L. Nitric oxide and flavonoids are systemically induced by UV-B in maize leaves. Plant Sci. 2012;193:103–9.

43. Choi YE, Lim S, Kim HJ, Han JY, Lee MH, Yang Y, Kim JA, Kim YS. Tobacco *NtLTP1*, a glandular-specific lipid transfer protein, is required for lipid secretion from glandular trichomes. Plant J. 2012;70:480–

91.

44. Trivedi P, Nguyen N, Klavins L, Kviesis J, Heinonen E, Remes J, Jokipii-Lukkari S, Klavins M, Karppinen K, Jaakola L, Haggman H. Analysis of composition, morphology, and biosynthesis of cuticular wax in wild type bilberry (*Vaccinium myrtillus* L.) and its glossy mutant. Food Chem. 2021;354:12957.

45. Gao HN, Jiang H, Lian XY, Cui JY, You CX, Hao YJ, Li YY. Identification and functional analysis of the *MdLTPG* gene family in apple. Crit. Rev. Biotechnol. 2021;163:338–47.

46. Emiliani J, Grotewold E, Ferreyra MLF, Casati P. Flavonols protect *Arabidopsis* plants against UV-B deleterious effects. Mol Plant. 2013;6:1376–9.

47. McKenzie R, Conner B, Bodeker G. Increased summertime UV radiation in New Zealand in response to ozone loss. Science. 1999;285:1709–11.

48. Lahari T, Lazaro J, Schroeder DF. *RAD4* and *RAD23/HMR* Contribute to *Arabidopsis* UV Tolerance. Genes. 2018;9:8.

49. Rai N, O'Hara A, Farkas D, Safronov O, Ratanasopa K, Wang F, Lindfors AV, Jenkins GI, Lehto T, Salojärvi J, et al. 2020. The photoreceptor *UVR8* mediates the perception of both UV-B and UV-A wavelengths up to 350 nm of sunlight with responsivity moderated by cryptochromes. Plant Cell and Environment 43:1513-1527.

50. Mohl P, von Buren R. S, Hiltbrunner E. Growth of alpine grasslandwill start and stop earlier under climate warming. Nat Commun. 2022;13:7398.

51. Liu JM, de Vos JM, Körner C, Yang Y. 2023. Phylogeny and phenotypic adjustments drive functional traits in across elevations in its diversity hot-spot in W-China. Alpine Botany 133:69-84.

52. Li YH, Zheng LY, Corke F, Smith C, Bevan MW. Control of final seed and organ size by the *DA1* gene family in *Arabidopsis thaliana*. Genes Dev. 2008;22:1331–6.

53. Titapiwatanakun B, Blakeslee JJ, Bandyopadhyay A, Yang H, Mravec J, Sauer M, Cheng Y, Adamec J, Nagashima A, Geisler M, et al. *ABCB19/PGP19* stabilises *PIN1* in membrane microdomains in *Arabidopsis*. Plant J. 2009;57:27–44.

54. Gao, YS, Badejo AA, Sawa Y, Ishikawa T. Analysis of two l-Galactono-1,4-Lactone-Responsive genes

with complementary expression during the development of Arabidopsis thaliana. Plant Cell Physiol. 2012;53:592–601.

55. Sun H, Niu Y, Chen YS, Song B, Liu CQ, Peng DL, Chen JG, Yang Y. Survival and reproduction of plant species in the Qinghai-Tibet Plateau. J Syst Evol. 2014;52:378–96.

56. Apte CV. Barometric Pressure at High Altitude: Revisiting West's Prediction Equation, and More. High Alt Med Biol. 2023;24:85–93.

57. Li XT, Guo W, Li SH, Zhang JZ, Ni XN. The different impacts of the daytime and nighttime land surface temperatures on the alpine grassland phenology. Ecosphere. 2021;12:e03578.

58. Wu QB, Liu YZ. Ground temperature monitoring and its recent change in Qinghai-Tibet Plateau. Cold Reg Sci Technol. 2004;38:85–92.

59. Clouse SD, Sasse JM. Brassinosteroids: Essential regulators of plant growth and development. Annu Rev Plant Physiol, Plant Mol Biol. 1998;49:427–51.

60. Chaudhuri A, Halder K, Abdin MZ, Majee M, Datta A. Abiotic stress tolerance in plants: brassinosteroids navigate competently. Int J Mol Sci. 2022;23:14577.

61. Riechmann JL, Meyerowitz EM. 1998. The AP2/EREBP family of plant transcription factors. Biol Chem 379:633-646.

62. Thomashow MF. Plant cold acclimation: Freezing tolerance genes and regulatory mechanisms. Annu Rev Plant Physiol Plant Mol Biol. 1999;50:571–99.

63. Cao K, Zhang ZY, Fan H, Tan Y, Xu HW, Zhou XF. Comparative transcriptomic analysis reveals gene expression in response to cold stress in *Rhododendron aureum* Georgi. Theor Exp Plant Physiol. 2022;34:347–66.

64. Zhang QY, Li Y, Cao K, Xu HW, Zhou XF. Transcriptome and proteome depth analysis indicate ABA, MAPK cascade and Ca^{2+} signaling co-regulate cold tolerance in *Rhododendron chrysanthum* Pall. Front Plant Sci. 2023;14:1146663.

65. Staiger D, Brown JWS. Alternative splicing at the intersection of biological timing, development, and stress responses. Plant Cell. 2013;25:3640–56.

66. Mao HT, Chen MY, Su YQ, Wu N, Yuan M, Yuan S, Brestic M, Zivcak M, Zhang HY, Chen Y. Comparison on photosynthesis and antioxidant defense systems in wheat with different ploidy levels and octoploid Triticale. Int J Mol Sci. 2018;19:3006.

67. Parra-Nunez P, Fernández-Jiménez N, Pachon-Penalba M, Sanchez-Moran E, Pradillo M, Santos JL. 2024. Synthetically induced autotetraploids provide insights into the analysis of meiotic mutants with altered crossover frequency. New Phytologist 241:197-208.

68. Singliarova B, Hojsgaard D, Muller-Scharer H, Mraz P. The novel expression of clonality following whole-genome multiplication compensates for reduced fertility in natural autopolyploids. Proc Biol Sci. 2023;290:20230389.

69. Morgan C, Zhang HK, Henry CE, Franklin FCH, Bomblies K. Derived alleles of two axis proteins affect meiotic traits in autotetraploid *Arabidopsis arenosa*. Proc Natl Acad Sci USA. 2020;117:8980–8988.

70. Séguéla-Arnaud M, Choinard S, Larchevêque C, Girard C, Froger N, Crismani W, Mercier R. 2016. *RMI1* and *TOP3α* limit meiotic CO formation through their C-terminal domains. Nucleic Acids Research 45:1860-1871.

71. Séguéla-Arnaud M, Crismani W, Larchevêque C, Mazel J, Froger N, Choinard S, Lemhemdi A, Macaisne N, Van Leene J, Gevaert K, et al. 2015. Multiple mechanisms limit meiotic crossovers: TOP3alpha and two BLM homologs antagonize crossovers in parallel to FANCM. Proc Natl Acad Sci U S A 112:4713-4718.

72. Bazile J, Nadaud I, Lasserre-Zuber P, Kitt J, De Oliveira R, Choulet F, Sourdille P. 2024. *TaRECQ4* contributes to maintain both homologous and homoeologous recombination during wheat meiosis. Frontiers in Plant Science 14.

73. Marcais G, Kingsford C. A fast, lock-free approach for efficient parallel counting of occurrences of k-mers. Bioinformatics. 2011;27:764–70.

74. Cheng HY, Concepcion T, Feng XW, Zhang HW, Li H. Haplotype-resolved de novo assembly using phased assembly graphs with hifiasm. Nat Methods. 2021;18:170–5.

75. Koren S, Rhie A, Walenz BP, Dilthey AT, Bickhart DM, Kingan SB, Hiendleder S, Williams JL, Smith TPL, Phillippy AM. De novo assembly of haplotype-resolved genomes with trio binning. Nat Biotechnol. 2018;36:1174–82.

76. Nurk S, Walenz BP, Rhie A, Vollger MR, Logsdon GA, Grothe R, Miga KH, Eichler EE, Phillippy AM, Koren S. HiCanu: accurate assembly of segmental duplications, satellites, and allelic variants from high-fidelity long reads. Genome Res. 2020;30:1291–305.

77. Zhang XT, Zhang SC, Zhao Q, Ming R, Tang HB. Assembly of allele-aware, chromosomal-scale autopolyploid genomes based on Hi-C data. Nat Plants. 2019;5:833–845. doi:10.1038/s41477-019-0487-8.

78. Durand NC, Robinson JT, Shamim S, Machol I, Mesirov P, Lander ES, Aiden EL. Juicebox provides a visualization system for Hi-C contact maps with unlimited zoom. Cell Syst. 2016;3:99–101.

79. Simao FA, Waterhouse RM, Ioannidis P, Kriventseva EV, Zdobnov EM. BUSCO: assessing genome assembly and annotation completeness with single-copy orthologs. Bioinformatics. 2015;31:3210–2.

80. Rhie A, Walenz BP, Koren S, Phillippy AM. Merqury: reference-free quality, completeness, and phasing assessment for genome assemblies. Genome Biol. 2020;21:245.

81. Li KP, Xu P, Wang JP, Yi X, Jiao YN. Identification of errors in draft genome assemblies at single-nucleotide resolution for quality assessment and improvement. Nat Commun. 2023;14:6556.

82. Li H, Durbin R. Fast and accurate short read alignment with Burrows-Wheeler transform. Bioinformatics. 2009;25:1754–60.

83. Li H, Handsaker B, Wysoker A, Fennell T, Ruan J, Homer N, Marth G, Abecasis G, Durbin R. The Sequence Alignment/Map format and SAMtools. Bioinformatics, 2009;25:2078–9.

84. Ellinghaus D, Kurtz S, Willhoeft U. LTRharvest, an efficient and flexible software for de novo detection of LTR retrotransposons. BMC Bioinformatics. 2008;9:18.

85. Zhao X, Wang H. LTR_FINDER: an efficient tool for the prediction of full-length LTR retrotransposons. Nucleic Acids Res. 2007;35:W265–W268.

86. Ou SJ, Jiang N. LTR_retriever: a highly accurate and sensitive program for identification of long terminal repeat retrotransposons. Plant Physiol. 2018;176:1410–22.

87. Tarailo-Graovac M, Chen NS. Using RepeatMasker to identify repetitive elements in genomic sequences. Current protocols in bioinformatics. 2009. Chapter 4:4.10.1–4.10.14. doi:10.1002/0471250953.bi0410s25

88. Lowe TM, Eddy SR. tRNAscan-SE: A program for improved detection of transfer RNA genes in genomic

sequence. Nucleic Acids Res. 1997;25:955–64.

89. Lagesen K, Hallin P, Rodland EA, Staerfeldt HH, Rognes T, Ussery DW. RNAmmer: consistent and rapid annotation of ribosomal RNA genes. Nucleic Acids Res. 2007;35:3100–8.

90. Nawrocki EP, Burge SW, Bateman A, Daub J, Eberhardt RY, Eddy SR, Floden EW, Gardner PP, Jones TA, Tate J, et al. Rfam 12.0: updates to the RNA families database. Nucleic Acids Res. 2015;43:D130–D137.

91. Nawrocki EP, Eddy SR. Infernal 1.1: 100-fold faster RNA homology searches. Bioinformatics. 2013;29:2933–5.

92. Kim D, Paggi JM, Park C, Bennett C, Salzberg SL. Graph-based genome alignment and genotyping with HISAT2 and HISAT-genotype. Nat Biotechnol. 2019;37:907–15.

93. Haas BJ, Papanicolaou A, Yassour M, Grabherr M, Blood PD, Bowden J, Couger MB, Eccles D, Li B, Lieber M, et al. De novo transcript sequence reconstruction from RNA-seq using the Trinity platform for reference generation and analysis. Nat. Protoc. 2013;8:1494–512.

94. Pertea M, Pertea GM, Antonescu CM, Chang TC, Mendell JT, Salzberg SL. StringTie enables improved reconstruction of a transcriptome from RNA-seq reads. Nat Biotechnol. 2015;33:290–5.

95. Hoff KJ, Lange S, Lomsadze A, Borodovsky M, Stanke M. BRAKER1: Unsupervised RNA-Seq-Based fenome annotation with GeneMark-ET and AUGUSTUS. Bioinformatics. 2016;32:767–769.

96. Haas BJ, Delcher AL, Mount SM, Wortman JR, Smith RK, Hannick LI, Maiti R, Ronning CM, Rusch DB, Town CD, et al. Improving the *Arabidopsis* genome annotation using maximal transcript alignment assemblies. Nucleic Acids Res. 2003;31:5654–66.

97. Korf I. Gene finding in novel genomes. BMC Bioinformatics. 2004;5:59. doi:10.1186/1471-2105-5-59.

98. Blanco E, Genis P, Roderic G. Using geneid to identify genes. Current protocols in bioinformatics. 2007. 4:4.3.1–4.3.28.

99. Keilwagen J, Hartung F, Grau J. GeMoMa: Homology-Based gene prediction utilizing intron position conservation and RNA-seq data. Methods Mol Biol. 2019;1962:161–77.

100. Haas BJ, Salzberg SL, Zhu W, Pertea M, Allen J. E, Orvis J, White O, Buell CR, Wortman JR. Automated eukaryotic gene structure annotation using EVidenceModeler and the program to assemble spliced

alignments. Genome Biol. 2008;9:R7.

101. Huerta-Cepas J, Szklarczyk D, Heller D, Hernandez-Plaza A, Forslund SK, Cook H, Mende DR, Letunic I, Rattei T, Jensen LJ, et al. eggNOG 5.0: a hierarchical, functionally and phylogenetically annotated orthology resource based on 5090 organisms and 2502 viruses. Nucleic Acids Res. 2019;47:D309–D314.

102. McGinnis S, Madden TL. BLAST: at the core of a powerful and diverse set of sequence analysis tools. Nucleic Acids Res. 2004;32:W20–W25.

103. Tang H, Bowers JE, Wang X, Ming R, Alam M, Paterson AH. 2008. Synteny and collinearity in plant genomes. Science 320:486–488.

104. Nguyen LT, Schmidt HA, von Haeseler A, Minh BQ. IQ-TREE: A fast and effective stochastic algorithm for estimating maximum-likelihood phylogenies. Mol Biol Evol. 2015;32:268–74.

105. Emms DM, Kelly S. OrthoFinder: phylogenetic orthology inference for comparative genomics. Genome Biol. 2019;20:238.

106. Edgar RC. MUSCLE: multiple sequence alignment with high accuracy and high throughput. Nucleic Acids Res. 2004;32:1792–7.

107. Capella-Gutierrez S, Silla-Martinez JM, Gabaldon T. trimAl: a tool for automated alignment trimming in large-scale phylogenetic analyses. Bioinformatics. 2009;25:1972–3.

108. Yang ZH. PAML 4: Phylogenetic analysis by maximum likelihood. Mol. Biol. Evol. 2007;24:1586–91.

109. Ma YZ, Mao XX, Wang J, Zhang L, Jiang YZ, Geng YY, Ma T, Cai LM, Huang SQ, Hollingsworth P, et al. Pervasive hybridization during evolutionary radiation of *Rhododendron* subgenus *Hymenanthes* in mountains of southwest China. Natl. Sci. Rev. 2022;9:nwac276.

110. Mendes FK, Vanderpool D, Fulton B, Hahn MW. CAFE 5 models variation in evolutionary rates among gene families. Bioinformatics. 2020;36:5516–8.

111. Wu TZ, Hu EQ, Xu SB, Chen MJ, Guo PF, Dai ZH, Feng TZ, Zhou L, Tang WL, Zhan L, et al. clusterProfiler 4.0:A universal enrichment tool for interpreting omics data. Innovation. 2021;2:100141.

112. Wang YP, Tang HB, DeBarry JD, Tan X, Li JP, Wang XY, Lee TH, Jin HZ, Marler B, Guo H, et al. MCScanX: a toolkit for detection and evolutionary analysis of gene synteny and collinearity. Nucleic Acids

Res. 2012;40:e49.

113. Wang Dapeng, Zhang YB, Zhang Z, Zhu J, Yu J. KaKs_Calculator 2.0: a toolkit incorporating gamma-series methods and sliding window strategies. Genom Proteom Bioinf. 2010;8:77–80.

114. Zhang Z, Xiao JF, Wu JY, Zhang HY, Liu GM, Wang XM, Dai L. ParaAT: A parallel tool for constructing multiple protein-coding DNA alignments. Biochem Biophys Res Commun. 2012;419:779–881.

115. Yang FS, Nie S, Liu, H, Shi TL, Tian XC, Zhou SS, Bao YT, Jia KH, Gou JF, Zhao W, et al. Chromosome-level genome assembly of a parent species of widely cultivated azaleas. Nat Commun. 2020;11:5269.

116. Pond SLK, Frost SDW, Muse SV. HyPhy: hypothesis testing using phylogenies. Bioinformatics. 2005;21:676–9. doi:10.1093/bioinformatics/bti079.

117. Smith MD, Wertheim JO, Weaver S, Murrell B, Scheffler K, Pond SLK. Less Is More: An Adaptive Branch-Site Random Effects Model for Efficient Detection of Episodic Diversifying Selection. Mol Biol Evol. 2015;32:1342–53.

118. Murrell B, Wertheim JO, Moola S, Weighill T, Scheffler K, Pond SLK. 2012. Detecting Individual Sites Subject to Episodic Diversifying Selection. PLoS Genet;8:e1002764..

119. Pond SLK, Wisotsky SR, Escalante A, Magalis BR, Weaver S. Contrast-FEL-A Test for Differences in Selective Pressures at Individual Sites among Clades and Sets of Branches. Mol Biol Evol. 2021;38:1184–98.

120. Dobin A, Davis CA, Schlesinger F, Drenkow J, Zaleski C, Jha S, Batut P, Chaisson M, Gingeras TR. STAR: ultrafast universal RNA-seq aligner. Bioinformatics. 2013;29:15–21.

121. Li B, Dewey CN. RSEM: accurate transcript quantification from RNA-Seq data with or without a reference genome. BMC Bioinformatics. 2011;12:323.

122. Zhang JS, Zhang XT, Tang HB, Zhang Q, Hua XT, Ma XK, Zhu F, Jones T, Zhu XG, Bowers J, et al. Allele-defined genome of the autopolyploid sugarcane *Saccharum spontaneum* L. Nat Genet. 2018;50:1565–73.

123. Qiao X, Li QH, Yin H, Qi KJ, Li LT, Wang RZ, Zhang SL, Paterson AH. Gene duplication and evolution

in recurring polyploidization-diploidization cycles in plants. Genome Biol. 2019;20:38.

124. Katoh K, Standley DM. MAFFT multiple sequence alignment software version 7: improvements in performance and usability. Mol Biol Evol. 2013;30:772–80.

125. Price MN, Dehal PS, Arkin AP. 2010. FastTree 2--approximately maximum-likelihood trees for large alignments. Plos One 5:e9490.

126. Chen CJ, Chen H, Zhang Y, Thomas HR, Frank MH, He YH, Xia R. TBtools: an integrative toolkit developed for interactive analyses of big biological data. Mol Plant. 2020;13:1194–202.

127. Librado P, Vieira FG, Rozas J. BadiRate: estimating family turnover rates by likelihood-based methods. Bioinformatics. 2012;28:279–81.

128. Lyu Z, Zhou X, Wang S, Yang G, Sun W, Zhang J, Zhang R, Shen S. Assembly and annotation of Rhododendron sp. genome (Rhonb_v1). Figshare. 2024. https://doi.org/10.6084/m9.figshare.26047735.v4

129. Lyu Z, Zhou X, Wang S, Yang G, Sun W, Zhang J, Zhang R, Shen S. Supporting data for "The first high-altitude autotetraploid haplotype-resolved genome assembled (Rhododendron nivale subsp. boreale) provides new insights into mountaintop adaptation". GigaScience Database. 2024. https://doi.org/10.5524/102563

Figure titles

Figure 1. Habitat and genomic characteristics of *R. nivale* subsp. *boreale*. A. habitat; B. habit; C. genome landscape, a, 52 pseudochromosomes, which belong to 13 homologous groups, and the length of the pseudochromosome; b, gene density; c, GC density; d, transposon element density; e, copia density; f, gypsy density; g, tandem repeat density; curved lines inside the circles link syntenic genes between different pseudochromosomes, the synteny between haplotype 1 and haplotype 2 is indicated in red, the synteny between haplotype 1 and haplotype 3 is indicated in green, the synteny between haplotype 1 and haplotype 4 is indicated in yellow. D. Hi-C heatmap for assembled pseudochromosomes; E. Smudgeplot analysis based on 21 *k-mers*.

Figure 2. Phylogenetic and comparative analysis between related species and haplotypes. A. Dot plot between *R. nivale* subsp. *boreale* and *R. ovatum*. B. Syntenic blocks between four haplotypes. C. phylogenetic relationships of Subsect. *Lapponica* based on the maximum likelihood (ML) analysis; yellow and green blocks show *R. nivale* and the sister clade of *R. nivale*, respectively; red block represents the data generated in this study (n1, n2, n3, n4, and *R. nivale* subsp. *boreale* represent the four haplotypes and transcriptome of *R. nivale* subsp. *Boreale,* respectively) and the blue block represents downloaded species data. D. Gene family characteristics between four haplotypes.

Figure 3. Comparative genomic analysis. A. ML phylogenetic tree showing the relationship between *R. nivale* subsp. *boreale* and 18 other species. Estimated divergence times (Mya, million years ago) are labeled at nodes in black. Bootstrap values are displayed on the nodes in circles (100%) and squares (≥ 95%). Expansion (orange) and contraction (blue) of gene families are shown on the branch, contraction and expansion of ancestors are represented by a pie chart, and extant species are indicated by numbers. WGD and WGT events are marked with D and T, respectively. B. A number of other orthologs, unique paralogs, multicopy orthologs and single-copy orthologs in 19 species. C. *Ks* of paralogs frequency distribution chart of seven species, namely, six Ericales (*Actinidia*, *Vaccinium* and Subg. *Hymenanthes*, Subg. *Furthermore*, Subg. *Rhododendron*, Subg. *Tsutsusi* one species each) and one *Vi. vinifera*, polyploidization events are represented by dotted lines. D. Homologous gene dot plots between *R. nivale* subsp. *boreale* and *Vi. vinifera*. The red box exemplifies the orthologous ratio of 1:2 between *Vi. vinifera* and *R. nivale* subsp. *boreale*.

Figure 4. KEGG and GO enrichment and gene duplication analysis of *R. nivale* subsp. *boreale*. A. KEGG (left) and GO (right) enrichment of genes in significantly expanded gene families. B. Venn diagram showing the number of shared and specific gene duplications between the significantly expanded genes (SEGs) and five categories of duplications (DSD, dispersed duplications; PD, proximal duplications; TD, tandem duplications; TRD, transposed duplications; WGD, whole genome duplications). C. *Ka/Ks* ratios of the five types of duplications. D. KEGG pathway enrichment analysis of the five duplication types.

Figure 5. Single-matched allelic expression analysis. A. The total amount of single-match allelic expression of 52 pseudochromosomes; the colors represent 13 homologous groups (HGs). B. Heatmap clustering analysis of single-match alleles in screening the position of DELs in *R. nivale* subsp. *boreale*. Each row represents a set of differentially expressed alleles, and each column represents a chromosome. The heatmap shows a homologous group.

Figure 6. Identification and evolution of key family and genes for adaptation to low mountaintop temperature and hypoxia. A. Rootless ML phylogenetic tree based on ultrafast 1,000 bootstrap samplings showed diversified AP2/ERF superfamily in 13 species, including 10 *Rhododendron* species and *Ar. thaliana*, Kiwifruit, *Va. darrowii.* The color of the clades indicates five subfamilies of the AP2/ERF superfamily. The labels are differently colored according to species. B. Schematic diagram of the gain and loss of key genes in 12 species of Ericales; numbers in pink and blue depict *ERF VII* and *CBF* gene family turnover. The numbers in the rectangles and circles represent the number of genes in ancestral and existing species. The + and − signs represent the gain and loss of genes, respectively. C. Expression levels of *ERF VIIs* and *CBFs*. The numbers in parentheses indicate the number of genes that are expressed. P represents the adjusted P value.

Figure 7. Characteristics of Ericales Cytochrome P450 (CYP). A. ML phylogenetic tree showing the relationship between 10 CYP clans (higher order groupings of CYP families). B. Heatmap showing the number of clan members for each species. C. Phylogenetic tree of the CYP members of 13 species based on GTR (generalized time-reversible). Different clans are represented by different colors. D. Number of CYP genes produced by duplication events in 10 species of *Rhododendron*.

Figure 1

Click here to access/download;Figure;Fig1.pdf

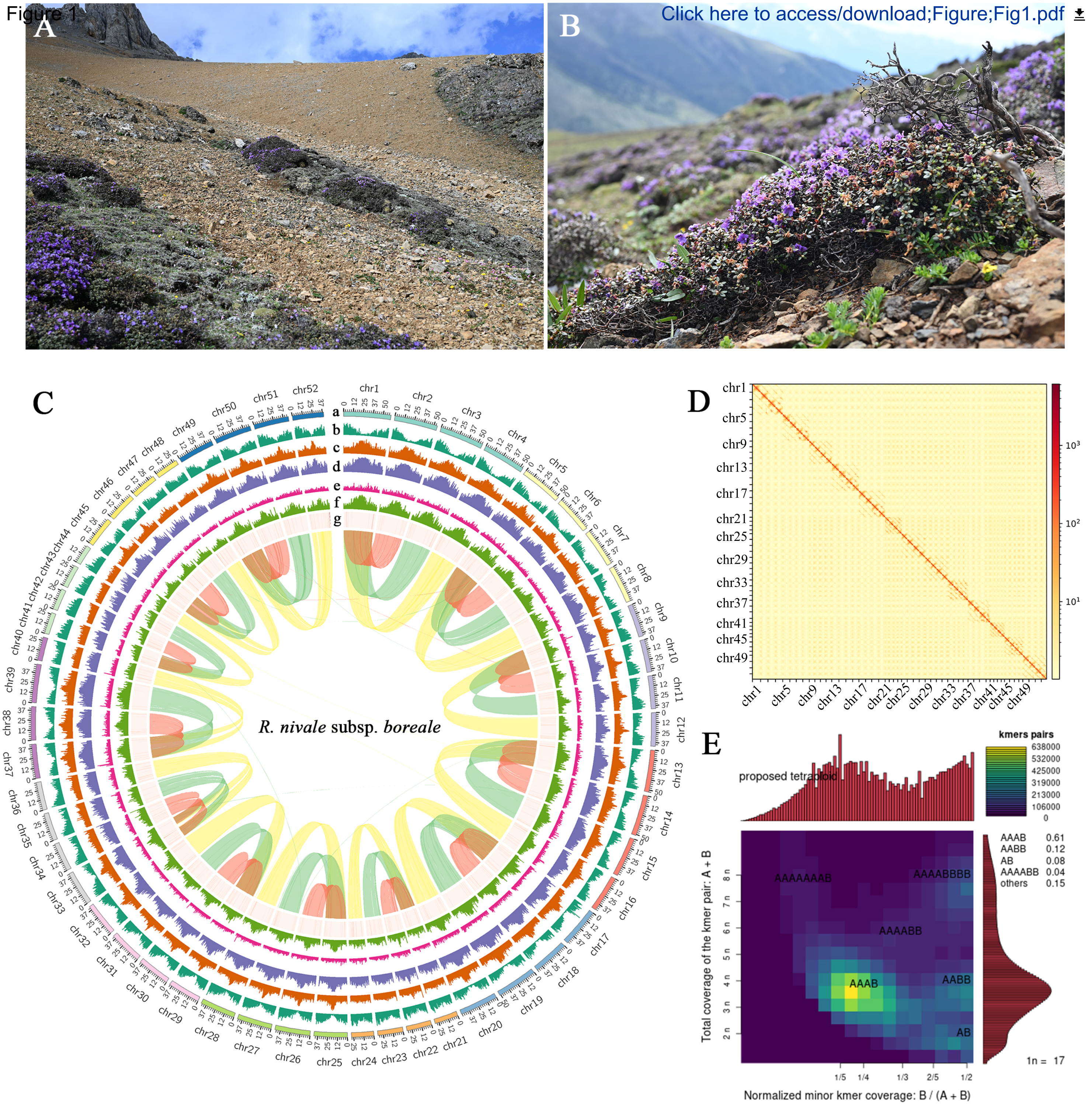

Figure 2

Click here to access/download;Figure;Fig2.pdf

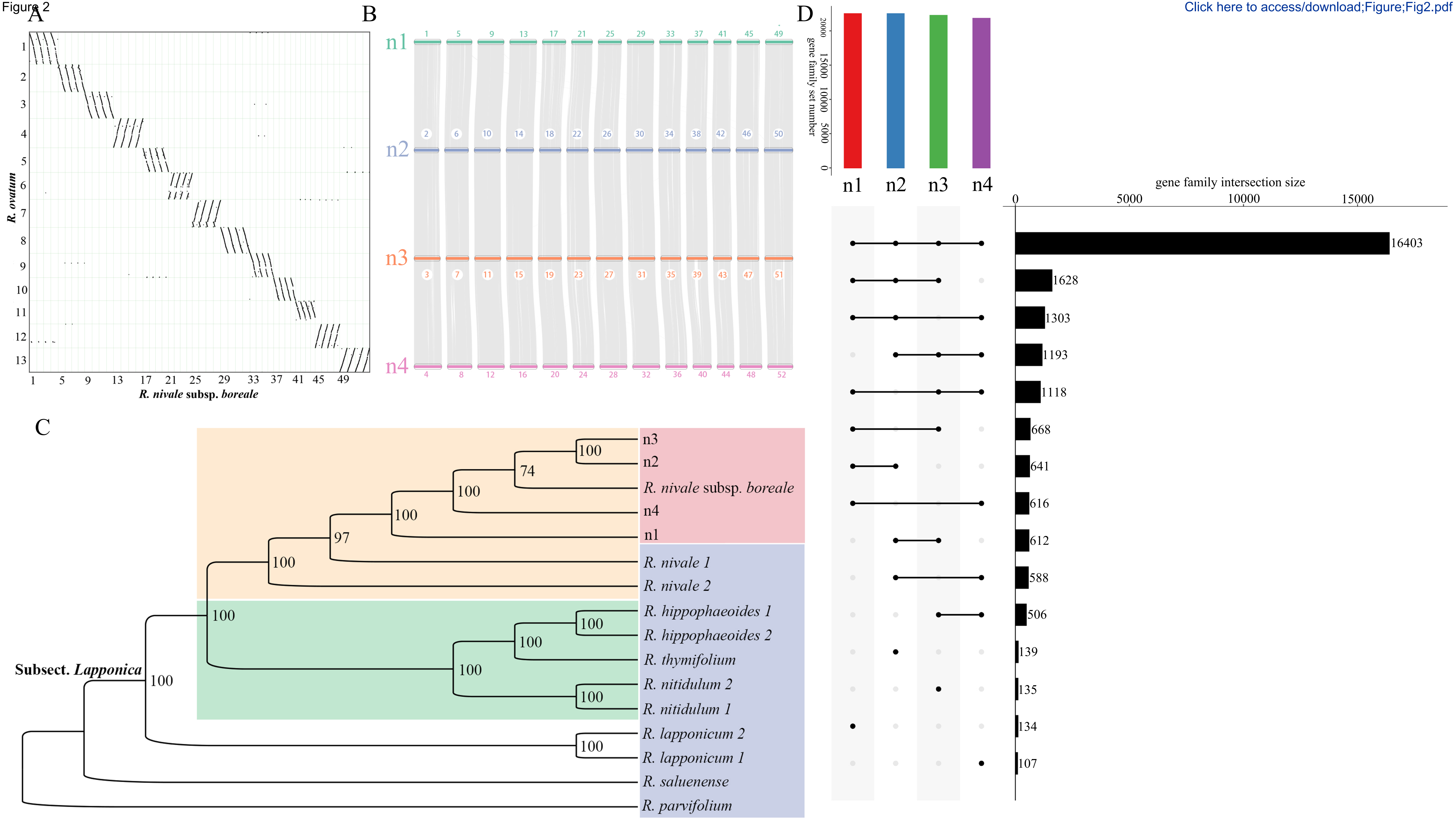

Figure 3

Click here to access/download;Figure;Fig3.pdf

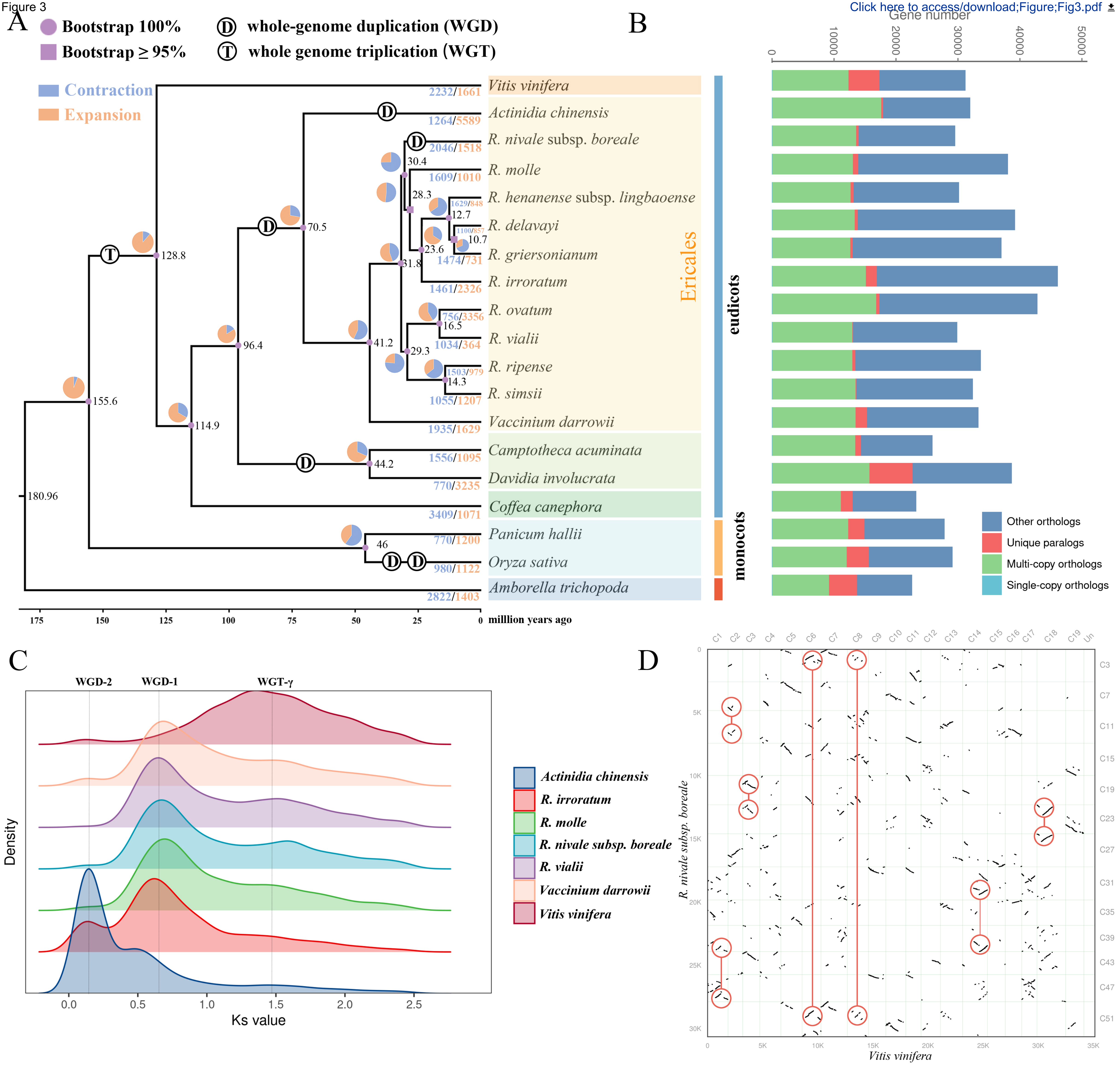

Figure 4

Click here to access/download;Figure;Fig4.pdf

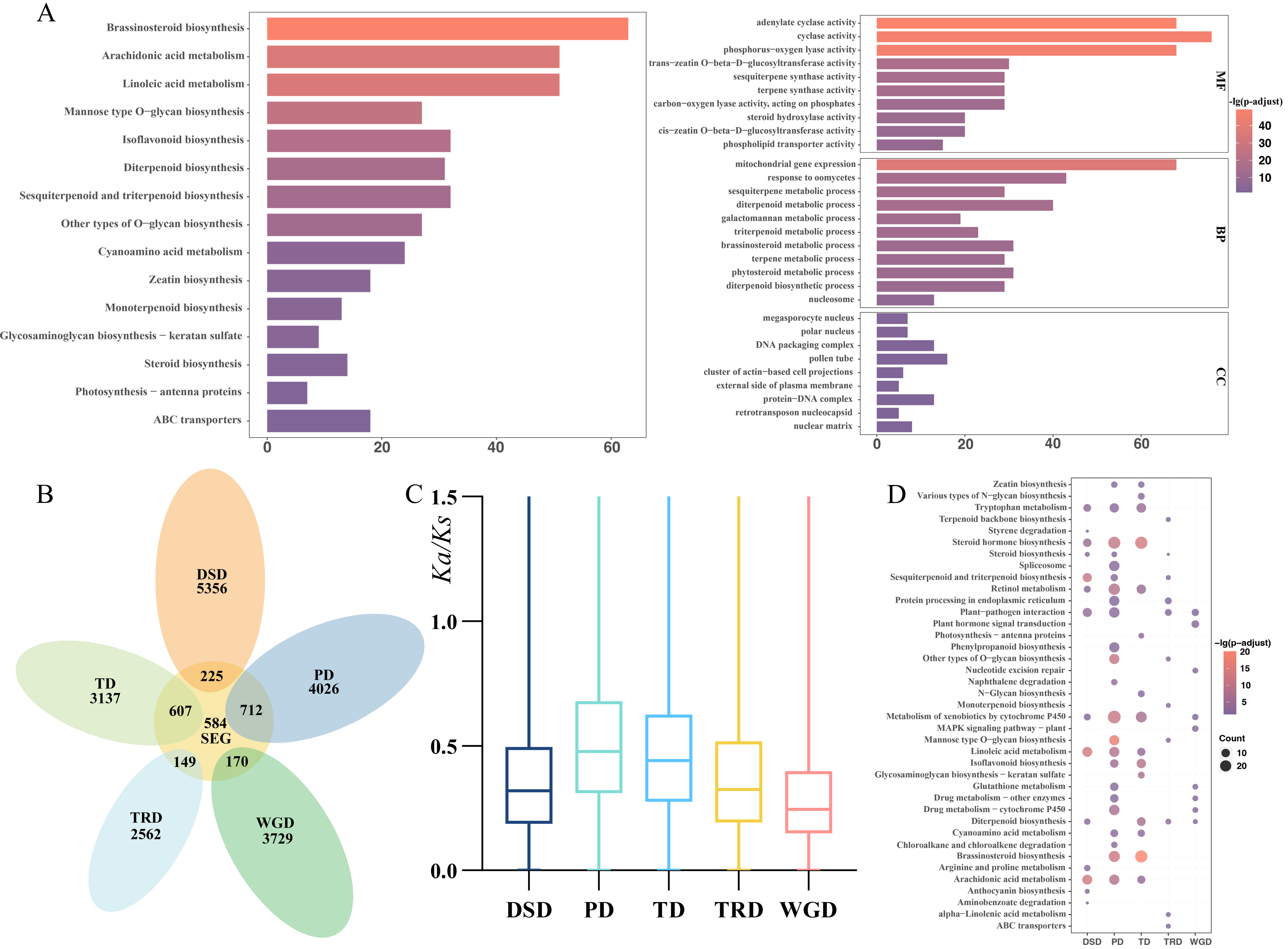

Figure 5

Click here to access/download;Figure;Fig5.pdf

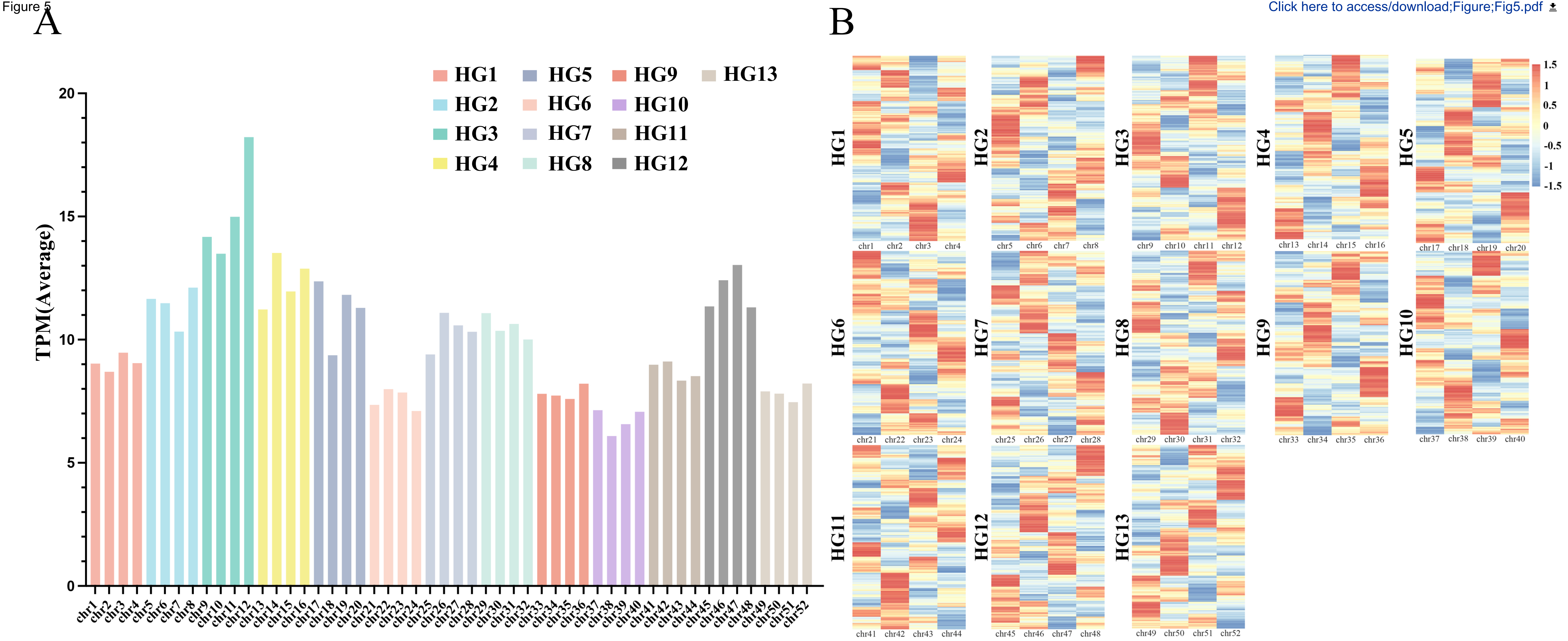

Figure 6

Click here to access/download;Figure;Fig6.pdf

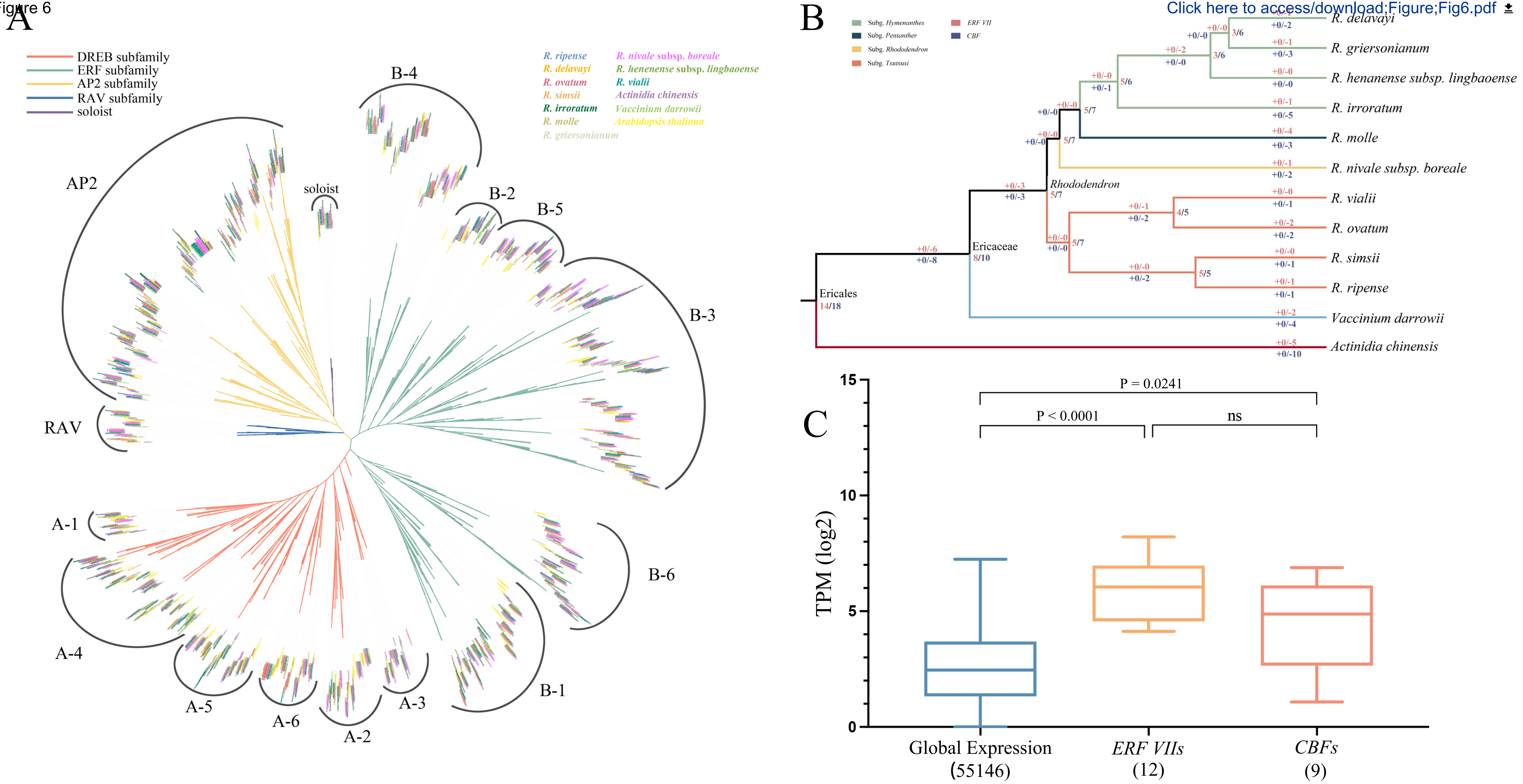

Figure 7

Click here to access/download;Figure;Fig7.pdf

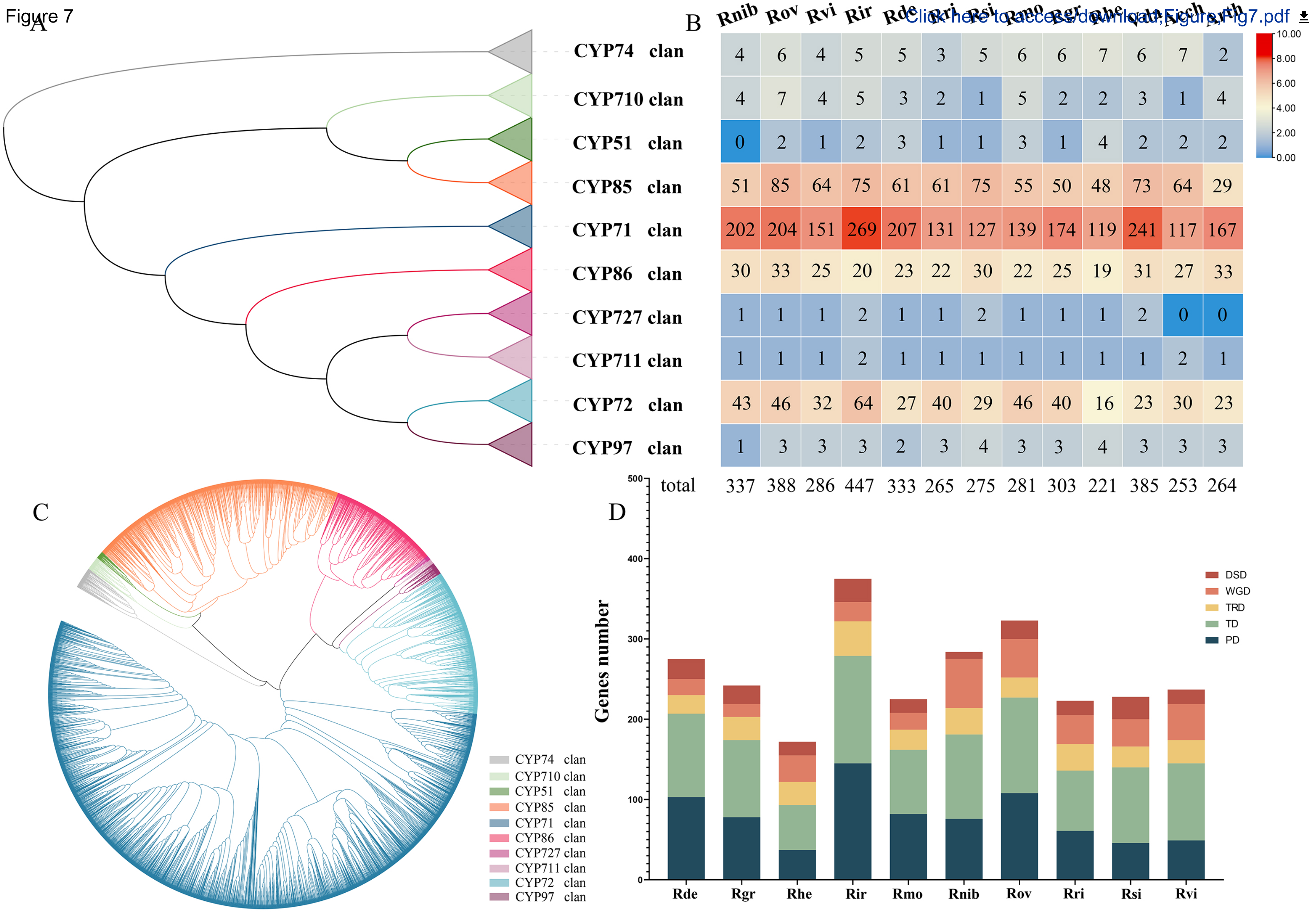

Supplementary Figure

Click here to access/download
Supplementary Material
Supplementary Figure.docx

Supplementary Table

Click here to access/download
Supplementary Material
Supplementary Table.xls

Personal Cover Click here to access/download;Personal Cover;Respond_to_editor_and_reviewers.docx

June 19, 2024

Dear Editor and Reviewers,

Thank you for the feedback on our manuscript "The first high-altitude autotetraploid haplotype-resolved genome assembled (*Rhododendron nivale* subsp. *boreale*) provides new insights into mountaintop adaptation " (GIGA-D-23-00395R1).

In this version, we double-checked all the data and conclusions and improved our manuscript based on reviewer comments. Additionally, we have submitted the genome sequences to the National Genomics Data Center (NGDC) and included the accession number (GWHETKQ00000000.1) in the Data Availability section.

We have marked the changes in blue in the revised manuscript to facilitate your review. Thank you again for considering our work. We hope this manuscript meets the requirements for publication at Gigascience.

Best regards

Sincerely yours,

Shi-Kang Shen

Reviewer #1:

Thank you for reviewing our manuscript. We have made improvements to the manuscript in response to your comments. The point-by-point response follows:

> **Comment:** line 42 - Spell out the abbreviation for Group VII Ethylene Response Factors in the abstract.

>> **Response:** Done. "Additionally, the expression levels of the (Group VII ethylene response factor transcription factors) *ERF VIIs* were significantly higher than the mean global gene expression." (line 42)

> **Comment:** line 112- what mountain top, I know this is in the methods but needs clarifying here too.

>> **Response:** We have clarified this part. "*R. nivale* subsp. *boreale* samples were collected from the alpine region at an altitude of 4300 m, treated with liquid nitrogen, and sequenced (Fig. 1A). " (line 113)

> **Comment:** Line 119 - The total size of the assembly doesn't differ much between the different strategies - report the contig N50 in the main text which will tell you reader how well the assemblers performed. Looking at table S4 HiCanu might have performed better than Hifiasm. Even though I also prefer Hifiasm, why did you choose that over the longer and less contigs in hicanu? you say better integrity of genes and LTRs but you don't present the results for this or point to supplement here.

>> **Response:** Thank you for your comment. Our LAI assessment of assemblies using different approaches was based on the whole genome, including four haplotypes. Although such an assessment may be inaccurate due to the influence of identical sequences between subgenomes, we believe it still reflects the continuity of the assemblies. We have included these results in the Supplementary Table and described the datasets used. The LAI evaluations for the assemblies using different approaches are as follows: Hifiasm (LAI=12.19), HiCanu (LAI=9.27), Canu (LAI=11.31). (Table S4)

> **Comment:** Line 148 - the ratio of single exonic needs the word "genes".

>> **Response:** Done. "The ratio of mono-exonic (single-exon) genes to multi-exonic (multiple-exon) genes was 0.245." (line 149-150)

> **Comment:** line 215- "In" the dot plot...

>> **Response:** Done. "In the dot plot comparing *Vi. vinifera* and *R. nivale* subsp. *boreale* (Fig. 3D, S7–8) …" (line 216)

> **Comment:** Line 227 -These two genes? Which genes? I think something is missing here.

>> **Response:** We have revised this sentence. Apologies for the oversight, there are four

genes here, not two. The corrected text is as follows: "Four genes exhibited no significant positive selection sites. Finally, 16 genes met all model criteria and were identified as PSGs (Table. S19)". (line 228-230)

> **Comment:** line 287 - consistent with previous research - citation needed

>> **Response:** Done. "Consistent with previous research [33] …" (line 289)

> **Comment:** Line 447 - probably play important roles? I think "might" is more appropriate

>> **Response:** We have changed "probably" to "might". "Our positive selection analysis results suggested that *M3K1* and *CNGC1,* which are associated with the MAPK cascade and Ca^{2+} signal transduction, might play important roles in low-temperature adaptation." (line 448-450)

> **Comment:** line 468 - change "likely serves" to "may serve"

>> **Response:** We have replaced "likely serves" with "may serve". "Notably, *TOP3α*, an important gene related to the BTR complex, exhibits positive selection in *R. nivale* subsp. *boreale* and may serve as a key factor in promoting accurate chromosomal segregation during meiosis in autopolyploids." (line 469-471)

> **Comment:** line 470 - grammar. and too strong tone.

>> **Response:** We have checked the grammar and softened the tone. The corrected text is as follows: "Our results revealed that *R. nivale* subsp. *boreale* distributed on the mountaintop is an autotetraploid, which might be mediated by the harsh environment at high altitudes." (line 472-473)

> **Comment:** line 476 - what do you mean here by dominant gene expression pattern? clarify.

>> **Response:** Thank you for your comment. We have clarified this sentence. The revised version is as follows: "Polyploidization likely plays an important role in

mountaintop survival because increased gene dosage can lead to enhanced stress tolerance, greater genetic diversity, and the potential for novel traits that enhance adaptability [18]." (line 478-481)

Reviewer #3:

Thank you for reviewing our manuscript. We have made improvements to the manuscript in response to your comments. The point-by-point response follows:

> **Comment:** Abstract: "As a high-altitude woody polyploid, this species plays a distinct role in the adaptability of alpine plants"
- This plant does not affect the adaptability of other plants, it can tell us something about how plants adapt to alpine environments however. I'm sure thats what the authors intended. Suggested revision:
"As a high-altitude woody polyploid, this species may serve as a model to understand how plants adapt to alpine environments"
>> **Response:** Thank you for your suggestion. We have made the modification. (line 24-25)

> **Comment:** Line 118: "k-mers" should just be "k-mer"
>> **Response:** Done. "This species was identified as a tetraploid based on *k-mer* analysis (Fig. 1E)." (line 119)
