# Supplementary material for: The first high-altitude autotetraploid haplotype-resolved genome assembled (Rhododendron nivale subsp. boreale) provides new insights into mountaintop adaptation
Source: Gigascience. 2024 Aug 7;13:giae052. doi: 10.1093/gigascience/giae052 (PMC11304948; doi:10.1093/gigascience/giae052)
Supplement: giae052_Supplemental_Files [file giae052_supplemental_files.zip › Supplementary Figure.docx]

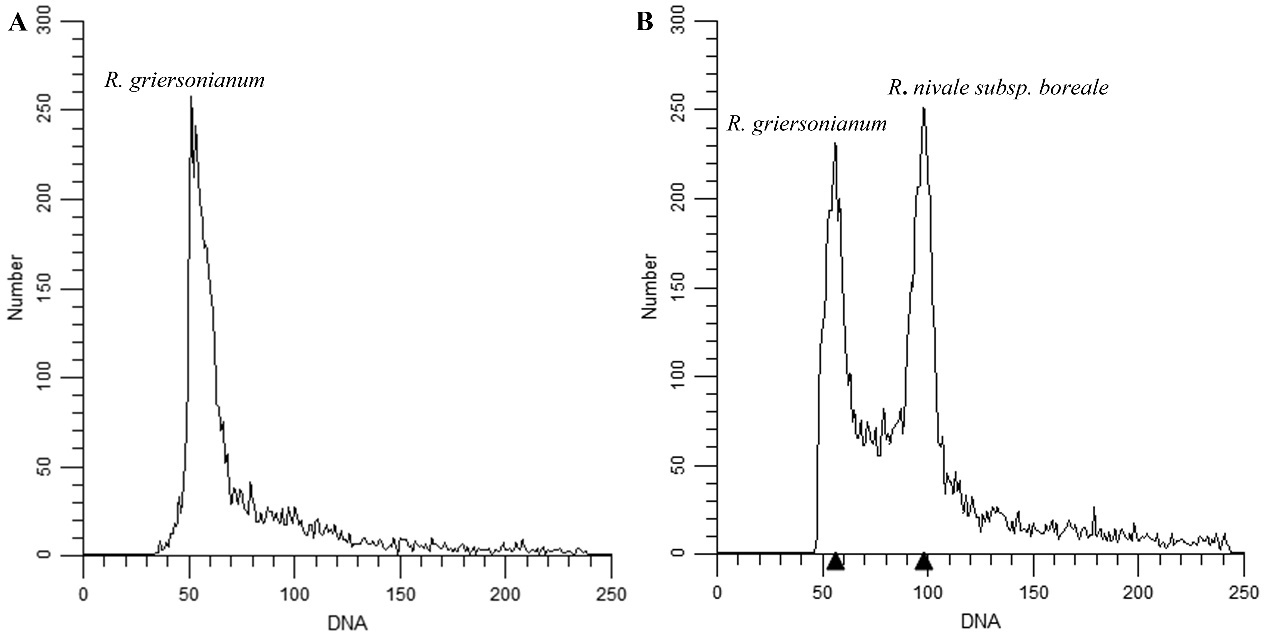


Fig. S1 Estimate of the *Rhododendron nivale* subsp. *boreale* genome via flow cytometry. A. flow cytometry of *R. griersonianum*; B. flow cytometry of *R. nivale* subp. *boreale* and *R. griersonianum*.


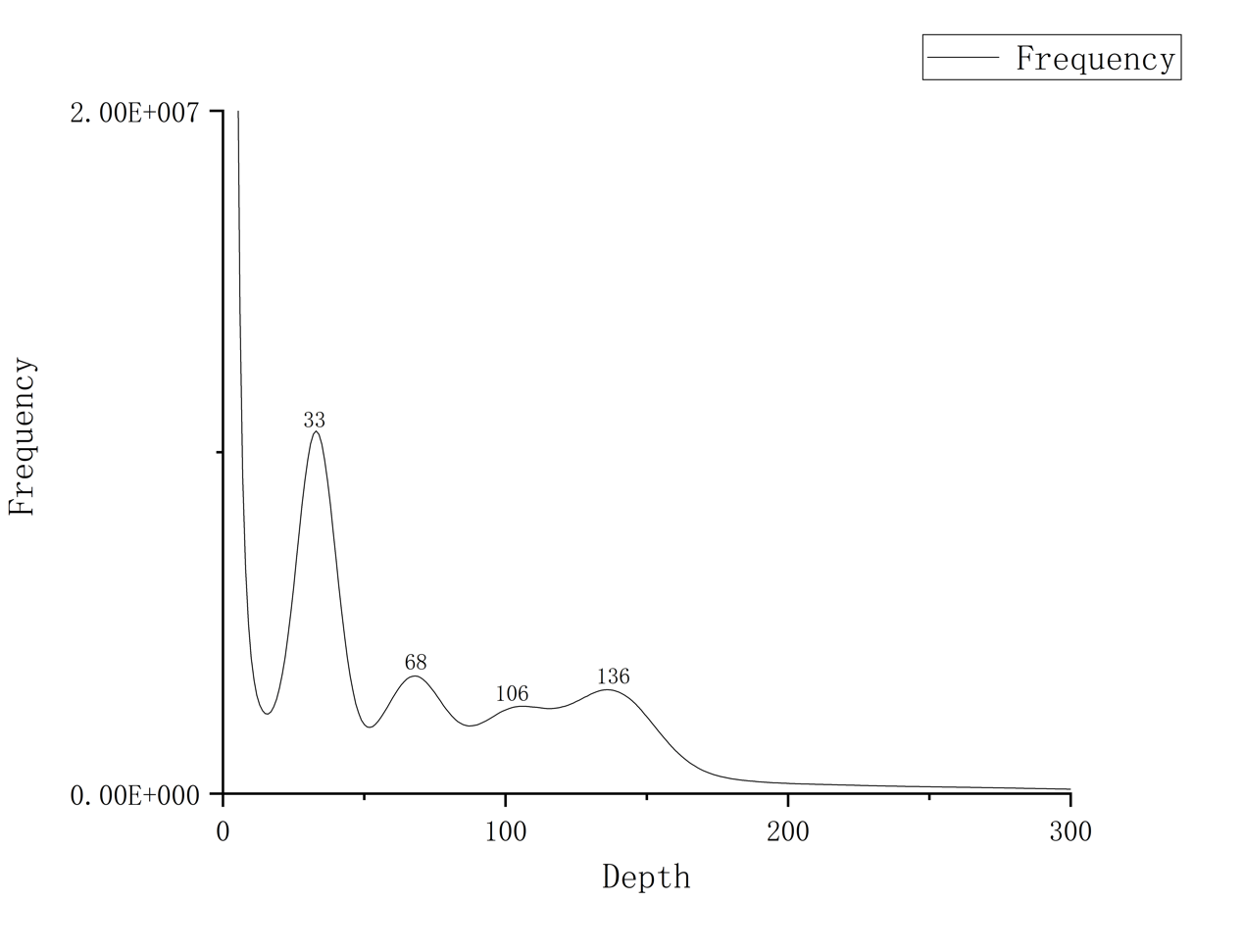


Fig. S2 *K-mer* distribution of the *R. nivale* subsp. *boreale* genome based on DNBseq data.


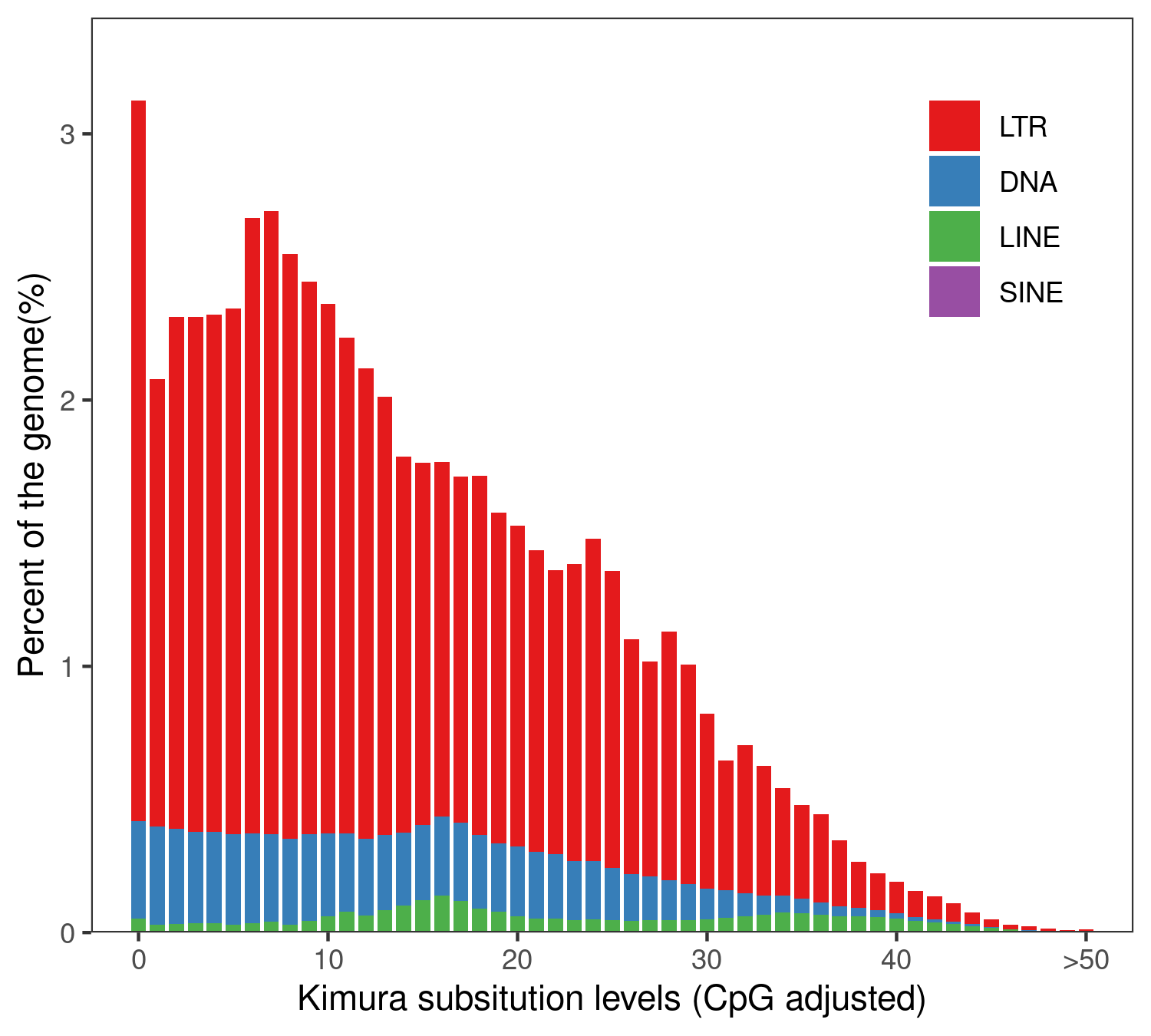


Fig. S3 The frequency of the four types of repetitive sequences in genome of *R. nivale* subsp. *boreale.*


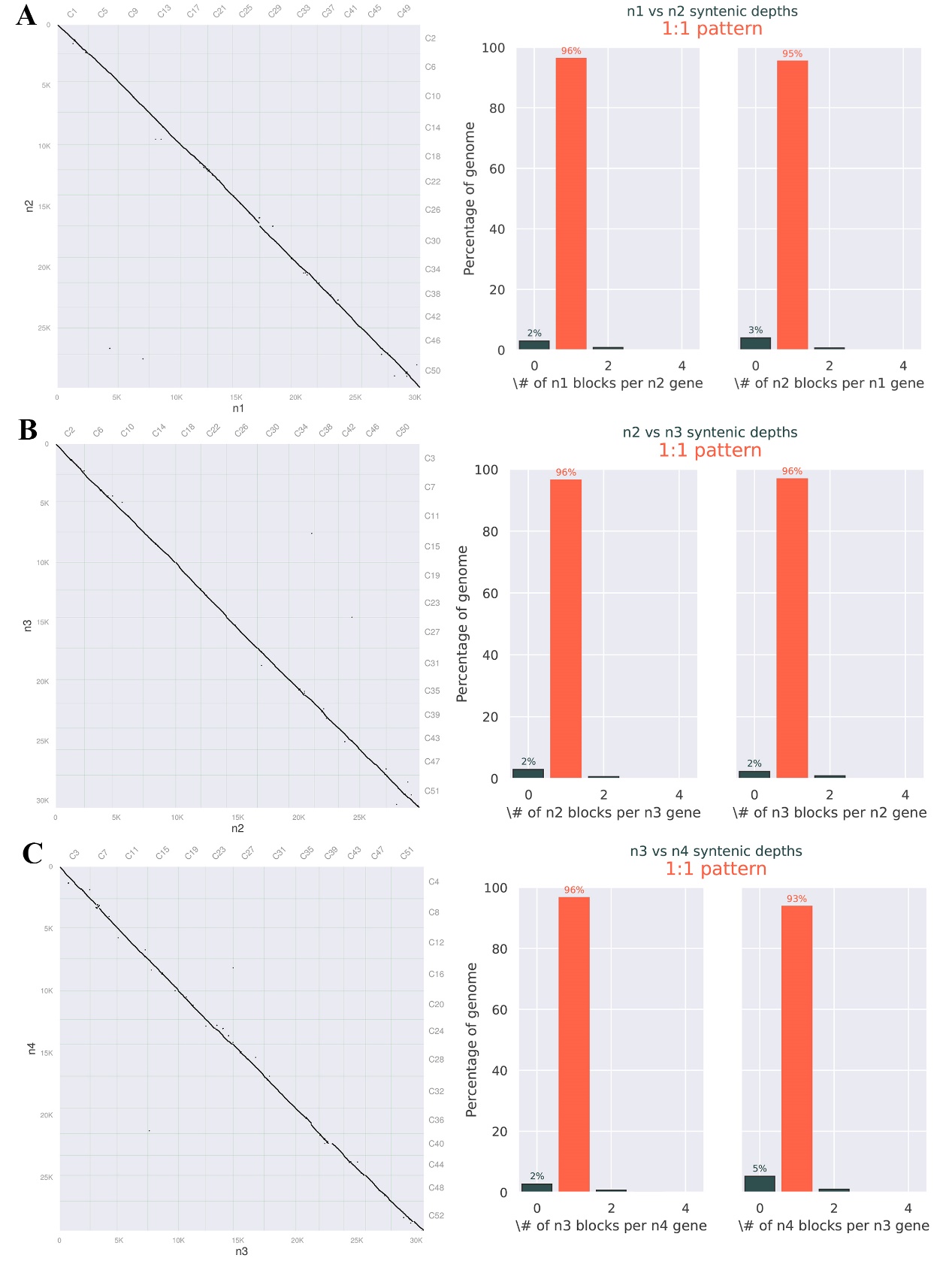


Fig. S4 Dot analysis between four haplotypes of *R. nivale* subsp. *boreale* genome. A. Dot analysis and syntenic depths between haplotype 1 (n1) and haplotype 2 (n2); B. Dot analysis and syntenic depths between haplotype 2 (n2) and haplotype 3 (n3); C. Dot analysis and syntenic depths between haplotype 1 (n3) and haplotype 2 (n4).


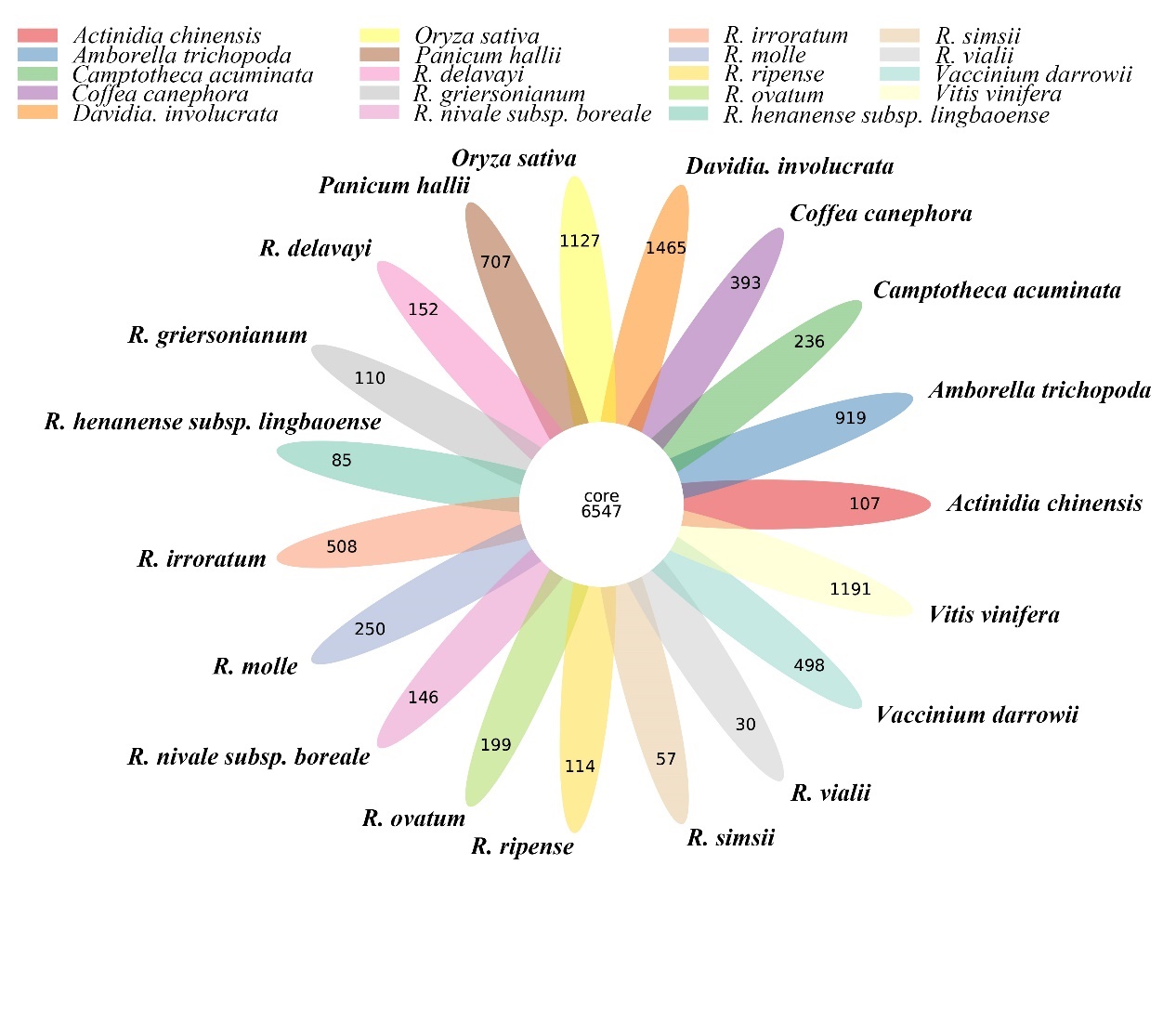


Fig. S5 flower plot indicated that the orthologous gene families of 19 species.


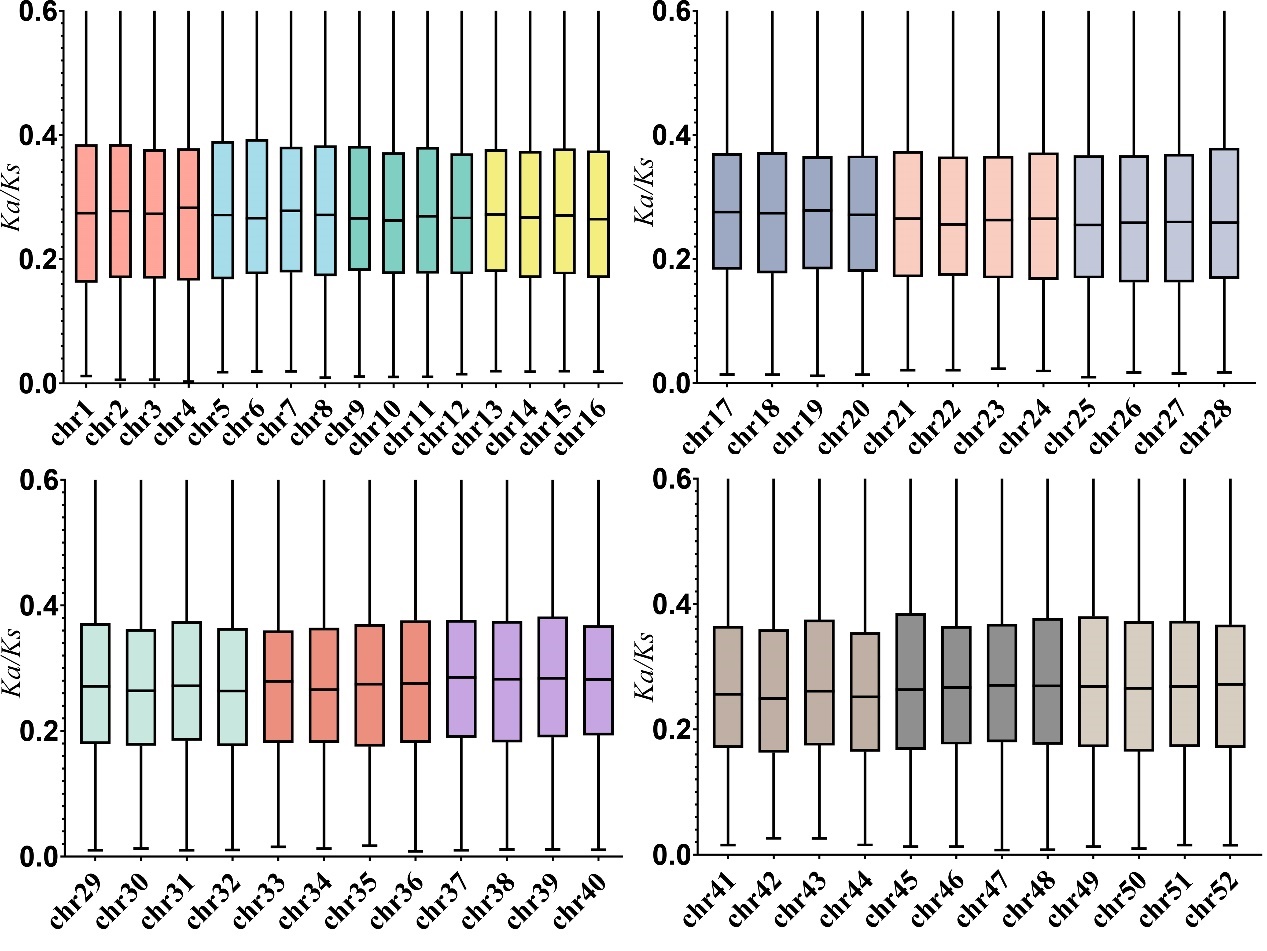


Fig. S6 *K*a/*K*s values of single match alleles in 52 pseudochromosomes of *R. nivale* subsp. *boreale*. Homologous groups are distinguished by different colors.

Fig. S7 Collinearity analysis between *R. nivale* subsp. *boreale* and Vitis vinifera. Green lines show the gene that have been doubled.


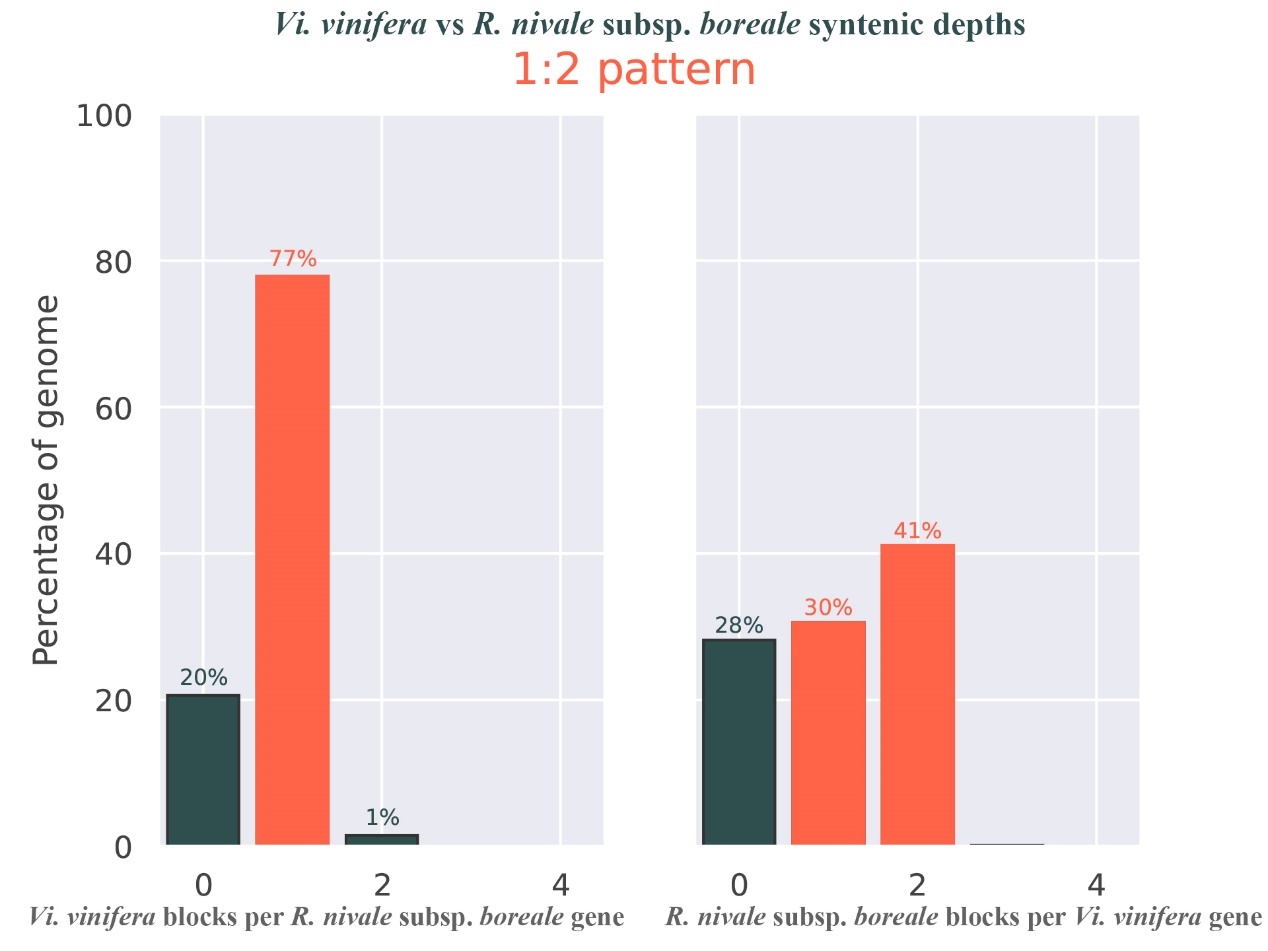


Fig. S8 The syntenic depth analysis between *R. nivale* subsp. *boreale* and *Vitis vinifera*.


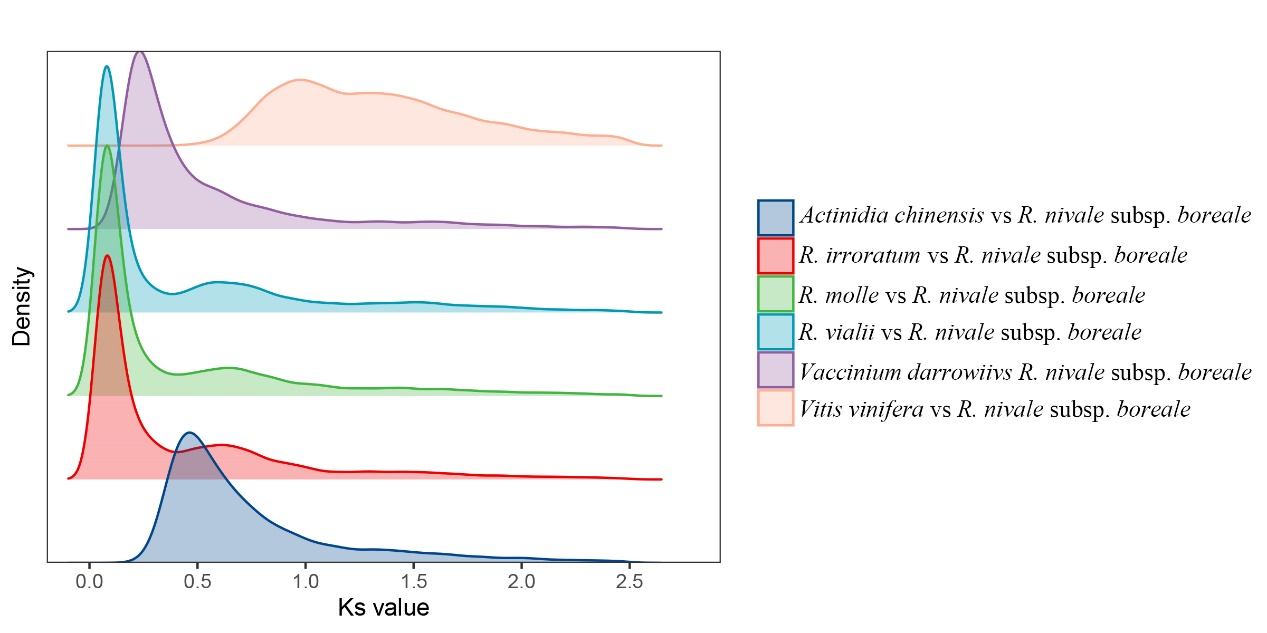


Fig. S9 *K*s of ortholog frequency distribution chart between *R. nivale* subsp. *boreale* and other six species.


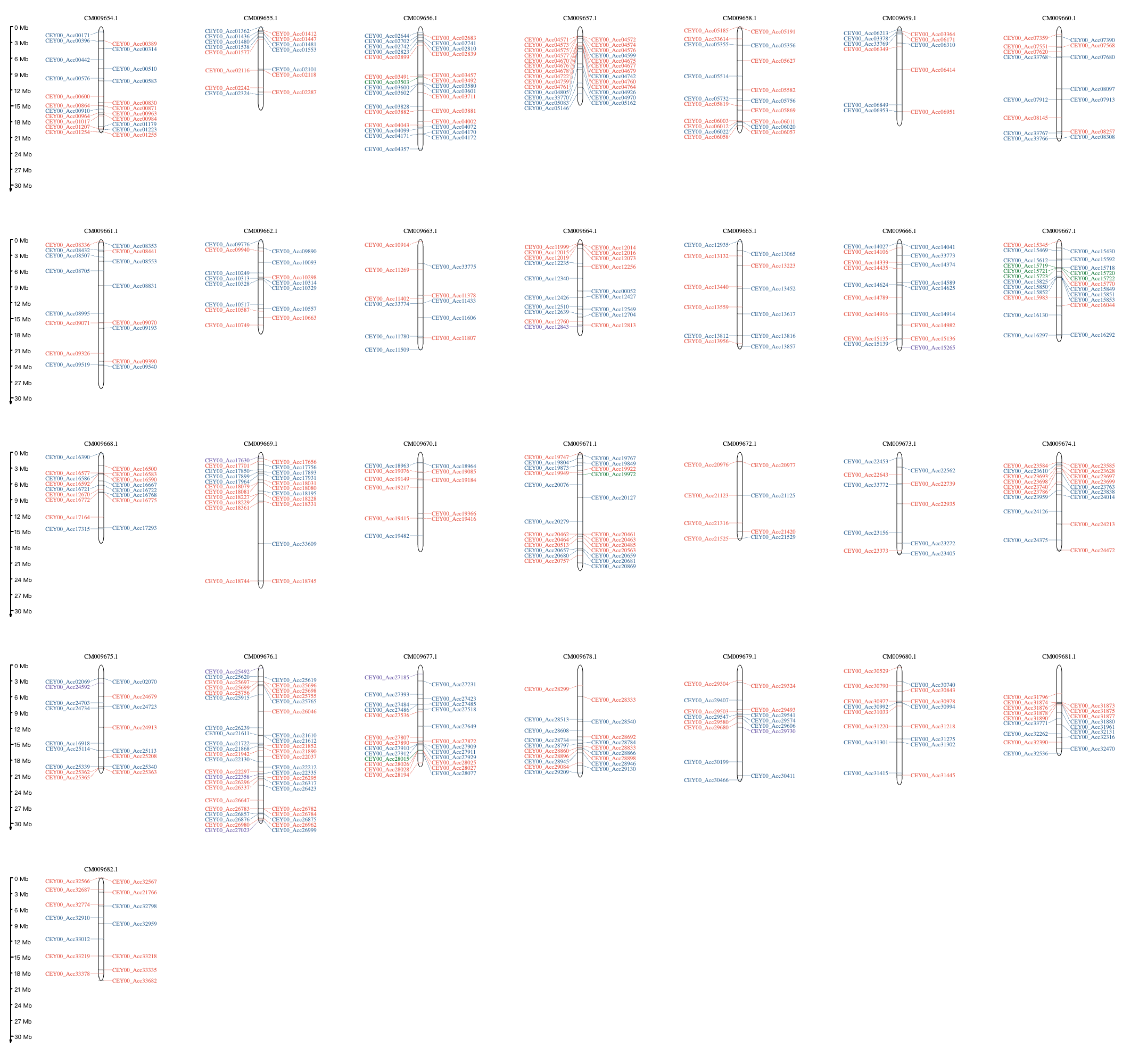


Fig. S10 Chromosomal distribution map of the AP2/ERF and CYP genes in *Ac. chinensis*. The chromosome ID is indicated at the top of each chromosome. The blue lines indicate positions of AP2/ERF gene, red lines indicate positions of CYP gene, purple lines indicate positions of *ERF VII* gene, green lines indicate positions of *CBF* gene.


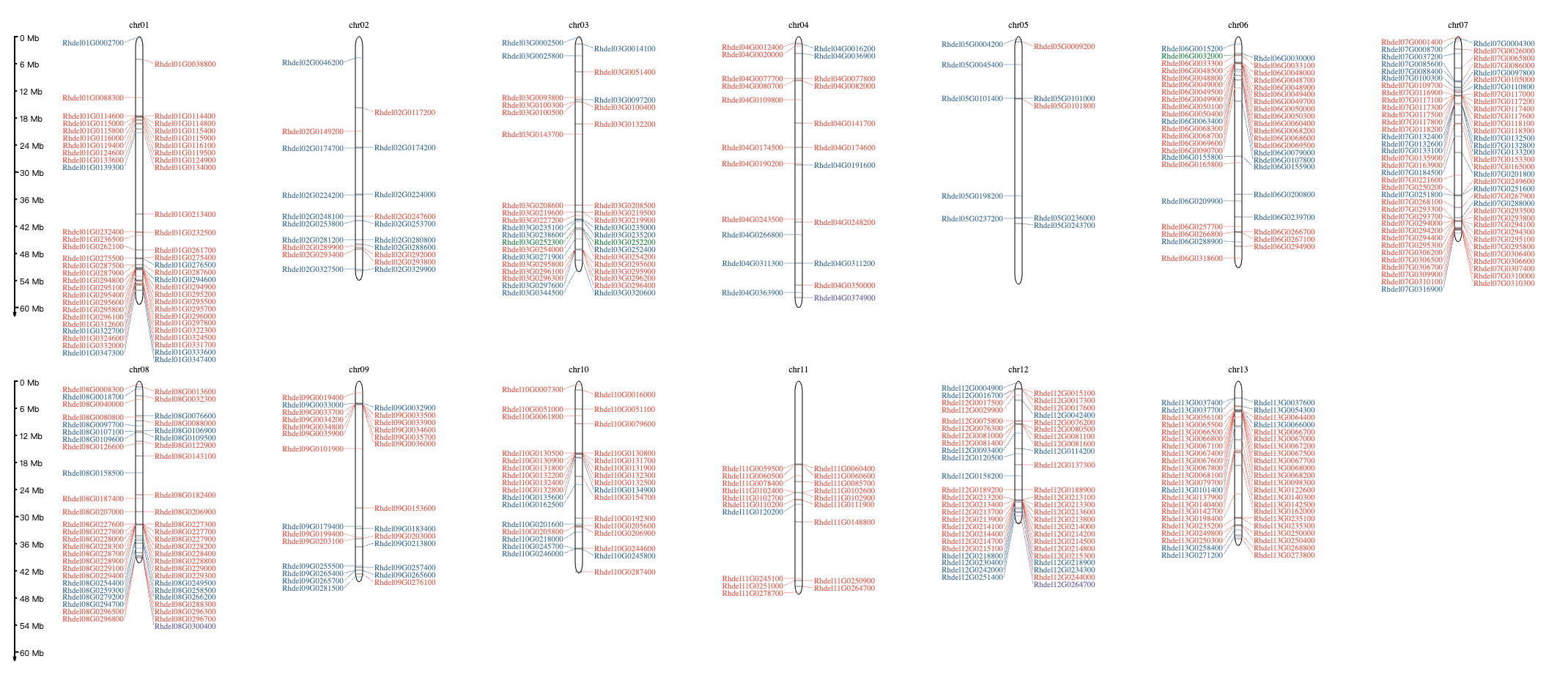


Fig. S11 Chromosomal distribution map of the AP2/ERF and CYP genes in *R. delavayi*. The chromosome ID is indicated at the top of each chromosome. The blue lines indicate positions of AP2/ERF gene, red lines indicate positions of CYP gene, purple lines indicate positions of *ERF VII* gene, green lines indicate positions of *CBF* gene.


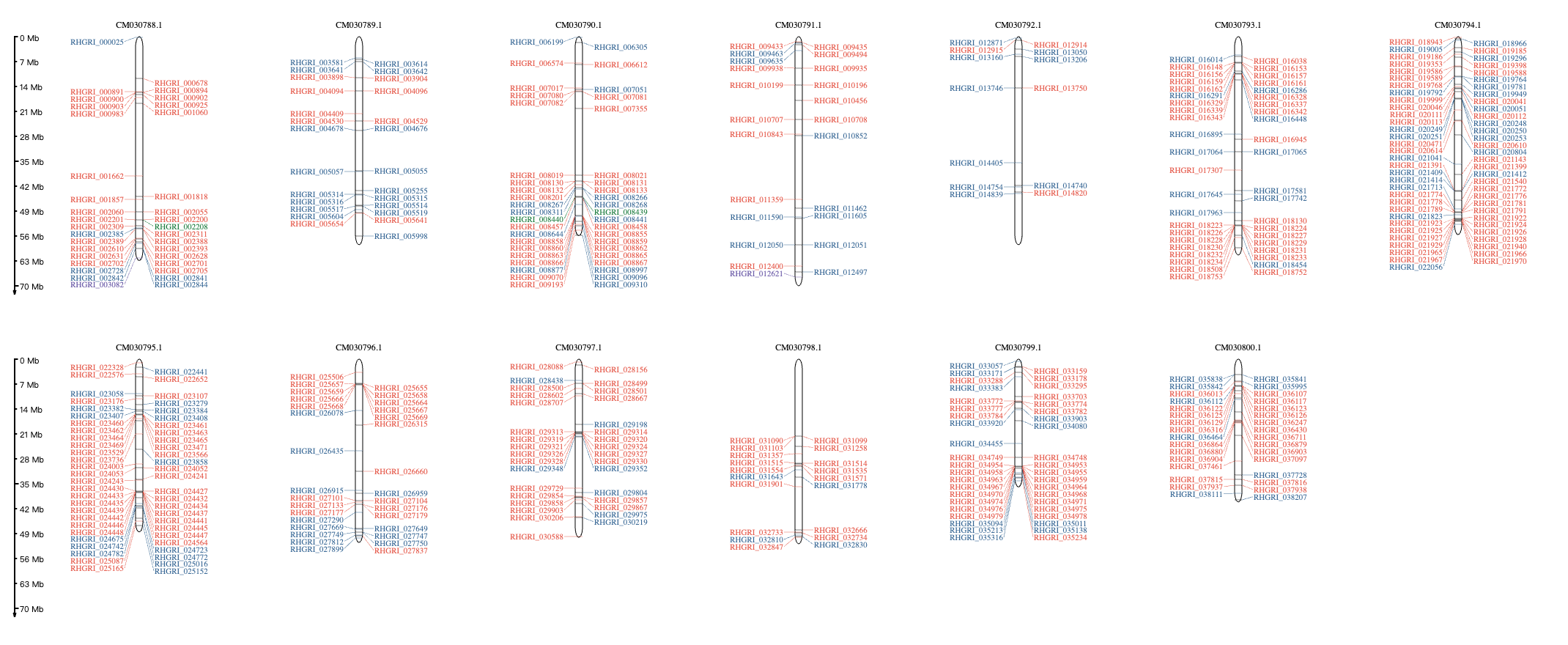


Fig. S12 Chromosomal distribution map of the AP2/ERF and CYP genes in *R. griersonianum*. The chromosome ID is indicated at the top of each chromosome. The blue lines indicate positions of AP2/ERF gene, red lines indicate positions of CYP gene, purple lines indicate positions of *ERF VII* gene, green lines indicate positions of *CBF* gene.


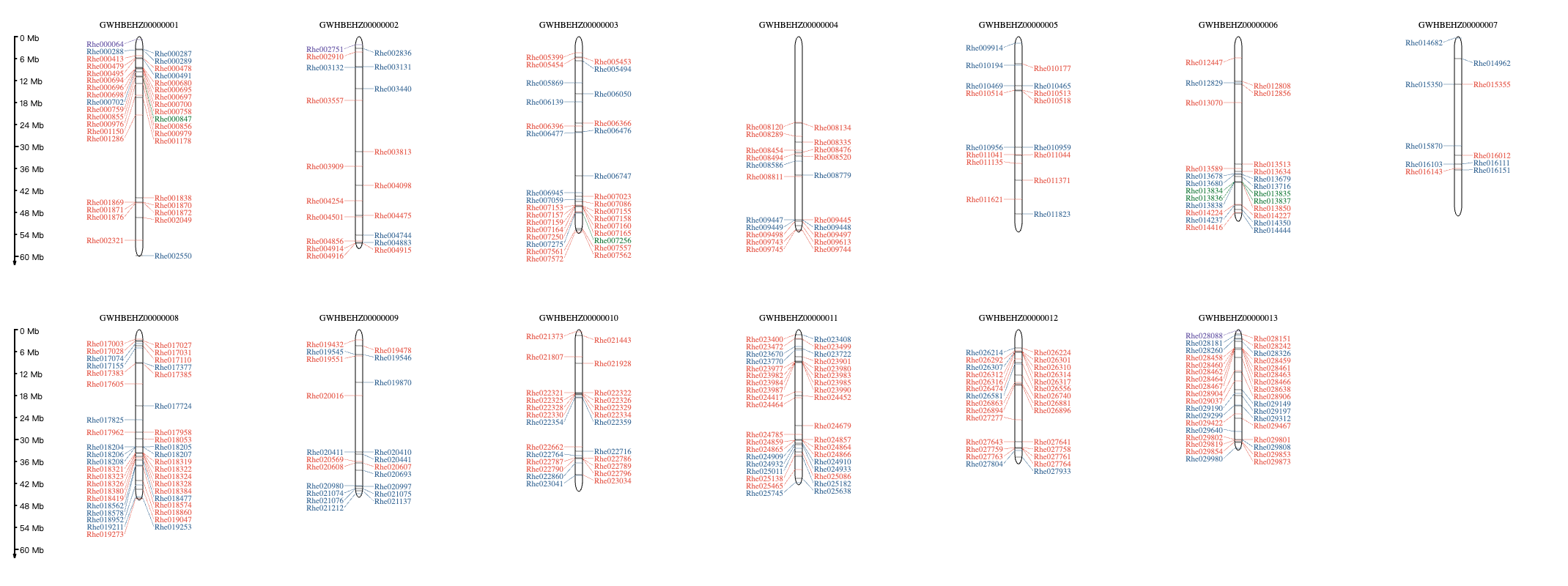


Fig. S13 Chromosomal distribution map of the AP2/ERF and CYP genes in *R. henanense subsp. lingbaoense*. The chromosome ID is indicated at the top of each chromosome. The blue lines indicate positions of AP2/ERF gene, red lines indicate positions of CYP gene, purple lines indicate positions of *ERF VII* gene, green lines indicate positions of *CBF* gene.


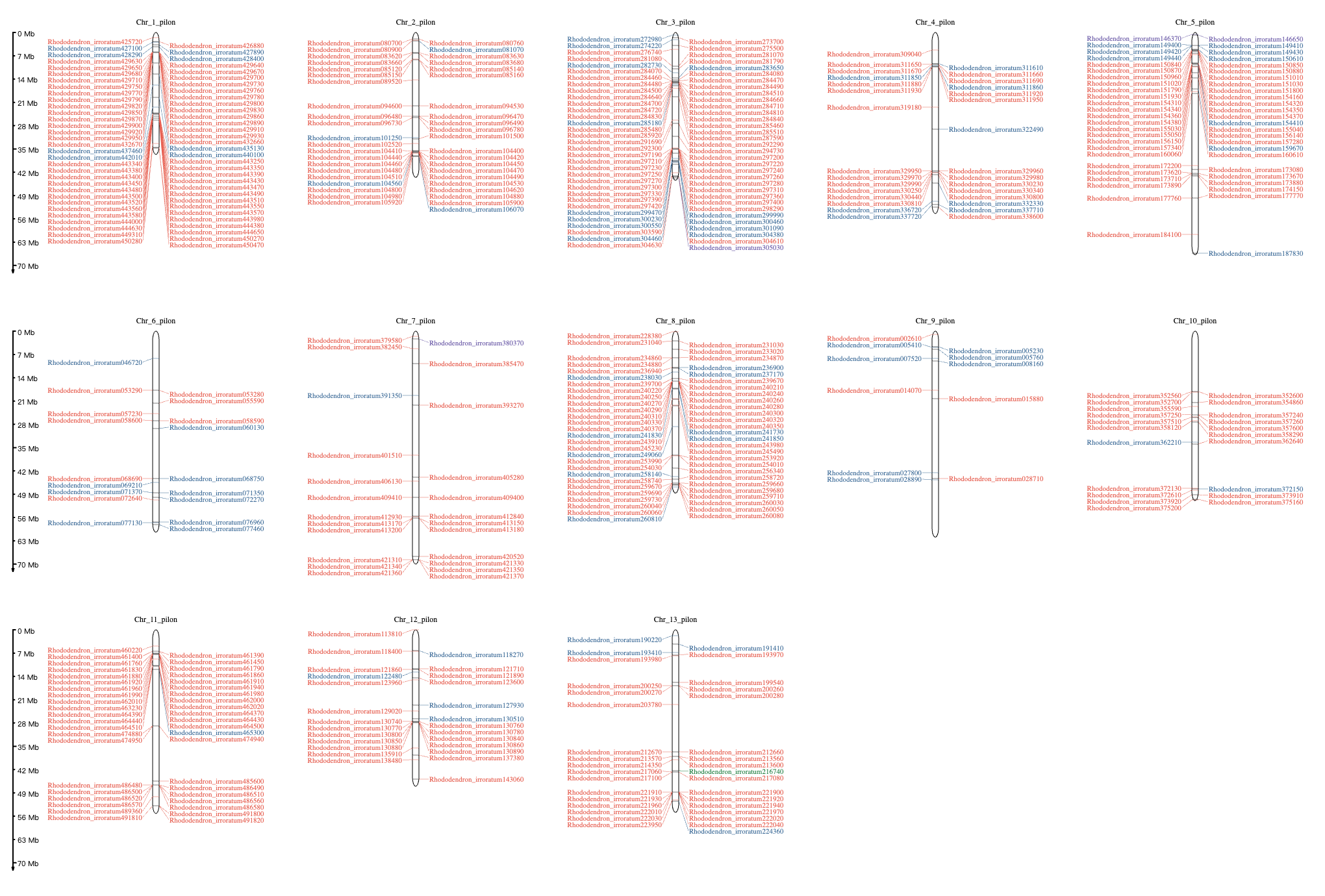


Fig. S14 Chromosomal distribution map of the AP2/ERF and CYP genes in *R. irroratum*. The chromosome ID is indicated at the top of each chromosome. The blue lines indicate positions of AP2/ERF gene, red lines indicate positions of CYP gene, purple lines indicate positions of *ERF VII* gene, green lines indicate positions of *CBF* gene.


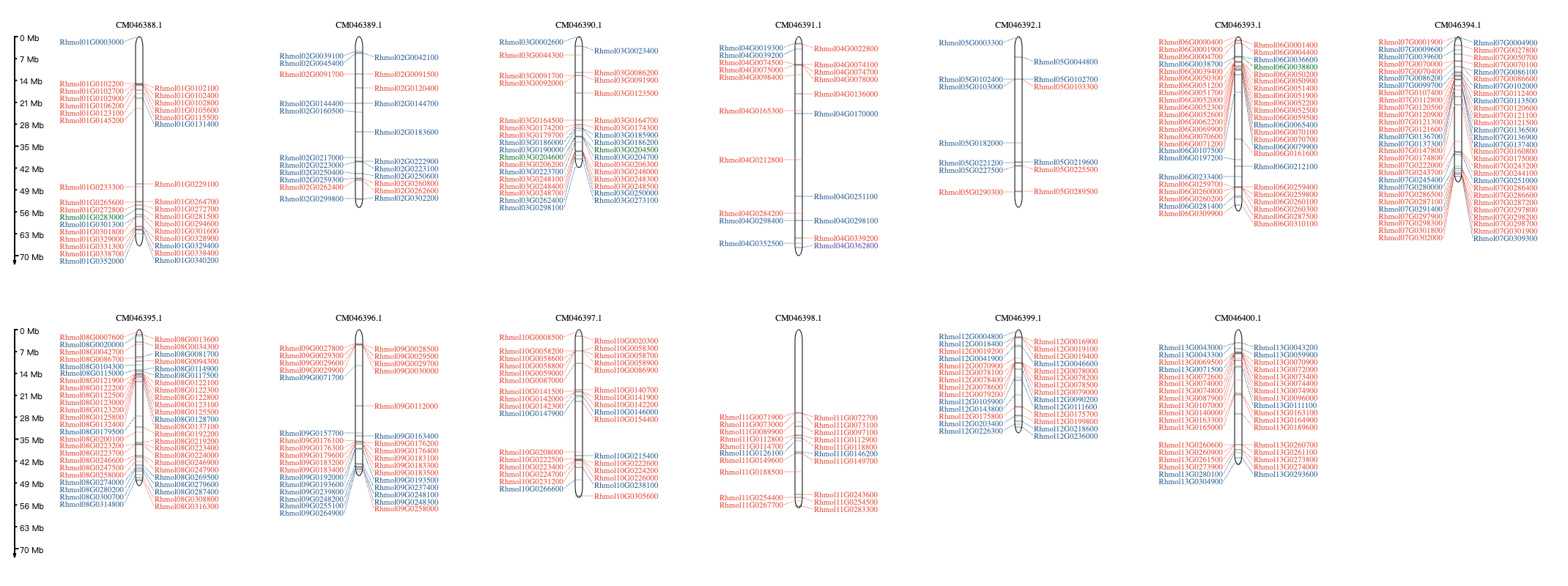


Fig. S15 Chromosomal distribution map of the AP2/ERF and CYP genes in *R. molle*. The chromosome ID is indicated at the top of each chromosome. The blue lines indicate positions of AP2/ERF gene, red lines indicate positions of CYP gene, purple lines indicate positions of *ERF VII* gene, green lines indicate positions of *CBF* gene.


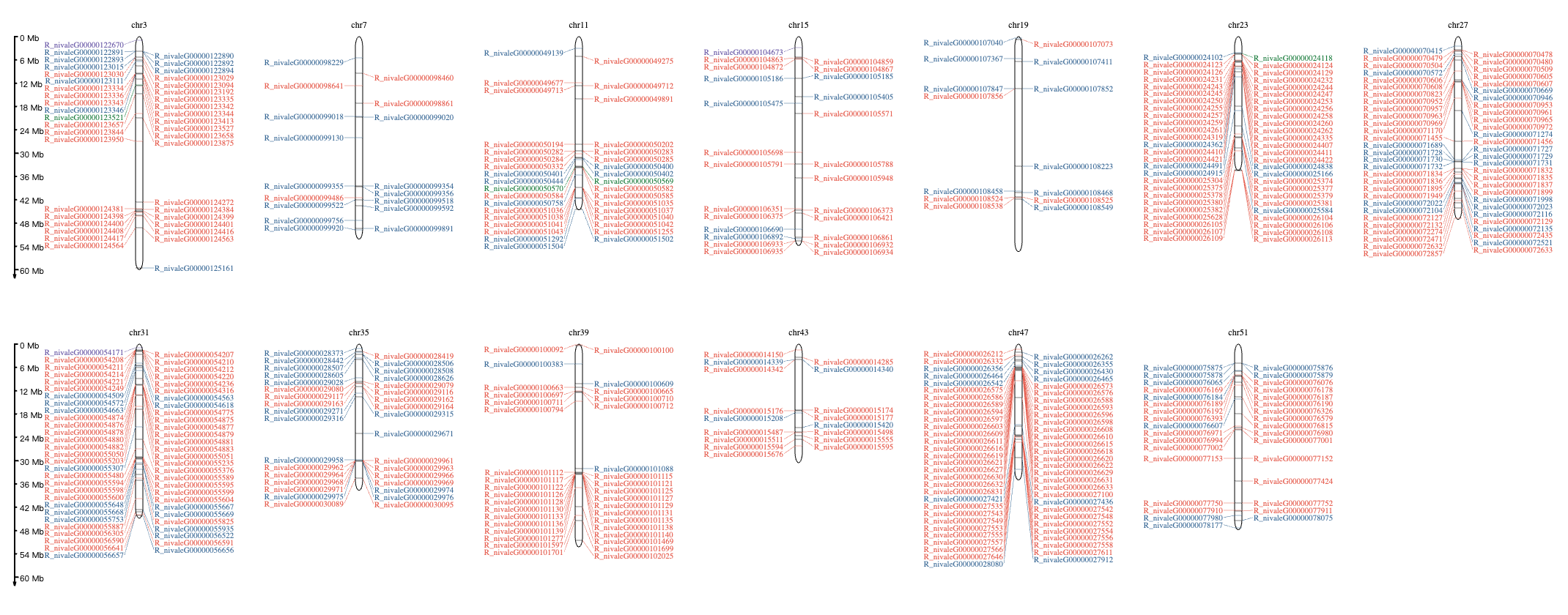


Fig. S16 Chromosomal distribution map of the AP2/ERF and CYP genes in *R. nivale subsp. boreale*. The chromosome ID is indicated at the top of each chromosome. The blue lines indicate positions of AP2/ERF gene, red lines indicate positions of CYP gene, purple lines indicate positions of *ERF VII* gene, green lines indicate positions of *CBF* gene.


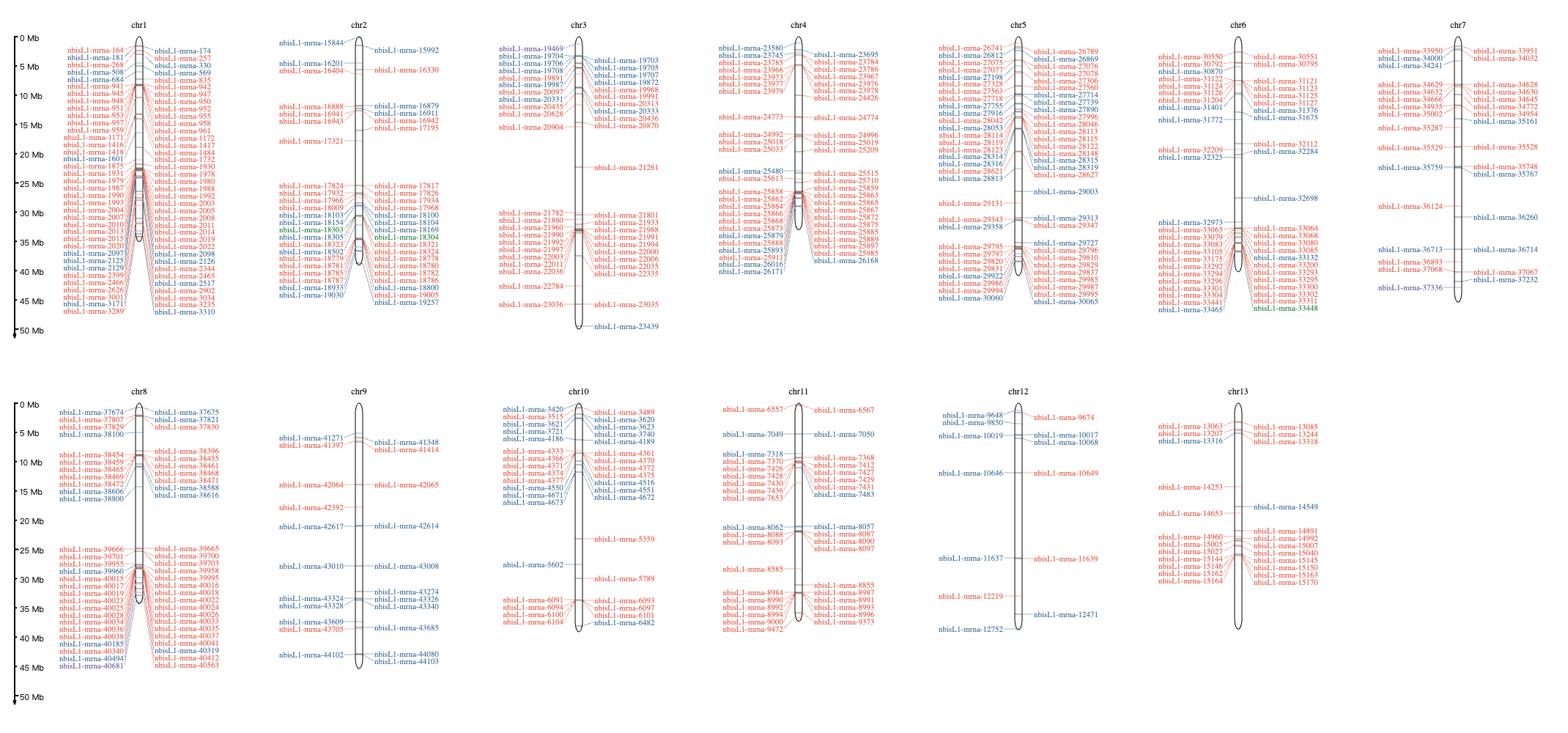


Fig. S17 Chromosomal distribution map of the AP2/ERF and CYP genes in *R. ovatum*. The chromosome ID is indicated at the top of each chromosome. The blue lines indicate positions of AP2/ERF gene, red lines indicate positions of CYP gene, purple lines indicate positions of *ERF VII* gene, green lines indicate positions of *CBF* gene.


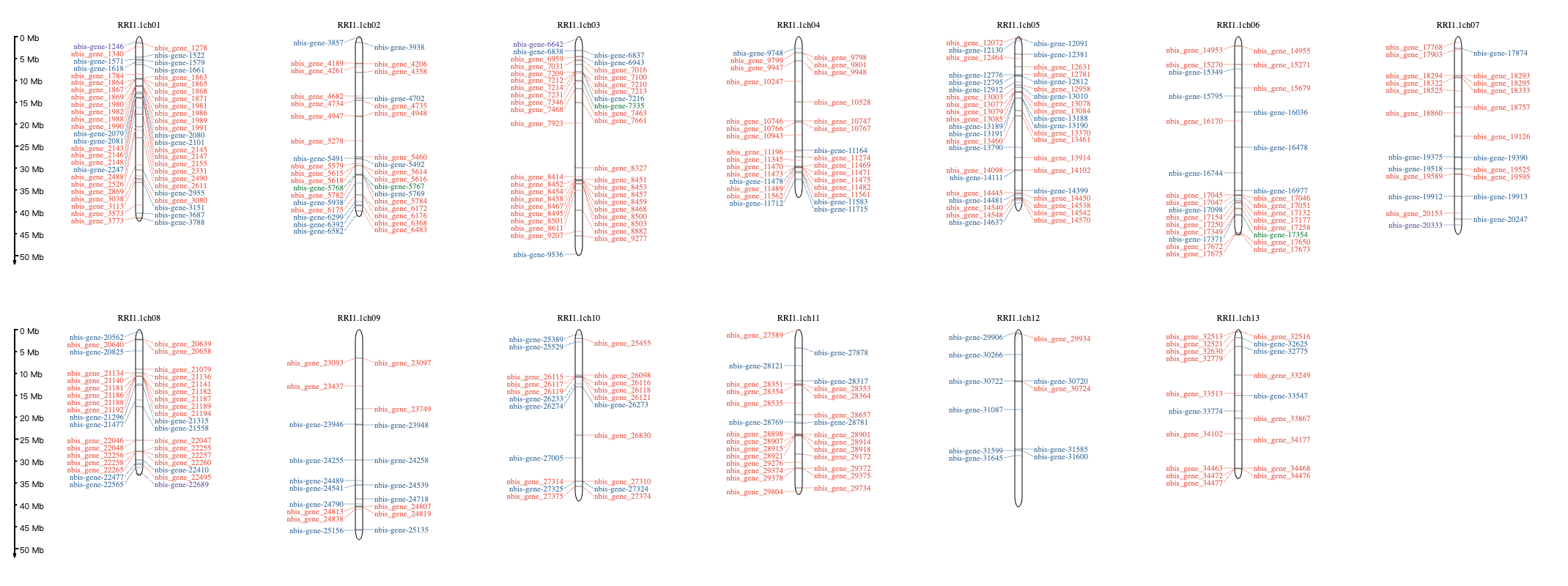


Fig. S18 Chromosomal distribution map of the AP2/ERF and CYP genes in *R. ripense*. The chromosome ID is indicated at the top of each chromosome. The blue lines indicate positions of AP2/ERF gene, red lines indicate positions of CYP gene, purple lines indicate positions of *ERF VII* gene, green lines indicate positions of *CBF* gene.


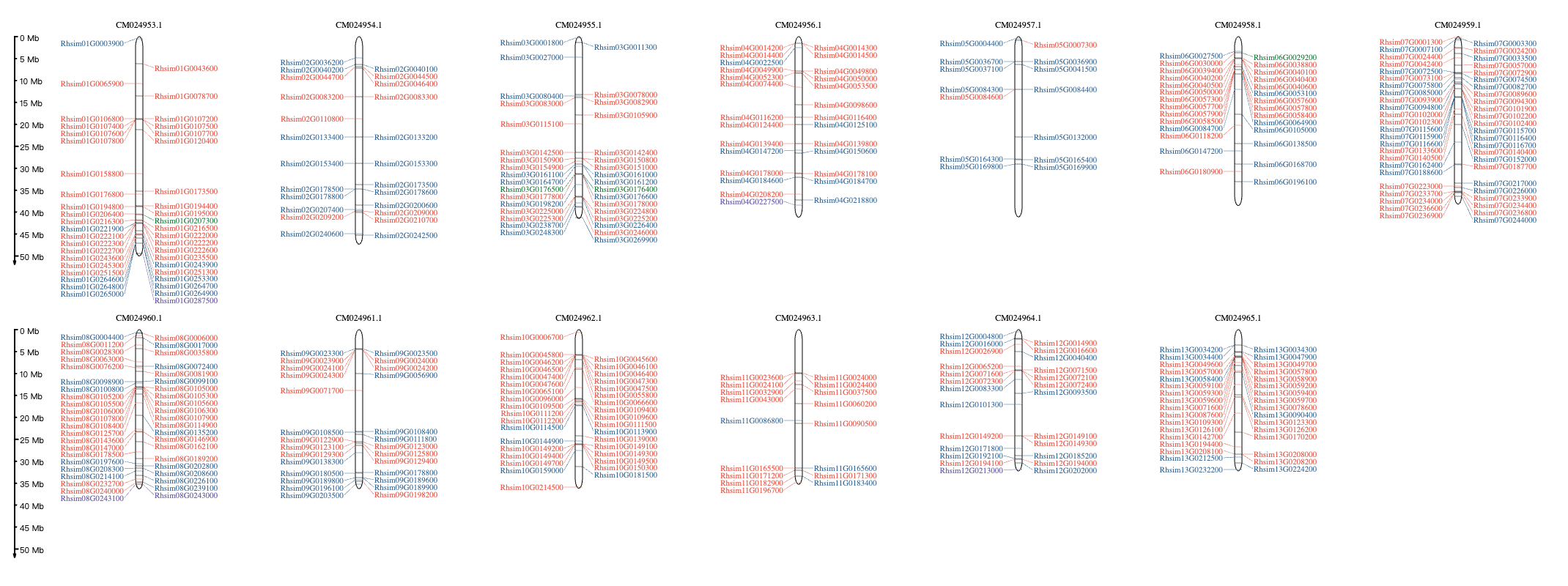


Fig. S19 Chromosomal distribution map of the AP2/ERF and CYP genes in *R. simsii*. The chromosome ID is indicated at the top of each chromosome. The blue lines indicate positions of AP2/ERF gene, red lines indicate positions of CYP gene, purple lines indicate positions of *ERF VII* gene, green lines indicate positions of *CBF* gene.


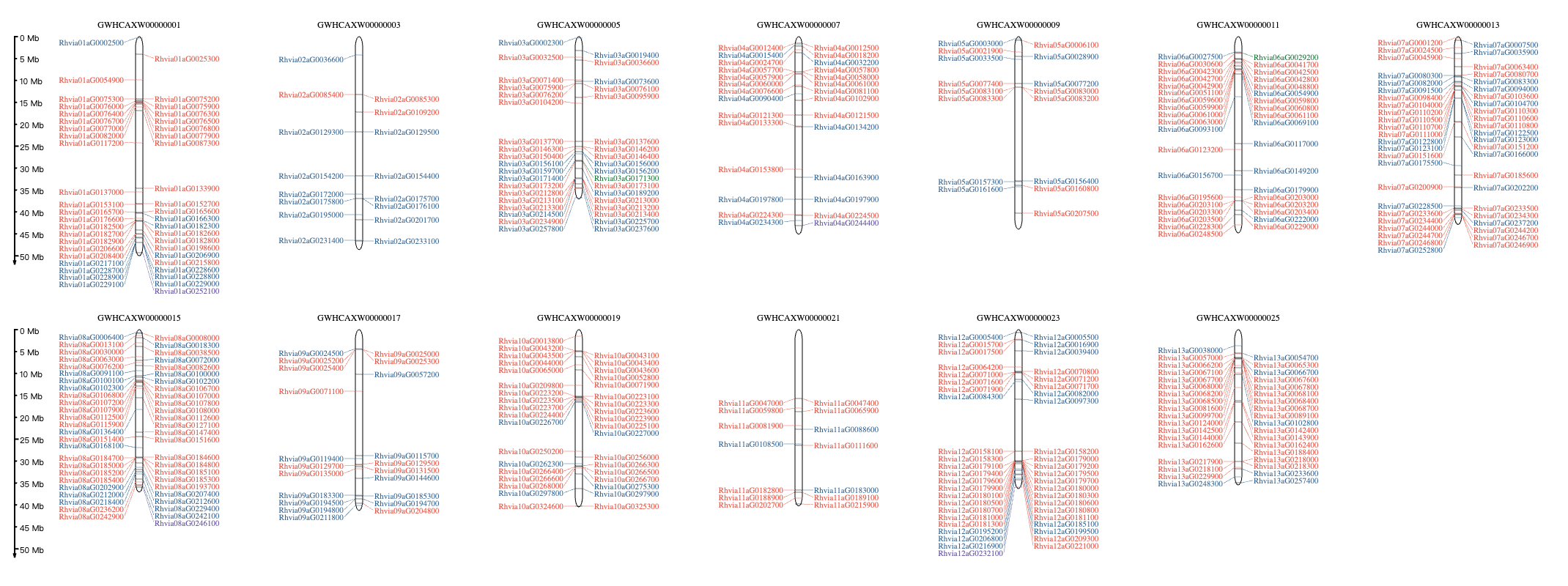


Fig. S20 Chromosomal distribution map of the AP2/ERF and CYP genes in *R. vialii*. The chromosome ID is indicated at the top of each chromosome. The blue lines indicate positions of AP2/ERF gene, red lines indicate positions of CYP gene, purple lines indicate positions of *ERF VII* gene, green lines indicate positions of *CBF* gene.


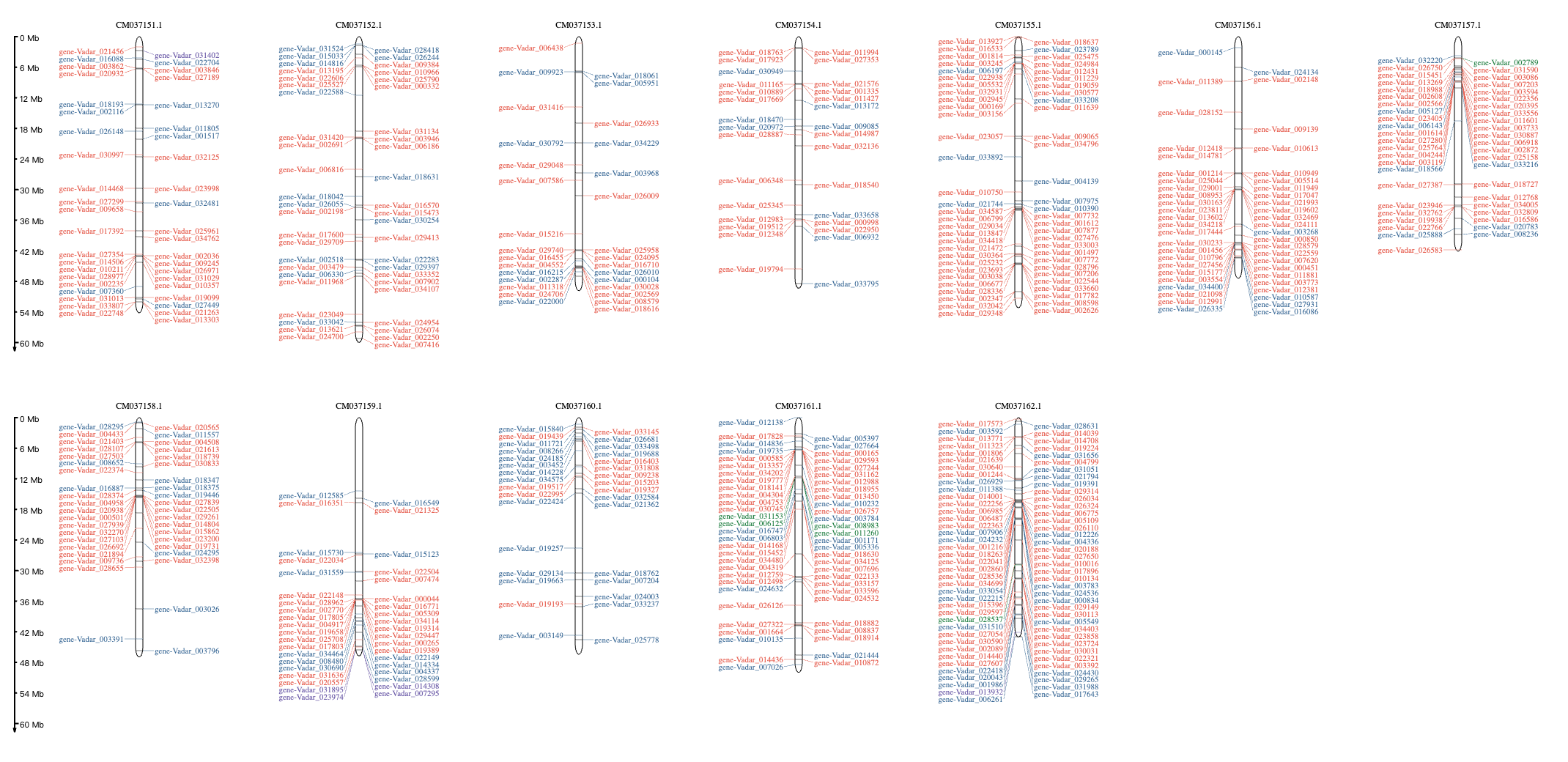


Fig. S21 Chromosomal distribution map of the AP2/ERF and CYP genes in *Va. darrowii*. The chromosome ID is indicated at the top of each chromosome. The blue lines indicate positions of AP2/ERF gene, red lines indicate positions of CYP gene, purple lines indicate positions of *ERF VII* gene, green lines indicate positions of *CBF* gene.


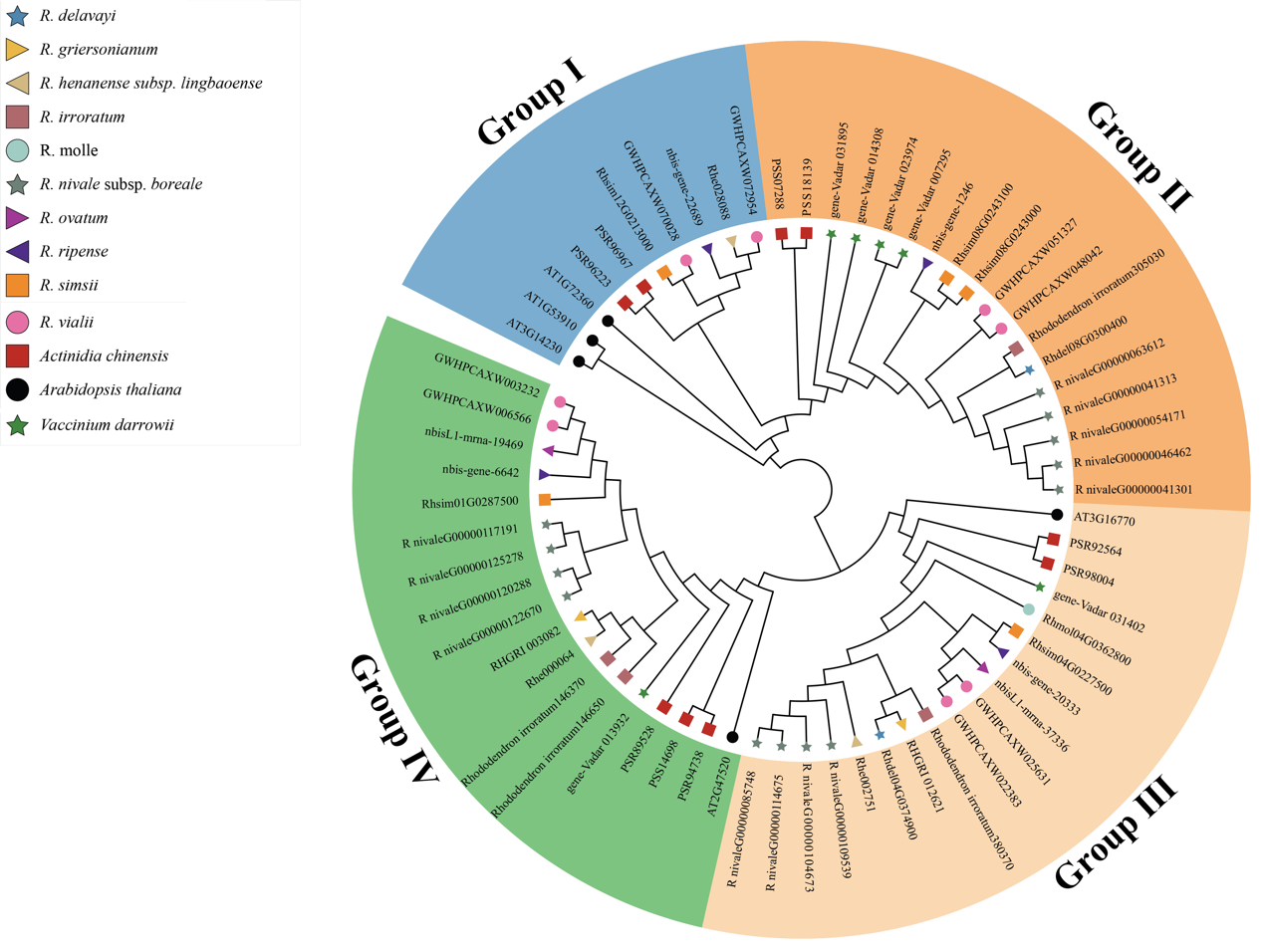


Fig. S22 Phylogenetic analysis of the ERF VII proteins in the 13 species. Group I to Iv were distinguished by four colors.


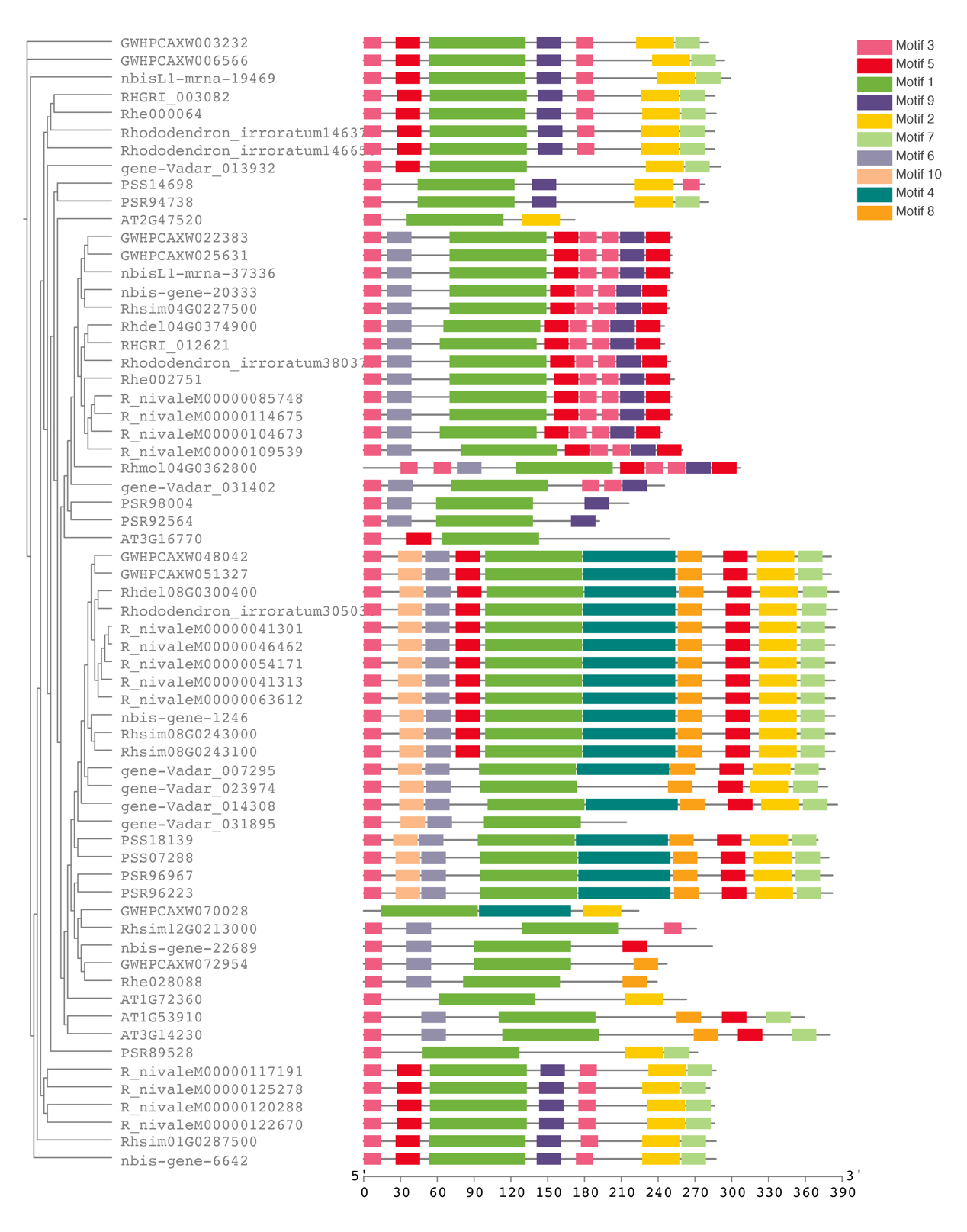


Fig. S23 Conserved motifs analysis of ERF VII. A. The phylogenetic tree of ERF VII proteins; B. Conserved motifs of the ERF VII. Different motifs were distinguished by color.


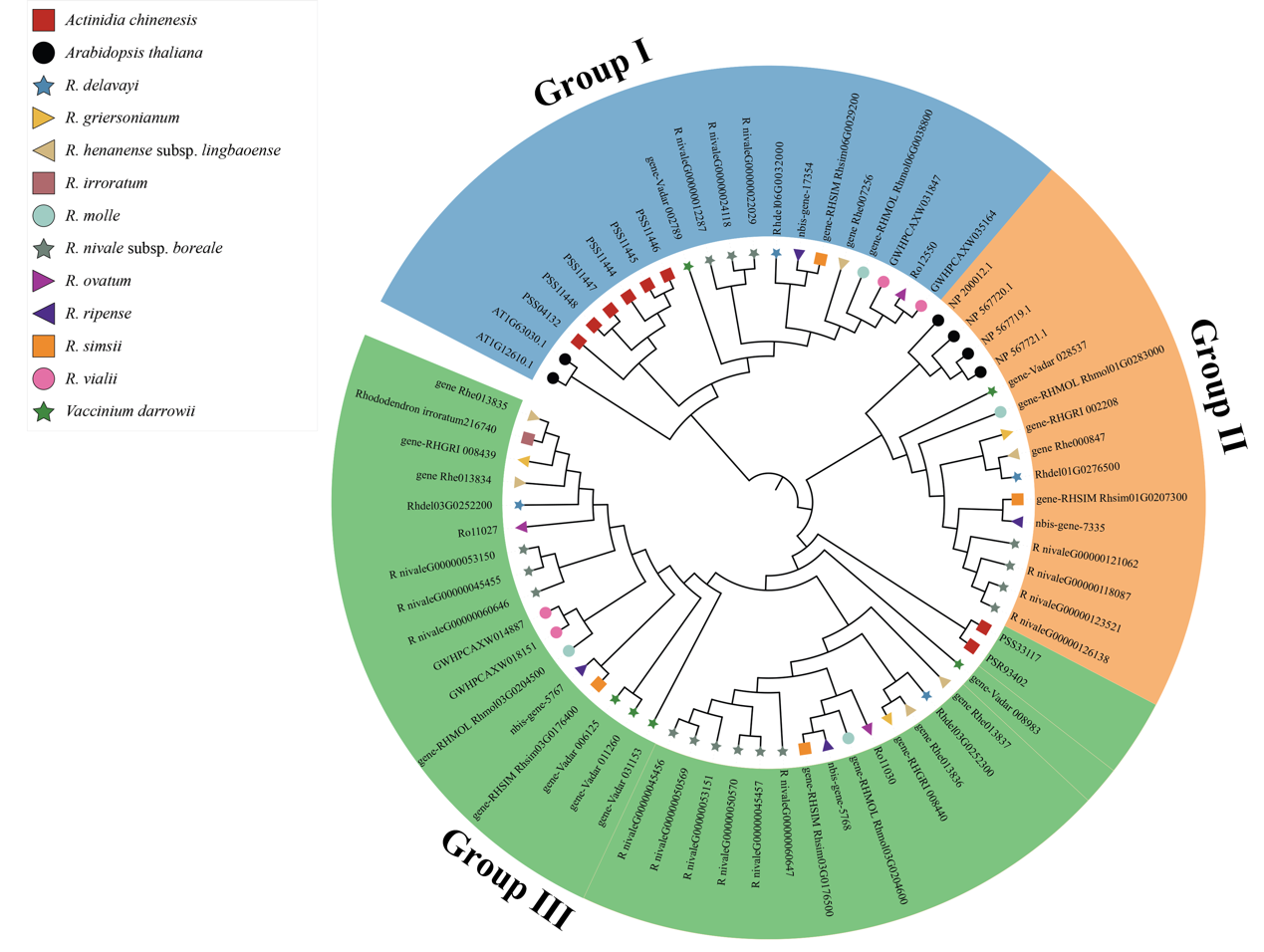


Fig. S24 Phylogenetic analysis of the CBF proteins in the 13 species. Group I to III were distinguished by four colors.


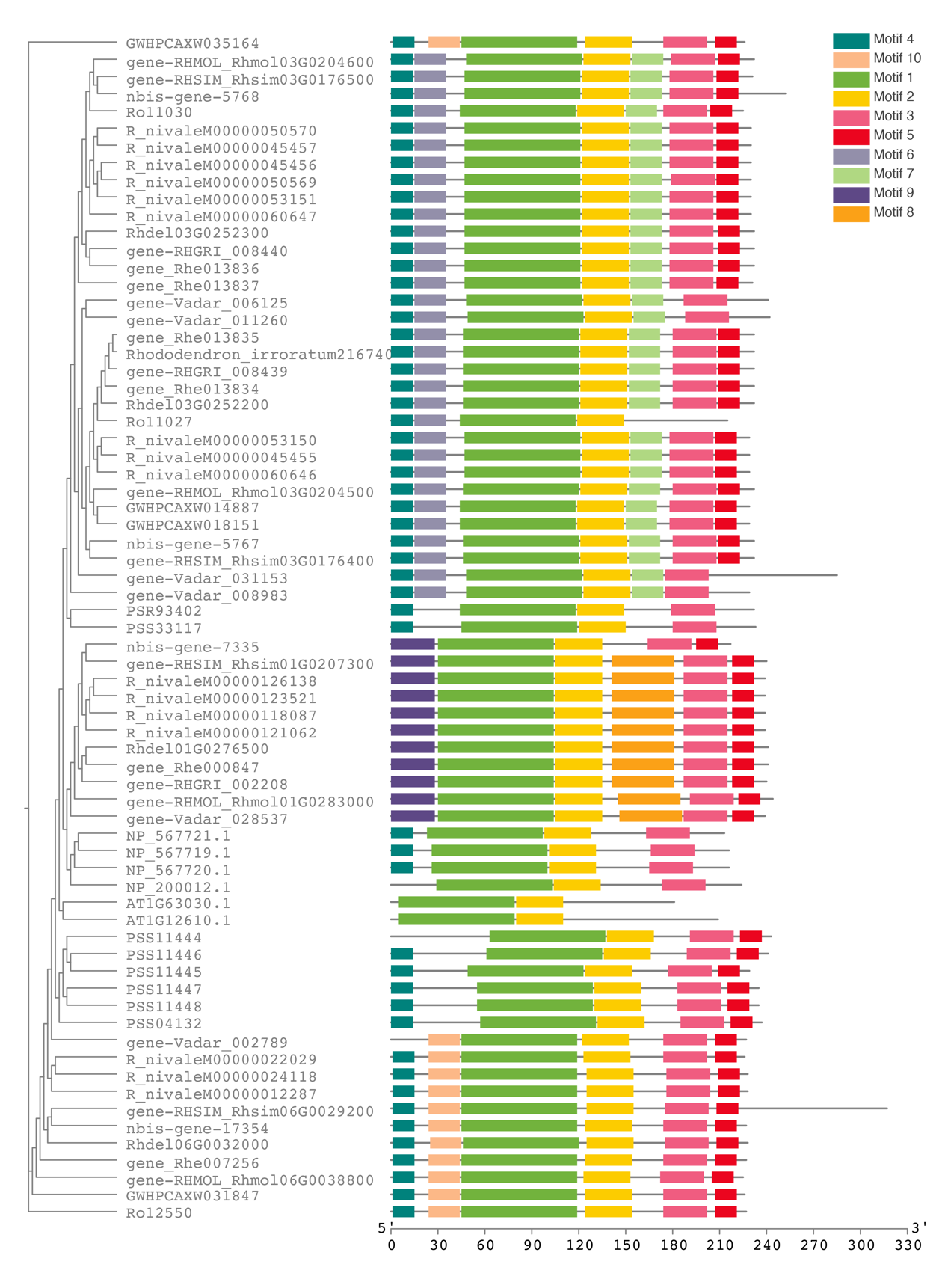


Fig. S25 Conserved motifs analysis of ERF VII. A. The phylogenetic tree of ERF VII proteins; B. Conserved motifs of the ERF VII. Different motifs were distinguished by color.
