# Supplementary material for: The first high-altitude autotetraploid haplotype-resolved genome assembled (Rhododendron nivale subsp. boreale) provides new insights into mountaintop adaptation
Source: Gigascience. 2024 Aug 7;13:giae052. doi: 10.1093/gigascience/giae052 (PMC11304948; doi:10.1093/gigascience/giae052)
Supplement: giae052_Response_to_Reviewer_Comments_Original_Submission [file giae052_response_to_reviewer_comments_original_submission.pdf]

Author's Response To Reviewer Comments

Dear Editor and Reviewers,
Thank you for your email and the opportunity to revise our manuscript, “The first high-altitude autotetraploid haplotype-resolved genome assembled (Rhododendron nivale subsp. boreale) provides new insights into mountaintop adaptation” (GIGA-D-23-00395).
These comments are valuable and helpful for revising and improving the quality of our manuscript, as well as providing important guidance for our future research. We have checked the manuscript and have tried our best to revise it carefully according to these comments. The main changes are as follows:
(1) We have entrusted our paper to a professional institution for English language polishing to improve the language and logic of the manuscript.
(2) We have emphatically adjusted the tone to show that the alpine adaptation strategy in the manuscript is speculative and needs to be verified by further biological experiments.
(3) We found that a small portion of positive selection genes was recorded incorrectly, and we are very sorry about that. To identify the positive selection signal more accurately, we have updated the detection approach. In the revision, a multi-model cross-validation strategy was used to detect positive selection genes. Although the results produced by this approach are slightly different, the overall conclusion has not changed.
(4) We have also changed the submission section to DATA NOTE to emphasize that our main contribution is the provision of an important alpine plant genetic resource.
We have marked the changes with different colors in the revised manuscript to facilitate your review of the changes (Blue: Modifications based on review comments; Green: English grammar, logic and other modifications). We earnestly appreciate the thorough work of the Editors/Reviewers, and sincerely hope that you find our responses and modifications satisfactory. Thank you again for considering our work. We hope that this version of our manuscript is suitable for publication in GigaScience.

Best regards
Sincerely yours,
Shi-Kang Shen

Reviewer #1:
Thank you for your thorough evaluation of our manuscript and for the constructive comments provided. We appreciate the time you’ve taken to review our work. We acknowledge the issues you have raised regarding the clarity of language and the logical flow of the manuscript. We understand that these are crucial for effectively communicating our research and ensuring that the scientific community can fully assess the quality and rigor of our work. To address your concerns, we made a complete revision of the manuscript:

Major Concern:
Comment: The paper is not yet ready for publication and requires a lot of writing improvement work to clarify the analyses and results.
Response: Thank you for your comment. For editors and reviewers to effectively evaluate our research, we conducted a careful examination of the manuscript, corrected typos, and improved the logic of the full text. In addition, we entrusted the manuscript to a professional agency to improve its readability.

Comment: I find that many of the claims that the authors suggest (especially concerning gene family expansion and contraction) are strongly worded and at the very least need to be toned down since they are only correlative at best.
Response: Thank you for your comment. We also realize that our study is preliminary and only speculates on high-altitude adaptation strategies. Therefore, we moderate our tone and show in the paper that our studies on adaptive strategies are speculative. In addition, we change the study to “Data Note”, showing that our study mainly provides an important genetic resource of high-altitude woody polyploid. We appreciate your attention to detail and guidance in enhancing the manuscript’s quality.

Other Concern:

Comment: Line 28 - the context and importance of R. nivale as the "first high-altitude woody flowering autotetraploid." Is missing. Why should the reader care about this very specific case study. I think some better lead up will help understand.
Response: Thank you for your comment. We have added a leading-in to the background of the abstract to make it easier for readers to quickly understand the issues of concern in this article. "As a high-altitude woody polyploid, this species occupies a distinctive role in the exploration of alpine plant adaptability." (Line 24-25)

Comment: 30 - "harbor" change to "which harbor" or "harboring"
Response: Thank you for pointing out the mistake. We have revised the sentence to include "harboring" to properly connect the relative clause, providing a more precise and readable statement. (Line 33)

Comment: 37 - ERF VII are undefined yet as a gene class.
Response: Thank you for your comment. Group VII ERFs is a phylogenetic cluster, which is conserved across angiosperms. We acknowledge that the ERF VII gene class has not been explicitly defined in the current. However, ERF VII is a widely used gene group (Abbas et al. 2022; Fan et al. 2023; Zubrycka et al. 2023). Our research is predicated on the homology with the well-studied and defined ERF VII gene family in Arabidopsis thaliana. The proposed ERF VIIs is based on sequence similarity and functional characteristics of these known genes, particularly their role in response to hypoxic conditions.
Reference:
Abbas M, Sharma G, Dambire C, et al. An oxygen-sensing mechanism for angiosperm adaptation to altitude. Nature. 2022;606:565–9.
Fan B, Liao K, Wang LN, et al. Calcium-dependent activation of CPK12 facilitates its cytoplasm-to-nucleus translocation to potentiate plant hypoxia sensing by phosphorylating ERF-VII transcription factors. Mol Plant. 2023;16:979–98.
Zubrycka A, Dambire C, Carbonare DL, et al. ERFVII action and modulation through oxygen-sensing in Arabidopsis thaliana. Nat Commun. 2023;14:4665.

Comment: 38 - meanwhile is not the best connector word here and elsewhere.
Response: Thank you for pointing out the problem of improper use of connective words. To improve the readability of the full text, we have checked the connectives in the full text and modified them accordingly according to the context.

Comment: Line 50- what do you mean by core genus. Provide a better definition.
Response: Thank you for pointing out the problem about definition. In the reworked manuscript, we have replaced "core genus" with "largest genus". (Line 55)

Comment: Line 58-62- (1) what do we know about high-altitude polyploids in general? (2) Is there any evidence for adaptive benefits? (3) Why should we care?
Response: Thank you for your comment. All information related to polyploids is presented in the third paragraph of the introduction. (1) Following polyploidy events, rapid shifts in gene expression and epigenetic modifications can bestow the polyploid with an almost instant competitive edge. (2) Although the evidence of polyploid high altitude adaptation is not completely sufficient, many studies have proved that polyploid plants have stronger abiotic stress tolerance than diploid plants. Therefore, polyploidy tends to be ecologically advantageous and occurs in variable climatic regions, such as the Qinghai-Tibet Plateau alpine and Pan-Arctic regions. (3) Comprehending these adaptive mechanisms in high-altitude polyploids not only illustrates evolutionary dynamics but also informs conservation methodologies. (Line 86-100)

Comment: Line 78-79 : Limited is repeated twice
Response: Thank you for your comment. We have modified the sentence to solve the problem of word redundancy. "Currently, the assembly of autopolyploid genomes presents significant challenges, resulting in the publication of only a select number of such genomes, including those of Medicago sativa, Saccharum spontaneum, Solanum tuberosum, and Rheum officinale." (Line 81-84)

Comment: 85 - polyploidy "event"
Response: Thank you for your carful checks. We have modified in manuscript. (Line 88)

Comment: 113- poor grammar and structure "Tetraploid was identified from the kmers present in sequencing reads (Fig. 1E)."
Response: Thank you for pointing out the problem. We have modified this sentence to "This species identified as a tetraploid based on the k-mers analysis." (Line 117-118)

Comment: 116 - great that you tried different assembly approaches. I think you should report some contig level statistics to show why you chose hifiasm. Minimum should report contig N50.
Response: Thank you for your recognition of our analytical methods. The results for each assembly approach are shown in Table S4.

Comment: 137 - LAI of reference suggests that the repeat content of the assembly is not well assembled. This can be improved by using HiFi pacbio reads. Although I understand that may not be in the scope of this manuscript however that would be an improvement for the assembly.
Response: Thank you for your suggestion to improve the assembly using HiFi PacBio reads. We agree that this approach could potentially enhance the assembly quality However, due to the extensive time and resources required for such an analysis, which includes not only closing gaps but also a complete re-run of all subsequent analyses, we are unable to include this in the current scope of our manuscript. To assess LAI more accurately, we have evaluated each haplotype independently, thereby reducing the negative impact of highly similar sequences between haplotypes on LAI regulation. The results showed that LAI of all haplotypes was greater than 14 (n1:14.78, n2:14.84, n3:14.35, n4:14.38). (Line 140-142) We have noted your recommendation as a valuable suggestion for future improvements to our assembly and will consider it for subsequent iterations of our work.

Comment: 159-163 the whole section about confirming the polyploidy using kmers should be explained better.
Response: Thank you for your comment. We have shown in the methods section how to determine auto and allotetraploid based kmers analysis. (Line 549-550). For more specific principles, please refer to the citation "[29] Ranallo-Benavidez TR, Jaron KS, Schatz MC. GenomeScope 2.0 and Smudgeplot for reference-free profiling of polyploid genomes. Nat Commun. 2020;11:1432."

Comment: 167 - I am not familiar with the term "exerted synteny" I don't think it is being used the correct way.
Response: Thank you for your comment. Upon check, we agree that the term 'exerted' may not be the most appropriate in this context. We have revised the manuscript accordingly. The text now reads: "As expected, the dot plot and syntenic blocks indicated synteny among the four haplotypes (Fig. 2A), with 20,172 gene pairs showing synteny between haplotypes 1 and 2, 20,249 between haplotypes 2 and 3, and 19,883 between haplotypes 3 and 4." (Line 169-172)

Comment: 169 - clarify what transcriptome data are being used here.
Response: Thank you for your comment. We provided relevant information of all the data in the manuscript in the Table S14, including species, data type, accession number and download address. The data generated in this study (n1, n2, n3, n4, and R. nivale subsp. boreale represent the four haplotypes and transcriptome of R. nivale subsp. boreale, respectively), are shown in Figure 2 title. (Line 969-971)

Comment: 214- incomplete sentence "In the dot plot between Vi. vinifera and R. nivale subsp. boreale (Fig. 3D, S7-8)."
Response: Thank you for pointing out the incomplete sentence. The sentence should be completed as follows: "The dot plot comparing Vi. vinifera and R. nivale subsp. boreale (Fig. 3D, S7–8), nearly every grape chromosome exhibited two highly compatible chromosomal regions in R. nivale subsp. boreale (orthologous ratio 1:2)." (Line 215-217).

Comment: 217 - the whole section on positive selection is not written well and was challenging to understand. Thus, the claims derived from these analyses are not well supported at this stage. There is no introduction to the section just an immediate dive into 44 genes. The reader is not prepared for that yet.
Response: Thank you for your valuable feedback regarding the section on positive selection. We apologize for any confusion caused by our initial presentation of the data and analysis. We acknowledge that the high-altitude adaptation mechanisms of R. nivale subsp. boreale cannot be fully resolved based on current

data. In this manuscript, our aim is to provide important high-altitude plant genomes and speculate the high-altitude adaptation strategies of Rhododendron based on existing data, providing a scientific basis for future adaptation studies. To detect the true positive selection events as much as possible, we have updated the positive selection gene recognition pathway. On the basis of aBSREL detection, the Clade Model was used to exclude genes that may not be affected by selection pressure. Finally, the positive selection sites were identified based on the intersection of the MEME and the Contrast-FEL. A gene is considered a positive selection gene when it meets all model criteria. The multi-method detection approach reduces the probability of false positive gene identification. Finally, the results of the positive selection analysis from KaKs_Calculator are integrated to collectively speculate on the adaptation mechanism. Additionally, we have revised this section to improve clarity and ensure that the narrative is more accessible. I appreciate the opportunity to enhance the quality and rigor of our manuscript. (Line 219-234; 581-604; Tables S18-S20)

Comment: 230 - 233 - can be moved to discussion
Response: Thank you for your suggestion. We very much agree with this adjustment and have moved this section to the discussion section. (Line 369-374)

Comment: 282 - "More species had approximately 140 genes." Unclear sentence and context
Response: Thank you for comment. The intent of this statement was to convey that a greater number of species were found to have the gene count of AP2/ERF close to 140 in our study. To clarify this, we have revised the sentence to read: “The majority of species in our study had gene counts of approximately 140.” (Line 283-284)

Comment: 291-295 - the whole section is missing citations and could be moved to the discussion.
Response: Thank you for your constructive feedback. We have added the appropriate references to ensure the arguments are well-founded. Additionally, we agree that the content may be better suited to the discussion part of the paper and have made the necessary adjustments to relocate the section. (Line 435-437)

Comment: 303 - citations.
Response: Thank you for your careful check. We have added the appropriate citation. The following revisions have been made to the manuscript: “Group VII ethylene response factor transcription factors (ERF VIIs) are associated with altitude adaptation [34].” (Line 291-292)

Comment: 307-310 conclusion is very strong for the evidence presented.
Response: Thank you for your comment. We understand your concern regarding the strength of the conclusion drawn from the observed gene expression. We would like to clarify that the high expression levels of these genes of R. nivale subsp. boreale are consistent with the plant’s adaptation to its natural habitat, which is characterized by low oxygen pressure. This inference is supported by the literature indicating that ERF VII genes probably are involved in the response to hypoxic conditions. However, we acknowledge that further experimental validation is needed to definitively establish the causal relationship. Therefore, we have moved it to discussion section and softened the language and said it was subject to experimental verification. We have revised the manuscript as follows: “The high expression levels of ERF VIIs in R. nivale subsp. boreale suggests a potential adaptation to low oxygen environments, which warrants further experimental investigation.” (Line 441-443)

Comment: 324 - typo : badiratE
Response: Thank you for pointing out the typo. We have corrected it. (Line 316)

Comment: 332- the distribution of the CYPs is shown? Reference a figure.
Response: Thank you for your comment. We have added the appropriate figures (S10-21), which visually represent the distribution of the AP2/ERFs, CYPs, ERF VIIs and CBFs in R. nivale subsp. boreale.

Comment: 336 - missing citations.
Response: Thank you for pointing out the missing citation. We have added the appropriate citation. (Line 330)

Comment: Overall the claims about positive selection of genes for altitude fitness should be toned down. The functional validation of the suggested genes is not clear and while the analysis might show they are under selection the role and fitness advantage is not supported by evidence presented herein.
Response: Thank you for your comment on genes for altitude fitness. We acknowledge that our results are preliminary and appreciate your suggestion to moderate the language used to describe these findings. To enhance the accuracy of analysis, we have modified our strategy of positive selection analysis to include multiple models for cross-validation, namely two branch site-based models (aBSREL and Clade Model) and two site-based models (MEME and Contrast-FEL). Additionally, we have revised the tone of the conclusion to reflect that it is a conjecture based on existing results, pending further experimental validation. Our aim is to provide valuable genetic resources and to formulate initial hypotheses regarding the high-altitude adaptation mechanisms in R. nivale subsp. boreale. In our latest revision, we have reclassified the submission as a DATA NOTE to emphasize that the primary contribution of our research is the provision of significant high-altitude genetic resources.

Comment: 431- what is anti-low temperature
Response: Thank you for your comment. We have modified it to "cold tolerance". (Line 444-445)

Comment: 444- CFBs are lowly expressed? What are the conditions of sampling? These are cold response genes.
Response: Thank you for bringing this to our attention. We recognize that the expression of CBFs is typically induced by cold conditions, and therefore, their low expression under the conditions sampled may not fully reflect their role in cold response. Therefore, we have deleted this section.

Comment: 510 - what is this analysis for Modifit?
Response: We are sorry for our careless mistakes. Thank you for your reminder. The correct name for the software is ModFit LT 5.0, and its download address has been added to the manuscript. (Line 511) ModFit LT is the open-ended modeling software for cytometry data. We use ModFit LT to process flow cytometry results (Fig. S1). (Line 506-507)

Comment: 538 - Clarify the steps you took to annotate. which transcripts were used where and for what software downstream.
Response: Thank you for your comment. We have rewritten the section based on transcriptome annotations. During our annotation, the transcriptome data were assembled using Trinity and StringTie. Next, all assembly results were fed into BRAKER3 and PASA for gene prediction and to generate Ab initio gene predictor training sets. We have made the subsequent changes to the manuscript: "In our transcriptome-based strategies, we used HISAT2 v2.2.1 [92] to align clean reads of the transcriptome with the genome. Trinity v2.14.0 [93] and StringTie v2.2.1 [94] were used to assemble transcripts. BRAKER3 [95] and PASA v2.5.2 [96] were used to predict gene structure based on the assembled transcripts and to generate ab initio gene predictor training sets." (Line 534-538)

Comment: 553 - You said you used smudgeplot at the top of the section not genomescope.
Response: Thank you for your comment. Genomescope2 and Smudgeplot are published in the same article, but they are completely different pipeline. Genomescope2 was used to estimate genome size and heterozygosity, and Smudgeplot was used to estimate genome ploidy. We used Genomescope2 to obtain an estimate of genomic heterozygosity (Table S3) and Smudgeplot to evaluate ploidy (Fig. 1E).

Comment: 556- JCVI has a better citation than the zenodo repo
Response: Thank you for your recommendation to provide a better citation for JCVI. We have updated the citation to reflect the primary literature associated with the JCVI utility libraries. The revised citation is as follows: "103. Tang H, Bowers JE, Wang X, Ming R, Alam M, Paterson AH. 2008. Synteny and collinearity in plant genomes. Science 320:486–488."

Comment: 615- BadiTate typo
Response: Thank you for pointing out the typo. We have corrected it. (Line 631)

Comment: 629 - clarify the author contributions what does "Investigation" mean.
Response: Thank you for your comment. In this study, in addition to bioinformatics analysis, cytological

experiments were used to estimate genome size (Fig. S1) and chromosome number. Gao-Ming Yang, Jie-Yu Zhang and Wen-Guang Sun provided experimental sites, reagents, and conducted experiments. Unfortunately, R. nivale subsp. boreale has a lot of small chromosomes, and despite numerous experiments, we have not yet obtained the desired results. We have modified “Investigation” to “Cytological experiment.” (Line 642-644)

Reviewer #2:
Thank you for your recognition of our work. Your suggestions are very important to us, both for improving the manuscript and our further research. We have studied these comments carefully and have made corrections as possible as we can. We hope these corrections could meet with approval. The point-by-point response is as follows:

Comment: Scientific names: Arabidopsis has been named as "Ar. thaliana" throughout the paper should be "A. thaliana".
Response: Thank you for your suggestion. We used Ar. thaliana to distinguish Ac. chinensis. Using the same abbreviation is likely to cause confusion.

Comment: Line 141: The completeness of protein coding genes were accessed by BUSCO. This is a good metric to access completeness, however sometimes BUSCO does not give us the complete picture. Hence I suggest, adding mono-exonic (single-exon) to multi- exonic (multiple-exon) ratio in the needed for further justification and support.
Response: Thank you for your valuable feedback. To address this, we calculated the ratio (0.245) of mono-exonic (single-exon) to multi- exonic (multiple-exon) genes and have added this information to the manuscript (Line 148-149). Additionally, we have provided more details about BUSCO in Table S8. This table contains BUSCO assessment results for different assembly approaches, different haplotypes, unitigs and protein-coding genes.

Comment: Line 289: The authors mention variable alpine climate for growth but do not provide any metric data related to the statement. Growing conditions with metrics like average temp difference, oxygen level and average pressure needs to be provided for better context.
Response: Thank you for your valuable feedback. We have added relevant metrics for alpine climate, including average annual temperature, air pressure and diurnal temperature differences, which are presented in the discussion section. The revised text is as follows: “Low temperature (average annual temperature below 0 °C), low partial pressure of oxygen (for every 1,000 m increase in altitude, air pressure drops by about 11%) and rapid weather changes (annual average diurnal temperature exceeding 20 °C) are the main factors limiting alpine plant survival.” (Line 422-425)

Comment: Line 303: "Previous studies have shown that ERF VIIs play an extremely important role in adapting to high-altitude environments" This statement needs citation for support.
Response: Thank you for your carefully checks. The following revisions have been made to the manuscript: “Group VII ethylene response factor transcription factors (ERF VIIs) are associated with altitude adaptation [34].” (Line 291-292)

Comment: In the discussion section author has discusses in great detail about different pathways regulating different morphological traits. But a section describing the morphological traits and the phenotypic adaptation of Rhododendron nivale subsp. boreale is necessary to better relate these pathways in the contest of this species being studied.
Response: Thank you for your insightful suggestion. We agree that a section detailing the morphological traits and phenotypic adaptations of R. nivale subsp. boreale would greatly enhance the discussion. Consequently, we have speculated potential altitudinal adaptation traits in R. nivale subsp. boreale from existing studies and have incorporated this information into the manuscript. These characteristics include plant height, leaf size, flowering changes, and seed size. We have updated the manuscript as indicated below: “In alpine environments, plants have evolved myriad morphological and physiological adaptations to contend with the rigor of high-altitude conditions [50]. R. nivale subsp. boreale native to mountaintops, typically reaches heights of less than 30 cm, with leaves that seldom exceed 5 mm in both length and width, exhibits delayed flowering, and produces seeds that are nearly indiscernible. These characteristics

are thought to be a response of Rhododendron to low temperatures at high altitudes, poor nutrition, and extremely short growth cycles [51].” (Line 408-413)

Comment: Line 444: Why CBFs and its transcription factors are relatively low expressed or absent? What does the author thinks is the reason? Further justification maybe necessary.
Response: Thank you for your comment. We are very sorry for ignoring CBFs as a class of cold response genes. The expression level of CBFs was closely related to our sampling conditions. It is possible that the gene expression observed may be influenced by various factors, such as the ambient temperature during sampling and the gene-dosage effect. Given these considerations, we cannot make reasonable speculation, and therefore, we have removed this section from the discussion.

Reviewer #3:
We would like to thank you for your professional review work, constructive comments, and valuable suggestions. Your insights have been instrumental in enhancing the quality of our research, and we have carefully considered each point to ensure our work meets high standards. We have revised our manuscript to clarify our claims and enhance the overall clarity of our work. We are confident that the revisions made will address your concerns. Followed by a point-by-point response:

Major Concern:
Comment: The authors logically and comprehensively describe their evidence to suspect these genes and pathways with relevant literature cited throughout and I applaud them for that. However, the evidence is not strong enough to support these final claims. They should be worded along the lines of, "we suspect these loci enabled the success of this species in high altitudes", or something of the sort rather than claiming they aid in survival or adaptability without having explicitly tested that fact.
Response: Thank you for acknowledging the logical structure and analytical comprehensiveness of our evidence presentation. We appreciate your suggestion to moderate the language used to describe the influence of the identified loci on the success of the species at high altitudes. We agree that without explicit testing, it is more appropriate to use tentative language. Therefore, we have revised our claims to reflect that these loci are suspected of contributing to the species’ success in high-altitude environments.

Other Concern:
Comment: Line 37: "Notably, highly expressed ERF VIIs aid survival in hypoxic mountaintop. Meanwhile, the extended families enriched in brassinosteroid biosynthesis, which enhanced adaptability to alpine weather and probably mediated by increased cytochrome P450 genes."
The authors present no experimental evidence for this claim. They did not explicitly test survival under hypoxic conditions, nor any physiological response to alpine weather.
Response: Thank you for your comment. We agree that all of these assumptions need to be tested experimentally. Therefore, we have revised the relevant sections to clarify that these are hypotheses based on gene expression patterns and known functions of these genes and gene families. The revised text now reads: “We identified extended gene families and signatures of positive selection that are involved not only in adaptation to the mountaintop ecosystem (response to stress and developmental regulation) but also in autotetraploid reproduction (meiotic stabilization). Additionally, the expression levels of the ERF VIIs were significantly higher than the mean global gene expression. We suspect that these changes have enabled the success of this species at high altitudes.” (Line 38-43)

Comment: Line 100: "and determined which genes or gene families play a role in alpine adaptation and the survival of polyploids"
Qualifying language required that you have well supported candidates but do not know if they directly play a role in adaptation or survival without testing for these phenotypes.
Response: Thank you for your comment. We acknowledge the necessity of qualifying our language regarding the role of specific genes or gene families in alpine adaptation and the survival of polyploids. We have revised to reflect that, while we have identified well-supported candidate genes, their direct involvement in adaptation or survival remains to be empirically tested. The revised statement now reads: “Based on this assembly, we identified polyploid types, deciphered whole genome duplication (WGD) events, and investigated which genes or gene families are potential candidates involved in alpine mountaintop adaptation and the survival of polyploids.” (Line 103-106)

Comment: Line 114: "The three initial assembly sizes were 2.48 Gb, 2.39 Gb and 2.40 Gb, which were assembled by Hifiasm, Canu v1.9 and Hicanu, respectively (Table S4). The assembled version of Hifiasm was used for subsequent analysis because it has higher integrity in genes and LTRs.
Thank you for providing some negative results! This helps benchmark software performance and gives the field some insight to how different assemblers handle autopolyploids.
Response: Thank you for your positive feedback. We are glad to contribute to this knowledge base.

Comment: Line 173: "A clade containing all four haplotypes of R. nivale subsp. boreale and two R. nivale was supported by 100% bootstrapping (Fig. 2 C)"
The placement of a 5th individual labeled "R. nivale subsp. boreale" was never explicitly mentioned. I assume this is all four haplotypes combined, but this needs mentioned
Response: We sincerely thank you for careful reading. The "R. nivale subsp. boreale" represents the transcriptome data generated in this study. And we have clarified in the figure title of Figure 2. (Line 969-971)

Comment: Line 175: "Therefore, four haploid chromosomes were considered to originate from the same species"
This claim is not supported by the evidence presented.
The observation of all four haplotypes clustering with one sample of R. nivale and not the other can arise from a few scenarios besides a single species origin. There could exist population structure within R. nivale from which multiple species have derived from one sub population and not the others.
Now is as good of a time as any but I do not believe in the technical distinction between auto and allo polyploids and especially not whether the progenitors belong to the same species being the delineation. I think differences between auto and allo are quantitative and the authors have done an excellent job layering analyses to address this question.
As it pertains to this claim of this genome deriving from a single species, the phylogenetic tree in Figure 2C is insufficient to claim this as a fact.
I recommend removing this statement.
Response: Thank you for recognizing our analysis of polyploid types. We completely agree with you. The difference between autopolyploids and allopolyploids should be quantitative. Otherwise, the type of polyploid hybrids in closely related species cannot be determined. Therefore, we have removed that statement.

Comment: Line 185: "1 basal angiosperm"
Though commonly referred to as a "basal" species, an extant species cannot be "basal". All extant species have been evolving for the same amount of time and contain their own derived characteristics. I recommend simply referring to this as "Amborella", or "1 species sister to all angiosperms" to keep the syntax consistent in that sentence.
Response: Thank you for your valuable feedback. We have revised to read “1 species sister to all other angiosperms (Amborella),” which accurately reflects the phylogenetic position of Amborella. (Line 188)

Comment: Line 191: "These species-specific genes were enriched in 12 KEGG pathways and 136 GO terms, such as arginine biosynthesis, nitrogen metabolism and flavonoid biosynthesis"
I cannot find where in the methods your GO enrichments are listed, but I did see not all genes were functionally annotated with a GO term. The background set to test these 369 R. nivale subsp. boreale specific genes should be all genes with a GO annotation rather than all genes in total. This is simply to test the robustness of your results which I do not expect to change, however reducing the background set is the more statistically appropriate test.
Response: Thank you for your meticulous review and valuable suggestion regarding the GO enrichment analysis. We used clusterProfiler for enrichment analysis. The background gene set used in the GO enrichment analysis was derived from the annotation result file (org.My.eg.db_1.0.tar.gz) produced in the annotation. This file contains all the genes that have been annotated by GO, and we have not made any changes. We present the analysis process as follows:

```
library(tidyverse)
library(clusterProfiler)
```

```
#KEGG
gene <- read.csv("~/enrichment /uni_gene.xls", sep="")
ee <-as.matrix(gene)
genev <-as.vector(ee)
gene2passway <- read.csv("~/enrichment / kegg_gene2passway.csv")
pathway2name <- read.delim("~/enrichment /kegg.pathway2name.tsv")
de_ekp <- enricher(genev, TERM2GENE = gene2passway, TERM2NAME = pathway2name, pvalueCutoff = 0.05, qvalueCutoff = 0.05)
de_ekp_df <- as.data.frame(de_ekp)

#GO
dir.create('R_Library', recursive = T)
install.packages('~/enrichment/org.My.eg.db_1.0.tar.gz', repos = NULL, lib = 'R_Library')
library(org.My.eg.db, lib = 'R_Library')
de_ego <- enrichGO(gene = genev, OrgDb = org.My.eg.db, keyType = 'GID', ont = 'ALL', qvalueCutoff = 0.05, pvalueCutoff = 0.05)
de_ego_df <- as.data.frame(de_ego)
```

Comment: Line 211: "In addition, the recent peak at Ks ~0.65 indicated that another polyploidy of Rhododendron, Va. darrowii and Ac. chinensis occurred ~78 Mya (Fig. 3C, S9)."
It was unclear to me both in the text and in the figure legend for Fig 3C whether the Ks distributions were of orthologs to R. nivale subsp. boreale or of paralogs within each genome. I can infer after reading some of the conclusions that these are intragenomic paralog Ks distributions but this needs made more clear in both the main text and in the figure legend.
Response: Thank you for your comment. Your inference is correct. We are very sorry for our unclear statement. We have clarified these in the text and in the figure title. The following revisions have been made to the manuscript: "In addition, the peak at Ks of the paralogs approximately 0.65 Mya suggests another polyploidization event in Rhododendron, Va. darrowii, and Ac. chinensis estimated to have occurred at approximately 78 Mya." (Line 213-214; Line 980; Fig S9 title)

Comment: Line 215: "We identified nearly every grape chromosome with two highly compatible chromosome regions in R. nivale subsp. boreale (orthologous ratio 1:2) (Fig. 3D)"
For Fig 3D, the axis labels are ambiguous. Also I assume to dot plot is between genes and not chromosomes. I may have missed where this was stated but if its absent if must be included.
Response: Thank you for your comment. Dot plot analysis is based on homologous genes, and this statement is shown in the figure title of Fig 3. As follows: "D. Homologous gene dot plots between R. nivale subsp. boreale and Vi. vinifera. The red box exemplifies the orthologous ratio of 1:2 between Vi. vinifera and R. nivale subsp. boreale." (Line 983-985)

Comment: Line 218: "A total of 44 genes were positively selected and functionally annotated based on Hyphy and kaks_calculator (Table S18; S19)"
I appreciate the inclusion of these results in the supplement but I recommend inclusion of quantitative values that indicated these genes were positively selected. I do not have experience with Hyphy so I'm not sure this is possible, but for kaks_calculator I think you can provide the Ka/Ks value for quantitative comparisons of these positively selected genes.
Response: Thank you for your suggestion to include more information for positively selected genes. We recognize that the lack of experimental validation is our key shortcoming. In the latest version of the manuscript, we have improved our analytical approach to detect positive selection events that are as real as possible. This approach eliminates possible false positive signals through cross-validation using multiple models. We used Hyphy for positive selection genetic testing based on an exploratory model. Next, we used the Clade Model to exclude genes not affected by selection pressure. Finally, we used MEME and Contrast-FEL to detect whether these potentially selected genes contain positive selection sites. Only when a gene meets all the model requirements do we consider it to be a valid positive selection gene. In addition, we have added the detailed results and methods of the selection analysis. We believe that the updated results are useful for better inferences of R. nivale subsp. boreale adaptation strategies to high altitude environments. (Line 219-234; Line 581-600; Tables S18-S20)

Comment: Line 233: "However, our results do not support this."
I think a statistical test is required to say whether the Ka/Ks distributions are not different within each homologous group for Fig S6. Otherwise the authors could rephrase this to "The distribution of Ka/Ks values do not appear different between our homologous groups (Fig S6)"
Response: Thank you for your suggestion. We have considered your recommendation to rephrase our statement, and have adjusted the text to "However, the distribution of Ka/Ks values did not differ between our homologous groups." In addition, we moved this statement to the discussion. (Line 369-374)

Comment: Line 263: "the transcript expression of homologous Group 3 was higher than that of the other groups (Fig. 5A)"
These values need normalized by the number of genes on each chromosome or by the total length of CDS as this pattern may simply reflect the number of genes.
Also it would be interesting to investigate this pattern across tissue types.
Response: Thank you for your comment. We agree that normalizing the transcript expression levels by the number of genes on each chromosome would provide a more accurate reflection of gene expression patterns. We have now included normalization based on the number of genes in our analysis and have updated Figure 5A accordingly. Additionally, we recognize the value in investigating expression patterns across different tissue types. While this was beyond the scope of our current study, we have noted it as a potential direction for future research.

Comment: Line 267: "Finally, 3,844 of the 6,388 (60.17%) single-match gene groups were identified as differential expression loci (DELs)"
(1) How were DEL determined?
(2) Were there biological replicates within each tissue?
(3) I also do not understand what conclusions are drawn from Fig 5B.
Response: We are very sorry that our unclear expression has caused your misunderstanding. (1) The identification strategy for DELs is shown in the method (Line 611-613). The method was published in the autotetraploid sugarcane genome study. Specifically, any two genes in a set of single-match genes with less than a two-fold difference in expression are defined as neutral; the other alleles are considered DELs. (2) Although our transcriptome comes from three replicates, samples are derived from a mixture of roots, stems, leaves, and buds. Expression levels in different tissues could not be analyzed. (3) As for Figure 5B, it illustrates the distribution of DELs across different homologous groups. As you mentioned in your comment, it is hard to tell the true haplotype origin of each chromosome. Thus, we used an independent analysis of each chromosome to compare differences in transcriptional expression level. In Figure 5B, we added labels for each heat map to indicate that our analysis was based on the chromosome level rather than haplotype. Each row of each heat map represents a set of single-match genes, while each column represents a chromosome. For example, in the heat map of Homologous group 1, 530 rows represent 530 sets of DEL alleles, and the colors represent differences in expression levels. The red and blue squares are scattered across different chromosomes within the homologous group, representing the distribution pattern of DELs.

Comment: Figure 6C:
What is the distribution of the "All" category when you removed genes that are not expressed. That should be a more appropriate comparison.
Response: Thank you for your advice. We have further clarified in figure 6 and the figure title. To address this, we have modified "All" to "Global Expression" to clearly show that the Figure 6C is drawn based on the expressed gene (Fig. 6C).

Comment: I was unable to access "PRJNA1040959" which is understandable as it should be private before publication but this led me to be unable to verify the availability of the data or if there were biological replicates for their expression analyses.
Response: Thank you for your comment. We provided a data access link from which the original data can be accessed (https://dataview.ncbi.nlm.nih.gov/object/PRJNA1040959?reviewer=46vvm122fdnc0s72kb0guu85fl). To obtain comprehensive transcriptional information, we collected and mixed samples from various tissues, ensuring three biological replicates.

Comment: Every phylogenetic tree in the main text and supplementary figures need explanations of how the trees were constructed including the program and methods (for example, maximum likelihood or parsimony?"
Response: Thank you for your comment. We have provided the construction methods of all the phylogenetic trees in the manuscript, including the figure title and the materials and methods section. These phylogenetic trees are constructed based on ML, and all are inferred using IQtree2 except for executing two large trees with thousands of sequences using Fasttree.

Comment: Some of conclusions of autopolyploid origin look at genome-wide patterns. I think some of them require a chromosome by chromosome approach like the expression analysis. Though the genome is phased, it is not phased across chromosomes, you do not know if homologous group 1 n1 is from the same parent as homologous group 5 n1.
Response: We could not agree with you more. We also expect to explore the origin of autopolyploids using a chromosome-by-chromosome approach. However, the lack of chromosome-level genomes in closely related species limits this approach. As the number of genetic resources increases, it will become more convenient and accurate to identify the origin of haplotypes in polyploids. For the level of gene expression, we have used a chromosome-by-chromosome process for analysis, such as allelic expression difference analysis (Fig. 5B).

Comment: Group 3 CBF are not monophyletic Fig S12, does this change any of your findings / is it biologically interesting?
Response: We are very sorry for our carelessness. Group III and Group IV should be combined. We have made changes accordingly (Fig. S24). Group III seem to have been split into two clades in the Ericaceae. Our modifications are as follows: “Phylogenetic analysis indicated that CBFs of Rhododendron were divided into 3 groups (Fig. S24).” (Line 313-314)

Comment: Line 457: "The presence of multiple alleles indicated that the expression level of R. nivale subsp. boreale is probably higher than that of the diploid ancestor"
I do not believe this result is supported by the data presented without being able to test the diploid ancestor. How do you know expression levels have not globally and proportionally reduced to compensate for the increase in copy number?
Response: Thank you for your comment. We acknowledge that our statement regarding the expression levels of R. nivale subsp. boreale compared to the diploid ancestor was speculative. Without direct expression data from the diploid ancestor, we cannot conclusively determine if the observed multiple alleles correlate with higher expression levels. Hence, we have removed this statement.
