# Supplementary material for: The first high-altitude autotetraploid haplotype-resolved genome assembled (Rhododendron nivale subsp. boreale) provides new insights into mountaintop adaptation
Source: Gigascience. 2024 Aug 7;13:giae052. doi: 10.1093/gigascience/giae052 (PMC11304948; doi:10.1093/gigascience/giae052)
Supplement: giae052_Response_to_Reviewer_Comments_Revision_1 [file giae052_response_to_reviewer_comments_revision_1.pdf]

Author's Response To Reviewer Comments

Dear Editor and Reviewers,

Thank you for the feedback on our manuscript "The first high-altitude autotetraploid haplotype-resolved genome assembled (Rhododendron nivale subsp. boreale) provides new insights into mountaintop adaptation " (GIGA-D-23-00395R1).
In this version, we double-checked all the data and conclusions and improved our manuscript based on reviewer comments. Additionally, we have submitted the genome sequences to the National Genomics Data Center (NGDC) and included the accession number (GWHETKQ00000000.1) in the Data Availability section.
We have marked the changes in blue in the revised manuscript to facilitate your review. Thank you again for considering our work. We hope this manuscript meets the requirements for publication at Gigascience.

Best regards
Sincerely yours,
Shi-Kang Shen

Reviewer #1:
Thank you for reviewing our manuscript. We have made improvements to the manuscript in response to your comments. The point-by-point response follows:

> Comment: line 42 - Spell out the abbreviation for Group VII Ethylene Response Factors in the abstract.
>> Response: Done. "Additionally, the expression levels of the (Group VII ethylene response factor transcription factors) ERF VIIs were significantly higher than the mean global gene expression." (line 42)

> Comment: line 112- what mountain top, I know this is in the methods but needs clarifying here too.
>> Response: We have clarified this part. "R. nivale subsp. boreale samples were collected from the alpine region at an altitude of 4300 m, treated with liquid nitrogen, and sequenced (Fig. 1A). " (line 113)

> Comment: Line 119 - The total size of the assembly doesn't differ much between the different strategies - report the contig N50 in the main text which will tell you reader how well the assemblers performed. Looking at table S4 HiCanu might have performed better than Hifiasm. Even though I also prefer Hifiasm, why did you choose that over the longer and less contigs in hicanu? you say better integrity of genes and LTRs but you don't present the results for this or point to supplement here.
>> Response: Thank you for your comment. Our LAI assessment of assemblies using different approaches was based on the whole genome, including four haplotypes. Although such an assessment may be inaccurate due to the influence of identical sequences between subgenomes, we believe it still reflects the continuity of the assemblies. We have included these results in the Supplementary Table and described the datasets used. The LAI evaluations for the assemblies using different approaches are as follows: Hifiasm (LAI=12.19), HiCanu (LAI=9.27), Canu (LAI=11.31). (Table S4)

> Comment: Line 148 - the ratio of single exonic needs the word "genes".
>> Response: Done. "The ratio of mono-exonic (single-exon) genes to multi-exonic (multiple-exon) genes was 0.245." (line 149-150)

> Comment: line 215- "In" the dot plot...
>> Response: Done. "In the dot plot comparing Vi. vinifera and R. nivale subsp. boreale (Fig. 3D, S7–8) …" (line 216)

> Comment: Line 227 -These two genes? Which genes? I think something is missing here.
>> Response: We have revised this sentence. Apologies for the oversight, there are four genes here, not two. The corrected text is as follows: "Four genes exhibited no significant positive selection sites. Finally, 16 genes met all model criteria and were identified as PSGs (Table. S19)". (line 228-230)

> Comment: line 287 - consistent with previous research - citation needed
>> Response: Done. "Consistent with previous research [33] …" (line 289)

> Comment: Line 447 - probably play important roles? I think "might" is more appropriate
>> Response: We have changed "probably" to "might". "Our positive selection analysis results suggested that M3K1 and CNGC1, which are associated with the MAPK cascade and Ca2+ signal transduction, might play important roles in low-temperature adaptation." (line 448-450)

> Comment: line 468 - change "likely serves" to "may serve"
>> Response: We have replaced "likely serves" with "may serve". "Notably, TOP3α, an important gene related to the BTR complex, exhibits positive selection in R. nivale subsp. boreale and may serve as a key factor in promoting accurate chromosomal segregation during meiosis in autopolyploids." (line 469-471)

> Comment: line 470 - grammar. and too strong tone.
>> Response: We have checked the grammar and softened the tone. The corrected text is as follows: "Our results revealed that R. nivale subsp. boreale distributed on the mountaintop is an autotetraploid, which might be mediated by the harsh environment at high altitudes." (line 472-473)

> Comment: line 476 - what do you mean here by dominant gene expression pattern? clarify.
>> Response: Thank you for your comment. We have clarified this sentence. The revised version is as follows: "Polyploidization likely plays an important role in mountaintop survival because increased gene dosage can lead to enhanced stress tolerance, greater genetic diversity, and the potential for novel traits that enhance adaptability [18]." (line 478-481)

Reviewer #3:
Thank you for reviewing our manuscript. We have made improvements to the manuscript in response to your comments. The point-by-point response follows:

> Comment: Abstract: "As a high-altitude woody polyploid, this species plays a distinct role in the adaptability of alpine plants"
- This plant does not affect the adaptability of other plants, it can tell us something about how plants adapt to alpine environments however. I'm sure thats what the authors intended. Suggested revision:
"As a high-altitude woody polyploid, this species may serve as a model to understand how plants adapt to alpine environments"
>> Response: Thank you for your suggestion. We have made the modification. (line 24-25)

> Comment: Line 118: "k-mers" should just be "k-mer"
>> Response: Done. "This species was identified as a tetraploid based on k-mer analysis (Fig. 1E)." (line 119)
