# Supplementary material for: The first high-altitude autotetraploid haplotype-resolved genome assembled (Rhododendron nivale subsp. boreale) provides new insights into mountaintop adaptation
Source: Gigascience. 2024 Aug 7;13:giae052. doi: 10.1093/gigascience/giae052 (PMC11304948; doi:10.1093/gigascience/giae052)
Supplement: giae052_Reviewer_1_Report_Original_Submission — Ben Mansfeld, PhD -- 2/21/2024 Reviewed [file giae052_reviewer_1_report_original_submission.pdf]

Reviewer Report

Title: The first high-altitude autotetraploid haplotype-resolved genome assembled (Rhododendron nivale subsp. boreale) provides new insights into mountaintop adaptation

Version: Original Submission **Date:** 2/21/2024

Reviewer name: Ben Mansfeld, PhD

Reviewer Comments to Author:

The manuscript reports a genome assembly and comparative genomic analyses for the high altitude autopolyploid Rhododendron nivale subsp. boreale. The authors assemble and resolve the a relatively contiguous tetraploid genome for the species using PacBio CSS and other technologies. After assembly was completed, the authors pursued several evolutionary and comparative genomics analyses to identify components that may contribute to the species success at high altitudes and high UV stress, including but not limited to expansion and contraction of certain gene families. Generally, the assembly and analysis of the genome for R. nivale is an important advancement for the field, from an evolutionary biology standpoint. However, the paper is not yet ready for publication and requires a lot of writing improvement work to clarify the analyses and results. Additionally, I find that many of the claims that the authors suggest (especially concerning gene family expansion and contraction) are strongly worded and at the very least need to be toned down since they are only correlative at best.Overall, the language is not at the level that it needs to be for publication. There are many fragmented sentences, type-o's and the use of connection words to clarify context needs to improve. For example, the word "Meanwhile" which is repeated multiple times often not used correctly as a connector word. In other cases, there are sentences that make claims with out properly citing sources. I fully respect that the authors may not have English as a first language and thus this critique is not personal but merely professional. Unfortunately, the quality of the writing takes away from the work, making it challenging to assess the quality and rigor of the science. I think at first, before the manuscript is ready for any type of re-submission it needs work on the writing and flow.Some specific comments:Abstract:Line 28 - the context and importance of of R. nivale as the "first high-altitude woody flowering autotetraploid." Is missing. Why should the reader care about this very specific case study. I think some better lead up will help understand.30 - "harbor" change to "which harbor" or "harboring"37 - ERF VII are undefined yet as a gene class.38 - meanwhile is not the best connector word here and elsewhere.Introduction:Line 50- what do you mean by core genus. Provide a better definition.Line 58-62- what do we know about high-altitude polyploids in general? Is there any evidence for adaptive benefits? Why should we care?Line 78-79 : Limited is repeated twice85 - polyploidy "event"Results:113- poor grammar and structure "Tetraploid was identified from the kmers present in sequencing reads (Fig. 1E)."116 - great that you tried different assembly approaches. I think you should report some contig level statistics to show why you chose hifiasm. Minimum should report contig N50.137 - LAI of reference suggests that the repeat content of the assembly is not well assembled. This can be improved by using HiFi pacbio reads. Although I understand that may not be in the scope of this manuscript however that would be an improvement for the assembly.159-163 the whole section about confirming the polyploidy using kmers

should be explained better.167 - I am not familiar with the term "exerted synteny" I don't think it is being used the correct way.169 - clarify what transcriptome data are being used here.214- incomplete sentence "In the dot plot between Vi. vinifera and R. nivale subsp. boreale (Fig. 3D, S7-8)."217 - the whole section on positive selection is not written well and was challenging to understand. Thus, the claims derived from these analyses are not well supported at this stage.There is no introduction to the section just an immediate dive into 44 genes. The reader is not prepared for that yet.230 - 233 - can be moved to discussion282 - "More species had approximately 140 genes." Unclear sentence and context291-295 - the whole section is missing citations and could be moved to the discussion.303 - citations.307-310 conclusion is very strong for the evidence presented.324 - typo : badiratE332- the distribution of the CYPs is shown? Reference a figure.336 - missing citations.Discussion:Overall the claims about positive selection of genes for altitude fitness should be toned down. The functional validation of the suggested genes is not clear and while the analysis might show they are under selection the role and fitness advantage is not supported by evidence presented herein.431- what is anti-low temperature444- CFBs are lowly expressed? What are the conditions of sampling? These are cold response genes.Methods:510 - what is this analysis for Modifit?538 - Clarify the steps you took to annotate. which transcripts were used where and for what software downstream553 - You said you used smudgeplot at the top of the section not genomescope.556- JCVI has a better citation than the zenodo repo615- BadiTate typo629 - clarify the author contributions what does "Investigation" mean.

Level of Interest

Please indicate how interesting you found the manuscript: Choose an item.

Quality of Written English

Please indicate the quality of language in the manuscript: Choose an item.

Declaration of Competing Interests

Please complete a declaration of competing interests, considering the following questions:

- Have you in the past five years received reimbursements, fees, funding, or salary from an organisation that may in any way gain or lose financially from the publication of this manuscript, either now or in the future?
- Do you hold any stocks or shares in an organisation that may in any way gain or lose financially from the publication of this manuscript, either now or in the future?
- Do you hold or are you currently applying for any patents relating to the content of the manuscript?
- Have you received reimbursements, fees, funding, or salary from an organization that holds or has applied for patents relating to the content of the manuscript?
- Do you have any other financial competing interests?
- Do you have any non-financial competing interests in relation to this paper?

If you can answer no to all of the above, write 'I declare that I have no competing interests' below. If your reply is yes to any, please give details below.

I declare that I have no competing interests

I agree to the open peer review policy of the journal. I understand that my name will be included on my report to the authors and, if the manuscript is accepted for publication, my named report including any attachments I upload will be posted on the website along with the authors' responses. I agree for my report to be made available under an Open Access Creative Commons CC-BY license (http://creativecommons.org/licenses/by/4.0/). I understand that any comments which I do not wish to be included in my named report can be included as confidential comments to the editors, which will not be published.

Choose an item.

To further support our reviewers, we have joined with Publons, where you can gain additional credit to further highlight your hard work (see: https://publons.com/journal/530/gigascience). On publication of this paper, your review will be automatically added to Publons, you can then choose whether or not to claim your Publons credit. I understand this statement.

Yes Choose an item.
