# Supplementary material for: The first high-altitude autotetraploid haplotype-resolved genome assembled (Rhododendron nivale subsp. boreale) provides new insights into mountaintop adaptation
Source: Gigascience. 2024 Aug 7;13:giae052. doi: 10.1093/gigascience/giae052 (PMC11304948; doi:10.1093/gigascience/giae052)
Supplement: giae052_Reviewer_1_Report_Revision_1 — Ben Mansfeld, PhD -- 5/15/2024 Reviewed [file giae052_reviewer_1_report_revision_1.pdf]

Reviewer Report

Title: The first high-altitude autotetraploid haplotype-resolved genome assembled (Rhododendron nivale subsp. boreale) provides new insights into mountaintop adaptation

Version: Revision 1 **Date:** 5/15/2024

Reviewer name: Ben Mansfeld, PhD

Reviewer Comments to Author:

The manuscript is much improved. Thank you for trying to address most of the points suggested by the reviewers.Overall, I still think that the tone of the conclusions need to be soften. Words such as "likely" should be changed to "may" etc. see comments below.After some more modifications I think the piece can be accepted at the new DATA NOTE level.Here are some minor comments that still need to be addressed:line 42 - Spell out the abbreviation for Group VII Ethylene Response Factors in the abstract.Line 112- what mountain top, I know this is in the methods but needs clarifying here too.Line 119 - The total size of the assembly doesn't differ much between the different strategies - report the contig N50 in the main text which will tell you reader how well the assemblers performed. Looking at table S4 HiCanu might have performed better than Hifiasm. Even though I also prefer Hifiasm, why did you choose that over the longer and less contigs in hicanu? you say better integrity of genes and LTRs but you don't present the results for this or point to supplement here.Line 148 - the ratio of single exonic needs the word "genes".line 215- "In" the dot plot...Line 227 -These two genes? Which genes? I think something is missing here.line 287 - consistent with previous research - citation neededLine 447 - probably play important roles? I think "might" is more appropriateline 468 - change "likely serves" to "may serve"line 470 - grammar. and too strong tone.line 476 - what do you mean here by dominant gene expression pattern? clarify.

Level of Interest

Please indicate how interesting you found the manuscript: Choose an item.

Quality of Written English

Please indicate the quality of language in the manuscript: Choose an item.

Declaration of Competing Interests

Please complete a declaration of competing interests, considering the following questions:

- Have you in the past five years received reimbursements, fees, funding, or salary from an organisation that may in any way gain or lose financially from the publication of this manuscript, either now or in the future?

- Do you hold any stocks or shares in an organisation that may in any way gain or lose financially from the publication of this manuscript, either now or in the future?
- Do you hold or are you currently applying for any patents relating to the content of the manuscript?
- Have you received reimbursements, fees, funding, or salary from an organization that holds or has applied for patents relating to the content of the manuscript?
- Do you have any other financial competing interests?
- Do you have any non-financial competing interests in relation to this paper?

If you can answer no to all of the above, write 'I declare that I have no competing interests' below. If your reply is yes to any, please give details below.

I declare that I have no competing interests

I agree to the open peer review policy of the journal. I understand that my name will be included on my report to the authors and, if the manuscript is accepted for publication, my named report including any attachments I upload will be posted on the website along with the authors' responses. I agree for my report to be made available under an Open Access Creative Commons CC-BY license (http://creativecommons.org/licenses/by/4.0/). I understand that any comments which I do not wish to be included in my named report can be included as confidential comments to the editors, which will not be published.

Choose an item.

To further support our reviewers, we have joined with Publons, where you can gain additional credit to further highlight your hard work (see: https://publons.com/journal/530/gigascience). On publication of this paper, your review will be automatically added to Publons, you can then choose whether or not to claim your Publons credit. I understand this statement.

Yes Choose an item.
