# Supplementary material for: The first high-altitude autotetraploid haplotype-resolved genome assembled (Rhododendron nivale subsp. boreale) provides new insights into mountaintop adaptation
Source: Gigascience. 2024 Aug 7;13:giae052. doi: 10.1093/gigascience/giae052 (PMC11304948; doi:10.1093/gigascience/giae052)
Supplement: giae052_Reviewer_2_Report_Original_Submission — Lav Yadav -- 3/1/2024 Reviewed [file giae052_reviewer_2_report_original_submission.pdf]

Reviewer Report

Title: The first high-altitude autotetraploid haplotype-resolved genome assembled (Rhododendron nivale subsp. boreale) provides new insights into mountaintop adaptation

Version: Original Submission **Date:** 3/1/2024

Reviewer name: Lav Yadav

Reviewer Comments to Author:

Overall a very well written paper.Scientific names: Arabidopsis has been named as "Ar. thaliana" throughout the paper should be "A. thaliana".Line 141: The completeness of protein coding genes were accessed by BUSCO. This is a good metric to access completeness, however sometimes BUSCO does not give us the complete picture. Hence I suggest, adding monoâ€�exonic (singleâ€�exon) to multiâ€�exonic (multipleâ€�exon) ratio in the needed for further justification and support.Line 289: The authors mention variable alpine climate for growth but do not provide any metric data related to the statement. Growing conditions with metrics like average temp difference, oxygen level and average pressure needs to be provided for better context.Line 303: "Previous studies have shown that ERF VIIs play an extremely important role in adapting to high-altitude environments" This statement needs citation for support.In the discussion section author has discusses in great detail about different pathways regulating different morphological traits. But a section describing the morphological traits and the phenotypic adaptation of Rhododendron nivale subsp. boreale is necessary to better relate these pathways in the contest of this species being studied.Line 444: Why CBFs and its transcription factors are relatively low expressed or absent? What does the author thinks is the reason? Further justification maybe necessary.

Level of Interest

Please indicate how interesting you found the manuscript: Choose an item.

Quality of Written English

Please indicate the quality of language in the manuscript: Choose an item.

Declaration of Competing Interests

Please complete a declaration of competing interests, considering the following questions:

- Have you in the past five years received reimbursements, fees, funding, or salary from an organisation that may in any way gain or lose financially from the publication of this manuscript, either now or in the future?
- Do you hold any stocks or shares in an organisation that may in any way gain or lose financially from the publication of this manuscript, either now or in the future?

- Do you hold or are you currently applying for any patents relating to the content of the manuscript?
- Have you received reimbursements, fees, funding, or salary from an organization that holds or has applied for patents relating to the content of the manuscript?
- Do you have any other financial competing interests?
- Do you have any non-financial competing interests in relation to this paper?

If you can answer no to all of the above, write 'I declare that I have no competing interests' below. If your reply is yes to any, please give details below.

I declare that I have no competing interests

I agree to the open peer review policy of the journal. I understand that my name will be included on my report to the authors and, if the manuscript is accepted for publication, my named report including any attachments I upload will be posted on the website along with the authors' responses. I agree for my report to be made available under an Open Access Creative Commons CC-BY license (http://creativecommons.org/licenses/by/4.0/). I understand that any comments which I do not wish to be included in my named report can be included as confidential comments to the editors, which will not be published.

Choose an item.

To further support our reviewers, we have joined with Publons, where you can gain additional credit to further highlight your hard work (see: https://publons.com/journal/530/gigascience). On publication of this paper, your review will be automatically added to Publons, you can then choose whether or not to claim your Publons credit. I understand this statement.

Yes Choose an item.
