# Supplementary material for: The first high-altitude autotetraploid haplotype-resolved genome assembled (Rhododendron nivale subsp. boreale) provides new insights into mountaintop adaptation
Source: Gigascience. 2024 Aug 7;13:giae052. doi: 10.1093/gigascience/giae052 (PMC11304948; doi:10.1093/gigascience/giae052)
Supplement: giae052_Reviewer_3_Report_Original_Submission — Alan E. Yocca -- 3/5/2024 Reviewed [file giae052_reviewer_3_report_original_submission.pdf]

Reviewer Report

Title: The first high-altitude autotetraploid haplotype-resolved genome assembled (Rhododendron nivale subsp. boreale) provides new insights into mountaintop adaptation

Version: Original Submission **Date:** 3/5/2024

Reviewer name: Alan E. Yocca

Reviewer Comments to Author:

This manuscript presents a contemporary quality genome assembly that was assembled and annotated according to the latest advancements in genomics. The authors present several lines of evidence suggesting an autopolyploid origin of this species. The analyses are thorough and consistent with best practices in phylogenomics. My concerns mostly relate to the way some of their claims are presented, some curiosity of how patterns are exhibited at the chromosome-level (rather than genome-wide), and the clarity of some analyses.Line 37: "Notably, highly expressed ERF VIIs aid survival in hypoxic mountaintop. Meanwhile, the extended families enriched in brassinosteroid biosynthesis, which enhanced adaptability to alpine weather and probably mediated by increased cytochrome P450 genes."The authors present no experimental evidence for this claim. They did not explicitly test survival under hypoxic conditions, nor any physiological response to alpine weatherThe authors logically and comprehensively describe their evidence to suspect these genes and pathways with relevant literature cited throughout and I applaud them for that. However, the evidence is not strong enough to support these final claims. They should be worded along the lines of, "we suspect these loci enabled the success of this species in high altitudes", or something of the sort rather than claiming they aid in survival or adaptability without having explicitly tested that fact.Most of the discussion has the correct qualifying language which is great!Line 100: "and determined which genes or gene families play a role in alpine adaptation and the survival of polyploids"Qualifying language required that you have well supported candidates but do not know if they directly play a role in adaptation or survival without testing for these phenotypes.Line 114: "The three initial assembly sizes were 2.48 Gb, 2.39 Gb and 2.40 Gb, which were assembled by Hifiasm, Canu v1.9 and Hicanu, respectively (Table S4). The assembled version of Hifiasm was used for subsequent analysis because it has higher integrity in genes and LTRs.Thank you for providing some negative results! This helps benchmark software performance and gives the field some insight to how different assemblers handle autopolyploidsLine 173: "A clade containing all four haplotypes of R. nivale subsp. boreale and two R. nivale was supported by 100% bootstrapping (Fig. 2 C)"The placement of a 5th individual labeled "R. nivale subsp. boreale" was never explicitly mentioned. I assume this is all four haplotypes combined, but this needs mentionedLine 175: "Therefore, four haploid chromosomes were considered to originate from the same species"This claim is not supported by the evidence presented.The observation of all four haplotypes clustering with one sample of R. nivale and not the other can arise from a few scenarios besides a single species origin. There could exist population structure within R. nivale from which multiple species have derived from one sub population and not the others.Now is as good of a time as any but I do not believe in the technical distinction between auto and allo polyploids and especially not whether the progenitors belong to the same species being the

delineation. I think differences between auto and allo are quantitative and the authors have done an excellent job layering analyses to address this questionAs it pertains to this claim of this genome deriving from a single species, the phylogenetic tree in Figure 2C is insufficient to claim this as a fact.I recommend removing this statementLine 185: "1 basal angiosperm"Though commonly referred to as a "basal" species, an extant species cannot be "basal". All extant species have been evolving for the same amount of time and contain their own derived characteristics. I recommend simply referring to this as "Amborella", or "1 species sister to all angiosperms" to keep the syntax consistent in that sentence.Line 191: "These species-specific genes were enriched in 12 KEGG pathways and 136 GO terms, such as arginine biosynthesis, nitrogen metabolism and flavonoid biosynthesis"I cannot find where in the methods your GO enrichments are listed, but I did see not all genes were functionally annotated with a GO term. The background set to test these 369 R. nivale subsp. boreale specific genes should be all genes with a GO annotation rather than all genes in total. This is simply to test the robustness of your results which I do not expect to change, however reducing the background set is the more statistically appropriate test.Line 211: "In addition, the recent peak at Ks ~0.65 indicated that another polyploidy of Rhododendron, Va. darrowii and Ac. chinensis occurred ~78 Mya (Fig. 3C, S9)."It was unclear to me both in the text and in the figure legend for Fig 3C whether the Ks distributions were of orthologs to R. nivale subsp. boreale or of paralogs within each genome. I can infer after reading some of the conclusions that these are intragenomic paralog Ks distributions but this needs made more clear in both the main text and in the figure legendLine 215: "We identified nearly every grape chromosome with two highly compatible chromosome regions in R. nivale subsp. boreale (orthologous ratio 1:2) (Fig. 3D)"For Fig 3D, the axis labels are ambiguous. Also I assume to dot plot is between genes and not chromosomes. I may have missed where this was stated but if its absent if must be included.Line 218: "A total of 44 genes were positively selected and functionally annotated based on Hyphy and kaks_calculator (Table S18; S19)"I appreciate the inclusion of these results in the supplement but I recommend inclusion of quantitative values that indicated these genes were positively selected. I do not have experience with Hyphy so I'm not sure this is possible, but for kaks_calculator I think you can provide the Ka/Ks value for quantitative comparisons of these positively selected genes.Line 233: "However, our results do not support this."I think a statistical test is required to say whether the Ka/Ks distributions are not different within each homologous group for Fig S6. Otherwise the authors could rephrase this to "The distribution of Ka/Ks values do not appear different between our homologous groups (Fig S6)"Line 263: "the transcript expression of homologous Group 3 was higher than that of the other groups (Fig. 5A)"These values need normalized by the number of genes on each chromosome or by the total length of CDS as this pattern may simply reflect the number of genes.Also it would be interesting to investigate this pattern across tissue typesLine 267: "Finally, 3,844 of the 6,388 (60.17%) single-match gene groups were identified as differential expression loci (DELs)"How were DEL determined?Were there biological replicates within each tissue?I also do not understand what conclusions are drawn from Fig 5BFigure 6C:What is the distribution of the "All" category when you removed genes that are not expressed. That should be a more appropriate comparisonOther comments:I was unable to access "PRJNA1040959" which is understandable as it should be private before publication but this led me to be unable to verify the availability of the data or if there were biological replicates for their expression analyses.Every phylogenetic tree in the main text and supplementary figures need explanations of how the trees were constructed including the program and methods (for example, maximum likelihood or

parsimony?"Some of conclusions of autopolyploid origin look at genome-wide patterns. I think some of them require a chromosome by chromosome approach like the expression analysis. Though the genome is phased, it is not phased across chromosomes, you do not know if homologous group 1 n1 is from the same parent as homologous group 5 n1.Group 3 CBF are not monophyletic Fig S12, does this change any of your findings / is it biologically interesting?Line 457: "The presence of multiple alleles indicated that the expression level of R. nivale subsp. boreale is probably higher than that of the diploid ancestor"I do not believe this result is supported by the data presented without being able to test the diploid ancestor. How do you know expression levels have not globally and proportionally reduced to compensate for the increase in copy number?

Level of Interest

Please indicate how interesting you found the manuscript: Choose an item.

Quality of Written English

Please indicate the quality of language in the manuscript: Choose an item.

Declaration of Competing Interests

Please complete a declaration of competing interests, considering the following questions:

- Have you in the past five years received reimbursements, fees, funding, or salary from an organisation that may in any way gain or lose financially from the publication of this manuscript, either now or in the future?
- Do you hold any stocks or shares in an organisation that may in any way gain or lose financially from the publication of this manuscript, either now or in the future?
- Do you hold or are you currently applying for any patents relating to the content of the manuscript?
- Have you received reimbursements, fees, funding, or salary from an organization that holds or has applied for patents relating to the content of the manuscript?
- Do you have any other financial competing interests?
- Do you have any non-financial competing interests in relation to this paper?

If you can answer no to all of the above, write 'I declare that I have no competing interests' below. If your reply is yes to any, please give details below.

'I declare that I have no competing interests

I agree to the open peer review policy of the journal. I understand that my name will be included on my report to the authors and, if the manuscript is accepted for publication, my named report including any attachments I upload will be posted on the website along with the authors' responses. I agree for my

report to be made available under an Open Access Creative Commons CC-BY license (http://creativecommons.org/licenses/by/4.0/). I understand that any comments which I do not wish to be included in my named report can be included as confidential comments to the editors, which will not be published.

Choose an item.

To further support our reviewers, we have joined with Publons, where you can gain additional credit to further highlight your hard work (see: https://publons.com/journal/530/gigascience). On publication of this paper, your review will be automatically added to Publons, you can then choose whether or not to claim your Publons credit. I understand this statement.

Yes Choose an item.
